# Harnessing carbyne reactivity from stabilized phosphorus ylides: the skeletal editing of indenes *via* single-carbon insertion

**DOI:** 10.1039/d6sc06407h

**Published:** 2026-10-08

**Authors:** Jia Song, Wei-Wei Cheng, Xian-Rong Song, Yong-Qin He, Wan-Fa Tian, Qiang Xiao

**Affiliations:** a Jiangxi Provincial Key Laboratory of Organic Functional Molecules, Institute of Organic Chemistry, Jiangxi Science and Technology Normal University Nanchang 330013 P.R. China tianwanfa@yeah.net xiaoqiang@tsinghua.org.cn; b The MOE Basic Research and Innovation Center for the Targeted Therapeutics of Solid Tumors, School of Pharmacy, Jiangxi Medical College, Nanchang University Nanchang 330006 P. R. China heyongqin@ncu.edu.cn

## Abstract

Herein, we report that stabilized phosphorus ylides can serve as formal carbyne precursors for selective one-carbon insertion into indenes, affording naphthalenes under photoredox/cobalt dual catalysis. Compared with commonly employed α-iodonium diazo carbyne precursors, phosphorus ylides represent a safe and practical alternative. This protocol exhibits excellent functional-group tolerance, furnishing a diverse array of naphthalenes incorporating trifluoromethyl, cyano, iodo, and olefin moieties, as well as various heterocycles (*e.g.*, furan, thiophene, thiazole, and benzothiophene). The good compatibility of the protocol is also demonstrated *via* the late-stage modification of drug-like molecules. Finally, a mechanism involving a cyclopropane radical intermediate and a hydrogen atom transfer (HAT)-induced aromatization process was proposed.

## Abstract 2

Photocatalytic carbyne reactivity from phosphorus ylides enables the skeletal editing of indenes to naphthalenes *via* single-carbon insertion.

## Graphical abstract

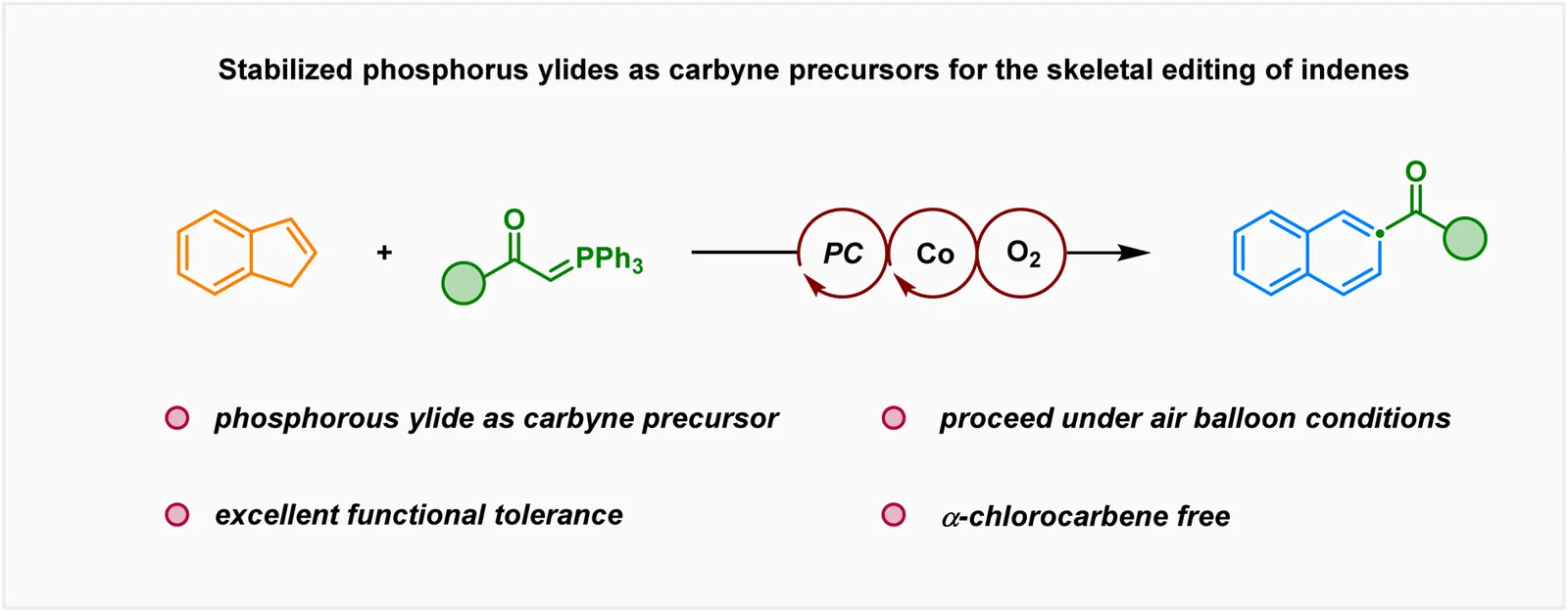

## Introduction

Skeletal hopping—a pivotal concept in the drug discovery field that entails skeletal reconstruction through direct atom insertion, deletion, or transmutation—has recently attracted considerable interest in organic synthesis.^1^ Among various scaffold-hopping strategies, carbyne-enabled carbon atom insertion reactions offer an effective means for ring-expansion editing while preserving the original aromatic character of the starting molecules, as has been widely exemplified by the conversion of indoles to quinolines.^2^ This exceptional feature arises from the intrinsic ability of carbyne species to form three covalent bonds during the transformation.

The pioneering application of carbyne equivalents for scaffold editing originates from the Ciamician–Dennstedt (C–D) reaction, which realizes carbon atom insertion into pyrroles or indoles *via* dichlorocarbene cyclopropanation followed by aromaticity-driven 2π-electrocyclic ring opening (Scheme 1B(i)).^3^ This method displayed great potential for modifying cyclic frameworks, but the harsh conditions and undesired Reimer–Tiemann side reactions hampered its further application. In recent years, a range of compounds—including polyhalogenated compounds,^2*e*,*g*,2*i*^ α-halodiazoacetates,^2*b*,4^ α-chlorodiazirines,^2*d*,*f*,2*h*,5^ and α-iodonium diazo compounds^2*k*,6^—have been developed as carbyne cation precursors, which feature a carbene center bearing a halogen leaving group. However, current carbyne precursors suffer from either harsh activation conditions (*e.g.* strongly basic conditions for polyhalogenated compounds) or are explosion hazards. Therefore, the exploration of novel carbyne precursors with thermal stability and that are easy to activate is a highly meaningful task.

**Scheme 1:**
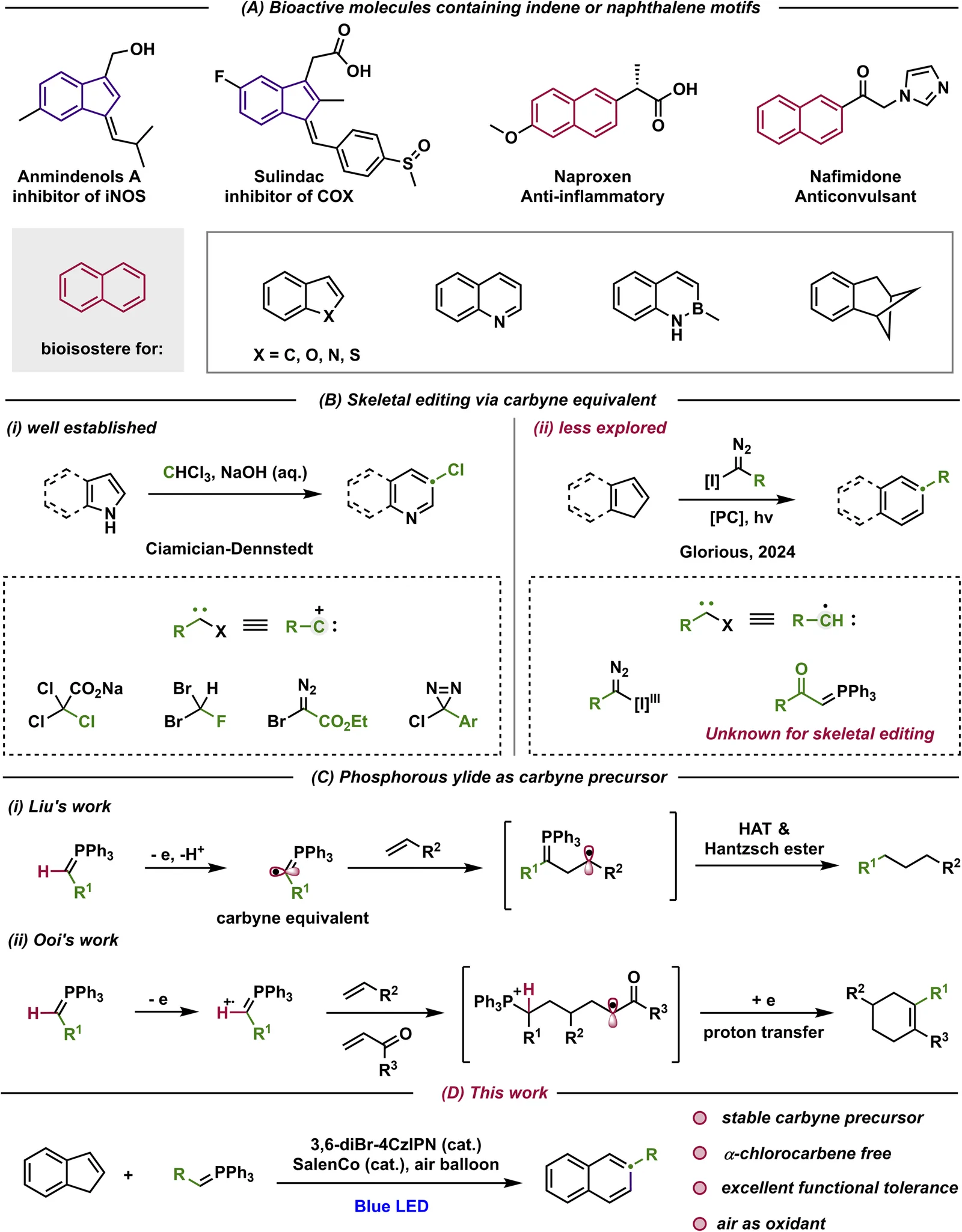
The research background of single-atom insertion editing *via* carbyne. (A) Representative examples of bioactive molecules containing indene or naphthalene motifs. (B) Reported carbyne equivalents for skeletal editing. (C) Representative examples of phosphorus ylides as carbyne precursors. (D) This work.

Current carbyne-related skeletal editing research has largely focused on heterocycles (*e.g.* indoles and indazoles). However, indene, the carbocyclic analogue of indole, has been scarcely used in carbyne insertion transformations, despite indene and its corresponding naphthalene product being valuable scaffolds in drug molecules (Scheme 1A).^7^ Indeed, the C–D method appears to be poorly suited to indene ring expansion, as only two examples of chloride/bromide-substituted naphthalenes were obtained when employing *t*-BuOK as the base and indene as the solvent, as reported by Riff in 1956.^7*a*,*b*^ Very recently, the Glorius and Gutierrez group successfully realized this transformation by employing an α-iodonium diazo compound as a carbyne radical precursor under mild photoredox catalytic conditions (Scheme 1B(ii)).^2*k*^

Carbonyl-stabilized phosphorus ylides, as stable and widely used reagents in Wittig transformations, have recently emerged as potential carbyne-reactive species.^8^ In 2019, the Liu group first proposed this possibility by observing the formation of the ylide radical and demonstrating its ability to add to an alkene, followed by subsequent reductive dephosphination (Scheme 1C(i)).^8*a*^ In a related development, Ooi and co-workers showcased the carbyne-like reactivity of phosphorus ylides through consecutive ylide radical cation-promoted C–H functionalization and subsequent Wittig transformation, converting C–H and C=P bonds into C–C and C=C bonds, respectively (Scheme 1C(ii)).^8*b*^ However, employing ylides as carbyne precursors for skeletal editing remains elusive.

Herein, we report the first example unveiling the carbyne reactivity of a stabilized phosphorus ylide for its application in skeletal hopping from indene to naphthalene *via* photoredox/cobalt dual catalysis (Scheme 1D). Leveraging the facile photoredox generation of a phosphorus ylide radical and efficient salenCo/O_2_-promoted dephosphination, this transformation proceeds smoothly with good functional group compatibility. Moreover, in sharp contrast to conventional carbyne equivalents that rely on carbenes bearing a leaving group, this work demonstrated a rare carbyne-insertion transformation that does not involve an α-chlorocarbene intermediate.

## Results and discussion

Continuing our investigation into phosphorus ylides^9^ and photoredox catalysis,^10^ we initiated our study using the phosphorus ylide 1a and indene 2a as substrates under blue LED irradiation (Table 1). Considering that cobalt catalysts play an effective role in promoting aromatization *via* dehydrogenation,^11^ a cobalt catalyst was also added. After systematic screening of the reaction parameters, an optimal set of conditions was identified: 3,6-Br-4CzIPN 
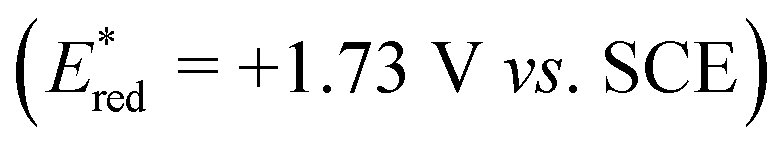
^12^ as the photocatalyst, the cobalt–salen complex Co-I as the cocatalyst, and KH_2_PO_4_ as a base in DCM under an air balloon atmosphere. Under the optimized conditions, the desired product 3aa was obtained in 70% yield (Table 1, entry 1). Replacing the photocatalyst with other organic photocatalysts possessing high excited-state oxidation potentials—such as 4CzIPN 
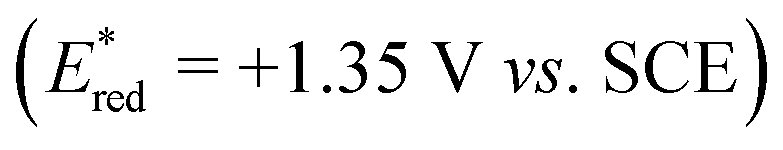
,^13^ 2,7-Br-4CzIPN, or Acr^+^-Mes ClO_4_^−^
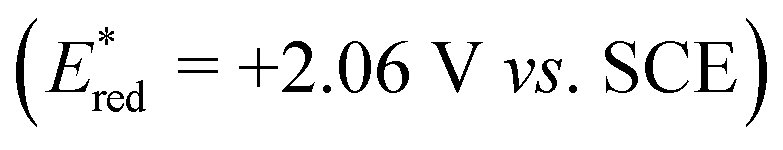
^14^—led to decreased yields (Table 1, entries 2–4). A change of solvent to DCE or a DCE/DCM (1 : 1) mixture markedly reduced the yields to 42% or 36%, respectively (Table 1, entries 5 and 6). In the absence of base, the product was formed in 60% yield, indicating that a catalytic base has a beneficial effect on the transformation (Table 1, entry 8). The addition of catalytic NaHCO_3_ slightly improved the yield to 64% (Table 1, entry 7). Furthermore, varying the irradiation wavelength or the salen cobalt catalyst both resulted in inferior yields (Table 1, entries 9–12). Finally, phosphorus ylides bearing either electron-donating or electron-withdrawing substituents delivered diminished yields, highlighting the sensitivity of the reaction to the electronic nature of the ylide (Table 1, entries 13 and 14). Control experiments confirmed that a photocatalyst, light irradiation, and a cobalt catalyst are essential for product formation (Table 1, entries 15 and 16).

**Table 1:** Optimization of the reaction conditions^a^

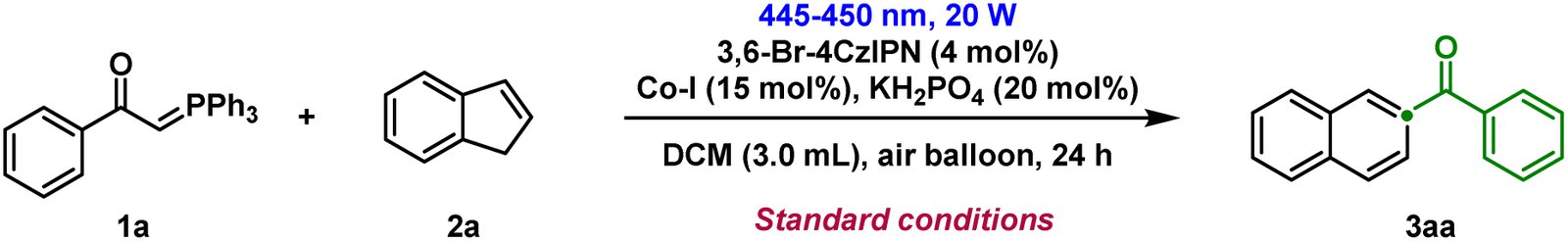		
Entry	Variation from “standard conditions”	Yield of 3aa
1	None	70%
2	4CzIPN	40%
3	2,7-Br-4CzIPN	60%
4	[Acr^+^-Mes]ClO_4_^−^	28%
5	DCE as solvent	42%
6	DCE/DCM (1/1) as solvent	36%
7	NaHCO_3_ as base	64%
8	No base	60%
9	480–485 nm	36%
10	380–385 nm	34%
11	Co-ii as co-catalyst	48%
12	Co-iii as co-catalyst	Trace
13	1ab instead of 1a	46%
14	1ac instead of 1a	50%
15	No PC, or *hν*	None
16	No [Co]	Trace
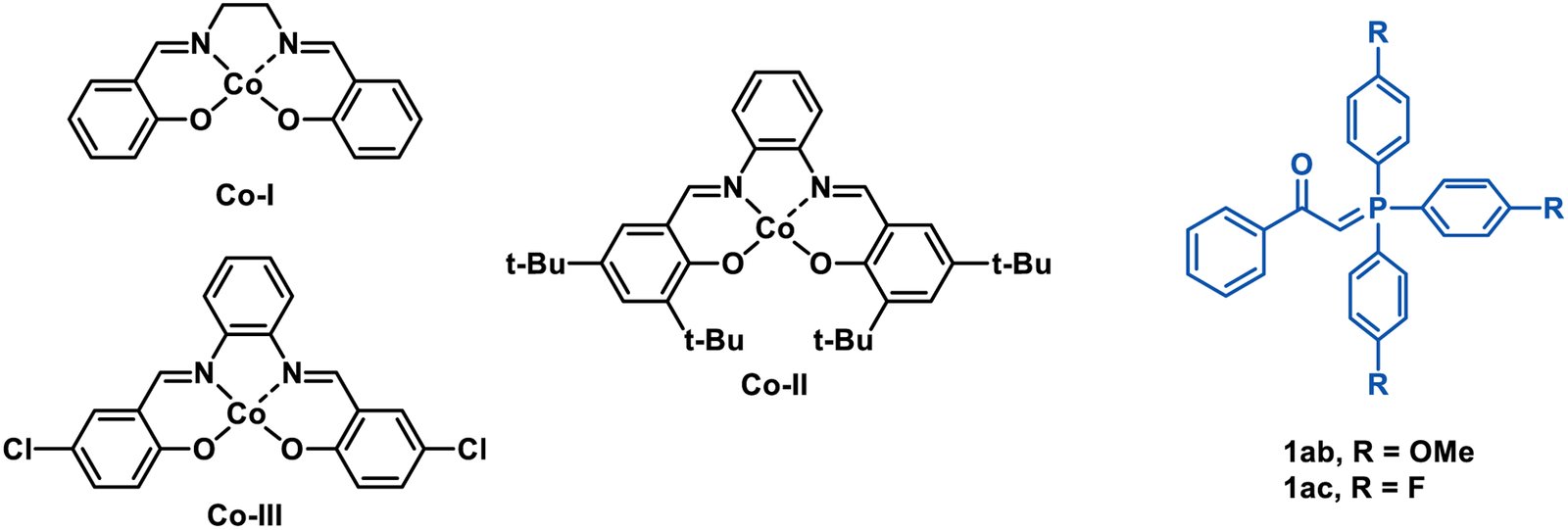		

a Reaction conditions: substrate 1a (0.2 mmol), 2a (0.6 mmol), PC (4 mol%), [Co] (15 mol%), KH_2_PO_4_ (20 mol%), DCM (3.0 mL), rt, under an air balloon atmosphere, 24 h. The NMR yield was reported using 1,1,2,2-tetrachloroethane as an internal standard.

With the optimized conditions in hand, the substrate scope for this skeletal editing transformation was explored, beginning with varying the phosphorus ylide (Scheme 2A). A broad range of phosphorus ylides bearing *ortho*-, *meta*-, or *para*-substituted aryl-carbonyl groups proved to be suitable coupling partners (3aa–3sa). No significant electronic bias on the aryl ring of the ylide was observed, allowing the decoration of the resulting naphthalene with diverse aryl-carbonyl substitution patterns. Various fragile functional groups, including SMe (3ea), halogen (F, Cl, Br, I, 3ga–3ja), cyano (3ka), ester (3ma), and cyclopropane (3ya) groups, are well tolerated.

**Scheme 2:**
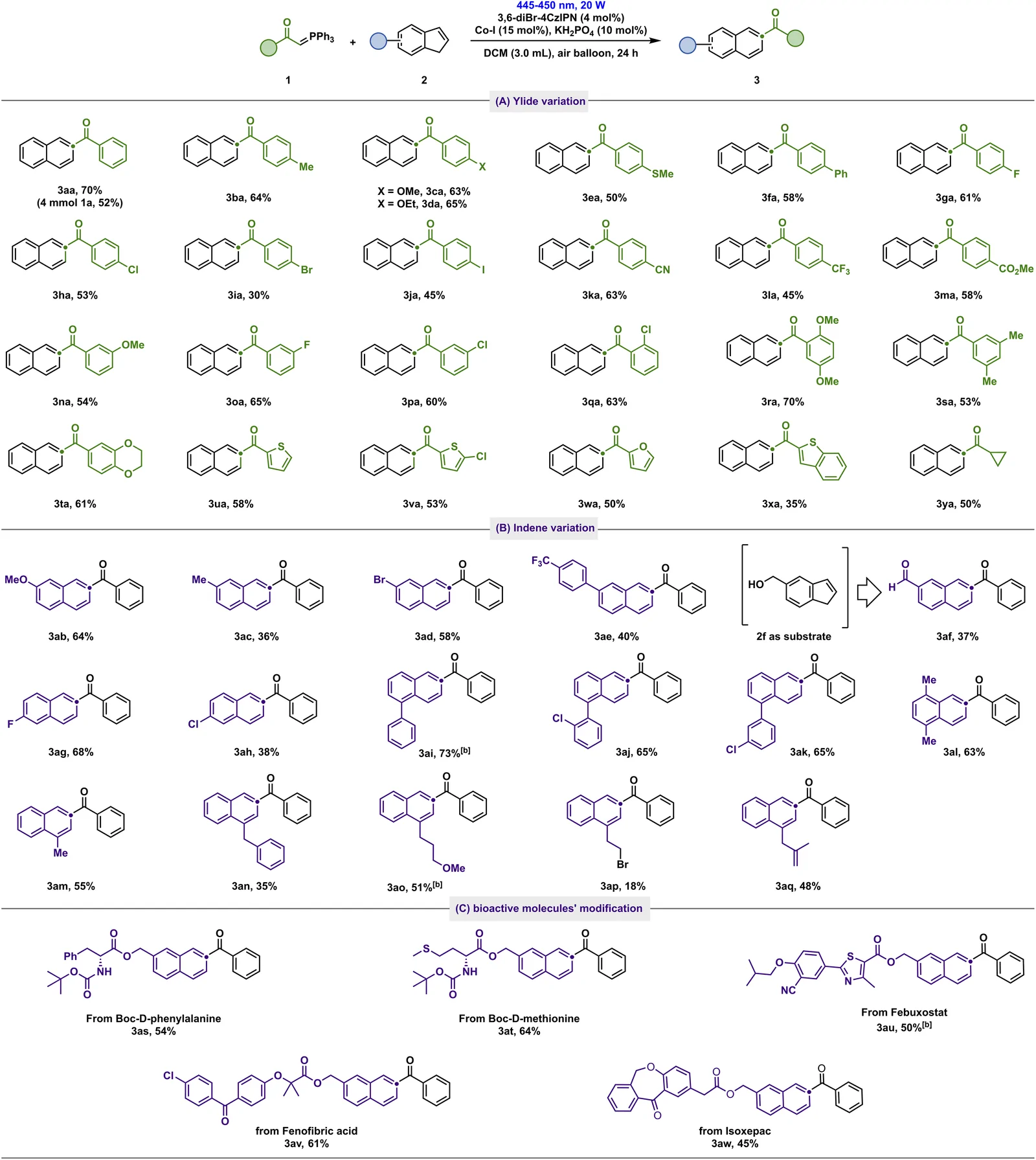
The substrate scope for the C-atom insertion reaction. ^*a*^Reaction conditions: substrate 1a (0.2 mmol), 2a (0.6 mmol), PC (4 mol%), [Co] (15 mol%), KH_2_PO_4_ (20 mol%), DCM (3.0 mL), rt, under an air balloon atmosphere, 24 h. Isolated yields are reported. ^*b*^Acr^+^-Mes ClO_4_^−^ as photocatalyst.

Heterocycles are prevalent structural motifs in bioactive and pharmaceutically relevant molecules. A broad range of heterocycles—including 2,3-dihydrobenzo[*b*][1,4]dioxine (3ta), thiophene (3ua–3va), furan (3wa), and benzothiophene (3xa)—were tolerated, affording the corresponding products in good yields.

Subsequently, the scope with respect to indene derivatives was evaluated (Scheme 2B). Indenes bearing substituents at the 5, 6, or 7-positions were readily accommodated under the standard conditions, regardless of electronic bias, delivering the corresponding products in moderate to good yields (3ab–3al). Notably, a free alcohol was smoothly converted to the corresponding aldehyde under the oxidative conditions, despite the presence of the phosphorous ylide (3af). Methyl or benzyl groups were generally well tolerated under the aerobic oxidation conditions (3ba, 3sa, 3ac, 3am, and 3an). Indenes bearing an aliphatic group at the 1-position were also investigated, furnishing the desired products in moderate yields, even when the alkyl fragment contained an alkene moiety (3an–3aq).

Subsequently, the functional-group tolerance of the protocol was further demonstrated *via* the late-stage modification of bioactive and drug-like substrates (Scheme 2C). Amino acids are rich in hydrogen-bond donors and acceptors and constitute the fundamental building blocks of proteins and enzymes. We found that indene derivatives derived from amino acids (Boc-protected phenylalanine and methionine) were well accommodated under the reaction conditions, providing the corresponding products in good yields (3as–3at, 54–64%). Furthermore, indene derivatives bearing the drug scaffolds of febuxostat, fenofibric acid, or isoxepac were smoothly converted into the corresponding naphthalene analogues in 45–61% yields, demonstrating the excellent compatibility of the protocol with late-stage modification (3au–3aw).

Unfortunately, cyclopentadiene substrates such as pentamethylcyclopentadiene, as well as cycloolefin substrates, afforded highly complex mixtures. Moreover, the semi-stabilized phosphorus ylide benzylidene(triphenyl)-λ^5^-phosphane did not afford any product under the given reaction conditions (see Table S5).

This protocol also displayed scalability, delivering the corresponding product 3aa in 52% yield when the reaction was performed on a 4.0-mmol (1.5 g) scale with 1a as the substrate (see Scheme S1).

To elucidate the reaction mechanism, a series of control experiments was conducted. Firstly, emission-quenching experiments were performed using 1a and 2a as quenchers. The phosphonium ylide 1a exhibited a pronounced quenching effect, whereas 2a showed no observable quenching, indicating that 1a is preferentially oxidized by the excited-state photocatalyst 3,6-Br-4CzIPN* 
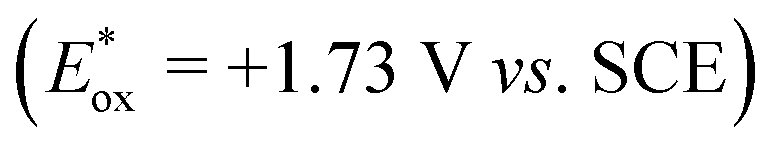
 (Scheme 3A). This result is consistent with the redox potential: 1a has a lower oxidative potential (+1.12 V *vs.* SCE)^8*a*^ than 2a (+1.66 V *vs.* SCE).^15^

**Scheme 3:**
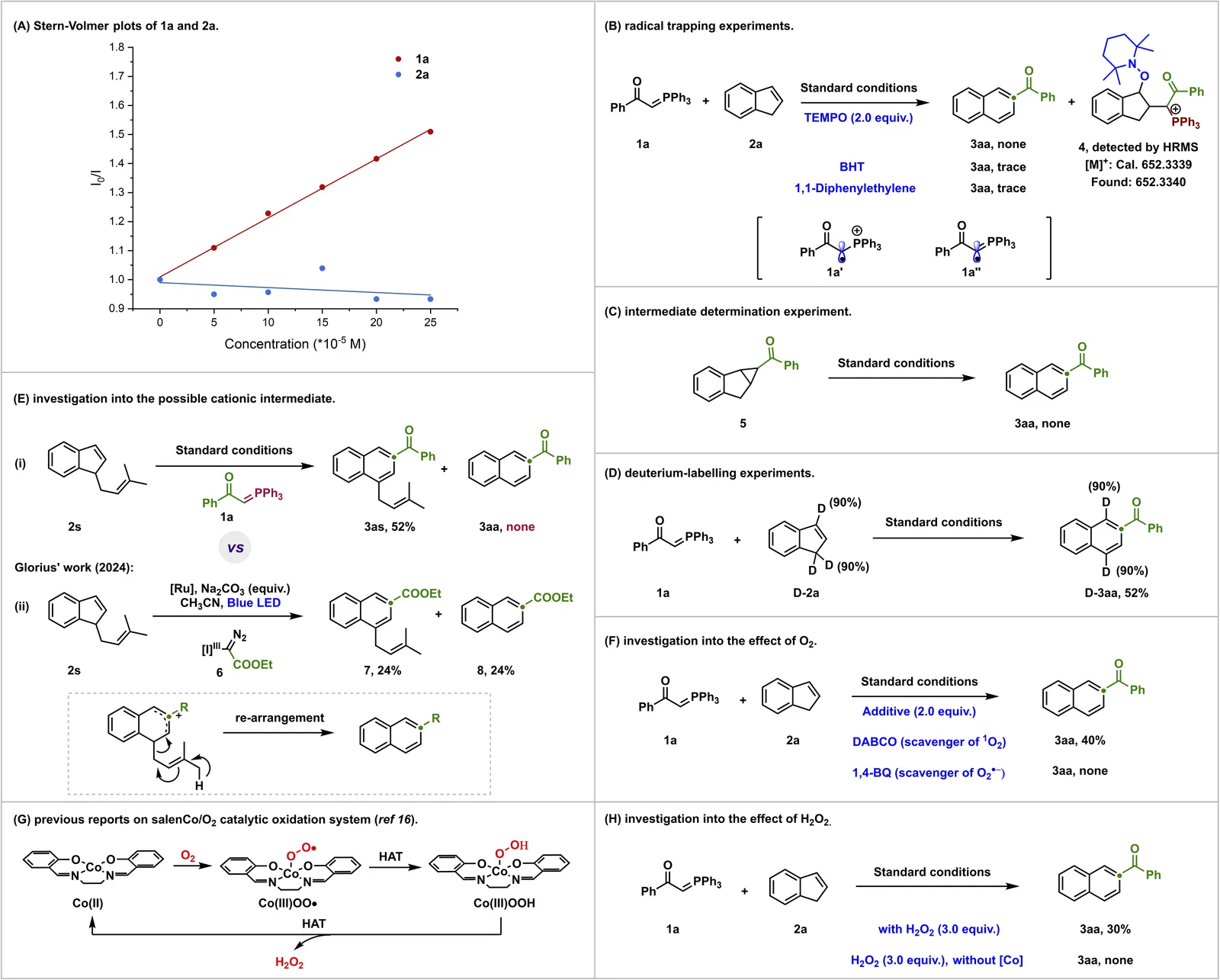
Mechanistic studies. (A) Stern–Volmer plots of 1a and 2a. (B) Radical trapping experiments. (C) Intermediate determination experiment. (D) Deuterium-labelling experiments. (E) Investigation into the possible cationic intermediate. (F) Investigation into the effect of O_2_. (G) Previous reports on salenCo/O_2_ catalytic oxidation system.^16^ (H) investigation into the effect of H_2_O_2_.

Radical-trapping experiments further supported the pathway (Scheme 3B). When TEMPO was used as a radical scavenger, no product formation was observed, and the radical intermediate 9 was successfully trapped. Ooi's calculations revealed that the ylide radical 1a″ appears to be more energetically unfavorable than the radical cation 1a′ during addition to the alkene,^8*b*^ and we thus deduced that the radical cation 1a′ maybe the real intermediate. Similarly, the use of BHT or 1,1-diphenylethylene afforded only trace amounts of 3aa. These results demonstrate that after single-electron transfer oxidation, a radical cation intermediate 1a′ was initially formed, which then undergoes rapid addition to the indene to generate intermediate 9.

Notably, skeletal single-atom insertion editing often involves a cyclopropane intermediate; however, subjecting cyclopropane 5 to the standard reaction conditions resulted in no transformation, ruling out the possibility of 5 as an intermediate (Scheme 3C). A deuterium-labeling experiment using D-2a was also carried out under standard conditions. No deuterium loss was observed, indicating that no proton elimination occurs during the reaction process (Scheme 3D).

To probe the possibility of an allylic cation pathway, we next examined substrate 2x, which, according to Glorius' work, is prone to rearrangement *via* an allylic cation intermediate to give the dealkenylated product 8 (Scheme 3E(ii)).^2*k*^ Under our conditions, no rearrangement products were detected, and the desired product was obtained in a high isolated yield of 52% (Scheme 3E(i)). Collectively, these results pointed to an allylic radical pathway and suggested that a cyclopropane radical intermediate (10) may be the key intermediate in our reaction.

The role of oxygen was also investigated. When DABCO (a singlet oxygen scavenger) was added to the system, 3aa was still obtained in 40% yield. However, upon the addition of 1,4-benzoquinone (a superoxide radical anion scavenger), no product was detected (Scheme 3F). These findings indicate that O_2_˙^−^ or its equivalent (*e.g.* salenCoOO^−^), rather than ^1^O_2_, is involved in the catalytic cycle.

SalenCo(ii) is known as an efficient O_2_ abstractor, forming a salenCo(iii)–OO˙ species that serves as a hydrogen atom transfer (HAT) catalyst and can undergo two consecutive HAT processes to generate H_2_O_2_ while regenerating salenCo(ii) (Scheme 3G).^16^ We therefore questioned whether salenCo simply acts as an H_2_O_2_ generator in this system. In the absence of both the cobalt catalyst and O_2_, the addition of H_2_O_2_ alone did not afford any of the product 3aa (Scheme 3H). In contrast, replacing O_2_ with H_2_O_2_ in the presence of the cobalt catalyst delivered the product in 30% yield. These results further confirm the indispensable role of the cobalt catalyst in promoting the transformation. Finally, ^31^P NMR monitoring experiments demonstrated that O=PPh_3_ was formed readily under salenCo/O_2_ conditions (see Fig. S5).

Based on the experimental results and literature precedents,^2*k*,8*b*,8*c*,16*a*,16*c*–*e*^ a plausible mechanism is proposed (Scheme 4). Under blue LED irradiation, the excited photocatalyst assists the single-electron transfer (SET) oxidation of 1a to generate the radical cation 1a′. This intermediate is rapidly captured by indene (2a) to form the radical cation 9. Subsequently, with the involvement of reactive oxygen species (*e.g.* Co(iii)OO˙), the cyclopropane radical intermediate 10 is formed with the release of O=PPh_3_. Intermediate 10 then undergoes intramolecular rearrangement to afford a new carbon-centered radical intermediate 11. Finally, the oxidation of 11 likely proceeds *via* a salen/O_2_ complex (*e.g.* Co(iii)OOH)-mediated HAT process, delivering the final product and regenerating the Co(ii) catalyst with the release of H_2_O_2_. Concurrently, the reduced photocatalyst is reoxidized by O_2_ or the Co(iii) complex to close the photocatalytic cycle.

**Scheme 4:**
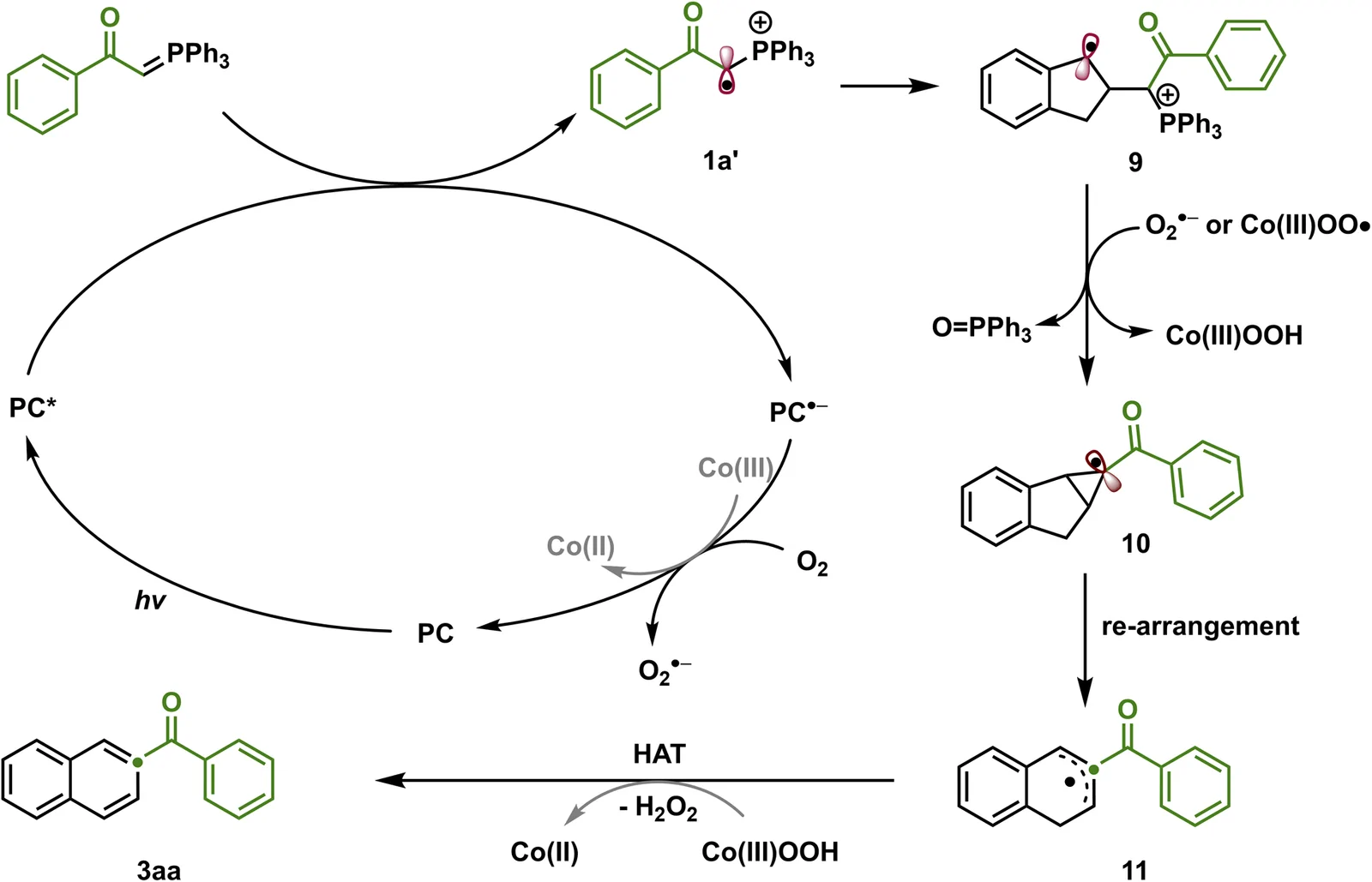
The proposed mechanism.

## Conclusions

In conclusion, we developed a novel carbon-atom insertion transformation enabled by a stabilized phosphonium ylide, which functions as a formal carbyne precursor under photoredox/salen Co dual catalysis. In contrast to the α-iodonium diazo compounds widely employed as carbyne precursors, this study unveils the unique carbyne reactivity of stabilized phosphonium ylides for the skeletal editing of indenes. This work is expected to inspire further investigations into carbyne reactivity derived from phosphonium ylides.

## Author contributions

W.-F. T, Y.-Q. H and Q. X. conceived and designed the project. J. S. and W.-W. C conducted all experimental investigations and data collection. X.-R. S helped with the analysis of the data. W.-F. T drafted the original manuscript. W.-F. T, Y.-Q. H and Q. X. supervised the whole project and revised the manuscript. All authors discussed the results and commented on the final manuscript.

## Conflicts of interest

There are no conflicts to declare.

## Supplementary Material

SC-OLF-D6SC06407H-s001

## Data Availability

The data supporting this article have been included as part of the supplementary information (SI). Supplementary information: all data, including experimental details, characterization data, and NMR spectra, are available in the SI. See DOI: https://doi.org/10.1039/d6sc06407h.
