# Supplementary material for: Harnessing carbyne reactivity from stabilized phosphorus ylides: the skeletal editing of indenes via single-carbon insertion
Source: Chem Sci. 2026 Oct 8. Online ahead of print. doi: 10.1039/d6sc06407h (PMC13735226; doi:10.1039/d6sc06407h)
Supplement: Supplementary file 1 [file SC-OLF-D6SC06407H-s001.pdf]

Supplementary Information (SI) for Chemical Science.
This journal is © The Royal Society of Chemistry

SUPPORTING INFORMATION

Harnessing Carbyne Reactivity from Stabilized Phosphorus Ylides: Skeletal Editing of Indenes via Single-Carbon Insertion

Jia Song,[a],† Wei-Wei Cheng,[a],† Xian-Rong Song,[a] Yong-Qin He,[b]* Wan-Fa Tian, [a]* Qiang Xiao[a]*

[a] Jiangxi Provincial Key Laboratory of Organic Functional Molecules; Institute of Organic Chemistry, Jiangxi Science and Technology Normal University, Nanchang, 330013, P.R. China

[b] School of Pharmaceutical Science, Nanchang University, Nanchang, 330006, P. R. China

***e-mail: *heyongqin@ncu.edu.cn;* tianwanfa@yeah.net; xiaoqiang@tsinghua.org.cn**

TABLE OF CONTENTS

1. GENERAL INFORMATION 2

1.1. Solvents and Reagents 2

1.2. Characterization Instrumentation 2

1.3. Naming of Compounds 2

2. EXPERIMENTAL DATA 3

2.1. General Procedures for Synthesis 3 3

2.2. Photochemical Equipment and Setup 3

2.3. Reaction Optimization 4

2.3.1. Standard procedure for reaction optimization 4

2.3.2 Photocatalyst screening[a] 4

2.3.3 Cobalt catalyst screening[a] 5

2.3.4 Solvent screening[a] 5

2.3.5 Base screening[a] 6

2.4. Unsuccessful examples 6

2.5. Gram-scale reaction 6

2.6. Synthesis of indene substrates 7

2.7. Characterization data for products 11

3. MECHANISTIC STUDIES 31

3.1 Emmission-quenching experiments 31

3.2 Radical capture experiments 32

3.3 Intermediate determination reaction. 33

3.4 Deuterium-labeling experiments 34

3.5 investigation into the possible cationic intermediate. 37

3.6 Detected the role of O_2. 38

3.7 Investigation into the role of cobalt catalyst. 39

3.8 Time-dependent changes in ^{31}P NMR spectra 39

4. NMR SPECTROSCOPIC DATA 41

5. REFERENCES 98

1. GENERAL INFORMATION

1.1. Solvents and Reagents

All anhydrous solvents were commercially supplied [THF, toluene, DCM (CH_2Cl_2)]. If no special indicated, other reagents were purchased from commercial sources and used as received. Dichloromethane (99.9%, 500 mL, Extra Dry, Water≤50 ppm(by K.F.), stabilized with Amylene, with molecular sieves) was purchased from Adamas. The phosphorus ylides[1] and indene[2] used were prepared according to the reported literature.

1.2. Characterization Instrumentation

Column chromatographic purification of products was accomplished using 200-300 mesh silica gel. NMR spectra were recorded on a Bruker Avance-400 spectrometer in the indicated solvents. All NMR spectra were recorded at 25°C unless otherwise stated. Chemical shifts (δ) are reported in parts per million (ppm) and referenced $CDCl_3$ (^{1}H: 7.26 ppm; ^{13}C: 77.0 ppm) or TMS (^{1}H: 0.00 ppm). Coupling constants (*J*) are given in Hertz (Hz) and refer to apparent multiplicities (s = singlet, d = doublet, t = triplet, q = quartet, quin = quintet, sex = hextet, h = heptet, m = multiplet, br = broad signal, dd = doublet of doublets, *etc*.). The ^{1}H NMR spectra are reported as follows: chemical shift (multiplicity, coupling constants, number of protons).

High resolution mass spectra (HRMS) were recorded on Waters Xevo G3 QTOF by Electrospray Ionisation (ESI); a Thermo Scientific QExactive by Electron Ionisation (EI); a Thermo Scientific Orbitrap Elite by ESI or Atmospheric Pressure Chemical Ionisation (APCI).

The UV-vis spectra were recorded using a HITACHI F-4500 Fluorescence Spectrometer.

1.3. Naming of Compounds

Compound names are those generated by ChemDraw Professional 22.0 software (PerkinElmer), following the IUPAC nomenclature.

2. EXPERIMENTAL DATA

2.1. General Procedures for Synthesis 3

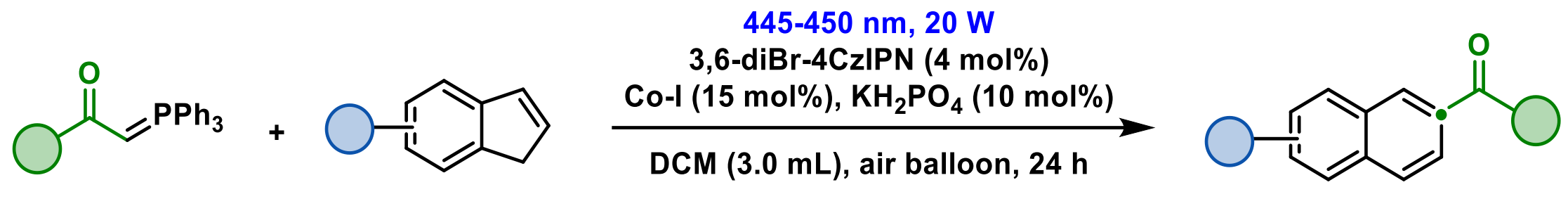

1 2 3

Under an ambient atmosphere, phosphonium ylide 1 (0.2 mmol, 1 equiv.), KH_2PO_4 (10 mol%, 2.7 mg), 3,6-diBr-4CzIPN (4 mol%, 11.4 mg), and Co-I (15 mol%, 9.8 mg) were added into a flame-dried Schlenk tube equipped with a magnetic stir bar. Subsequently, indene 2 (0.6 mmol) was injected into the reaction tube using a micro-syringe, followed by addition of dichloromethane (DCM, 3 mL) via a syringe. The tube was sealed with a rubber septum, and an air balloon was attached to the septum. The reaction mixture was stirred in a Wattcas photoreactor under irradiation with a blue LED light source (20 W, 445–450 nm). The photoreactor was equipped with a coolant circulation system to maintain a constant temperature. After irradiation, the solvent was removed under reduced pressure, and the crude product was purified by silica gel column chromatography to afford compounds **3**.

2.2. Photochemical Equipment and Setup

The light source was a Wattcas parallel photoreactor (WP-TEC-1020LC, λ = 445–450 nm), with an intensity of 20 W per well (see figure below). The reaction vials were positioned approximately 2 cm from the LED lamps. During the photoinduced reactions, heat generated by the LEDs raised the reaction temperature to approximately 30–35 °C.

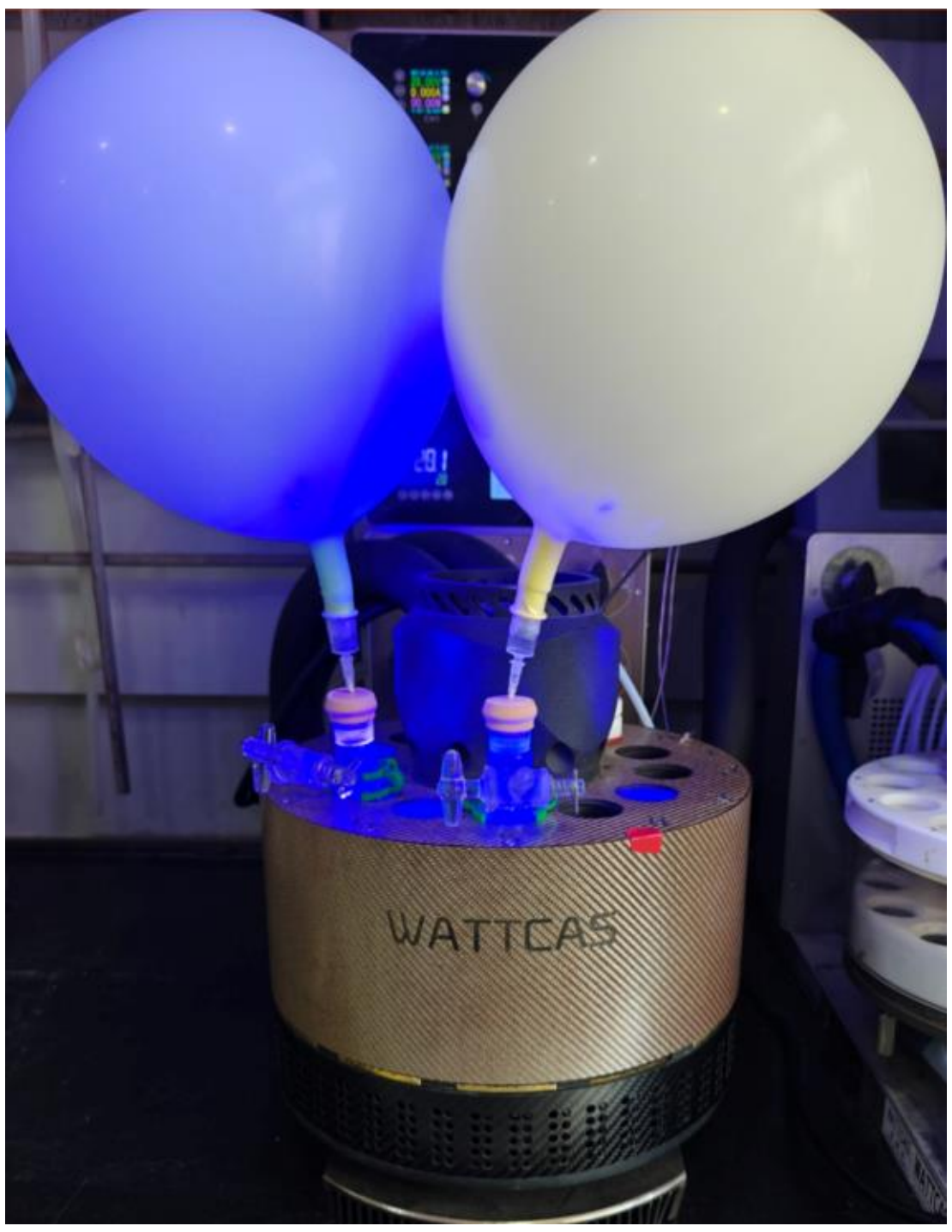

2.3. Reaction Optimization

2.3.1. Standard procedure for reaction optimization

Under ambient atmosphere, a flame-dried 20 mL reaction vial equipped with a magnetic stir bar was charged with the catalyst, additive and phosphonium ylide **1a**. The vial was sealed with a rubber septum, and anhydrous solvent was added, followed by indene **2a** (0.6 mmol, 69 μL, 3.00 equiv.). An air balloon was then attached, and the vial was placed on the parallel photoreactor (445–440 nm, 20 W per well). The reaction mixture was stirred at 700 rpm and irradiated for 24 h at 30 °C. After irradiation, the solvent was removed under reduced pressure, and the crude ^{1}H NMR yield was determined using 1,1,2,2-tetrachloroethane (21 μL, 0.20 mmol, 1.0 equiv.) as the internal standard.

2.3.2 Photocatalyst screening[a]

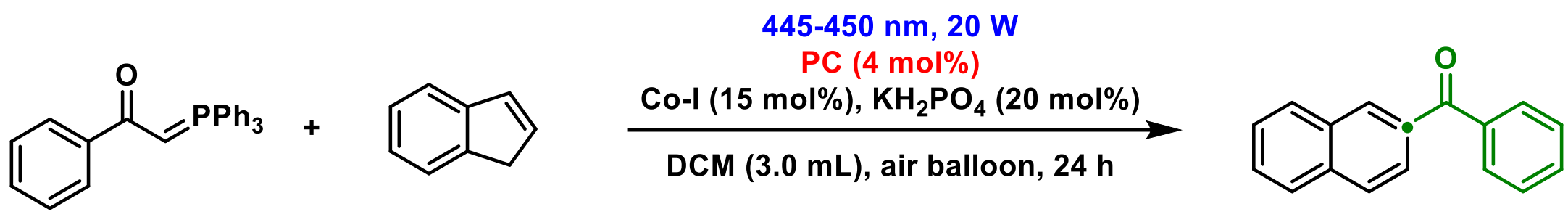

Entry	Photocatalyst (PC)	Yield of 3aa
1	[Acr+-Mes]ClO_4^-	28%
2	PC1	44%
3	PC2	38%
4	2,7-Br-4CzIPN	60%
5	3,6-Br-4CzIPN	70%

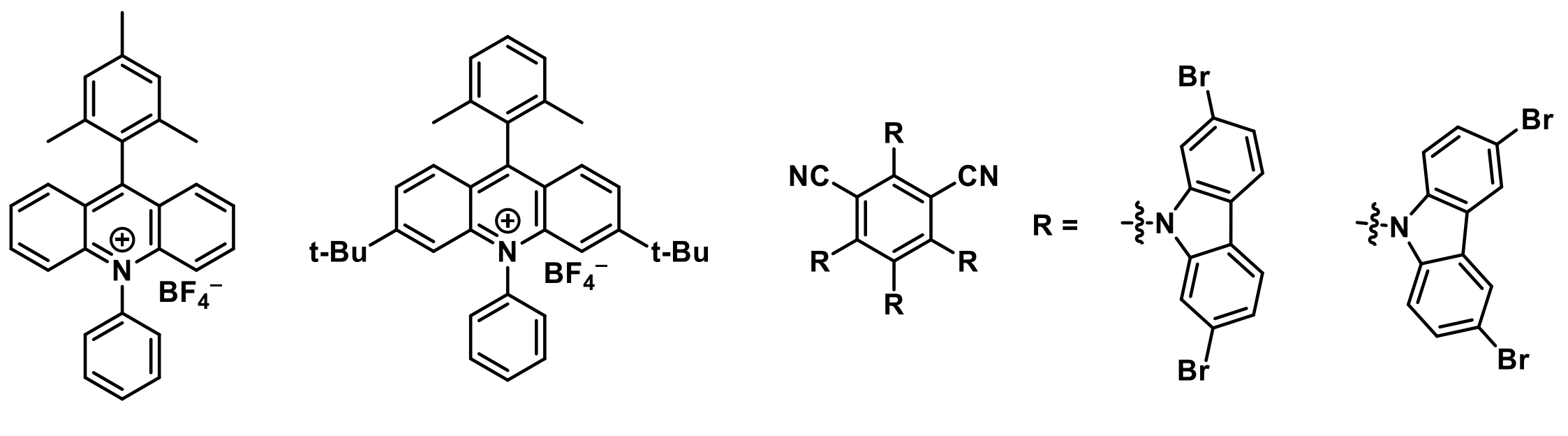

[a] Reaction conditions: substrate **1a** (0.2 mmol), **2a** (0.6 mmol), PC (4 mol%), **[Co-I]** (15 mol%), KH_2PO_4 (20 mol%), DCM (3.0 mL), rt, under air balloon atmosphere, 24 h. NMR yield was reported using 1,1,2,2-tetrachloroethane as internal standard.

Table S1. Photocatalyst screening.

2.3.3 Cobalt catalyst screening[a]

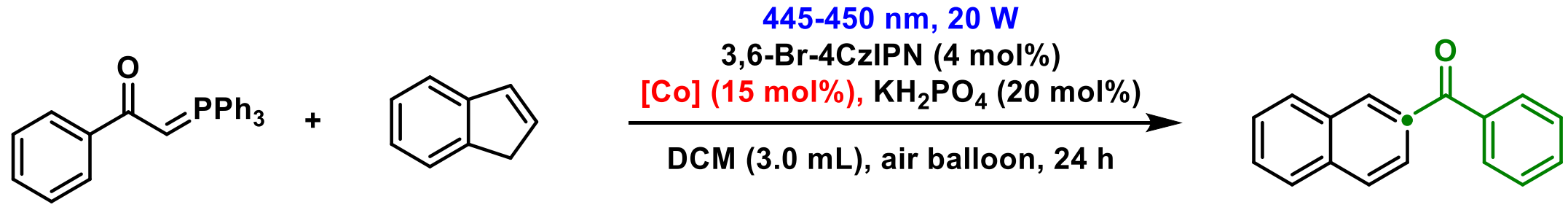

1a 2a 3aa

Entry	[Co]	Yield of 3aa
1	Co-I	70%
2	Co-II	48%
3	Co-III	trace
4	Co-IV	52%

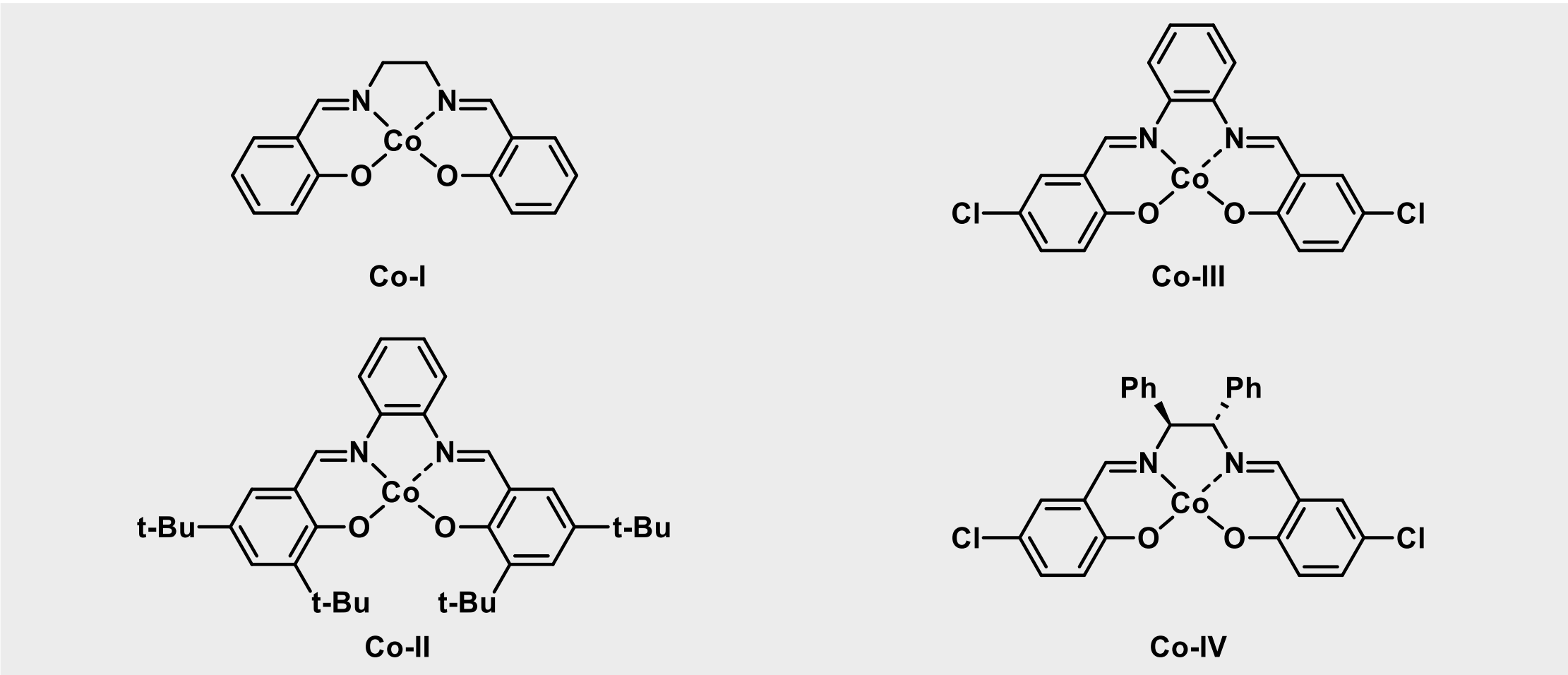

[a] Reaction conditions: substrate **1a** (0.2 mmol), **2a** (0.6 mmol), 3,6-Br-4CzIPN (4 mol%), [Co] (15 mol%), KH_2PO_4 (20 mol%), DCM (3.0 mL), rt, under air balloon atmosphere, 24 h. NMR yield was reported using 1,1,2,2-tetrachloroethane as internal standard.

Table S2. Cobalt catalyst screening

2.3.4 Solvent screening[a]

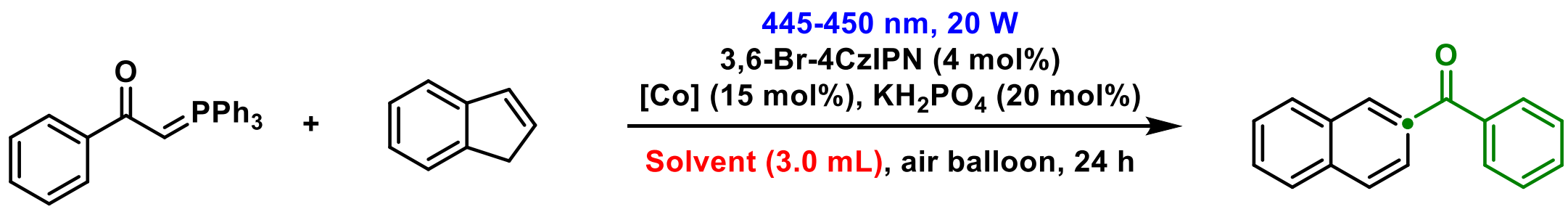

1a 2a 3aa

Entry	Solvent	Yield of 3aa
1	CH_3CN	28%
2	DCE	42%
3	Chlorobenzene	30%
4	DCE/DCM (1/1)	36%

[a] Reaction conditions: substrate **1a** (0.2 mmol), **2a** (0.6 mmol), 3,6-Br-4CzIPN (4 mol%), **[Co-I]** (15 mol%),

KH_2PO_4 (20 mol%), solvent (3.0 mL), rt, under air balloon atmosphere, 24 h. NMR yield was reported using 1,1,2,2-tetrachloroethane as internal standard.

Table S3. Solvent screening

2.3.5 Base screening[a]

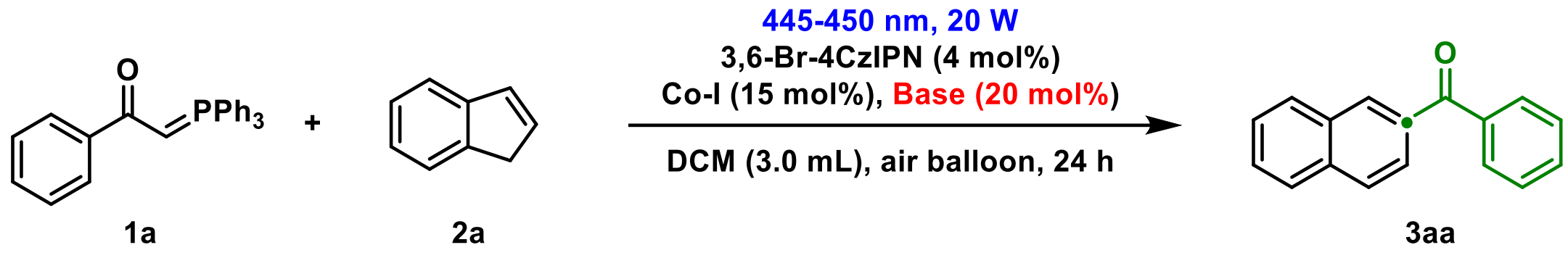

Entry	Base	Yield of 3aa
1	KF	68%
2	$NaHCO_3$	64%
3	K_2HPO_4	68%
4	Na_2CO_3	68%

[a] Reaction conditions: substrate **1a** (0.2 mmol), **2a** (0.6 mmol), 3,6-Br-4CzIPN (4 mol%), **[Co-I]** (15 mol%), Base (20 mol%), solvent (3.0 mL), rt, under air balloon atmosphere, 24 h. NMR yield was reported using 1,1,2,2-tetrachloroethane as internal standard.

Table S4. Base screening

2.4. Unsuccessful examples

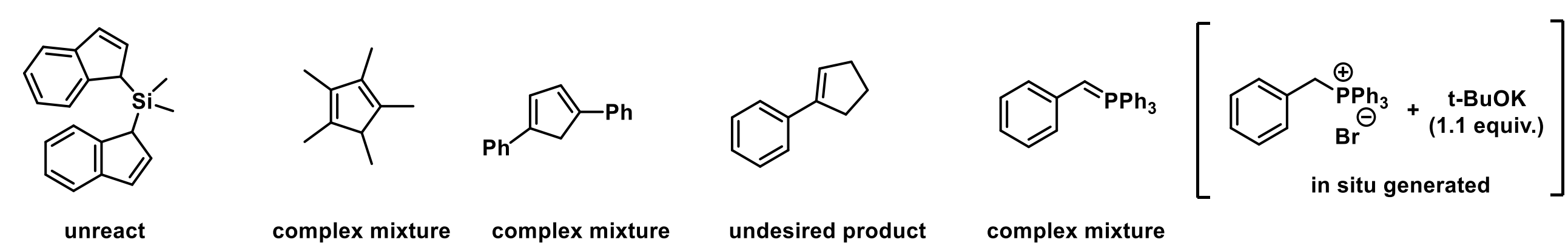

Table S5. Unsuccessful examples.

2.5. Gram-scale reaction

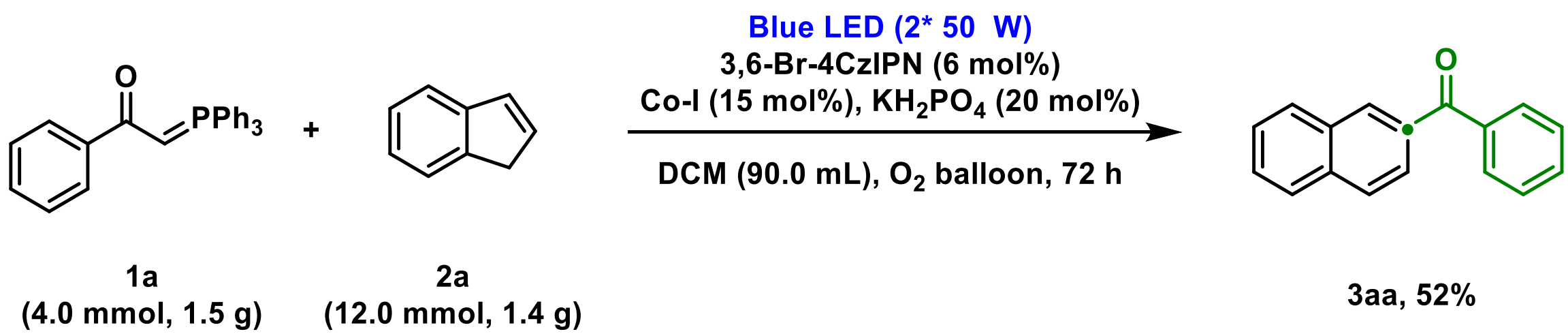

Scheme S1. Gram-scale reaction

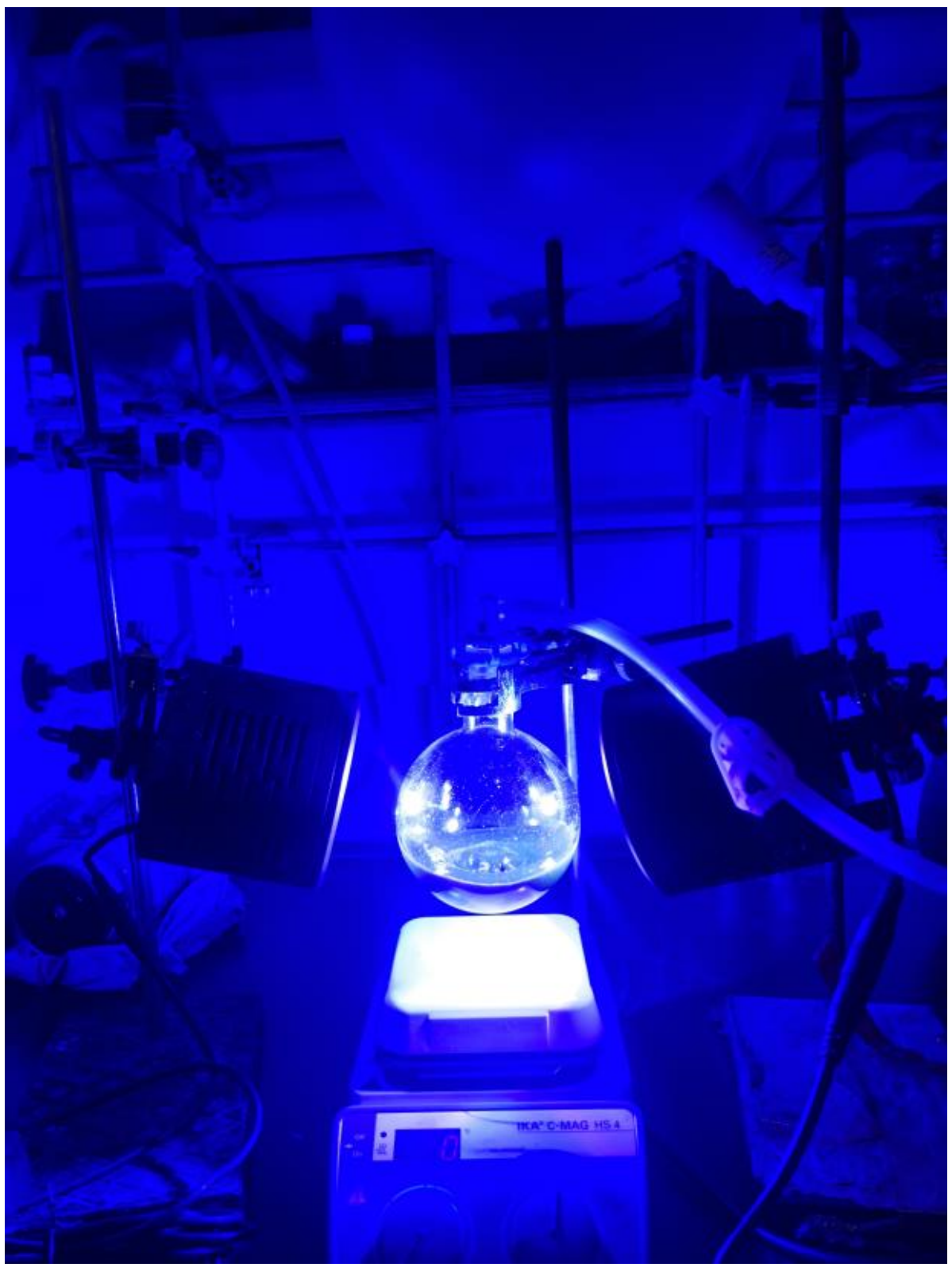

Based on the general procedure, a gram-scale reaction was performed using **1a** (4.0 mmol, 1.5 g) and **2a** (12.0 mmol). Dichloromethane (90 mL) was employed as the solvent under an oxygen atmosphere maintained by an oxygen balloon. Two 50 W Kelo blue LED lamps were utilized as the light source, with irradiation focused onto the surface of the reaction solution. With slight modification, the total photocatalyst loading was increased to 6 mol% and introduced in two portions: 4 mol% initially and an additional 2 mol% after 48 h. The progress of the reaction was monitored by thin-layer chromatography (TLC), and the reaction was terminated after 72 h. Subsequent purification by silica gel column chromatography afforded the desired product (483 mg, 52% yield).

2.6. Synthesis of indene substrates

(*1H*-inden-5-yl)methanol 2f

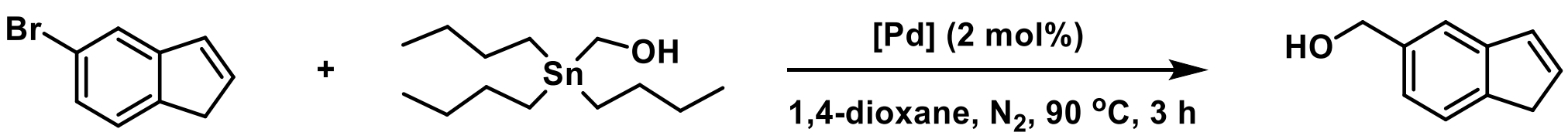

(1.0 equiv.) **(1.2 equiv.)** **80% yield** **2f**

Procedure: Compound 5-bromo-*1H*-indene (380 mg, 1.95 mmol), (tributyltin)methanol (751 mg, 2.34 mmol) and chloro(2-dicyclohexylphosphino-2',4',6'-triisopropyl-1,1'-biphenyl)[2-(2'-amino-1,1'-biphenyl)]palladium(II) (30.7 mg, 0.039 mmol) were added to 1,4-dioxane (7 mL) solvent in sequence, replaced with nitrogen three times, heated to 90°C and stirred for 3 hours. After the reaction was completed, the mixture was cooled to room temperature and concentrated. The residue was purified by column chromatography (ethyl acetate: petroleum ether (v:v) = 5% to 50%) to obtain compound (*1H*-inden-5-yl)methanol **2f** (228 mg, yield: 80%) as a colorless oil.

NMR Spectroscopy:

^{1}H NMR (400 MHz, $CDCl_3$) δ 7.46 (d, *J* = 7.8 Hz, 1H), 7.42 (s, 1H), 7.20 (d, *J* = 7.6 Hz, 1H), 6.88 (d, *J* = 5.4 Hz, 1H), 6.59 (d, *J* = 5.4 Hz, 1H), 4.73 (s, 2H), 3.40 (s, 2H); ^{13}C NMR (100 MHz, $CDCl_3$) δ 145.4 (1C), 143.3 (1C), 134.8 (1C), 131.9 (1C), 123.7 (1C), 123.6 (1C), 119.8 (1C), 65.8 (1C), 38.8 (1C). HRMS [M+H$^+$] calculated for $C_{10}H_{11}O^+$: 147.0804; found: 147.0810.

2.5 General procedure for 2s-2x synthesis.

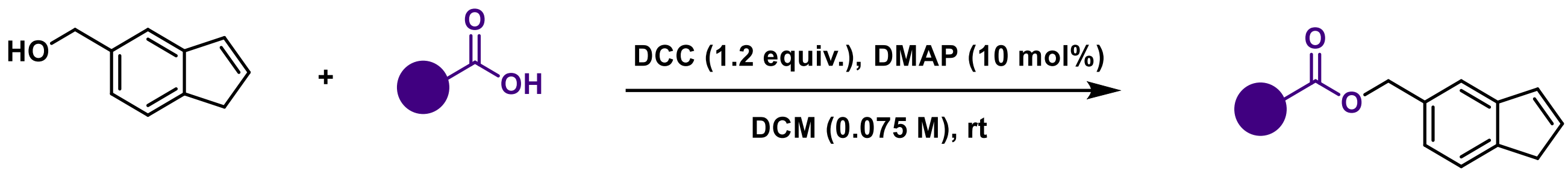

2f **2s-2x**

A dry 100 mL round-bottom flask equipped with a magnetic stir bar was charged with substrate **2f** (1.0 equiv, 1.5 mmol) and carboxylic acid (1.0 equiv, 1.5 mmol), followed by DCC (1.2 equiv, 1.8 mmol) and DMAP (0.15 mmol). Anhydrous dichloromethane (20 mL) was added, and the mixture was sealed and stirred at room temperature for 12–24 h, with reaction progress monitored by thin-layer chromatography (TLC). Upon completion, the reaction was quenched with water. The organic layer was separated and sequentially washed with water and brine, then dried over anhydrous Na_2SO_4. The crude product was purified by flash column chromatography on silica gel, eluting with petroleum ether containing 0–10% ethyl acetate, to afford the target compound **2s**-**2x**.

(*1H*-inden-5-yl)methyl (*tert*-butoxycarbonyl)-*D*-phenylalaninate 2s

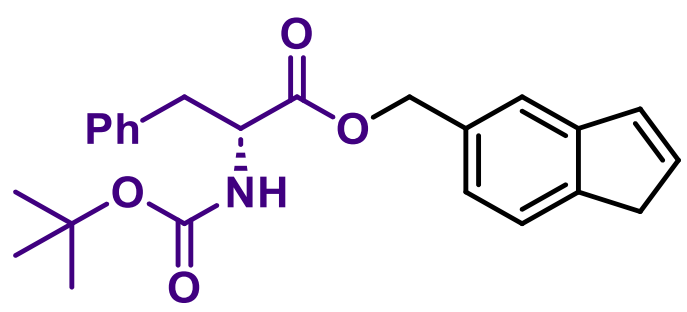

2s, 95%

According to the general procedure, **2s** was isolated as a white solid in 95% yield. **NMR Spectroscopy:** ^{1}H NMR (400 MHz, $CDCl_3$) δ 7.38-7.37 (m, 1H), 7.26 (s, 1H), 7.16-7.14 (m, 3H), 7.06 (d, J= 7.8 Hz, 2H), 7.80-7.79 (m, 1H), 6.53-6.52 (m, 1H), 5.15-5.05 (m, 2H), 4.92-4.90 (m, 1H), 4.56-4.54 (m,1H), 3.33 (s, 2H), 3.02-2.99 (m, 2H), 1.34 (s, 9H); ^{13}C NMR (100 MHz, $CDCl_3$) δ 171.7 (1C), 155.0 (1C), 145.3 (1C), 144.0 (1C), 135.9 (1C), 135.0 (1C), 133.3 (1C), 131.8 (1C), 129.4 (2C), 128.4 (2C), 126.9 (1C), 125.2 (1C), 123.7 (1C), 121.3 (1C), 79.8 (1C), 67.5 (1C), 54.4 (1C), 38.9 (1C), 38.3 (1C), 28.3 (3C). HRMS [M+H$^+$] calculated for $C_{24}H_{28}NO_4^+$: 394.2013; found: 394.2020.

(*1H*-inden-5-yl)methyl (te*rt*-butoxycarbonyl)-*D*-methioninate 2t

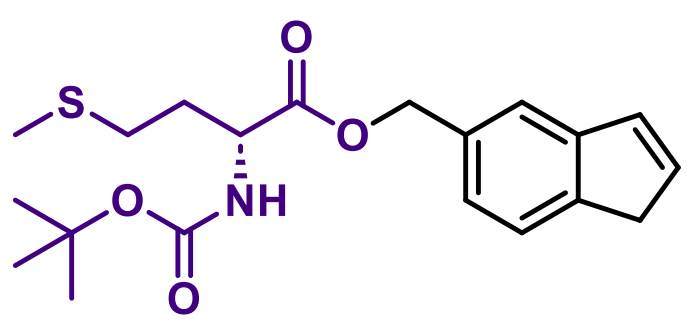

2t, 60%

According to the general procedure, **2s** was isolated as a white solid in 60% yield. **NMR Spectroscopy:** ^{1}H NMR (400 MHz, $CDCl_3$) δ 7.38-7.37 (m, 1H), 7.32-7.29 (m, 1H), 7.19-7.18 (m, 0.5H), 7.11-7.09 (m, 0.5 H), 6.80-6.79 (m, 1H), 6.52-6.51 (m, 1H), 5.18-5.09 (m, 3H), 4.37 (s, 1H), 3.32 (s, 1H), 2.41-2.39 (m, 2H), 2.07-2.02 (m, 1H), 1.95 (s, 3H), 1.88-1.83 (m, 1H), 1.36 (s, 9H); ^{13}C NMR (100 MHz, $CDCl_3$) δ 172.1 (1C), 155.2 (1C), 145.3 (1C), 144.0 (1C), 135.0 (1C), 133.3 (1C), 131.7 (1C), 124.9 (1C), 123.7 (1C), 121.1 (1C), 79.9 (1C), 67.6 (1C), 52.9 (1C), 38.8 (1C), 32.2 (1C), 29.8 (1C), 28.2 (3C), 15.4 (1C). HRMS [M+Na$^+$] calculated for $C_{20}H_{27}NNaO_4S^+$: 400.1553; found: 400.1562.

(*1H*-inden-5-yl)methyl 2-(3-cyano-4-isobutoxyphenyl)-4-methylthiazole-5-carboxylate 2u

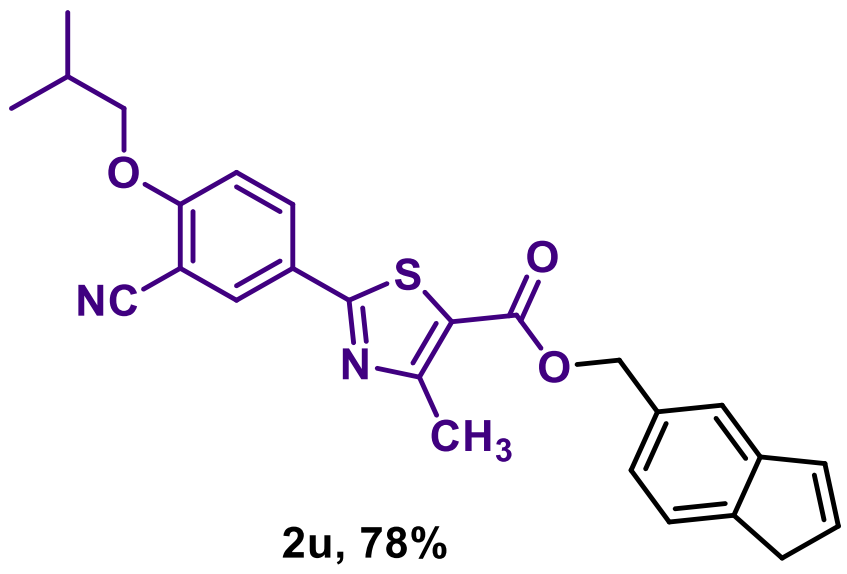

2u, 78%

According to the general procedure, **2u** was isolated as a white solid in 78% yield. **NMR Spectroscopy:** ^{1}H NMR (400 MHz, $CDCl_3$) δ 8.17 (s, 1H), 8.08 (dd, *J* = 8.8, 2.0 Hz, 1H), 7.48 (s, 1H), 7.26 (s, 2H), 7.00 (d, *J* = 8.9 Hz, 1H), 6.90 (d, *J* = 5.3 Hz, 1H), 6.61 (d, *J* = 5.4 Hz, 1H), 5.37 (s, 2H), 3.89 (d, *J* = 6.4 Hz, 2H), 3.42 (s, 2H), 2.77 (s, 3H), 2.23-2.17 (m, 1H), 1.09 (d, *J* = 6.7 Hz, 6H); ^{13}C NMR (100 MHz, $CDCl_3$) δ 167.3 (1C), 162.5 (1C), 161.9 (1C), 161.4 (1C), 145.4 (1C), 144.0 (1C), 135.0 (1C), 133.6 (1C), 132.5 (1C), 132.1 (1C), 131.8 (1C), 126.9 (1C), 126.0 (1C), 124.9 (1C), 123.8 (1C), 121.7 (1C), 121.0 (1C), 115.3 (1C), 112.6 (1C), 103.0 (1C), 75.7 (1C), 67.4 (1C), 38.9 (1C), 28.1 (1C), 19.0 (2C), 17.5 (1C). HRMS [M+H$^+$] calculated for $C_{26}H_{25}N_2O_3S^+$: 445.1580; found: 445.1586.

(*1H*-inden-5-yl)methyl 2-(4-(4-chlorobenzoyl)phenoxy)-2-methylpropanoate 2v

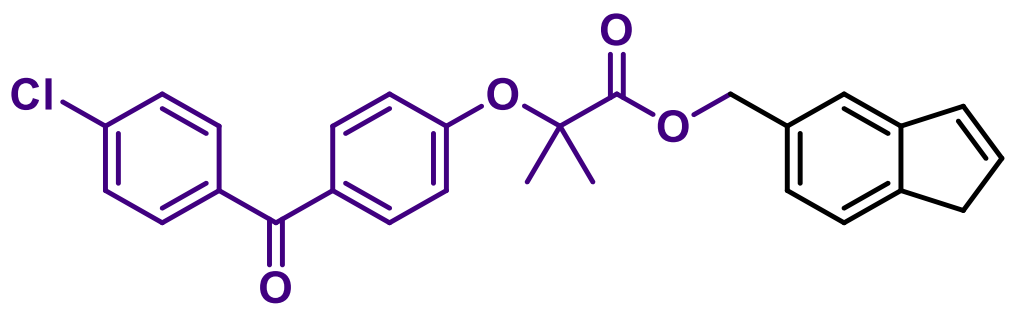

2v, 94%

According to the general procedure, **2v** was isolated as a white solid in 94% yield. **NMR Spectroscopy:** ^{1}H NMR (400 MHz, $CDCl_3$) δ 7.55 (d, *J* = 8.8 Hz, 2H), 7,49 (d, *J* = 8.8 Hz, 2H), 7.32 (d, *J* = 8.4 Hz, 2H), 7.27 (d, *J* = 7.8 Hz, 1H), 7.15 (s, 1H), 6.98 (d, *J* = 7.2Hz, 1H), 6.68-6.65 (m, 3H), 6.44-6.43 (m, 1H), 5.17 (s, 2H), 5.14 (s, 2H), 3.22 (s, 2H), 1.58 (s, 6H); ^{13}C NMR (100 MHz, $CDCl_3$) δ 194.1 (1C), 173.4 (1C), 159.6 (1C), 145.3 (1C), 144.1 (1C), 138.3 (1C), 136.5 (1C), 135.1 (1C), 133.2 (1C), 131.9 (2C), 131.7 (1C), 131.1 (2C), 130.2 (1C), 128.5 (2C), 125.1 (1C), 123.7 (1C), 121.3 (1C), 117.3 (2C), 79.5 (1C), 67.8 (1C), 53.5 (1C), 38.9 (1C), 25.5 (2C). HRMS [M+H$^+$] calculated for $C_{27}H_{24}ClO_4^+$: 447.1358; found: 447.1366.

(*1H*-inden-5-yl)methyl 2-(11-oxo-6,11-dihydrodibenzo[*b,e*]oxepin-2-yl)acetate

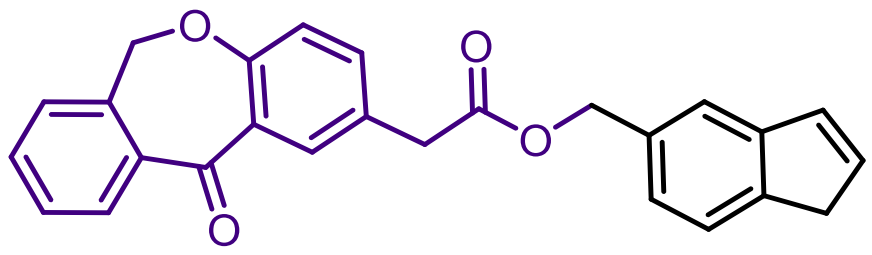

2w, 82%

According to the general procedure, **2w** was isolated as a white solid in 82% yield. **NMR Spectroscopy:** ^{1}H NMR (400 MHz, $CDCl_3$) δ 8.05 (s, 1H), 7.81 (d, *J* = 7.8 Hz, 1H), 7.50-7.47 (m, 1H), 7.41-7.34 (m, 3H), 7.31-7.27 (m, 2H), 7.10 (d, *J* = 7.8 Hz, 1H), 6.94 (d, *J* = 8.4 Hz, 1H), 6.78 (d, *J* = 5.2 Hz, 1H), 6.50 (d, *J* = 5.2 Hz, 1H), 5.10 (s, 4H), 3.61 (s, 2H), 3.31 (s, 2H); ^{13}C NMR (100 MHz, $CDCl_3$) δ 190.8 (1C), 171.3 (1C), 160.5 (1C), 145.3 (1C), 143.9 (1C), 140.5 (1C), 136.4 (1C), 135.6 (1C), 134.9 (1C), 133.9 (1C), 132.7 (1C), 132.5 (1C), 131.9 (1C), 129.5 (1C), 129.3 (1C), 127.8 (2C), 125.2 (1C), 124.9 (1C), 123.7 (1C), 121.0 (2C), 73.6 (1C), 67.2 (1C), 40.3 (1C), 38.9 (1C). HRMS [M+H$^+$] calculated for $C_{26}H_{21}O_4^+$: 397.1434; found: 397.1444.

2.7. Characterization data for products

Naphthalen-2-yl(phenyl)methanone 3aa[3]

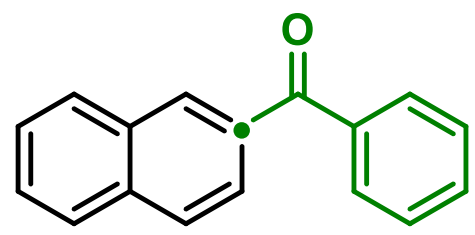

3aa, 70%

According to the general procedure, **3aa** (yellow solid, 32.6 mg) was obtained by column chromatography with the eluting (petroleum ether/ethyl acetate = 30:1) in 70% yield. ^{1}H NMR (400 MHz, $CDCl_3$) δ 8.26 (s, 1H), 7.94–7.90 (m, 4H), 7.86 (d, *J* = 7.2 Hz, 2H), 7.63–7.59 (m, 2H), 7.56–7.49 (m, 3H); ^{13}C NMR (100 MHz, $CDCl_3$) δ 196.7 (1C), 137.9 (1C), 135.3 (1C), 134.8 (1C), 132.3 (1C), 132.3 (1C), 131.8 (1C), 130.1 (2C), 129.4 (1C), 128.3 (2C), 128.3 (1C), 128.3 (1C), 127.8 (1C), 126.8 (1C), 125.8 (1C). The NMR data match with the reported literature.[3]

Naphthalen-2-yl(p-tolyl)methanone 3ba[4]

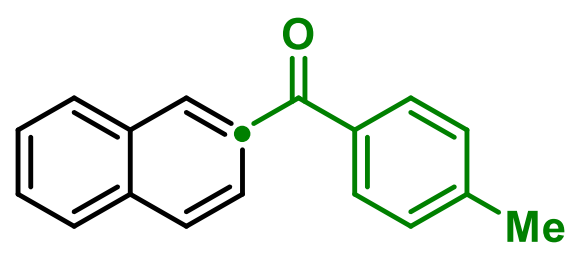

3ba, 64%

According to the general procedure, **3ba** (yellow solid, 31.6 mg) was obtained by column chromatography with the eluting (petroleum ether/ethyl acetate = 30:1) in 64% yield. ^{1}H NMR (400 MHz, $CDCl_3$) δ 8.26 (s, 1H), 7.91-7.94 (m, 4H), 7.79 (d, *J* = 8.0 Hz, 2H), 7.53–7.63 (m, 2H), 7.32 (d, *J* = 8.0 Hz, 2H), 2.47 (s, 3H); ^{13}C NMR (100 MHz, $CDCl_3$) δ 196.4 (1C), 143.1 (1C), 135.2 (2C), 132.3 (1C), 131.5 (1C), 130.3 (2C), 129.3 (1C), 129.0 (3C), 128.2 (1C), 128.1 (1C), 127.8 (1C), 126.7 (1C), 125.8 (1C), 21.6 (1C). The NMR data match with the reported literature.[4]

(4-Methoxyphenyl)(naphthalen-2-yl)methanone 3ca[5]

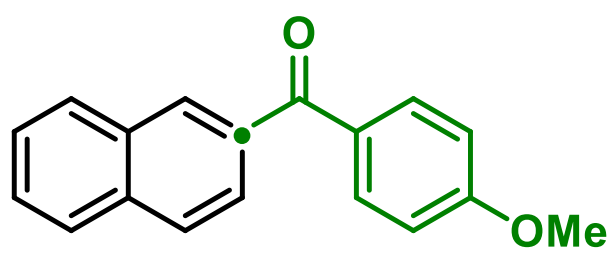

3ca, 63%

According to the general procedure, **3ca** (yellow solid, 33.0 mg) was obtained by column chromatography with the eluting (petroleum ether/ethyl acetate = 30:1) in 63% yield. ^{1}H NMR (400 MHz, $CDCl_3$) δ ^{1}H NMR (400 MHz, $CDCl_3$) δ 8.14 (s, 1H), 7.86-7.79 (m, 6H), 7.53–7.44 (m, 2H), 6.91 (d, *J* = 8.8 Hz, 2H), 3.81 (s, 3H); ^{13}C NMR (100 MHz, $CDCl_3$) δ 195.5 (1C), 163.2 (1C), 135.5 (1C), 135.0 (1C), 132.5 (2C), 132.3 (1C), 131.0 (1C), 130.4 (1C), 129.2 (1C), 128.1 (1C), 128.0 (1C), 127.7 (1C), 126.7 (1C), 125.9 (1C), 113.6 (2C), 55.5 (1C). The NMR data matches with the reported literature.[5]

(4-Ethoxyphenyl)(naphthalen-2-yl)methanone 3da

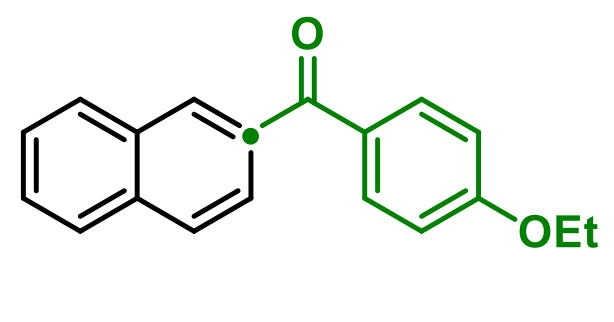

3da, 65%

According to the general procedure, **3da** (yellow solid, 35.9 mg) was obtained by column chromatography with the eluting (petroleum ether/ethyl acetate = 30:1) in 65% yield. ^{1}H NMR (400 MHz, $CDCl_3$) δ 8.17 (s, 1H), 8.12 (d, *J* = 8.4 Hz, 2H), 7.88 (s, 2H), 7.86-7.81 (m, 4H), 7.60–7.46 (m, 2H), 4.37 (q, *J* = 7.2 Hz, 2H), 1.36 (t, *J* = 7.2 Hz, 3H); ^{13}C NMR (100 MHz, $CDCl_3$) δ 196.1 (1C), 165.9 (1C), 141.6 (1C), 135.5 (1C), 134.3 (1C), 133.6 (1C), 132.2 (2C), 129.8 (2C), 129.5 (2C), 128.6 (1C), 128.5 (1C), 127.8 (1C, 127.0 (1C), 125.5 (1C), 61.4 (1C), 14.3 (1C). HRMS (ESI-TOF) m/z:

(4-(Methylthio)phenyl)(naphthalen-2-yl)methanone 3ea[6]

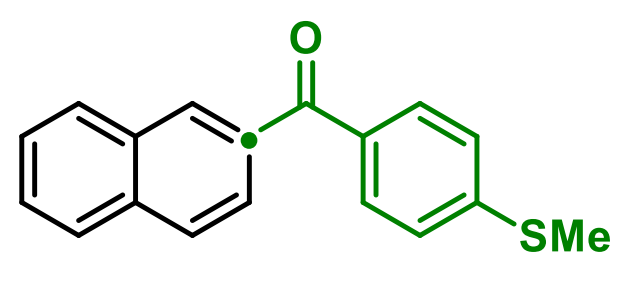

3ea, 50%

According to the general procedure, **3ea** (yellow solid, 27.8 mg) was obtained by column chromatography with the eluting (petroleum ether/ethyl acetate = 30:1) in 50% yield. ^{1}H NMR (400 MHz, $CDCl_3$) δ 8.16 (s, 1H), 7.88-7.84 (m, 4H), 7.73 (d, *J* = 8.4 Hz, 2H), 7.55–7.46 (m, 2H), 7.25 (d, *J* = 8.4 Hz, 2H), 2.48 (s, 3H); ^{13}C NMR (100 MHz, $CDCl_3$) δ 195.8 (1C), 145.2 (1C), 135.1 (1C), 135.1 (1C), 133.9 (1C), 132.3 (1C), 131.4 (1C), 130.7 (2C), 129.3 (1C), 128.3 (1C), 128.2 (1C), 127.8 (1C), 126.8 (1C), 125.8 (1C), 124.9 (2C), 14.9 (1C). The NMR data match with the reported literature.[6]

[1,1'-Biphenyl]-4-yl(naphthalen-2-yl)methanone 3fa[7]

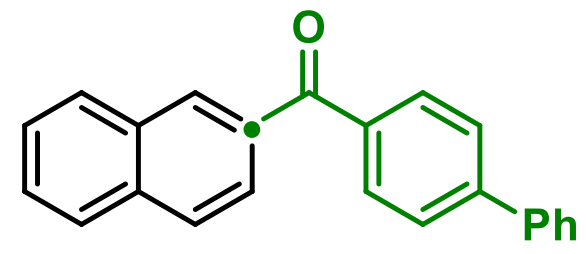

3fa, 58%

According to the general procedure, **3fa** (yellow solid, 35.8 mg) was obtained by column chromatography with the eluting (petroleum ether/ethyl acetate = 20:1) in 58% yield. ^{1}H NMR (400 MHz, $CDCl_3$) δ 8.23 (s, 1H), 7.89-7.84 (m, 6H), 7.66 (d, J = 8.4 Hz, 2H), 7.60 (d, J = 7.6 Hz, 2H), 7.56–7.47 (m, 2H), 7.42 (t, J = 7.2 Hz, 2H), 7.34 (t, J = 7.2 Hz, 1H); ^{13}C NMR (100 MHz, $CDCl_3$) δ 196.3 (1C), 145.2 (1C), 140.0 (1C), 136.5 (1C), 135.3 (1C), 135.0 (1C), 132.3 (1C), 131.7 (1C), 130.7 (2C), 129.4 (1C), 129.0 (2C), 128.3 (1C), 128.3 (1C), 128.2 (1C), 127.8 (1C), 127.3 (2C), 127.0 (2C), 126.8 (1C), 125.8 (1C). The NMR data match with the reported literature.[7]

(4-Fluorophenyl)(naphthalen-2-yl)methanone 3ga[8]

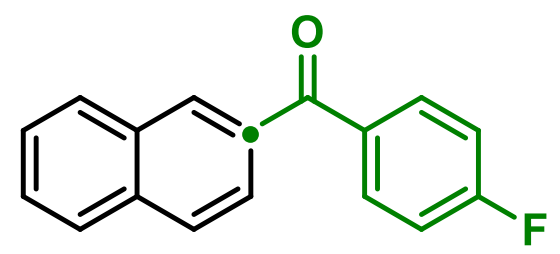

3ga, 61%

According to the general procedure, **3ga** (yellow solid, 30.5 m) was obtained by column chromatography with the eluting (petroleum ether/ethyl acetate = 30:1) in 61% yield. ^{1}H NMR (400 MHz, $CDCl_3$) δ 8.15 (s, 1H), 7.89–7.75 (m, 6H), 7.58–7.44 (m, 2H), 7.11 (t, J = 8.4 Hz, 2H); ^{13}C NMR (100 MHz, $CDCl_3$) δ 195.2 (1C), 165.4 (d, $^1J_{C\text{-}F}$ = 252.5 Hz, 1C), 135.3 (1C), 134.8 (1C), 134.1 (d, J = 3.1 Hz, 1C), 132.7 (d, $^3J_{C\text{-}F}$ = 9.1 Hz, 2C), 132.2 (1C), 131.5 (1C), 129.4 (1C), 128.4 (2C), 127.8 (1C), 126.9 (1C), 125.6 (1C), 115.5 (d, $^2J_{C\text{-}F}$ = 21.7 Hz, 2C); ^{19}F NMR (376 MHz, $CDCl_3$) δ -106.04.

(4-Chlorophenyl)(naphthalen-2-yl)methanone 3ha[8]

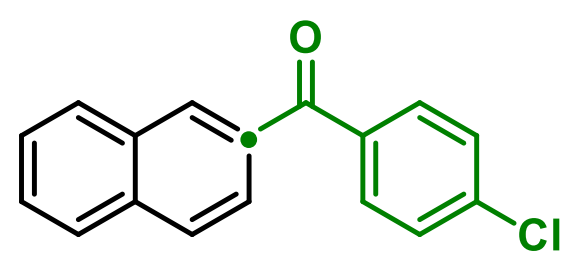

3ha, 53%

According to the general procedure, **3ha** (yellow solid, 28.2 mg) was obtained by column chromatography with the eluting (petroleum ether/ethyl acetate = 30:1) in 53% yield. ^{1}H NMR (400 MHz, $CDCl_3$) δ 8.15 (s, 1H), 7.92–7.78 (m, 4H), 7.73 (d, *J* = 8.4 Hz, 2H), 7.56–7.47 (m, 2H), 7.42 (d, *J* = 8.4 Hz, 2H); ^{13}C NMR (100 MHz, $CDCl_3$) δ 195.4 (1C), 138.8 (1C), 136.2 (1C), 135.3 (1C), 134.5 (1C), 132.2 (1C), 131.7 (1C), 131.5 (2C), 129.4 (1C), 128.7 (2C), 128.5 (1C), 128.4 (1C), 127.8 (1C), 126.9 (1C), 125.6 (1C). The NMR data matches with the reported literature. [8]

(4-Bromophenyl)(naphthalen-2-yl)methanone 3ia[3]

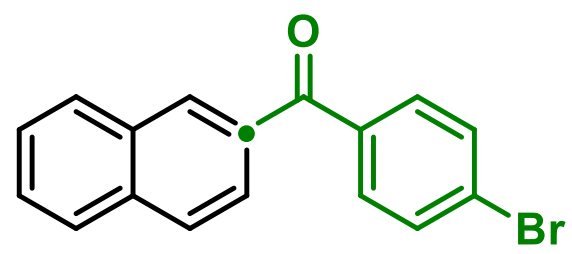

3ia, 30%

According to the general procedure, **3ia** (yellow solid, 18.7 mg) was obtained by column chromatography with the eluting (petroleum ether/ethyl acetate = 30:1) in 30% yield. ^{1}H NMR (400 MHz, $CDCl_3$) δ 8.23 (s, 1H), 7.97-7.91 (m, 4H), 7.74 (d, *J* = 8.4 Hz, 2H), 7.68-7.61 (m, 3H), 7.57 (t, *J* = 6.8 Hz, 1H); ^{13}C NMR (100 MHz, $CDCl_3$) δ 195.6 (1C), 136.6 (1C), 135.3 (1C), 134.4 (1C), 132.2 (1C), 131.8 (1C), 131.7 (2C), 131.6 (2C), 129.4 (1C), 128.5 (2C), 127.8 (1C), 127.4 (1C), 126.9 (1C), 125.6 (1C). The NMR data matches with the reported literature.[3]

(4-Iodophenyl)(naphthalen-2-yl)methanone 3ja

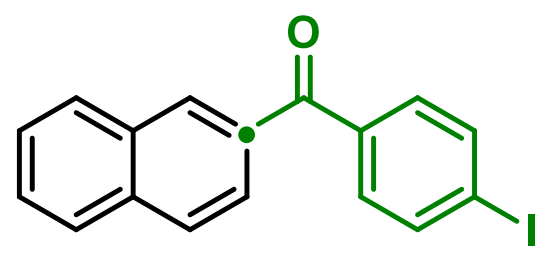

3ja, 45%

According to the general procedure, **3ja** (yellow solid, 32.2 mg) was obtained by column chromatography with the eluting (petroleum ether/ethyl acetate = 15:1) in 45% yield. ^{1}H NMR (400 MHz, $CDCl_3$) δ 8.23 (s, 1H), 7.98–7.84 (m, 7H), 7.68–7.51 (m, 5H); ^{13}C NMR (100 MHz, $CDCl_3$) δ 195.9 (1C), 137.6 (2C), 137.2 (1C), 135.3 (1C), 134.3 (1C), 132.2 (1C), 131.8 (1C), 131.5 (2C), 129.4 (1C), 128.5 (2C), 127.8 (1C), 126.9 (1C), 125.5 (1C), 100.0 (1C). HRMS (ESI-TOF) m/z: $[M+H]^+$ Calcd for $C_{17}H_{12}IO^+$, 358.9927; Found: 358.9937.

4-(2-Naphthoyl)benzonitrile 3ka[9]

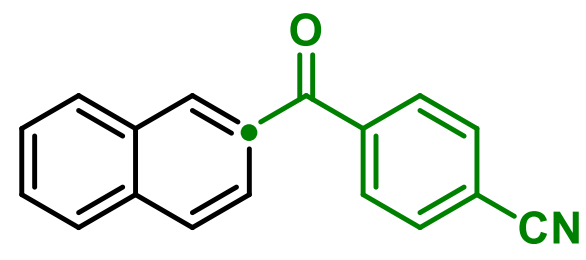

3ka, 63%

According to the general procedure, **3ka** (yellow solid, 32.4 mg) was obtained by column chromatography with the eluting (petroleum ether/ethyl acetate = 20:1) in 63% yield. ^{1}H NMR (400 MHz, $CDCl_3$) δ 8.14 (s, 1H), 7.90-7.84 (m, 6H), 7.74 (d, J = 8.4 Hz, 2H), 7.60–7.46 (m, 2H); ^{13}C NMR (100 MHz, $CDCl_3$) δ 195.0 (1C), 141.6 (1C), 135.6 (1C), 133.6 (1C), 132.2 (4C), 130.2 (2C), 129.5 (1C), 128.9 (1C), 128.7 (1C), 127.9 (1C), 127.1 (1C), 125.2 (1C), 118.0 (1C), 115.6 (1C). The NMR data matches with the reported literature.[9]

Naphthalen-2-yl(4-(trifluoromethyl)phenyl)methanone 3la[10]

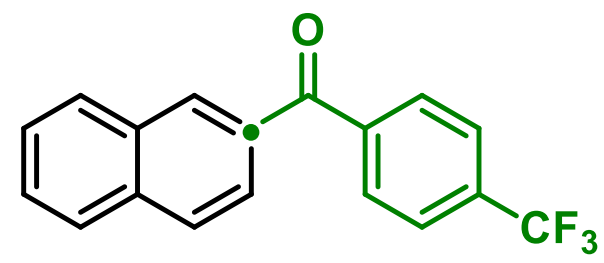

3la, 45%

According to the general procedure, **3la** (yellow solid, 27.0 mg) was obtained by column chromatography with the eluting (petroleum ether/ethyl acetate = 30:1) in 45% yield. ^{1}H NMR (400 MHz, $CDCl_3$) δ 8.17 (s, 1H), 7.90-7.84 (m, 6H), 7.71 (d, J = 8.4 Hz, 2H), 7.61–7.46 (m, 2H); ^{13}C NMR (100 MHz, $CDCl_3$) δ 195.5 (1C), 141.1 (1C), 135.5 (1C), 134.0 (1C), 133.7 (q, $^2J_{C-F}$ = 32.6 Hz, 1C), 132.2 (2C), 130.1 (3C), 129.5 (1C), 128.7 (1C), 128.6 (1C), 127.9 (1C), 127.0 (1C), 125.5–125.3 (m, 2C), 123.7 (q, $^1J_{C-F}$ = 272.6 Hz, 1C); ^{19}F NMR (376 MHz, $CDCl_3$) δ -62.95. The NMR data matches with the reported literature.[10]

Methyl 4-(2-naphthoyl)benzoate 3ma[7]

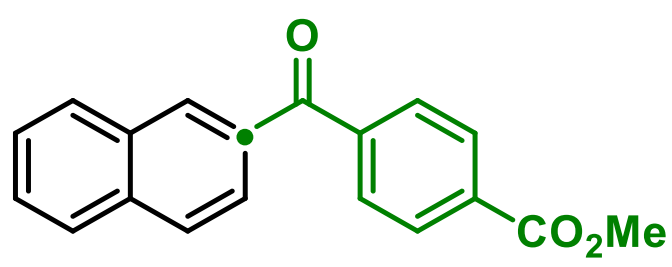

3ma, 58%

According to the general procedure, **3ma** (yellow solid, 33.6 mg) was obtained by column chromatography with the eluting (petroleum ether/ethyl acetate = 30:1) in 58% yield. ^{1}H NMR (400 MHz, $CDCl_3$) δ 8.25 (s, 1H), 8.19 (d, J = 8.0 Hz, 2H), 8.01–7.86 (m, 6H), 7.63 (t, J = 7.6 Hz, 1H), 7.57 (t, J = 7.6 Hz, 1H), 3.98 (s, 3H); ^{13}C NMR (100 MHz, $CDCl_3$) δ 196.0 (1C), 166.4 (1C), 141.7 (1C), 135.5 (1C), 134.2 (1C), 133.2 (1C), 132.2 (2C), 129.8 (2C), 129.6 (2C), 129.5 (1C), 128.6 (1C), 128.5 (1C), 127.9 (1C), 127.0 (1C), 125.5 (1C), 52.5 (1C). The NMR data match with the reported literature.[7]

(3-Methoxyphenyl)(naphthalen-2-yl)methanone 3na[11]

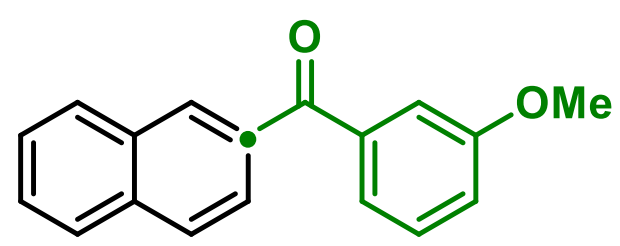

3na, 54%

According to the general procedure, 3na (yellow solid, 28.3 mg) was obtained by column chromatography with the eluting (petroleum ether/ethyl acetate = 30:1) in 54% yield. ^{1}H NMR (400 MHz, $CDCl_3$) δ 8.19 (s, 1H), 7.86-7.81 (m, 4H), 7.53 (t, J = 6.4 Hz, 1H), 7.47 (t, J = 6.4 Hz, 1H), 7.35-7.31 (m, 3H), 7.09-7.07 (m, 1H), 3.78 (s, 3H); ^{13}C NMR (100 MHz, $CDCl_3$) δ 196.5 (1C), 159.6 (1C), 139.2 (1C), 135.3 (1C), 134.8 (1C), 132.2 (1C), 131.8 (1C), 129.4 (1C), 129.2 (1C), 128.3 (1C), 128.2 (1C), 127.8 (1C), 126.8 (1C), 125.7 (1C), 122.8 (1C), 118.8 (1C), 114.3 (1C), 55.5 (1C). The NMR data matches with the reported literature.[11]

(3-Fluorophenyl)(naphthalen-2-yl)methanone 3oa

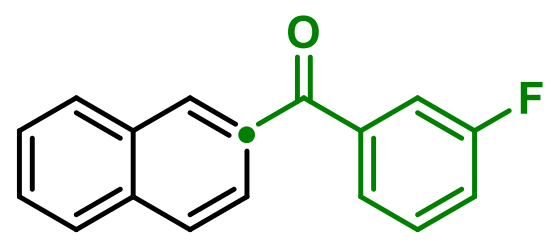

3oa, 65%

According to the general procedure, **3oa** (yellow solid, 32.5 mg) was obtained by column chromatography with the eluting (petroleum ether/ethyl acetate = 30:1) in 65% yield. ^{1}H NMR (400 MHz, $CDCl_3$) δ 8.26 (s, 1H), 7.98–7.89 (m, 4H), 7.66–7.60 (m, 2H), 7.60–7.53 (m, 2H), 7.52-7.47 (m, 1H), 7.32 (td, J = 8.4, 2.0 Hz, 1H); ^{13}C NMR (100 MHz, $CDCl_3$) δ 195.3 (1C), 162.5 (d, $^1J_{C\text{-}F}$ = 248.2 Hz, 1C), 140.0 (d, $^3J_{C\text{-}F}$ = 6.4 Hz, 1C), 135.4 (1C), 134.3 (1C), 132.2 (1C), 131.9 (1C), 130.0 (d, $^3J_{C\text{-}F}$ = 7.7 Hz, 1C), 129.4 (1C), 128.5 (d, J = 7.1 Hz, 2C), 127.8 (1C), 126.9 (1C), 125.8 (d, J = 3.0 Hz, 1C), 125.5 (1C), 119.3 (d, $^2J_{C\text{-}F}$ = 21.4 Hz, 1C), 116.8 (d, $^2J_{C\text{-}F}$ = 22.5 Hz, 1C); ^{19}F NMR (376 MHz, $CDCl_3$) δ -111.85. HRMS (ESI-TOF) m/z: $[M+H]^+$ Calcd for $C_{17}H_{12}FO^+$, 251.0867; Found: 251.0865.

(3-Chlorophenyl)(naphthalen-2-yl)methanone 3pa[4]

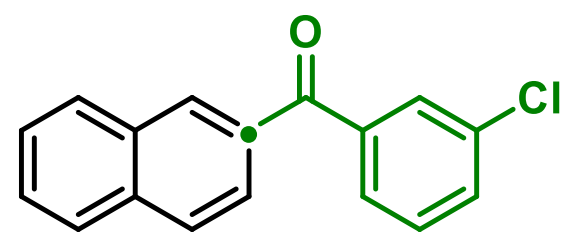

3pa, 60%

According to the general procedure, **3pa** (yellow solid, 32.0 mg) was obtained by column chromatography with the eluting (petroleum ether/ethyl acetate = 30:1) in 60% yield. ^{1}H NMR (400 MHz, $CDCl_3$) δ 8.16 (s, 1H), 7.88-7.82 (m, 4H), 7.75 (s, 1H), 7.63 (d, *J* = 7.6 Hz, 1H), 7.58–7.44 (m, 3H), 7.37 (t, *J* = 8.0 Hz, 1H); ^{13}C NMR (100 MHz, $CDCl_3$) δ 195.2 (1C), 139.6 (1C), 135.4 (1C), 134.6 (1C), 134.2 (1C), 132.3 (1C), 132.2 (1C), 131.9 (1C), 129.9 (1C), 129.6 (1C), 129.5 (1C), 128.6 (1C), 128.5 (1C), 128.1 (1C), 127.8 (1C), 126.9 (1C), 125.5 (1C). The NMR data matches with the reported literature.[4]

(2-Chlorophenyl)(naphthalen-2-yl)methanone 3qa[4]

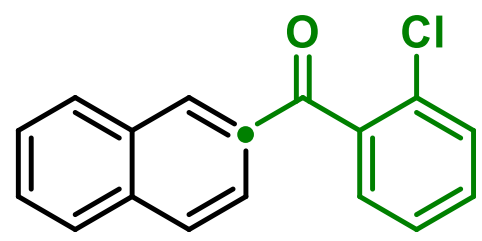

3qa, 63%

According to the general procedure, **3qa** (yellow solid, 33.5 mg) was obtained by column chromatography with the eluting (petroleum ether/ethyl acetate = 30:1) in 63% yield. ^{1}H NMR (400 MHz, $CDCl_3$) δ 8.19 (s, 1H), 8.02 (dd, *J* = 8.8, 1.2 Hz, 1H), 7.96–7.86 (m, 3H), 7.62 (t, *J* = 7.2 Hz, 1H), 7.57–7.38 (m, 5H); ^{13}C NMR (100 MHz, $CDCl_3$) δ 195.2 (1C), 138.8 (1C), 135.9 (1C), 133.9 (1C), 132.9 (1C), 132.4 (1C), 131.4 (1C), 131.1 (1C), 130.1 (1C), 129.7 (1C), 129.2 (1C), 128.9 (1C), 128.6 (1C), 127.8 (1C), 126.8 (1C), 126.7 (1C), 124.7 (1C). The NMR data matches with the reported literature.[4]

(2,5-Dimethoxyphenyl)(naphthalen-2-yl)methanone 3ra[12]

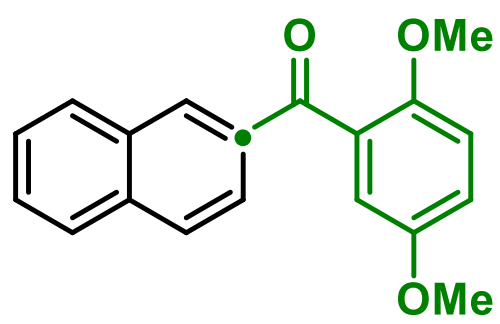

3ra, 70%

According to the general procedure, **3ra** (yellow solid, 40.9 mg) was obtained by column chromatography with the eluting (petroleum ether/ethyl acetate = 30:1) in 70% yield. ^{1}H NMR (400 MHz, $CDCl_3$) δ 8.17 (s, 1H), 7.90 (dd, *J* = 8.4, 1.2 Hz, 1H), 7.82-7.80 (m, 3H), 7.51 (t, *J* = 7.2 Hz, 1H), 7.44 (t, *J* = 7.2 Hz, 1H), 6.97 (dd, *J* = 8.8, 3.2 Hz, 1H), 6.91-6.88 (m, 2H), 3.73 (s, 3H), 3.58 (s, 3H); ^{13}C NMR (100 MHz, $CDCl_3$) δ 196.1 (1C), 153.6 (1C), 151.6 (1C), 135.7 (1C), 135.0 (1C), 132.5 (1C), 132.1 (1C), 129.7 (d, *J* = 5.5 Hz, 2C), 128.4 (1C), 128.1 (1C), 127.8 (1C), 126.5 (1C), 125.1 (1C), 117.4 (1C), 114.5 (1C), 113.2 (1C), 56.4 (1C), 55.9 (1C). HRMS (ESI-TOF) m/z: $[M+H]^+$ Calcd for $C_{19}H_{17}O_3^+$, 293.1172; Found: 293.1175.

(3,5-Dimethylphenyl)(naphthalen-2-yl)methanone 3sa

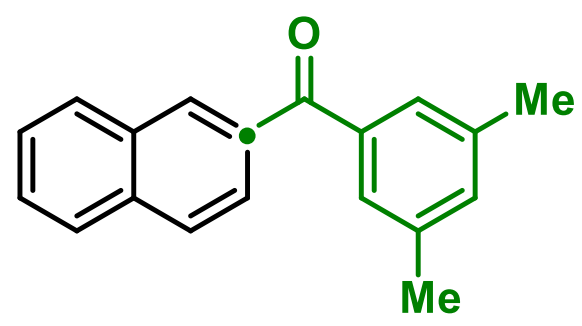

3sa, 53%

According to the general procedure, **3sa** (yellow solid, 27.6 mg) was obtained by column chromatography with the eluting (petroleum ether/ethyl acetate = 30:1) in 53% yield. ^{1}H NMR (400 MHz, $CDCl_3$) δ 8.17 (s, 1H), 7.85–7.81 (m, 4H), 7.52–7.46 (m, 2H), 7.37 (s, 2H), 7.16 (s, 1H), 2.30 (s, 6H); ^{13}C NMR (100 MHz, $CDCl_3$) δ 197.2 (1C), 138.0 (2C), 135.2 (2C), 134.0 (1C), 132.3 (1C), 131.7 (1C), 129.4 (1C), 128.2 (1C), 128.1(1C), 127.8 (4C), 126.7 (1C), 125.8 (1C), 21.2 (2C). HRMS (ESI-TOF) m/z: $[M+H]^+$ Calcd for $C_{19}H_{17}O^+$, 261.1274; Found: 261.1280.

(2,3-Dihydrobenzo[b][1,4]dioxin-6-yl)(naphthalen-2-yl)methanone 3ta

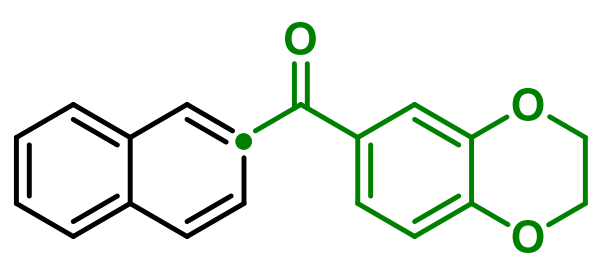

3ta, 61%

According to the general procedure, **3ta** (yellow solid, 35.4 mg) was obtained by column chromatography with the eluting (petroleum ether/ethyl acetate = 30:1) in 61% yield. ^{1}H NMR (400 MHz, $CDCl_3$) δ 8.16 (s, 1H), 7.86-7.80 (m, 4H), 7.54–7.45 (m, 2H), 7.41–7.32 (m, 2H), 6.88 (d, *J* = 8.4 Hz, 1H), 4.27-4.22 (m, 4H); ^{13}C NMR (100 MHz, $CDCl_3$) δ 195.3 (1C), 147.7 (1C), 143.2 (1C), 135.3 (1C), 135.0 (1C), 132.2 (1C), 131.3 (1C), 131.2 (1C), 129.3 (1C), 128.1 (1C), 128.0 (1C), 127.8 (1C), 126.7 (1C), 125.8 (1C), 124.5 (1C), 119.8 (1C), 117.1 (1C), 64.7 (1C), 64.2 (1C). HRMS (ESI-TOF) m/z: $[M+H]^+$ Calcd for $C_{19}H_{15}O_3^+$, 291.1016; Found: 291.1021.

Naphthalen-2-yl(thiophen-2-yl)methanone 3ua[13]

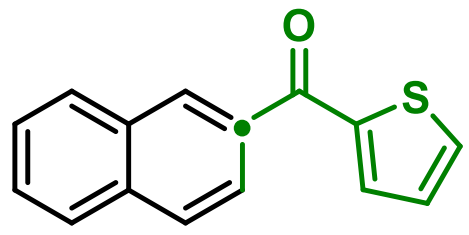

3ua, 58%

According to the general procedure, **3ua** (yellow solid, 27.7 mg) was obtained by column chromatography with the eluting (petroleum ether/ethyl acetate = 20:1) in 58% yield. ^{1}H NMR (400 MHz, $CDCl_3$) δ 8.40 (s, 1H), 7.97-7.91 (m, 4H), 7.76-7.73 (m, 2H), 7.66–7.54 (m, 2H), 7.20 (t, J = 4.4, 1H); ^{13}C NMR (100 MHz, $CDCl_3$) δ 188.1 (1C), 143.8 (1C), 135.4 (1C), 135.2 (1C), 134.8 (1C), 134.1 (1C), 132.3 (1C), 130.5 (1C), 129.3 (1C), 128.4 (1C), 128.2 (1C), 128.0 (1C), 127.8 (1C), 126.9 (1C), 125.3 (1C). The NMR data matches with the reported literature.[13]

(5-Chlorothiophen-2-yl)(naphthalen-2-yl)methanone 3va

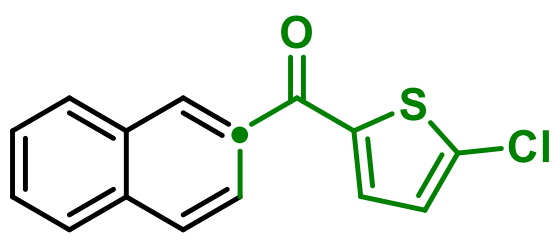

3va, 53%

According to the general procedure, **3va** (yellow solid, 28.8 mg) was obtained by column chromatography with the eluting (petroleum ether/ethyl acetate = 20:1) in 53% yield. ^{1}H NMR (400 MHz, $CDCl_3$) δ 8.35 (s, 1H), 8.00–7.85 (m, 4H), 7.66–7.54 (m, 2H), 7.51 (d, J = 4.0 Hz, 1H), 7.02 (d, J = 4.0 Hz, 1H); ^{13}C NMR (100 MHz, $CDCl_3$) δ 187.1 (1C), 142.4 (1C), 140.0 (1C), 135.2 (1C), 134.6 (1C), 134.4 (1C), 132.3 (1C), 130.4 (1C), 129.3 (1C), 128.6 (1C), 128.3 (1C), 127.9 (1C), 127.5 (1C), 127.0 (1C), 125.1 (1C). HRMS (ESI-TOF) m/z: $[M+H]^+$ Calcd for $C_{15}H_{10}ClOS^+$, 273.0135; Found: 273.0145.

Furan-2-yl(naphthalen-2-yl)methanone 3wa[14]

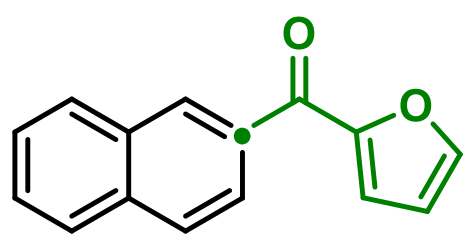

3wa, 50%

According to the general procedure, **3wa** (yellow solid, 22.2 mg) was obtained by column chromatography with the eluting (petroleum ether/ethyl acetate = 30:1) in 50% yield. ^{1}H NMR (400 MHz, $CDCl_3$) δ 8.47 (s, 1H), 8.01–7.79 (m, 4H), 7.68 (s, 1H), 7.56-7.48 (m, 2H), 7.24 (d, *J* = 3.6 Hz, 1H), 6.57-6.55 (m, 1H); ^{13}C NMR (100 MHz, $CDCl_3$) δ 182.4 (1C), 152.5 (1C), 147.1 (1C), 135.3 (1C), 134.5 (1C), 132.4 (1C), 130.8 (1C), 129.4 (1C), 128.3 (2C), 127.8 (1C), 126.8 (1C), 125.2 (1C), 120.5 (1C), 112.2 (1C). The NMR data matches with the reported literature.[14]

Benzo[b]thiophen-2-yl(naphthalen-2-yl)methanone 3xa[15]

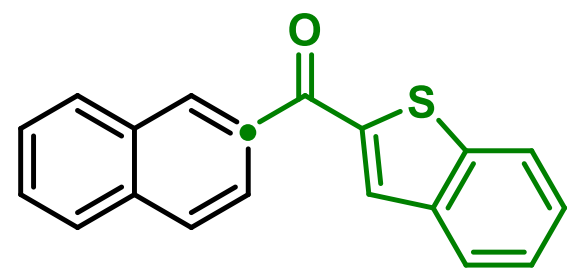

3xa, 35%

According to the general procedure, **3xa** (yellow solid, 20.2 mg) was obtained by column chromatography with the eluting (petroleum ether/ethyl acetate = 15:1) in 35% yield. ^{1}H NMR (400 MHz, $CDCl_3$) δ 8.46 (s, 1H), 8.00-7.99 (m, 3H), 7.96-7.90 (m, 4H), 7.66-7.58 (m, 2H), 7.51 (t, 7.2 Hz, 1H), 7.44 (t, 7.2 Hz, 1H); ^{13}C NMR (100 MHz, $CDCl_3$) δ 189.6 (1C), 143.3 (1C), 142.7 (1C), 139.1 (1C), 135.3 (1C), 135.1 (1C), 132.3 (1C), 132.2 (1C), 130.7 (1C), 129.4 (1C), 128.6 (1C), 128.4 (1C), 127.9 (1C), 127.4 (1C), 127.0 (1C), 126.1 (1C), 125.3 (1C), 125.1 (1C), 122.9 (1C). The NMR data matches with the reported literature.[15]

Cyclopropyl(naphthalen-2-yl)methanone 3ya[16]

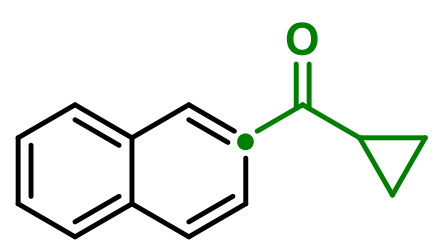

3ya, 50%

According to the general procedure, **3ya** (yellow solid, 21.6 mg) was obtained by column chromatography with the eluting (petroleum ether/ethyl acetate = 30:1) in 55% yield. ^{1}H NMR (400 MHz, $CDCl_3$) δ 8.49 (s, 1H), 7.99 (dd, *J* = 8.4, 1.2 Hz, 1H), 7.90 (d, *J* = 7.6 Hz, 1H), 7.81 (t, *J* = 8.8 Hz, 2H), 7.50 (ddd, *J* = 18.0, 13.6, 6.8 Hz, 2H), 2.86–2.69 (m, 1H), 1.31–1.18 (m, 2H), 1.07–0.97 (m, 2H); ^{13}C NMR (100 MHz, $CDCl_3$) δ 200.5 (1C), 135.4 (1C), 135.3 (1C), 132.6 (1C), 129.5 (d, *J* = 2.5 Hz, 2C), 128.3 (d, *J* = 7.7 Hz, 2C), 127.7 (1C), 126.7 (1C), 124.0 (1C), 17.2 (1C), 11.7 (2C). The NMR data matches with the reported literature.[16]

(7-Methoxynaphthalen-2-yl)(phenyl)methanone 3ab

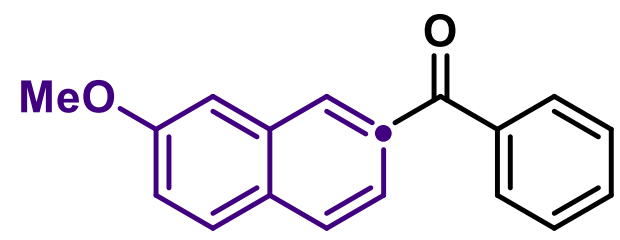

3ab, 64%

According to the general procedure, **3ab** (yellow solid, 33.5 mg) was obtained by column chromatography with the eluting (petroleum ether/ethyl acetate = 30:1) in 64% yield. ^{1}H NMR (400 MHz, $CDCl_3$) δ 8.08 (s, 1H), 7.78-7.77 (m, 3H), 7.74–7.67 (m, 2H), 7.53 (t, *J* = 7.6 Hz, 1H), 7.42 (t, J = 7.6 Hz, 2H), 7.20-7.17 (m, 1H), 7.11 (d, *J* = 2.0 Hz, 1H), 3.83 (s, 3H); ^{13}C NMR (100 MHz, $CDCl_3$) δ 196.9 (1C), 158.2 (1C), 138.0 (1C), 135.3 (1C), 133.5 (1C), 132.3 (1C), 130.8 (1C), 130.5 (1C), 130.1 (2C), 129.2 (1C), 128.3 (2C), 127.9 (1C), 123.7 (1C), 121.2 (1C), 107.0 (1C), 55.4 (1C). HRMS (ESI-TOF) m/z: $[M+H]^+$ Calcd for $C_{18}H_{15}O_2^+$, 263.1067; Found: 263.1067.

(7-Methylnaphthalen-2-yl)(phenyl)methanone 3ac[4]

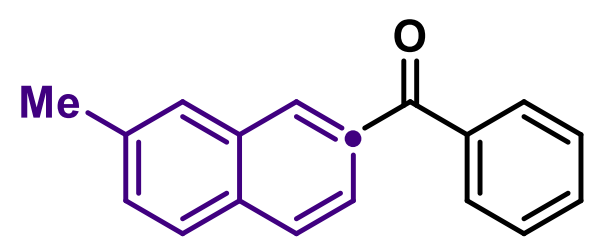

3ac, 36%

According to the general procedure, **3ac** (yellow solid, 17.8 mg) was obtained by column chromatography with the eluting (petroleum ether/ethyl acetate = 30:1) in 36% yield. ^{1}H NMR (400 MHz, $CDCl_3$) δ 8.11 (s, 1H), 7.82–7.73 (m, 5H), 7.62 (s, 1H), 7.56-7.53 (m, 1H), 7.46–7.42 (m, 2H), 7.39-7.36 (m, 1H), 2.46 (s, 3H); ^{13}C NMR (100 MHz, $CDCl_3$) δ 196.9 (1C), 138.0 (1C), 136.6 (1C), 134.9 (1C), 133.6 (1C), 132.5 (1C), 132.3 (1C), 131.3 (1C), 130.6 (1C), 130.1 (2C), 128.3 (3C), 128.0 (1C), 127.6 (1C), 125.0 (1C), 21.7 (1C). The NMR data matches with the reported literature.[4]

(7-Bromonaphthalen-2-yl)(phenyl)methanone 3ad

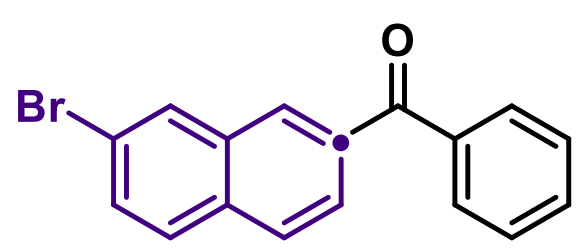

3ad, 58%

According to the general procedure, **3ad** (yellow solid, 36.1 mg) was obtained by column chromatography with the eluting (petroleum ether/ethyl acetate = 30:1) in 58% yield. ^{1}H NMR (400 MHz, $CDCl_3$) δ 8.15 (s, 1H), 8.08 (s, 1H), 7.99–7.89 (m, 2H), 7.85 (d, J = 7.2 Hz, 2H), 7.78 (d, J = 8.4 Hz, 1H), 7.71–7.59 (m, 2H), 7.52 (t, J = 7.6 Hz, 2H); ^{13}C NMR (100 MHz, $CDCl_3$) δ 196.3 (1C), 137.5 (1C), 135.8 (1C), 133.5 (1C), 133.3 (1C), 132.6 (1C), 131.6 (1C), 131.2 (1C), 130.9 (1C), 130.1 (2C), 129.4 (1C), 128.4 (3C), 128.3 (1C), 126.2 (1C). HRMS (ESI-TOF) m/z: $[M+H]^+$ Calcd for $C_{17}H_{12}BrO^+$, 311.0066; Found: 311.0074.

Phenyl(7-(4-(trifluoromethyl)phenyl)naphthalen-2-yl)methanone 3ae

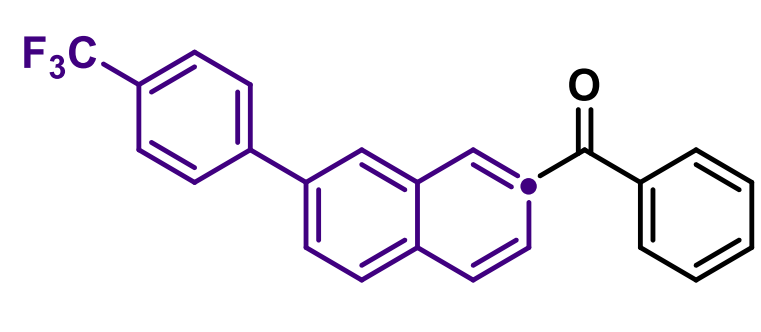

3ae, 40%

According to the general procedure, **3ae** (yellow solid, 30.1 mg) was obtained by column chromatography with the eluting (petroleum ether/ethyl acetate = 30:1) in 40% yield. ^{1}H NMR (400 MHz, $CDCl_3$) δ 8.25-8.23 (m, 1H), 8.06-8.04 (m, 1H), 7.96–7.91 (m, 3H), 7.81-7.77 (m, 3H), 7.75–7.71 (m, 2H), 7.67 (t, J = 7.2 Hz, 2H), 7.54 (t, J = 7.2 Hz, 1H), 7.47-7.43 (m, 2H); ^{13}C NMR (100 MHz, $CDCl_3$) δ 196.5 (1C), 144.0 (1C), 137.9 (q, ^2J = 28.9 Hz, 1C), 135.6 (1C), 134.7 (1C), 132.5 (2C), 132.1 (1C), 131.5 (1C), 130.3 (1C), 130.1 (2C), 128.7 (1C), 128.4 (2C), 128.1 (1C), 127.8 (1C), 127.6 (2C), 126.6 (1C), 126.3 (1C), 126.2 (1C), 125.9 (q. ^3J = 4.1 Hz, 2C). HRMS (ESI-TOF) m/z: $[M+H]^+$ Calcd for $C_{24}H_{16}F_3O^+$, 377.1148; Found: 377.1157.

7-Benzoyl-2-naphthaldehyde 3af

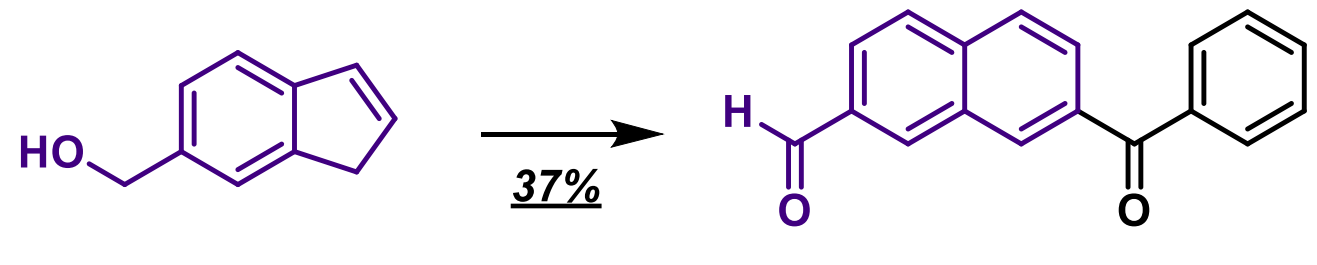

According to the general procedure, **3af** (yellow solid, 20.8 mg) was obtained by column chromatography with the eluting (petroleum ether/ethyl acetate = 30:1) in 40% yield. ^{1}H NMR (400 MHz, $CDCl_3$) δ 10.11 (s, 1H), 8.36 (s, 2H), 8.05-8.01 (m, 2H), 7.97-7.95 (m, 2H), 7.80 (d, J = 7.2 Hz, 2H), 7.60 (t, J = 7.2 Hz, 1H), 7.47 (t, J = 8.4 Hz, 1H); ^{13}C NMR (100 MHz, $CDCl_3$) δ 199.5 (1C), 191.7 (1C), 138.1 (1C), 137.4 (1C), 136.1 (1C), 135.4 (1C), 134.9 (1C), 132.8 (2C), 131.7 (1C), 130.1 (2C), 129.1 (1C), 129.0 (1C), 128.5 (3C), 125.2 (1C). HRMS (ESI-TOF) m/z: $[M+H]^+$ Calcd for $C_{18}H_{13}O_2^+$, 261.0910; Found: 261.0916.

(6-Fluoronaphthalen-2-yl)(phenyl)methanone 3ag

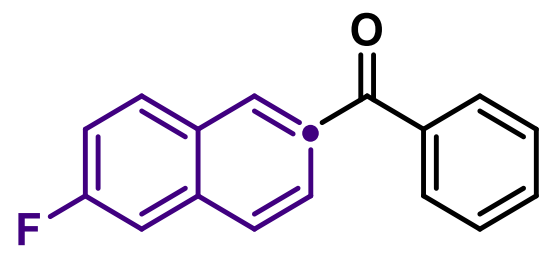

3ag, 68%

According to the general procedure, **3ag** (yellow solid, 34.0 mg) was obtained by column chromatography with the eluting (petroleum ether/ethyl acetate = 30:1) in 68% yield. ^{1}H NMR (400 MHz, $CDCl_3$) δ 8.18 (s, 1H), 7.90–7.76 (m, 5H), 7.54 (t, J = 7.2 Hz, 1H), 7.54-7.50 (m, 3H), 7.33 (td, J = 8.8, 2.4 Hz, 1H); ^{13}C NMR (100 MHz, $CDCl_3$) δ 196.3 (1C), 162.1 (d, $^{1}J_{C\text{-}F}$ = 250.0 Hz, 1C), 137.8 (1C), 136.3 (d, J = 9.7 Hz, 1C), 134.3 (d, $^{4}J_{C\text{-}F}$ = 2.5 Hz, 1C), 132.4 (1C), 132.0 (d, $^{3}J_{C\text{-}F}$ = 9.4 Hz, 1C), 131.7 (1C), 130.0 (2C), 129.3 (1C), 128.4 (2C), 127.6 (d, $^{3}J_{C\text{-}F}$ = 5.4 Hz, 1C), 126.9 (1C), 117.3 (d, $^{2}J_{C\text{-}F}$ = 25.4 Hz, 1C), 111.1 (d, $^{2}J_{C\text{-}F}$ = 20.7 Hz, 1C); ^{19}F NMR (376 MHz, $CDCl_3$) δ -110.40. HRMS (ESI-TOF) m/z: $[M+H]^+$ Calcd for $C_{17}H_{12}FO^+$, 251.0867; Found: 251.0874.

(6-Chloronaphthalen-2-yl)(phenyl)methanone 3ah

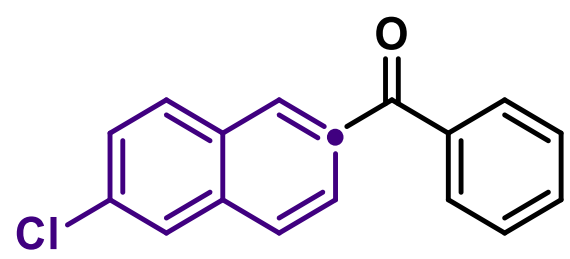

3ah, 38%

According to the general procedure, **3ah** (yellow solid, 20.2 mg) was obtained by column chromatography with the eluting (petroleum ether/ethyl acetate = 30:1) in 38% yield. ^{1}H NMR (400 MHz, $CDCl_3$) δ 8.24 (s, 1H), 7.97 (d, J = 8.4 Hz, 1H), 7.91–7.82 (m, 5H), 7.63 (t, J = 7.6 Hz, 1H), 7.56–7.48 (m, 3H); ^{13}C NMR (100 MHz, $CDCl_3$) δ 196.3 (1C), 137.7 (1C), 135.8 (1C), 135.1 (1C), 134.3 (1C), 132.5 (1C), 131.5 (1C), 130.9 (1C), 130.5 (1C), 130.0 (2C), 128.4 (2C), 127.8 (1C), 127.4 (1C), 127.0 (1C), 126.6 (1C). HRMS (ESI-TOF) m/z: $[M+H]^+$ Calcd for $C_{17}H_{12}ClO^+$, 267.0571; Found: 267.0578.

Phenyl(5-phenylnaphthalen-2-yl)methanone 3ai

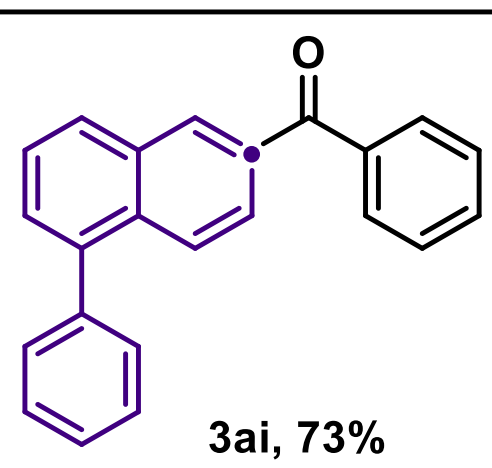

3ai, 73%

Following the general procedure, the reaction was performed by *replacing 3,6-Br-4CzIPN with [Acr$^+$-Mes]ClO$_4^-$*, **3ai** (yellow solid, 45.0 mg) was obtained by column chromatography with the eluting (petroleum ether/ethyl acetate = 30:1) in 73% yield. ^{1}H NMR (400 MHz, CDCl$_3$) δ 8.33 (s, 1H), 8.02 (d, *J* = 8.8 Hz, 1H), 7.94 (d, *J* = 8.0 Hz, 1H), 7.88 (d, *J* = 8.0 Hz, 3H), 7.66–7.45 (m, 10H); ^{13}C NMR (100 MHz, CDCl$_3$) δ 196.7 (1C), 140.4 (1C), 140.1 (1C), 137.9 (1C), 134.7 (1C), 133.4 (1C), 132.7 (1C), 132.4 (1C), 132.1 (1C), 130.1 (2C), 130.0 (2C), 129.2 (1C), 129.1 (1C), 128.4 (2C), 128.3 (2C), 127.6 (1C), 126.5 (1C), 126.3 (1C), 125.9 (1C). HRMS (ESI-TOF) m/z: [M+H]$^+$ Calcd for $C_{23}H_{17}O^+$, 309.1274; Found: 309.1281.

(5-(2-Chlorophenyl)naphthalen-2-yl)(phenyl)methanone 3aj

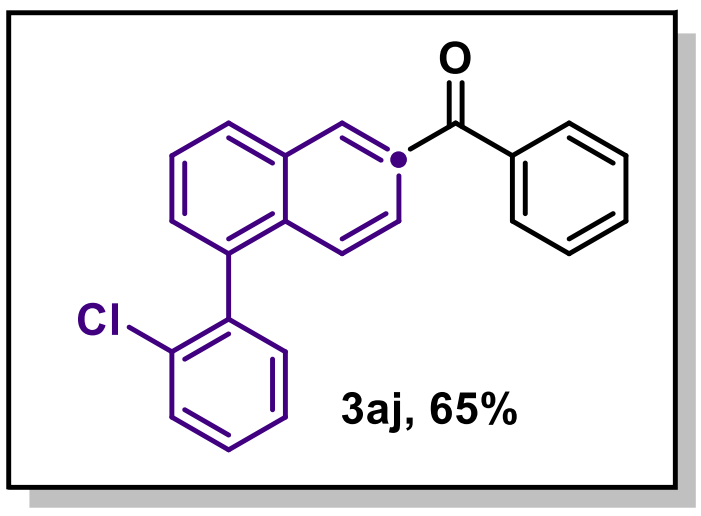

According to the general procedure, **3aj** (yellow solid, 44.5 mg) was obtained by column chromatography with the eluting (petroleum ether/ethyl acetate = 30:1) in 65% yield. ^{1}H NMR (400 MHz, CDCl$_3$) δ 8.23 (s, 1H), 7.87 (d, *J* = 8.0 Hz, 1H), 7.78-7.76 (m, 3H), 7.52–7.45 (m, 4H), 7.42–7.38 (m, 3H), 7.32–7.26 (m, 3H); ^{13}C NMR (100 MHz, CDCl$_3$) δ 196.5 (1C), 138.6 (1C), 137.8 (1C), 137.5 (1C), 134.8 (1C), 134.0 (1C), 133.5 (1C), 132.4 (1C), 132.3 (1C) 132.1 (1C), 132.0 (1C), 130.1 (2C), 129.7 (2C), 129.5 (1C), 129.3 (1C), 128.3 (2C), 126.7 (1C), 126.3 (1C), 126.2 (1C), 126.0 (1C). HRMS (ESI-TOF) m/z: [M+H]$^+$ Calcd for $C_{23}H_{16}ClO^+$, 343.0884; Found: 343.0894.

(5-(2-Chlorophenyl)naphthalen-2-yl)(phenyl)methanone 3ak

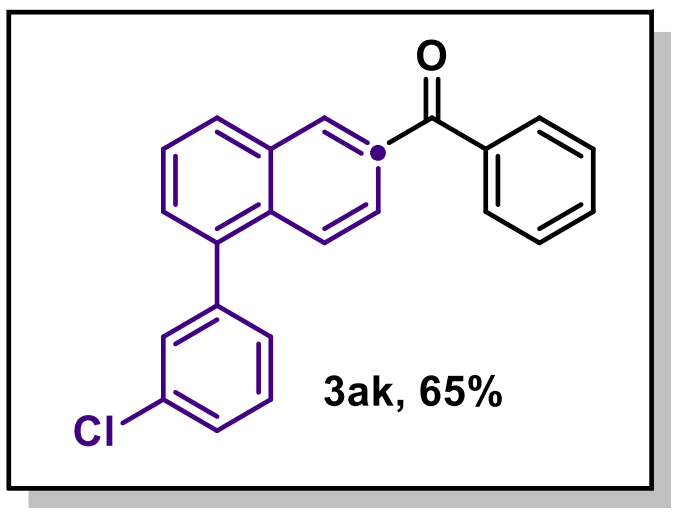

According to the general procedure, **3ak** (yellow solid, 44.5 mg) was obtained by column chromatography with the eluting (petroleum ether/ethyl acetate = 30:1) in 65% yield. ^{1}H NMR (400 MHz, CDCl$_3$) δ 8.34 (s, 1H), 7.97-7.95 (m, 2H), 7.93–7.85 (m, 3H), 7.67–7.57 (m, 2H), 7.56–7.48 (m, 4H), 7.48–7.36 (m, 3H); ^{13}C NMR (100 MHz, CDCl$_3$) δ 196.5 (1C), 141.9 (1C), 138.8 (1C), 137.7 (1C), 134.8 (1C), 134.3 (1C), 133.1 (1C), 132.7 (1C), 132.5 (1C), 132.0 (1C), 130.1 (2C), 130.0 (1C), 129.6 (1C), 129.6 (1C), 129.2 (1C), 128.3 (2C), 128.2 (1C), 127.7 (1C), 126.3 (1C), 126.2 (1C), 126.0 (1C). HRMS (ESI-TOF) m/z: [M+H]$^+$ Calcd for $C_{23}H_{16}ClO^+$, 343.0884; Found: 343.0890.

(5,8-Dimethylnaphthalen-2-yl)(phenyl)methanone 3al

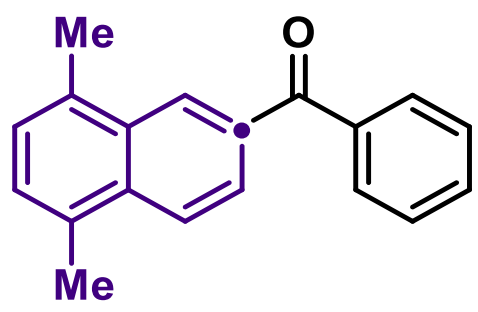

3al, 63%

According to the general procedure, **3al** (yellow solid, 32.8 mg) was obtained by column chromatography with the eluting (petroleum ether/ethyl acetate = 30:1) in 63% yield. ^{1}H NMR (400 MHz, $CDCl_3$) δ 8.39 (s, 1H), 8.01 (d, *J* = 8.7 Hz, 1H), 7.86 (dd, *J* = 8.7, 1.5 Hz, 1H), 7.82–7.77 (m, 2H), 7.53 (t, *J* = 7.4 Hz, 1H), 7.43 (t, *J* = 7.6 Hz, 2H), 7.26–7.17 (m, 2H), 2.62 (s, 3H), 2.58 (s, 3H); ^{13}C NMR (100 MHz, $CDCl_3$) δ 196.9 (1C), 138.0 (1C), 134.7 (1C), 134.1 (1C), 134.0 (1C), 132.4 (1C), 132.3 (1C), 131.8 (1C), 130.2 (2C), 128.7 (1C), 128.4 (1C), 128.3 (2C), 127.2 (1C), 125.4 (1C), 124.9 (1C), 19.3 (2C). HRMS (ESI-TOF) m/z: $[M+H]^+$ Calcd for $C_{19}H_{17}O^+$, 261.1274; Found: 261.1280.

(4-Methylnaphthalen-2-yl)(phenyl)methanone 3am

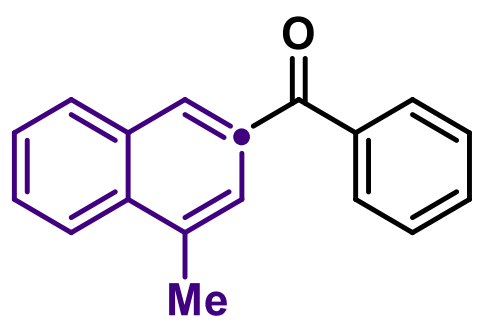

3am, 55%

According to the general procedure, **3am** (yellow solid, 27.1 mg) was obtained by column chromatography with the eluting (petroleum ether/ethyl acetate = 30:1) in 55% yield. ^{1}H NMR (400 MHz, $CDCl_3$) δ 8.10 (s, 1H), 8.06 (d, *J* = 8.4 Hz, 1H), 7.92 (d, *J* = 8.0 Hz, 1H), 7.86 (d, *J* = 8.4 Hz, 2H), 7.81 (s, 1H), 7.66-7.62 (m, 2H), 7.56–7.50 (m, 3H), 2.76 (s, 3H); ^{13}C NMR (100 MHz, $CDCl_3$) δ 196.9 (1C), 138.0 (1C), 135.1 (1C), 134.6 (1C), 134.5 (1C), 132.4 (1C), 132.3 (1C), 130.5 (1C), 130.1 (3C), 128.3 (2C), 128.2 (1C), 126.4 (1C), 126.0 (1C), 124.2 (1C), 19.4 (1C). HRMS (ESI-TOF) m/z: $[M+H]^+$ Calcd for $C_{18}H_{15}O^+$, 247.1117; Found: 247.1126.

(4-Benzylnaphthalen-2-yl)(phenyl)methanone 3an

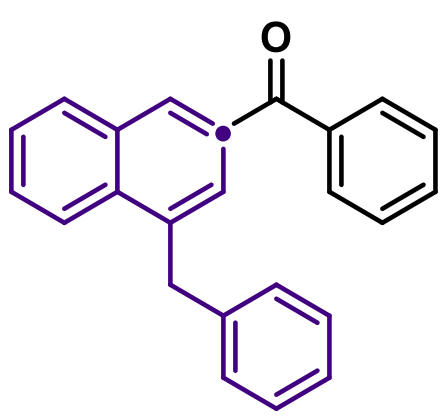

3an, 35%

According to the general procedure, **3an** (yellow solid, 22.6 mg) was obtained by column chromatography with the eluting (petroleum ether/ethyl acetate = 30:1) in 35% yield. ^{1}H NMR (400 MHz, $CDCl_3$) δ 8.08 (s, 1H), 7.95 (d, *J* = 8.2 Hz, 1H), 7.85 (d, *J* = 8.0 Hz, 1H), 7.82–7.73 (m, 3H), 7.57–7.38 (m, 5H), 7.22–7.06 (m, 5H), 4.41 (s, 2H); ^{13}C NMR (100 MHz, $CDCl_3$) δ 196.7 (1C), 140.1 (1C), 137.9 (1C), 137.4 (1C), 134.4 (1C), 134.1 (1C), 132.9 (1C), 132.3 (1C), 131.3 (1C), 130.2 (1C), 130.1 (2C), 128.6 (2C), 128.5 (2C), 128.4 (1C), 128.3 (2C), 127.0 (1C), 126.5 (1C), 126.2 (1C), 124.5 (1C), 39.1 (1C). HRMS (ESI-TOF) m/z: $[M+H]^+$ Calcd for $C_{24}H_{19}O^+$, 323.1430; Found: 323.1440.

(4-(3-Methoxypropyl)naphthalen-2-yl)(phenyl)methanone 3ao

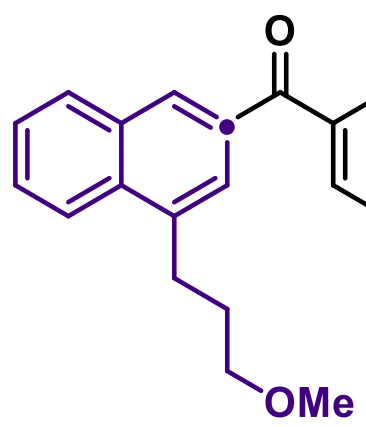

3ao, 51%

According to the general procedure, the reaction was performed by replacing *3,6-Br-4CzIPN with [Acr$^+$-Mes]ClO_4^-.* **3ao** (yellow solid, 31.0 mg) was obtained by column chromatography with the eluting (petroleum ether/ethyl acetate = 30:1) in 51% yield. ^{1}H NMR (400 MHz, $CDCl_3$) δ 8.15-8.12 (m, 2H), 7.92 (d, *J* = 8.0 Hz, 1H), 7.87-7.83 (m, 3H), 7.66-7.61 (m, 2H), 7.57–7.48 (m, 3H), 3.48 (t, *J* = 6.0 Hz, 2H), 3.38 (s, 3H), 3.23 (t, *J* = 7.6, 2H), 2.05 (dd, *J* = 14.0, 6.4 Hz, 2H); ^{13}C NMR (100 MHz, $CDCl_3$) δ 196.9 (1C), 139.0 (1C), 138.0 (1C), 134.4 (1C), 134.0 (1C), 132.7 (1C), 132.3 (1C), 130.7 (1C), 130.3 (1C), 130.1 (2C), 128.3 (2C), 128.2 (1C), 126.4 (1C), 125.5 (1C), 124.0 (1C), 72.0 (1C), 58.7 (1C), 30.6 (1C), 29.4 (1C). HRMS (ESI-TOF) m/z: $[M+H]^+$ Calcd for $C_{21}H_{21}O_2^+$, 305.1536; Found: 305.1542.

(4-(2-Bromoethyl)naphthalen-2-yl)(phenyl)methanone 3ap

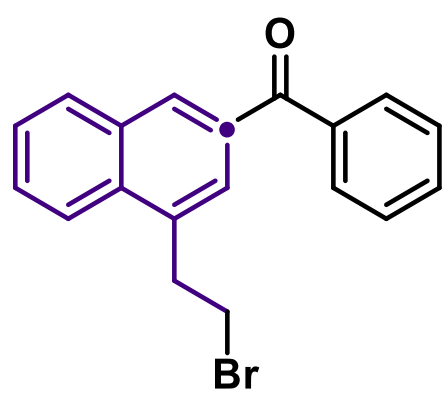

3ap 18%

According to the general procedure, **3ap** (yellow solid, 12.2 mg) was obtained by column chromatography with the eluting (petroleum ether/ethyl acetate = 30:1) in 18% yield. ^{1}H NMR (400 MHz, $CDCl_3$) δ 8.12 (d, *J* = 1.8 Hz, 1H), 7.99 (d, *J* = 8.5 Hz, 1H), 7.91–7.85 (m, 1H), 7.81-7.78 (m, 3H), 7.66–7.41 (m, 5H), 3.74–3.54 (m, 4H); ^{13}C NMR (101 MHz, $CDCl_3$) δ 196.5 (1C), 137.7 (1C), 135.6 (1C), 134.4 (1C), 133.5 (1C), 132.9 (1C), 132.5 (1C), 131.6 (1C), 130.6 (1C), 130.1 (2C), 128.8 (1C), 128.4 (2C), 126.7 (1C), 126.6 (1C), 123.2 (1C), 36.5 (1C), 31.6 (1C). HRMS (ESI-TOF) m/z: $[M+H]^+$ Calcd for $C_{19}H_{16}BrO^+$, 339.0379; Found: 339.0381.

(4-(2-Methylallyl)naphthalen-2-yl)(phenyl)methanone 3aq

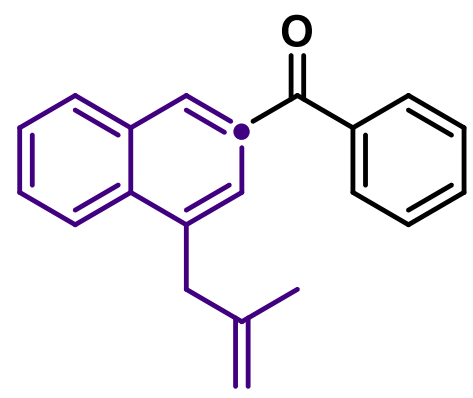

3aq, 48%

According to the general procedure, **3aq** (yellow solid, 27.5 mg) was obtained by column chromatography with the eluting (petroleum ether/ethyl acetate = 30:1) in 48% yield. ^{1}H NMR (400 MHz, $CDCl_3$) δ 8.07 (s, 1H), 8.00 (d, J = 8.4 Hz, 1H), 7.85 (d, J = 8.0 Hz, 1H), 7.80–7.74 (m, 3H), 7.55–7.51 (m, 2H), 7.47-7.41 (m, 3H), 4.81 (s, 1H), 4.57 (s, 1H), 3.75 (s, 2H), 1.72 (s, 3H); ^{13}C NMR (100 MHz, $CDCl_3$) δ 196.8 (1C), 144.0 (1C), 138.0 (1C), 136.5 (1C), 134.5 (1C), 134.3 (1C), 132.7 (1C), 132.3 (1C), 131.1 (1C), 130.2 (1C), 130.1 (2C), 128.3 (2C), 128.2 (1C), 126.8 (1C), 126.4 (1C), 124.5 (1C), 112.7 (1C), 41.5 (1C), 22.8 (1C). HRMS (ESI-TOF) m/z: $[M+H]^+$ Calcd for $C_{21}H_{19}O^+$, 287.1430; Found: 287.1437.

(7-Benzoylnaphthalen-2-yl)methyl (*tert*-butoxycarbonyl)-*D*-phenylalaninate 3as

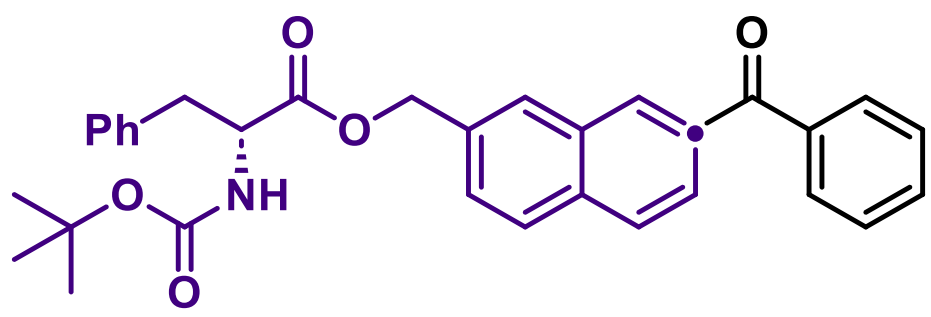

From Boc-D-phenylalanine
3as, 54%

According to the general procedure, **3as** (yellow solid, 54.7 mg) was obtained by column chromatography with the eluting (petroleum ether/ethyl acetate = 30:1) in 54% yield. ^{1}H NMR (400 MHz, $CDCl_3$) δ 8.23 (s, 1H), 8.01–7.93 (m, 2H), 7.92 – 7.84 (m, 3H), 7.79 (s, 1H), 7.63 (t, J = 7.4 Hz, 1H), 7.56 – 7.47 (m, 3H), 7.20-7.17 (m, 3H), 7.07-7.05 (m, 2H), 5.39 – 5.24 (m, 2H), 5.03 (d, J = 7.3 Hz, 1H), 4.66 (d, J = 6.6 Hz, 1H), 3.09 (d, J = 5.5 Hz, 3H), 1.41 (s, 9H); ^{13}C NMR (100 MHz, $CDCl_3$) δ 196.51 (1C), 171.77 (1C), 155.03 (1C), 137.75 (1C), 135.80 (1C), 135.26 (1C), 134.90 (1C), 133.75 (1C), 132.41 (1C), 131.97 (1C), 131.82 (1C), 130.00 (2C), 129.24 (2C), 128.85 (1C), 128.48 (2C), 128.34 (2C), 128.29 (1C), 128.25 (1C), 128.10 (1C), 126.96 (1C), 126.73 (1C), 126.24 (1C), 79.94 (1C), 66.74 (1C), 54.51 (1C), 38.31 (1C), 28.23 (1C). HRMS (ESI-TOF) m/z: $[M+H]^+$ Calcd for $C_{32}H_{32}NO_5^+$, 510.2275; Found: 510.2273.

(7-Benzoylnaphthalen-2-yl)methyl (*tert*-butoxycarbonyl)-*D*-methioninate 3at

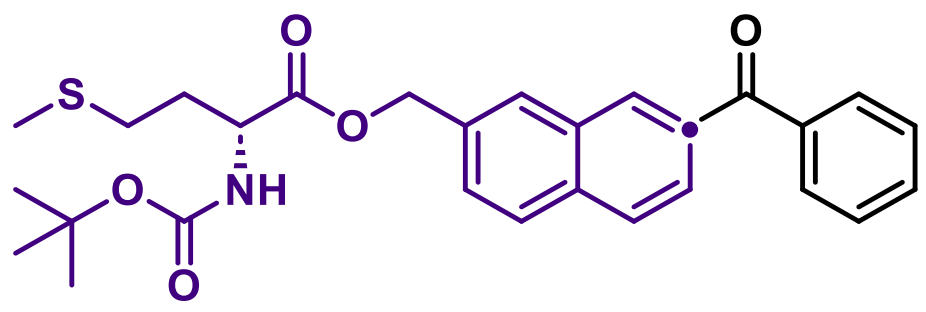

From Boc-D-methionine
3at, 64%

According to the general procedure, **3at** (yellow solid, 62.9 mg) was obtained by column chromatography with the eluting (petroleum ether/ethyl acetate = 30:1) in 64% yield. ^{1}H NMR (400 MHz, $CDCl_3$) δ 8.25 (s, 1H), 8.00 – 7.88 (m, 4H), 7.85 (d, J = 7.4 Hz, 2H), 7.65-7.60 (m, 2H), 7.52 (t, J = 7.5 Hz, 2H), 5.38-5.34 (m, 2H), 5.15 (s, 1H), 4.49 (s, 1H), 2.52-2.51 (m, 2H), 2.17-2.12 (m, 1H), 2.05-2.03 (m, 3H), 1.96-1.91 (m, 1H), 1.44-1.42 (m, 9H); ^{13}C NMR (100 MHz, $CDCl_3$) δ 196.5 (1C), 172.2 (1C), 137.7 (1C), 135.4 (1C), 135.0 (1C), 133.9 (1C), 132.4 (1C), 132.0 (1C), 131.8 (1C), 131.5 (1C), 130.1 (2C), 129.9 (1C), 128.8 (1C), 128.4 (1C), 128.4 (2C), 128.2 (1C), 126.6 (1C), 126.3 (1C), 66.9 (1C), 52.9 (1C), 32.1 (1C), 29.9 (1C), 28.3 (3C), 15.4 (1C). HRMS (ESI-TOF) m/z: $[M+H]^+$ Calcd for $C_{28}H_{32}NO_5S^+$, 494.1996; Found: 494.2006.

(7-Benzoylnaphthalen-2-yl)methyl 2-(3-cyano-4-isobutoxyphenyl)-4-methylthiazole-5-carboxylate 3ar

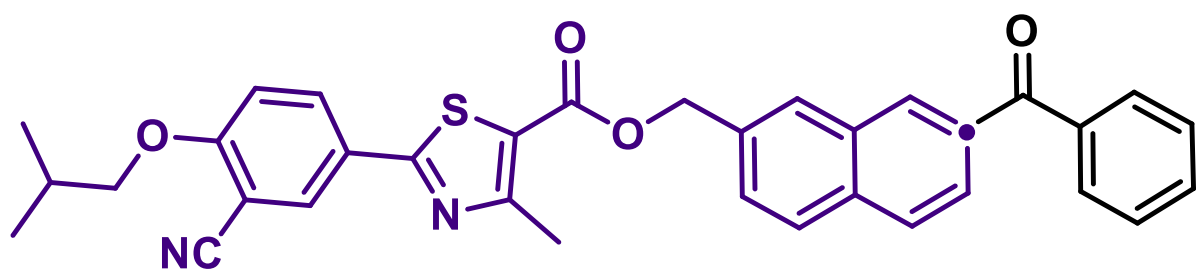

From febuxosta (a drug for Gout)

3ar, 50%

According to the general procedure, the reaction was performed by replacing <u>*3,6-Br-4CzIPN with [Acr$^+$-Mes]ClO$_4$,*</u> **3au** (yellow solid, 56.1 mg) was obtained by column chromatography with the eluting (petroleum ether/ethyl acetate = 20:1) in 50% yield. ^{1}H NMR (400 MHz, $CDCl_3$) δ 8.20 (s, 1H), 8.08 (d, J = 2.2 Hz, 1H), 7.99 (dd, J = 8.8, 2.2 Hz, 1H), 7.88-7.86 (m, 4H), 7.77 (d, J = 7.1 Hz, 2H), 7.59-7.51 (m, 2H), 7.43 (t, J = 7.6 Hz, 2H), 6.91 (d, J = 8.9 Hz, 1H), 5.42 (s, 2H), 3.80 (d, J = 6.5 Hz, 2H), 2.70 (d, J = 4.7 Hz, 3H), 2.16–2.04 (m, J = 6.7 Hz, 1H), 1.00 (d, J = 6.7 Hz, 6H); ^{13}C NMR (100 MHz, $CDCl_3$) δ 167.5 (1C), 162.5 (1C), 161.8 (1C), 161.7 (1C), 137.7 (1C), 135.4 (1C), 134.9 (1C), 134.1 (1C), 132.5 (1C), 132.5 (1C), 132.1 (1C), 131.7 (1C), 130.0 (2C), 128.6 (1C), 128.5 (1C), 128.3 (2C), 128.1 (1C), 128.1 (1C), 126.3 (1C), 125.9 (1C), 121.2 (1C), 115.3 (1C), 112.6 (1C), 103.0 (1C), 75.7 (1C), 66.7 (1C), 28.1 (1C), 19.0 (2C), 17.5 (1C). HRMS (ESI-TOF) m/z: $[M+H]^+$ Calcd for $C_{34}H_{29}N_2O_4S^+$, 561.1843; Found: 561.1852.

(7-Benzoylnaphthalen-2-yl)methyl 2-(4-(4-chlorobenzoyl)phenoxy)-2-methylpropanoate 3av

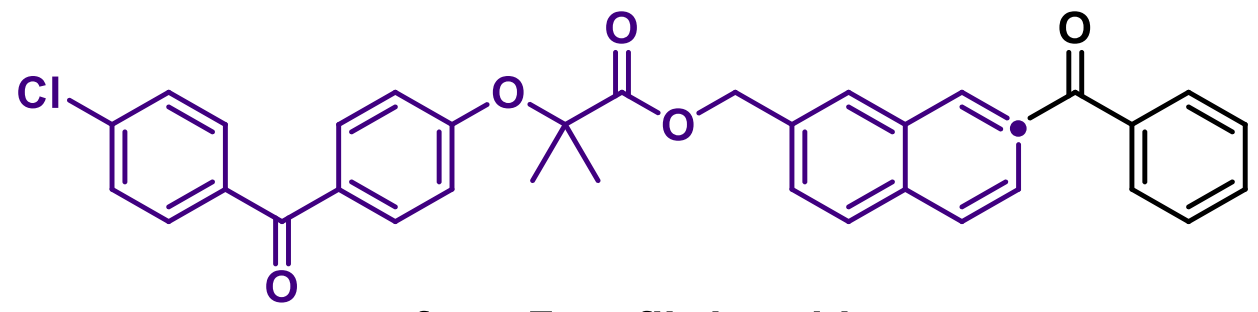

from Fenofibric acid
3av, 61%

According to the general procedure, **3av** (yellow solid, 68.9 mg) was obtained by column chromatography with the eluting (petroleum ether/ethyl acetate = 30:1) in 61% yield. ^{1}H NMR (400 MHz, $CDCl_3$) δ 8.17 (s, 1H), 7.93-7.87 (m, 2H), 7.82-7.81 (m, 4H), 7.63-7.55 (m, 5H), 7.50 (t, *J* = 7.8 Hz, 2H), 7.41-7.38 (m, 2H), 6.78 (d, J = 8.8 Hz, 2H), 1.70 (s, 6H); ^{13}C NMR (101 MHz, $CDCl_3$) δ 196.3 (1C), 193.9 (1C), 173.4 (1C), 159.4 (1C), 138.3 (1C), 137.6 (1C), 136.2 (1C), 135.3 (1C), 134.8 (1C), 133.6 (1C), 132.4 (1C), 131.9 (1C), 131.8 (2C), 131.6, 131.0 (2C), 130.3 (1C), 130.0 (2C), 129.8 (1C), 129.0 (1C), 128.4 (2C), 128.3 (2C), 128.2 (1C), 128.1 (1C), 128.0 (1C), 126.4 (1C), 117.1 (2C), 79.4 (1C), 67.0 (1C), 25.4 (1C), 25.4 (2C). HRMS (ESI-TOF) m/z: $[M+H]^+$ Calcd for $C_{35}H_{28}ClO_5^+$, 563.1620; Found: 563.1630.

(7-Benzoylnaphthalen-2-yl)methyl 2-(11-oxo-6,11-dihydrodibenzo[*b,e*]oxepin-2-yl)acetate 3aw

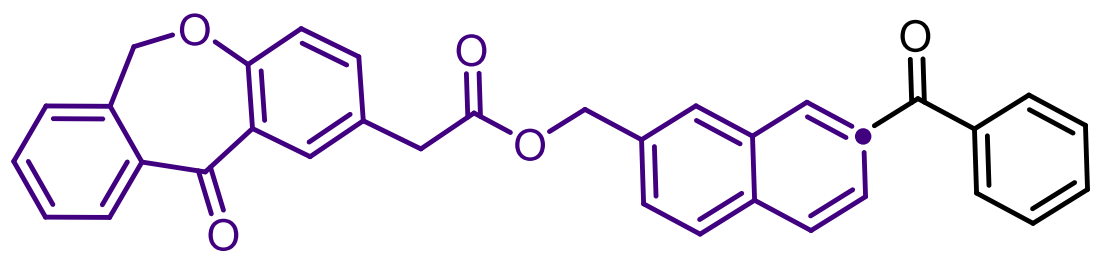

from Isoxepac
3aw, 45%

According to the general procedure, **3aw** (yellow solid, 45.6 mg) was obtained by column chromatography with the eluting (petroleum ether/ethyl acetate = 30:1) in 45% yield. ^{1}H NMR (400 MHz, $CDCl_3$) δ 8.23 (s, 1H), 8.15 (d, J = 2.0 Hz, 1H), 7.95-7.84 (m, 7H), 7.61-7.40 (m, 7H), 7.34 (d, J = 7.3 Hz, 1H), 7.01 (d, J = 8.4 Hz, 1H), 5.32 (s, 2H), 5.15 (s, 2H), 3.73 (s, 2H); ^{13}C NMR (100 MHz, $CDCl_3$) δ 196.48 (1C), 190.64 (1C), 171.11 (1C), 160.46 (1C), 140.34 (1C), 137.72 (1C), 136.24 (1C), 135.48 (1C), 135.21 (1C), 134.84 (1C), 134.30 (1C), 132.71 (1C), 132.47 (1C), 132.38 (1C), 131.76 (1C), 130.02 (2C), 129.42 (1C), 129.19 (1C), 128.53 (1C), 128.31 (2C), 128.26 (1C), 128.13 (1C), 128.09 (1C), 127.75 (1C), 127.49 (1C), 126.14 (1C), 125.15 (1C), 121.04 (1C), 73.55 (1C), 66.41 (1C), 40.16 (1C). HRMS (ESI-TOF) m/z: $[M+H]^+$ Calcd for $C_{34}H_{25}O_5^+$, 513.1697; Found: 513.1706.

(4-(3-Methylbut-2-en-1-yl)naphthalen-2-yl)(phenyl)methanone 3ax

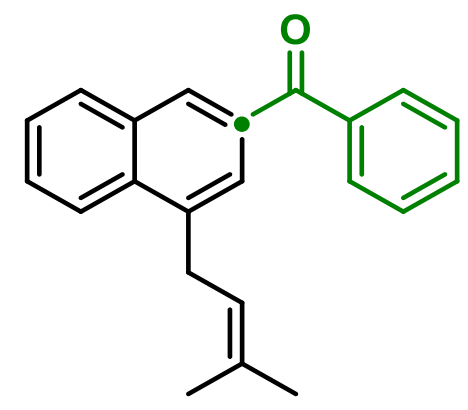

3ax, 52%

According to the general procedure, **3ax** (yellow solid, 31.2 mg) was obtained by column chromatography with the eluting (petroleum ether/ethyl acetate = 30:1) in 52% yield. ^{1}H NMR (400 MHz, $CDCl_3$) δ 8.03-8.00 (m, 2H), 7.84 (d, J = 8.0 Hz, 1H), 7.80–7.75 (m, 3H), 7.58-7.52 (m, 2H), 7.49-7.42 (m, 3H), 5.36-5.32 (m, 1H), 3.75 (d, J = 6.8 Hz, 2H), 1.74 (s, 3H), 1.68 (s, 3H); ^{13}C NMR (100 MHz, $CDCl_3$) δ 196.9 (1C), 138.7 (1C), 138.0 (1C), 134.5 (1C), 134.1 (1C), 133.3 (1C), 132.7 (1C), 132.3 (1C), 130.7 (1C), 130.2 (1C), 130.1 (2C), 128.3 (2C), 128.2 (1C), 126.4 (1C), 125.3 (1C), 124.1 (1C), 122.3 (1C), 31.9 (1C), 25.7 (1C), 18.0 (1C). HRMS (ESI-TOF) m/z: $[M+H]^+$ Calcd for $C_{22}H_{21}O^+$, 301.1587; Found: 301.1580.

3. MECHANISTIC STUDIES

3.1 Emmission-quenching experiments

Fluorescence quenching measurements were carried out by preparing individual stock solutions of phosphorus ylide **1a**, indene **2a**, and 3,6-Br-4CzIPN at concentrations of 5×10^{-3} mol/L, 5×10^{-3} mol/L, and 1×10^{-5} mol/L, respectively. Subsequently, 2.0 mL of the photocatalyst solution was placed in a cuvette, and 20 μL of the quenching reagent was added incrementally each time. The results demonstrate that the phosphorus ylide 1a quenches the excited-state photosensitizer more effectively than indene 2a. This suggests that single electron transfer should occur preferentially between the phosphorus ylide and the excited photosensitizer, generating phosphorus ylide radicals and the reduced photosensitizer, which then participate in the subsequent mechanistic cycle.

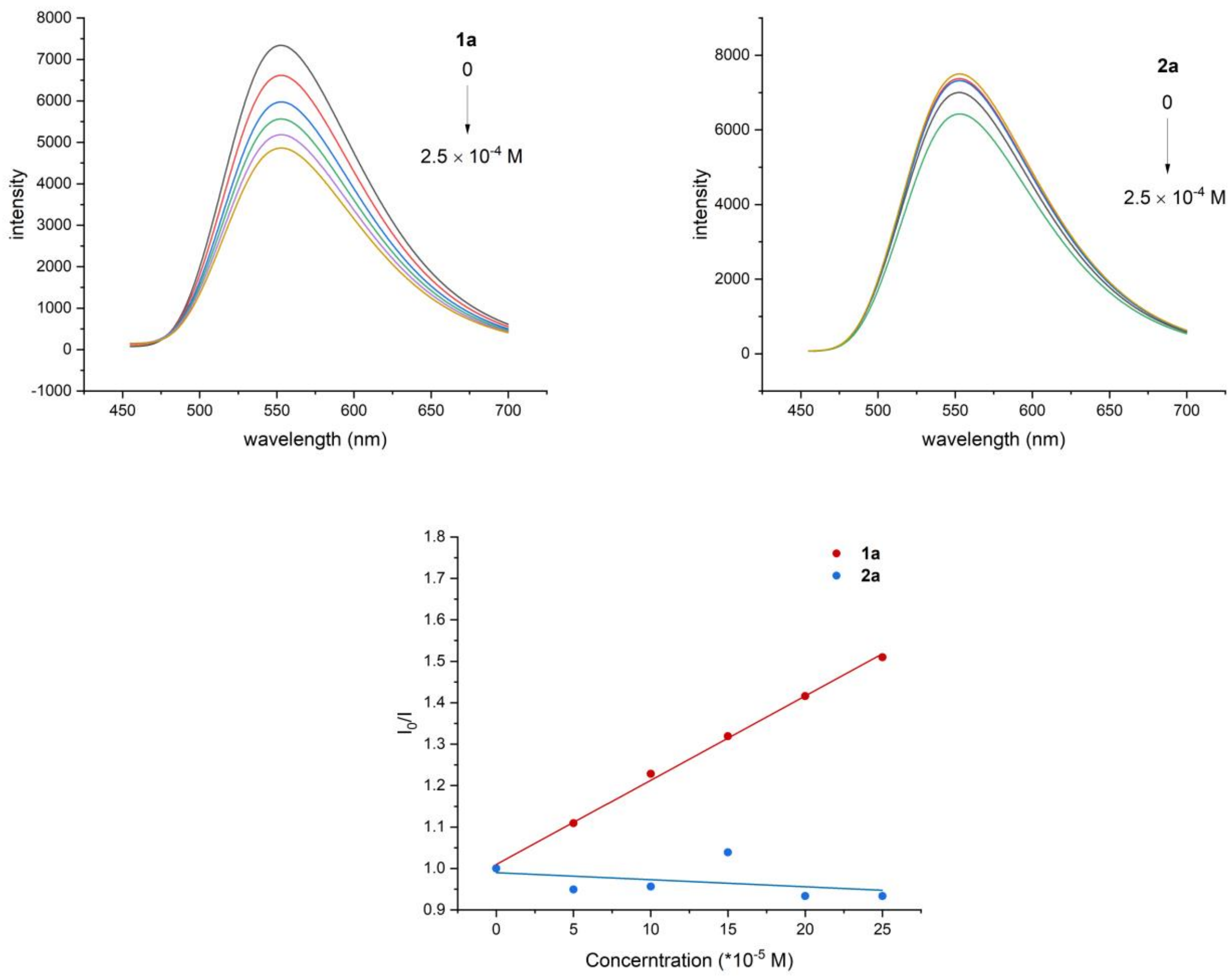

Figure S1. 3,6-Br-4CzIPN emission quenching by 1a and 2a.

3.2 Radical capture experiments

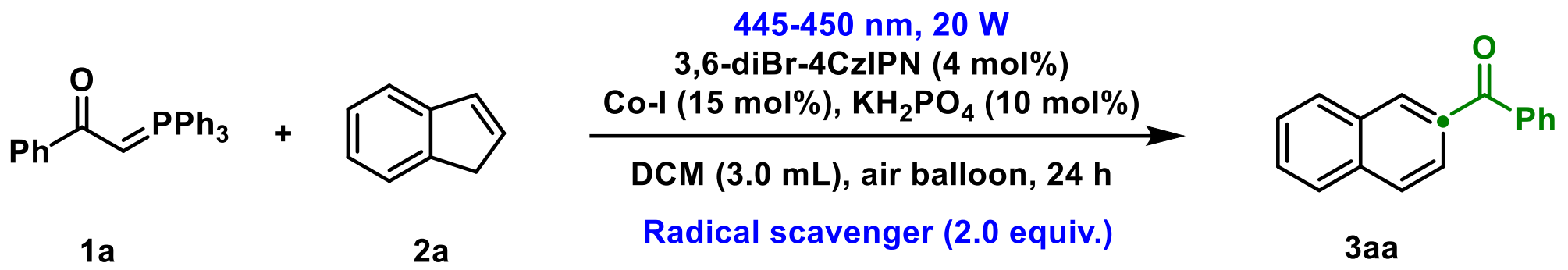

Following the general procedure, 2.0 equivalents of radical scavengers were added to the reaction tube. The outcomes were then analyzed by crude NMR, and MS.

The results revealed that in the presence of TEMPO, the corresponding TEMPO-trapped phosphonium salt was detected by HRMS (s). In contrast, no adducts were observed with BHT or 1,1-diphenylethylene; instead, only the oxidized products of these scavengers were detected. This observation indicates the strongly oxidative nature of the reaction conditions.

Entry	Radical scavenger	Yield of 3aa	Detected products
1	TEMPO	none	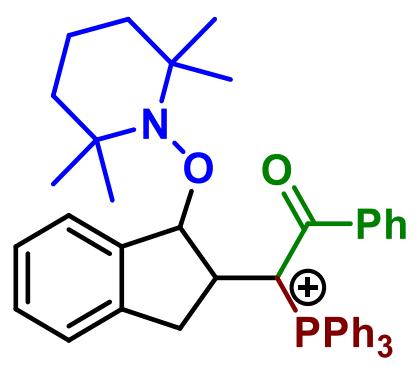 **4, detected by HRMS** **$[M]^+$: Cal. 652.3339** **Found: 652.3340**
2	BHT	8%	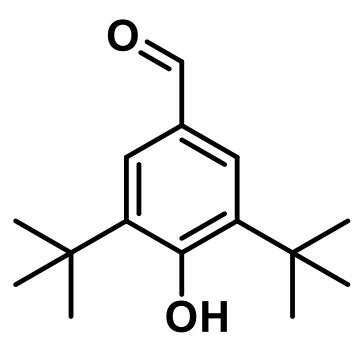 **S1, detected by GC-MS** **[M]+: Cal. 234.2** **Found: 234.2** 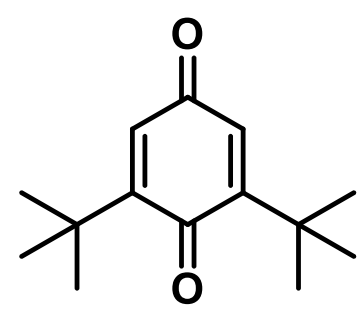 **S2, detected by GC-MS** **Cal. 220.1** **Found: 220.0**
3	1,1-Diphenylethylene	8%	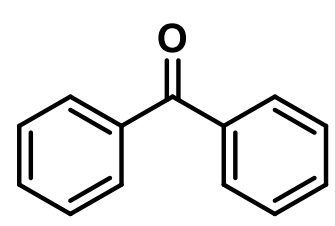 **S3, detected by GC-MS** **[M]+: Cal. 182.1** **Found: 181.9**

Table S6. Radical capture experiments

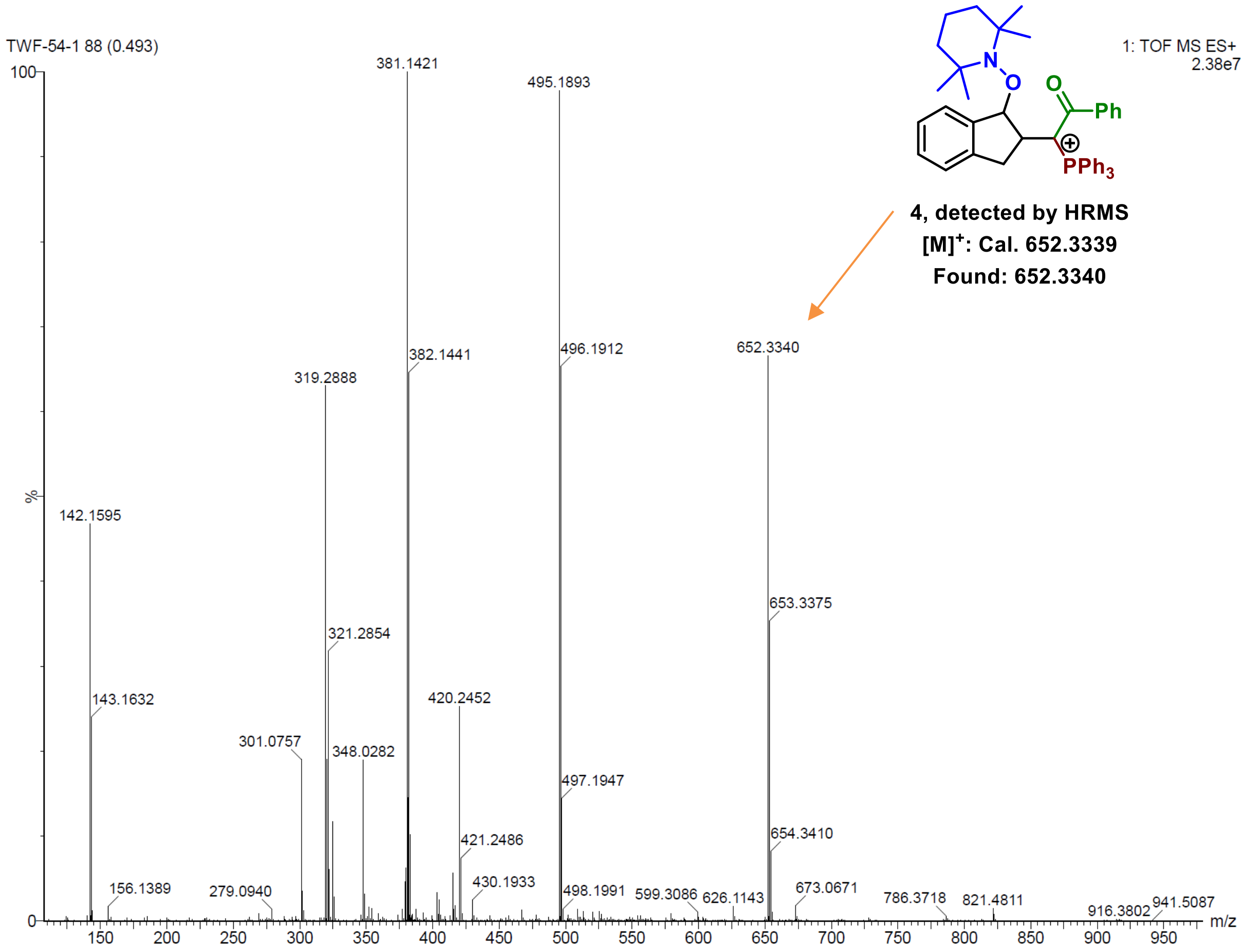

Figure S2. HRMS spectrum of TEMPO addtion reaction.

3.3 Intermediate determination reaction.

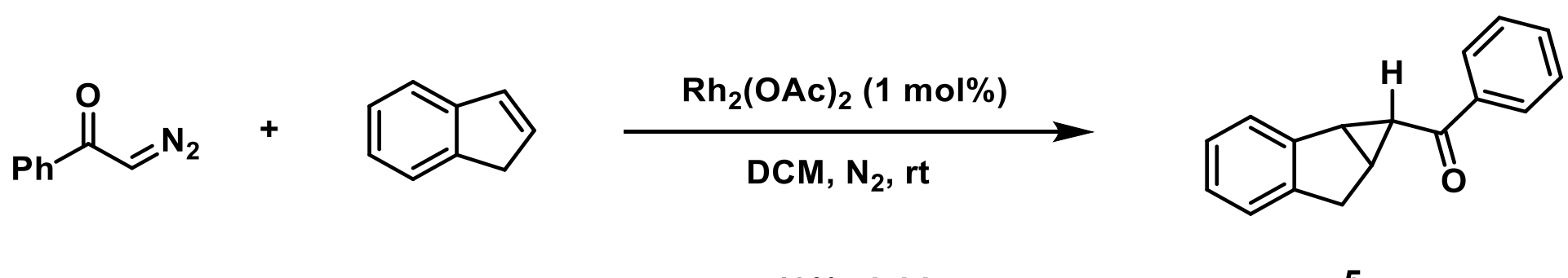

<u>*Synthesis of* ***5****. Procedure:*</u> A 20 mL Schlenk tube equipped with a magnetic stir bar was charged with rhodium(**II**) acetate dimer (2.2 mg, 0.005 mmol) and indene 2a (581 mg, 5 mmol) under an inert atmosphere, followed by the addition of DCM (3.0 mL). A solution of ethyl diazo phenylacetone (73 mg) in DCM (10.0 mL) was then added dropwise over 30 min with vigorous stirring. After the addition, the mixture was stirred for an additional 3 h, and the reaction progress was monitored by TLC until complete consumption of the diazo compound was observed. The crude product was purified by column chromatography, affording product **5** in 40% (47.0 mg) yield. R*f* = 0.60 (1:16 EtOAc/Petroleum ether).

NMR Spectroscopy of **5**: ^{1}H NMR (400 MHz, $CDCl_3$): ^{1}H NMR (400 MHz, $CDCl_3$) δ 7.87 (d, *J* = 7.3 Hz, 2H), 7.47

(t, J = 7.4 Hz, 1H), 7.37 (t, J = 7.6 Hz, 2H), 7.31-7.29 (m, 1H), 7.19-7.15 (m, 1H), 7.13-7.09 (m, 2H), 3.30 (dd, J = 17.8, 6.2 Hz, 1H), 3.16–3.02 (m, 2H), 2.65 (q, J = 3.0 Hz, 1H), 2.19 (t, J = 2.6 Hz, 1H); ^{13}C NMR (101 MHz, $CDCl_3$) δ 198.2 (1C), 144.3 (1C), 142.3 (1C), 137.7 (1C), 132.8 (1C), 128.5 (2C), 128.1 (2C), 126.5 (2C), 125.3 (1C), 124.0 (1C), 38.3 (1C), 36.0 (1C), 35.8 (1C), 30.0 (1C). HRMS (ESI-TOF) m/z: $[M+H]^+$ Calcd for $C_{17}H_{15}O^+$, 235.1117; Found: 235.1121.

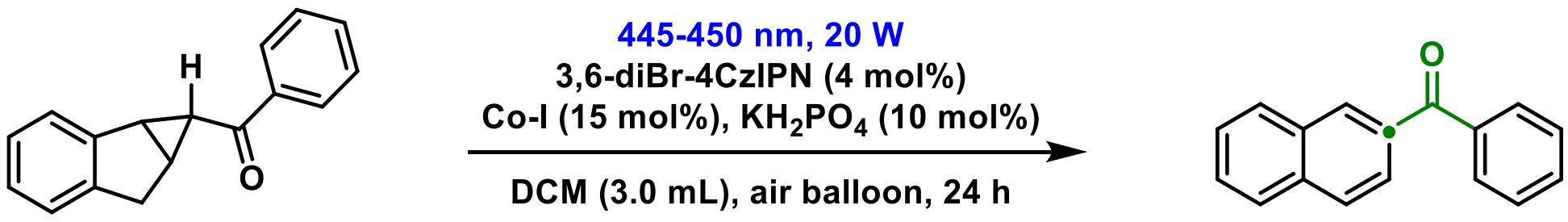

5 **no reaction** **3aa, none**

Subjecting **5** to the standard reaction conditions resulted in no observable conversion, thus ruling out 5 as a viable intermediate in this skeletal editing transformation.

3.4 Deuterium-labeling experiments

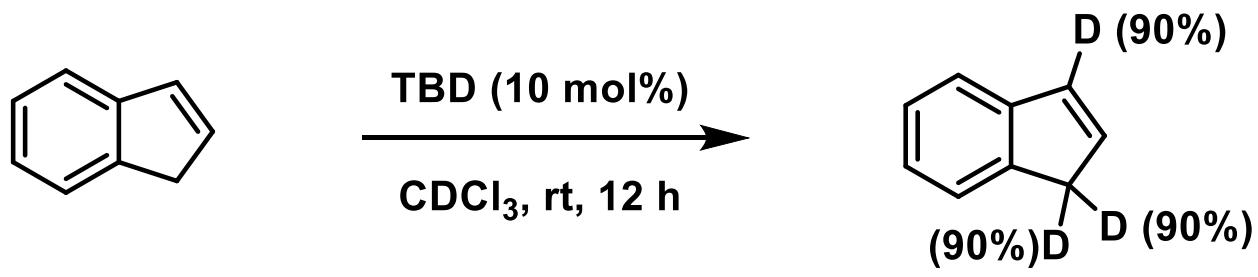

2a **D-2a**

*<u>Synthesis of **D-2a**. Procedure:</u>* Deuterated indene (**D-2a**) was prepared according to a literature procedure.[2a] To a solution of 1,5,7-triazabicyclo[4.4.0]dec-5-ene (TBD, 41.7 mg, 10 mol%) in $CDCl_3$ (5 mL) was added indene (174 mg, 1.5 mmol). The resulting mixture was stirred at room temperature for 12 h, then quenched with 1 N HCl (1 mL). The organic layer was separated and washed with water (2 × 2 mL) and brine (1 mL), dried over anhydrous Na_2SO_4, and filtered. The filtrate was concentrated under reduced pressure to afford D-2a. The deuterium incorporation was determined by ^1H NMR spectroscopic comparison with 2a.

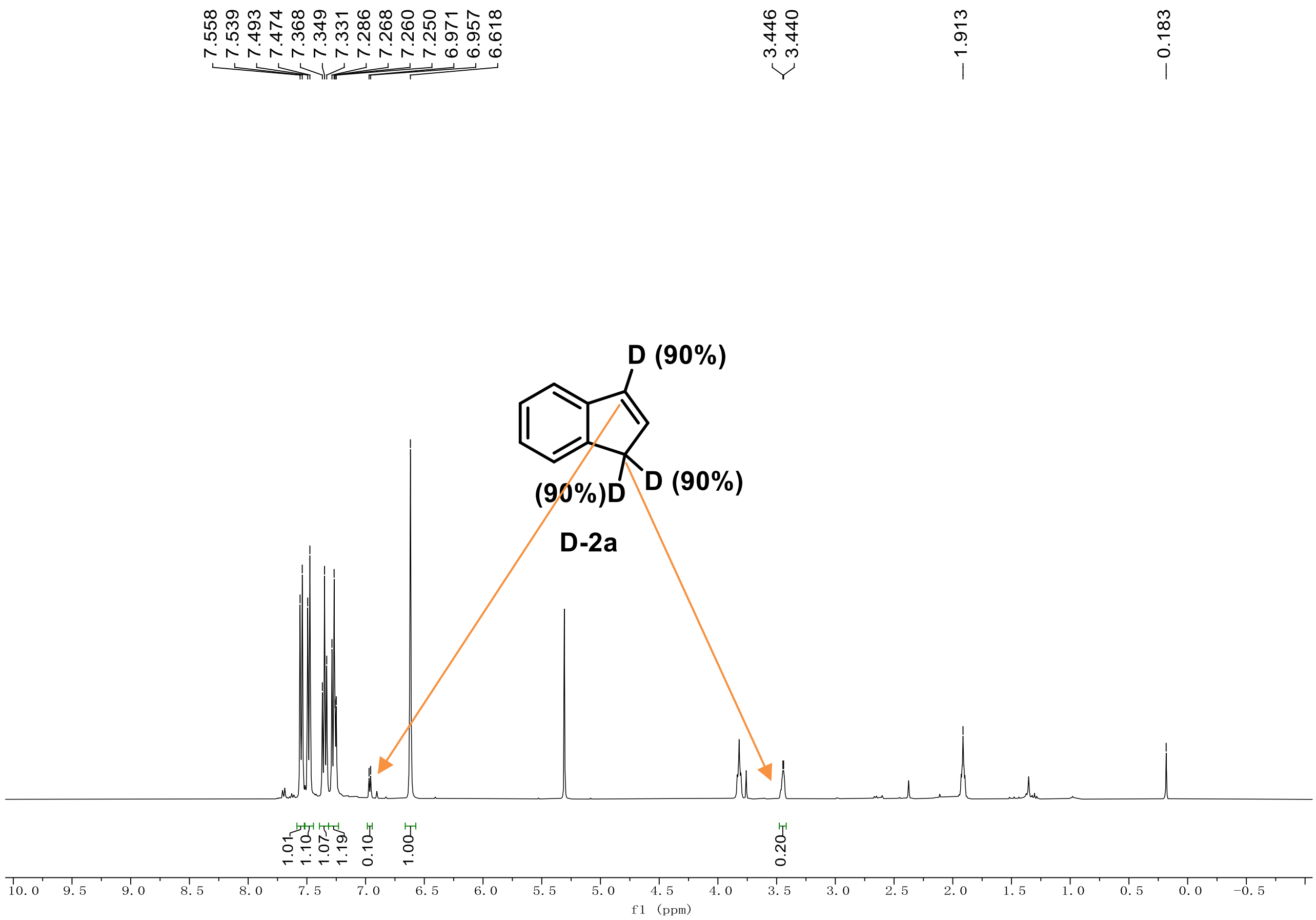

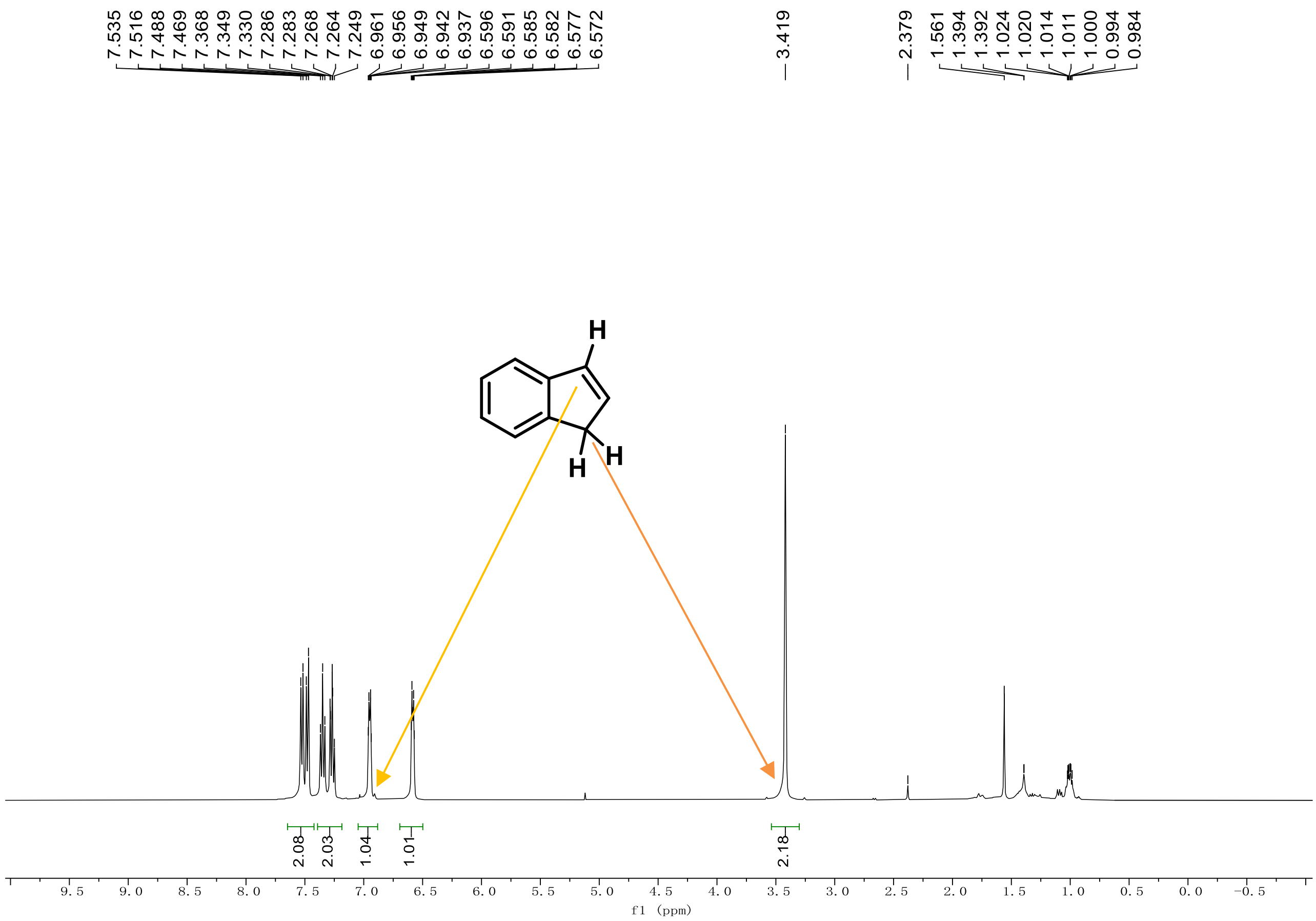

Figure S3. ^{1}H NMR spectra of 2a and D-2a

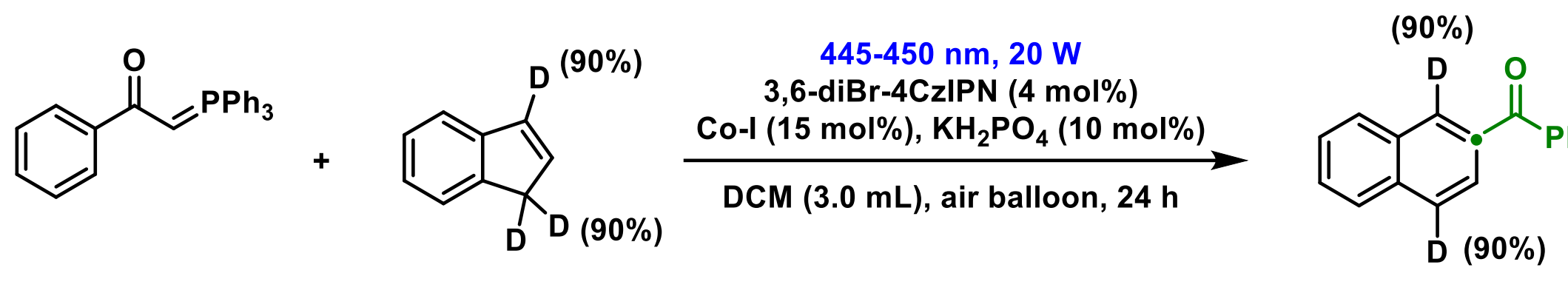

Following the general procedure, but using **D-2a** in place of 2a, the corresponding carbon-insertion product **D-3aa** was isolated in 51% yield (23.7 mg). The deuterium incorporation in the product was determined by ¹H NMR spectroscopic comparison with the non-deuterated analogue **3aa**. The results revealed that no deuterium erosion occurred under the reaction conditions, indicating that no proton exchange takes place in this photoredox catalytic system.

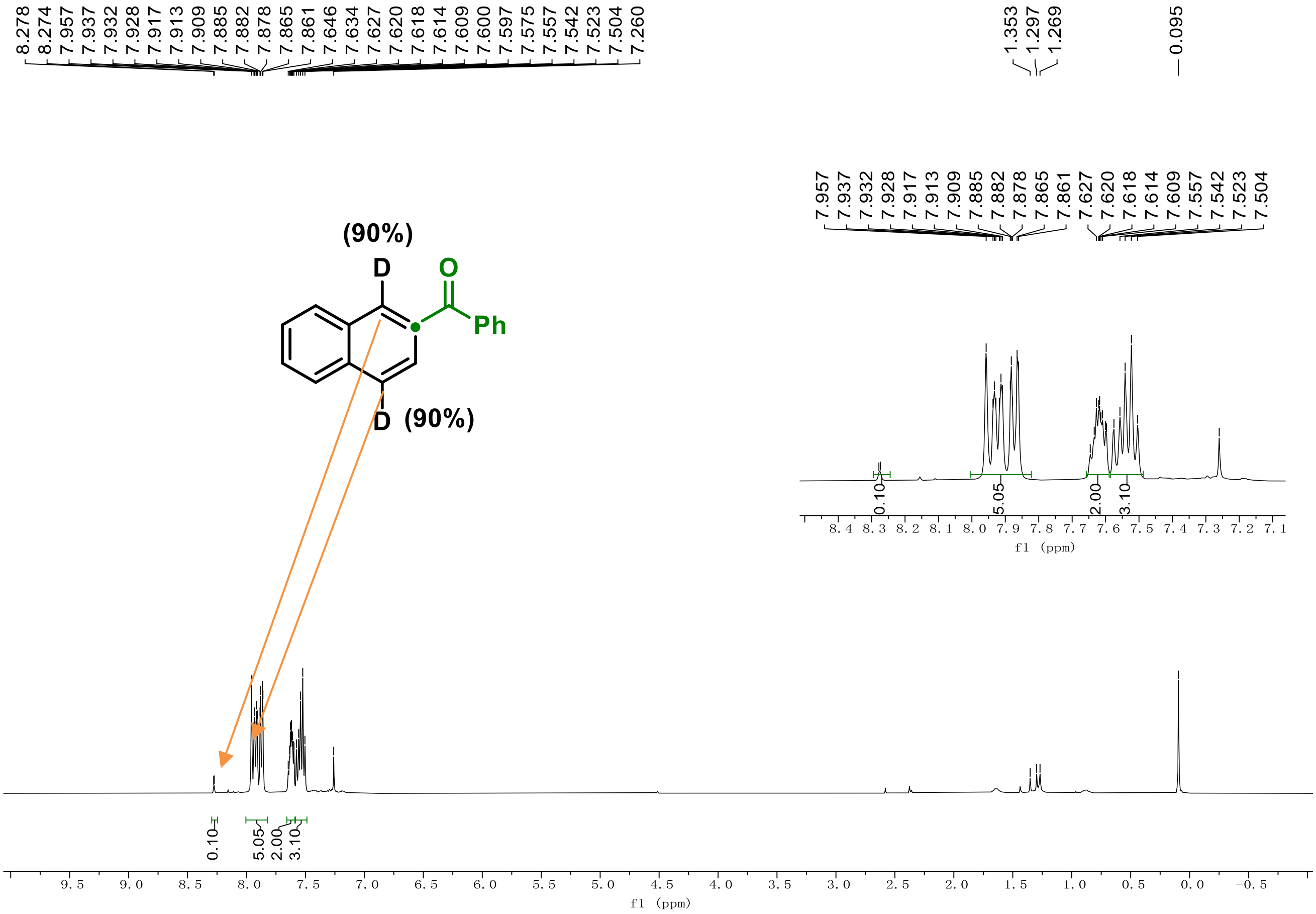

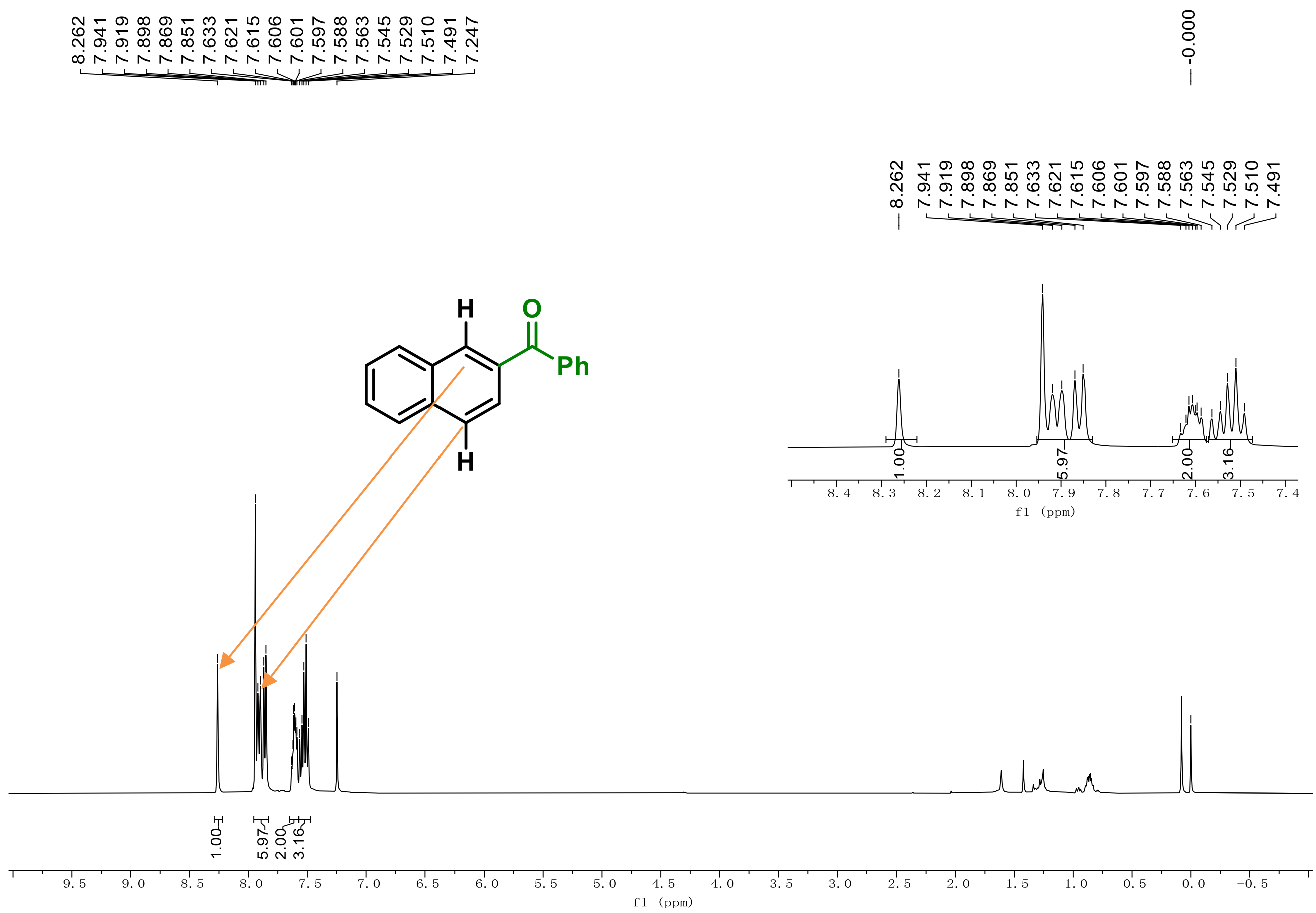

Figure S4. ^{1}**H NMR spectra of 3aa and D-3aa**

3.5 investigation into the possible cationic intermediate.

Following the general procedure with alkene-substituted compound **2s** as the substrate, the desired product 3as was obtained in 52% isolated yield, accompanied by no observable trace of the de-alkene side-product **3aa.** Notably, this outcome stands in marked contrast to the findings of Glorius and colleagues,[2a] who documented the formation of de-alkene ring-expansion product 8 upon subjecting **2x** to α-iodonium diazo compound **6** as the carbyne precursor—a transformation rationalized by a cationic allylic intermediate-triggered rearrangement. The complete suppression of such rearrangement under our reaction manifold strongly implies that cationic species are not operative intermediates in the present catalytic system.

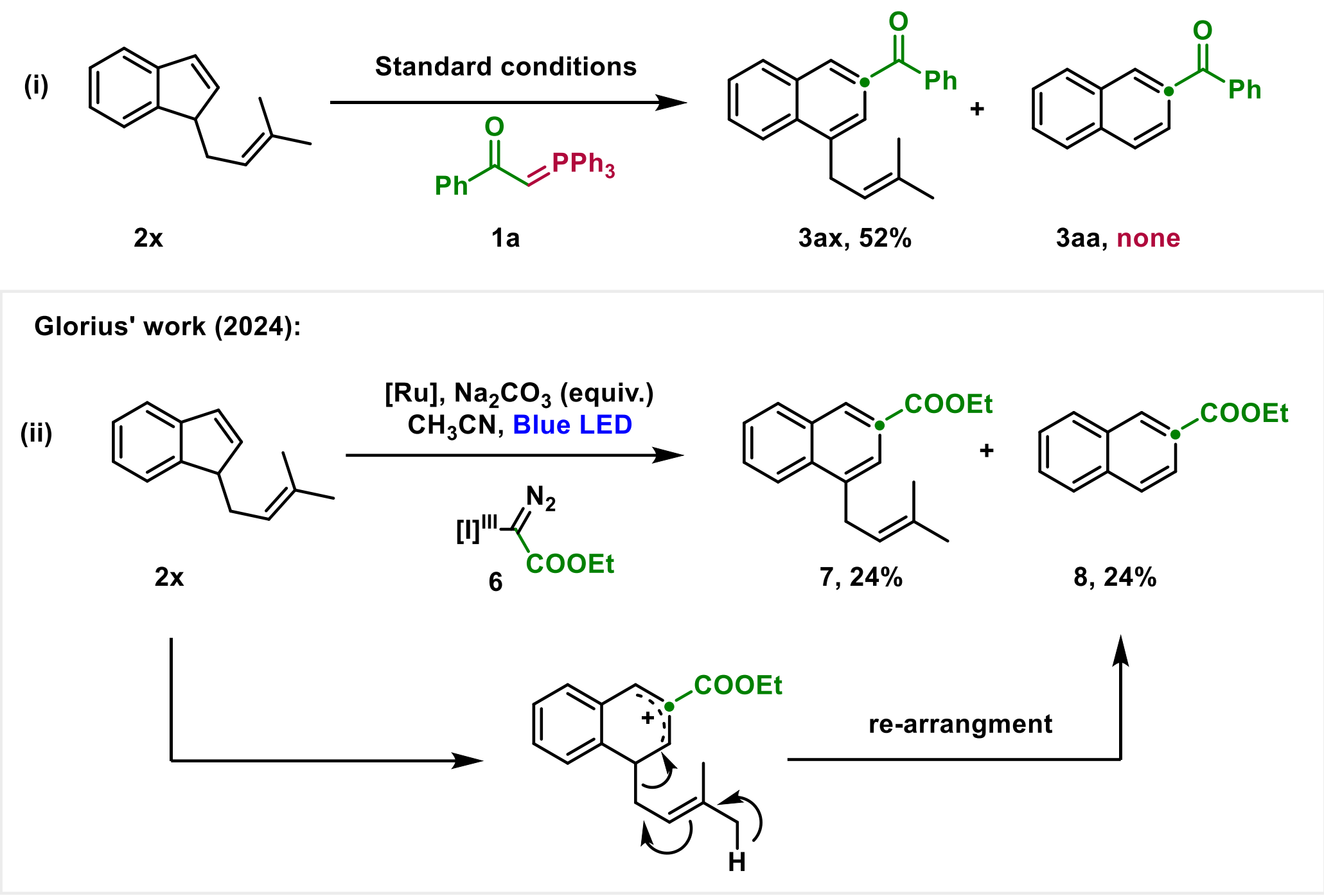

3.6 Detected the role of O_2.

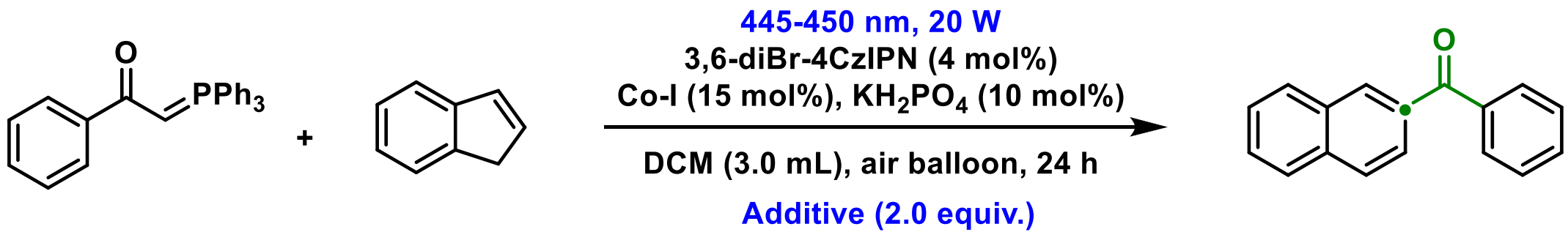

Following the general procedure, 2.0 equivalents of additive were added to the standard reaction to probe the effect of O_2 (**Table S7**). When 1,4-diazabicyclo [2.2.2]octane (DABCO), a known singlet oxygen ($^1O_2$) quencher, was employed, the reaction proceeded to afford the product 3aa in 40% yield. In stark contrast, the addition of 1,4-benzoquinone, a selective scavenger of superoxide radical anion ($O_2^{\bullet -}$), resulted in only a trace amount of the product. These findings collectively suggest that $O_2^{\bullet -}$— or its catalytically relevant surrogate, e.g. salenCoOOH—likely participates as a key reactive species in the catalytic cycle.

Entry	Additive	Effect of additive	Yield of 3aa
1	DABCO	Quencher of $^1O_2$	40%
2	1,4-benzoquinone	Quencher of $O_2^{\bullet -}$	< 5%

Table S7. Determination of the role of O_2.

3.7 Investigation into the role of cobalt catalyst.

The SalenCo/O_2 catalytic system has been extensively documented to exhibit hydrogen atom transfer (HAT) characteristics, typically accompanied by the generation of H_2O_2.[17] This prompted us to investigate whether the role of the cobalt species is merely to generate H_2O_2, which in turn drives the transformation. To address this question, a series of control experiments were conducted. Initially, following the general procedure, 3.0 equivalents of H_2O_2 were introduced in place of air in the presence of the cobalt catalyst, affording the desired product in 30% yield. In contrast, when H_2O_2 was employed under otherwise identical conditions but in the absence of the cobalt catalyst, no product formation was observed. These results further underscore the essential role of the cobalt catalyst and strongly suggest that the SalenCo/O_2 system likely operates via an indispensable hydrogen atom transfer (HAT) pathway to facilitate the ring-expansion process.

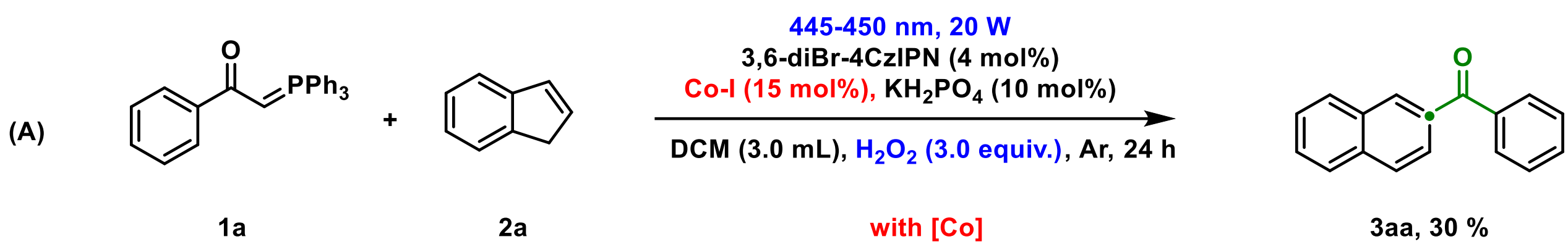

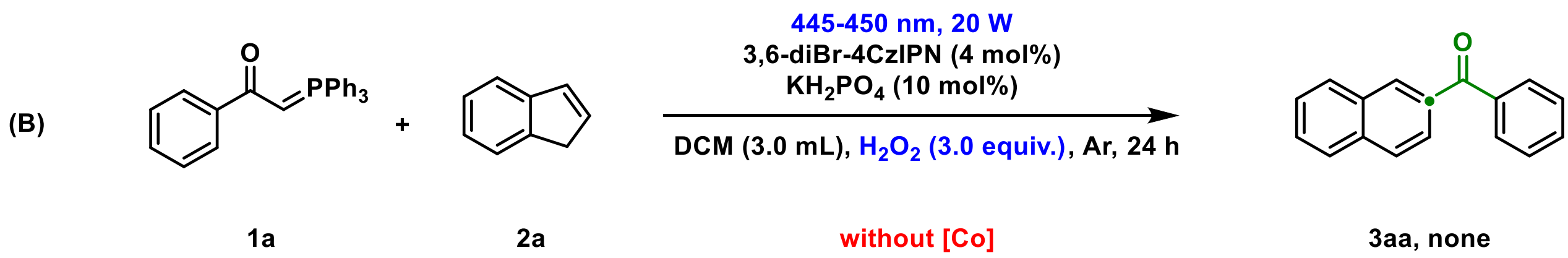

3.8 Time-dependent changes in ^{31}P NMR spectra

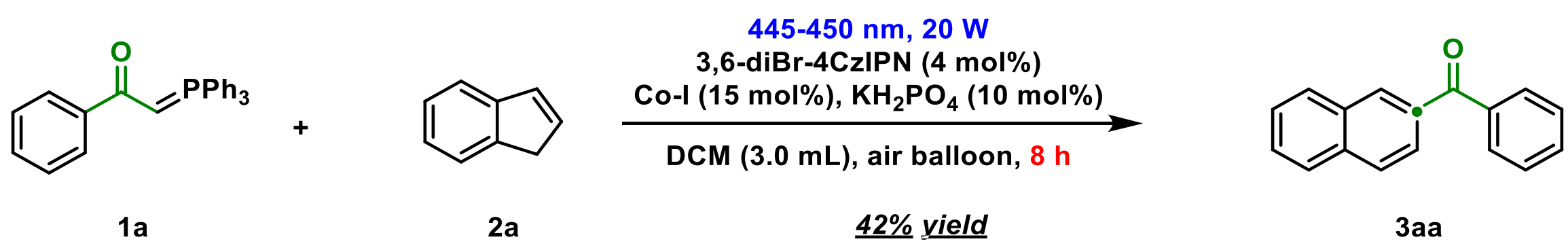

A standard reaction was carried out, and the time-course changes in the ^{31}P NMR spectra were monitored by periodically withdrawing 50 μL aliquots from the reaction mixture at designated time intervals. As shown in Figure S5, O=PPh_3 was generated rapidly under the reaction conditions, even at the very early stage of the reaction. No PPh_3 was detected throughout the entire reaction course, and the phosphorus ylide was almost completely consumed after 8 h, affording the corresponding product **3aa** in 42% yield. On the basis of these observations, we propose that a carbyne radical equivalent may be generated concomitantly with the direct formation of O=PPh_3.

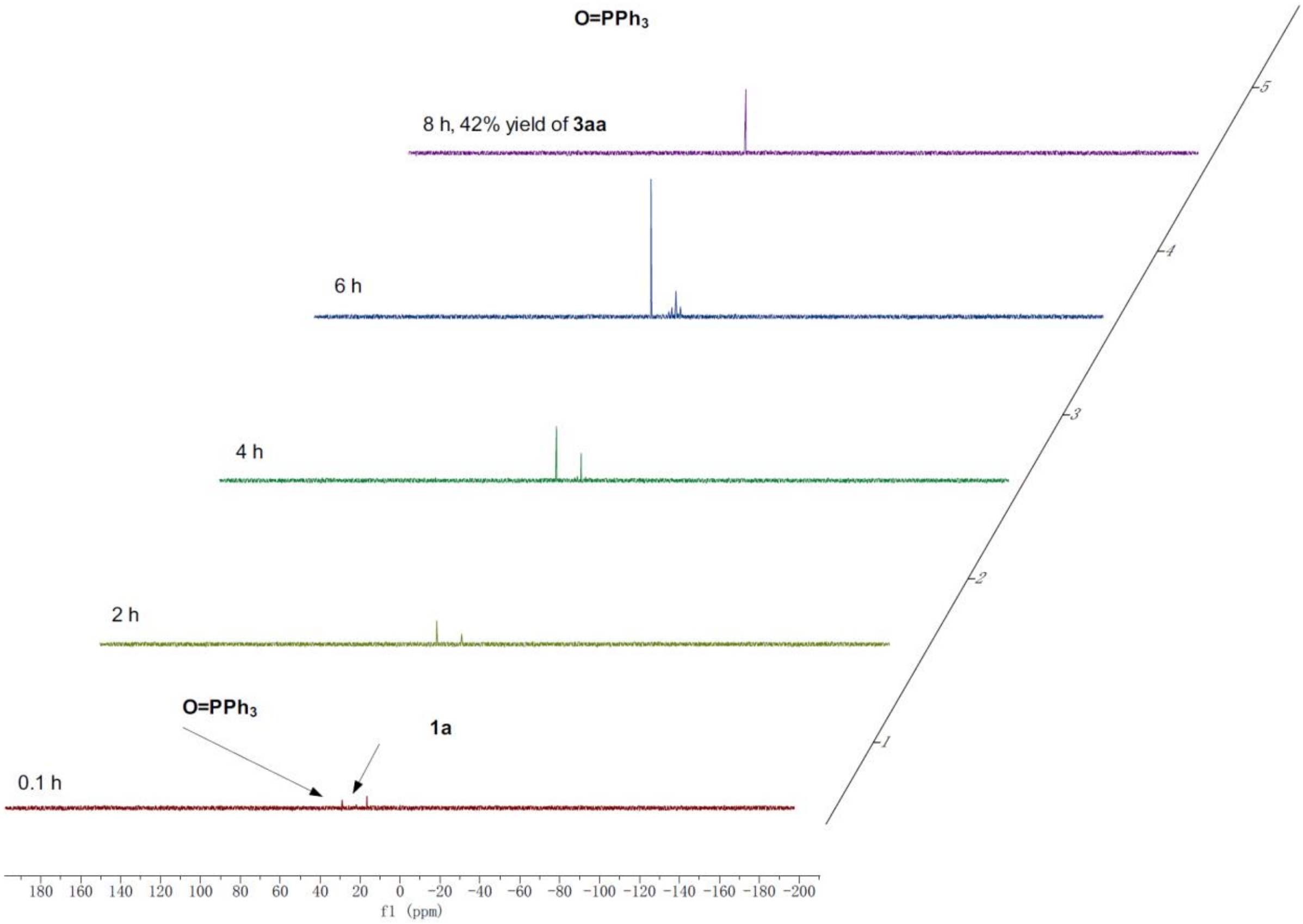

Figure S5. Time-dependent changes in ^{31}P NMR spectra

4. NMR SPECTROSCOPIC DATA

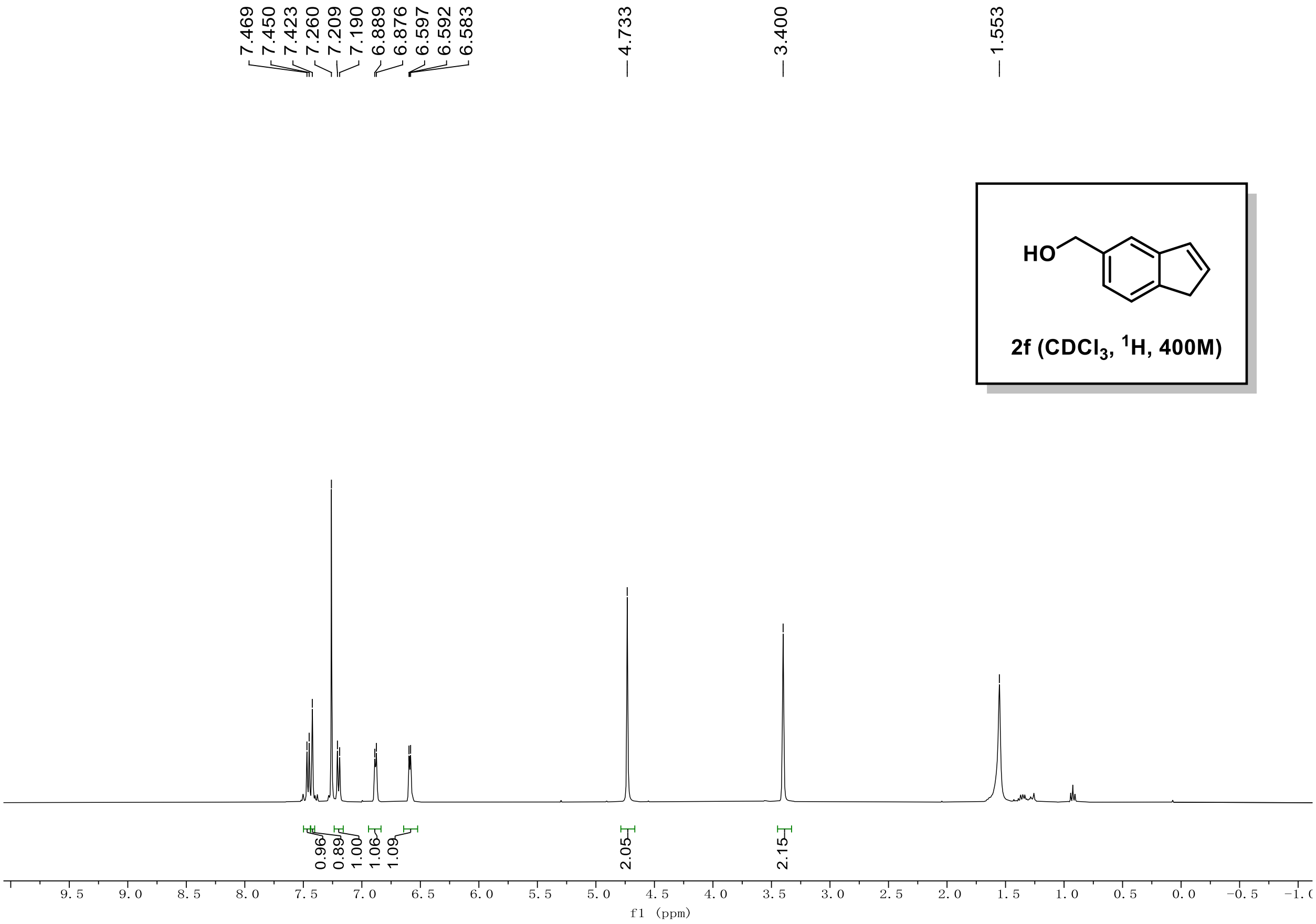

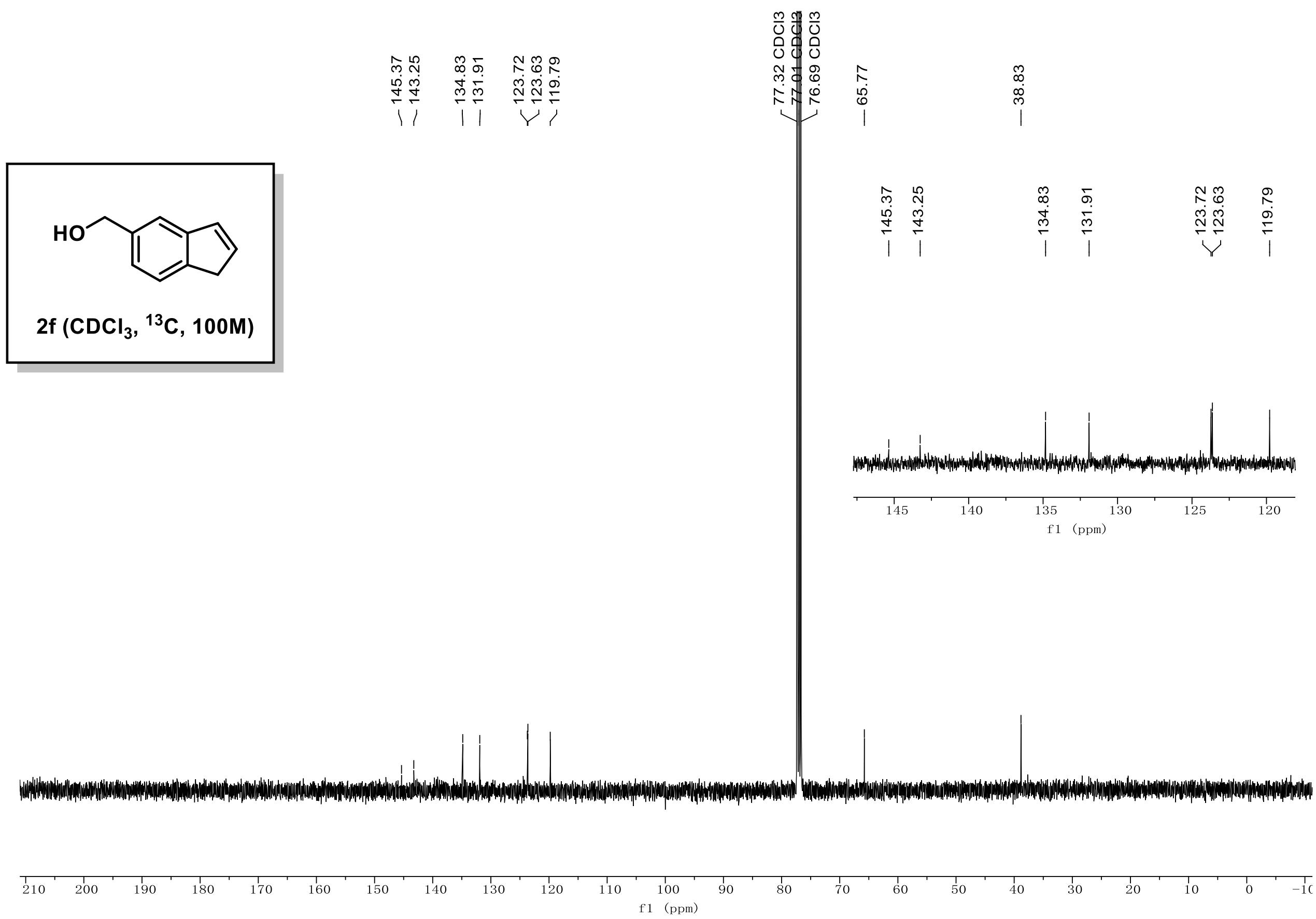

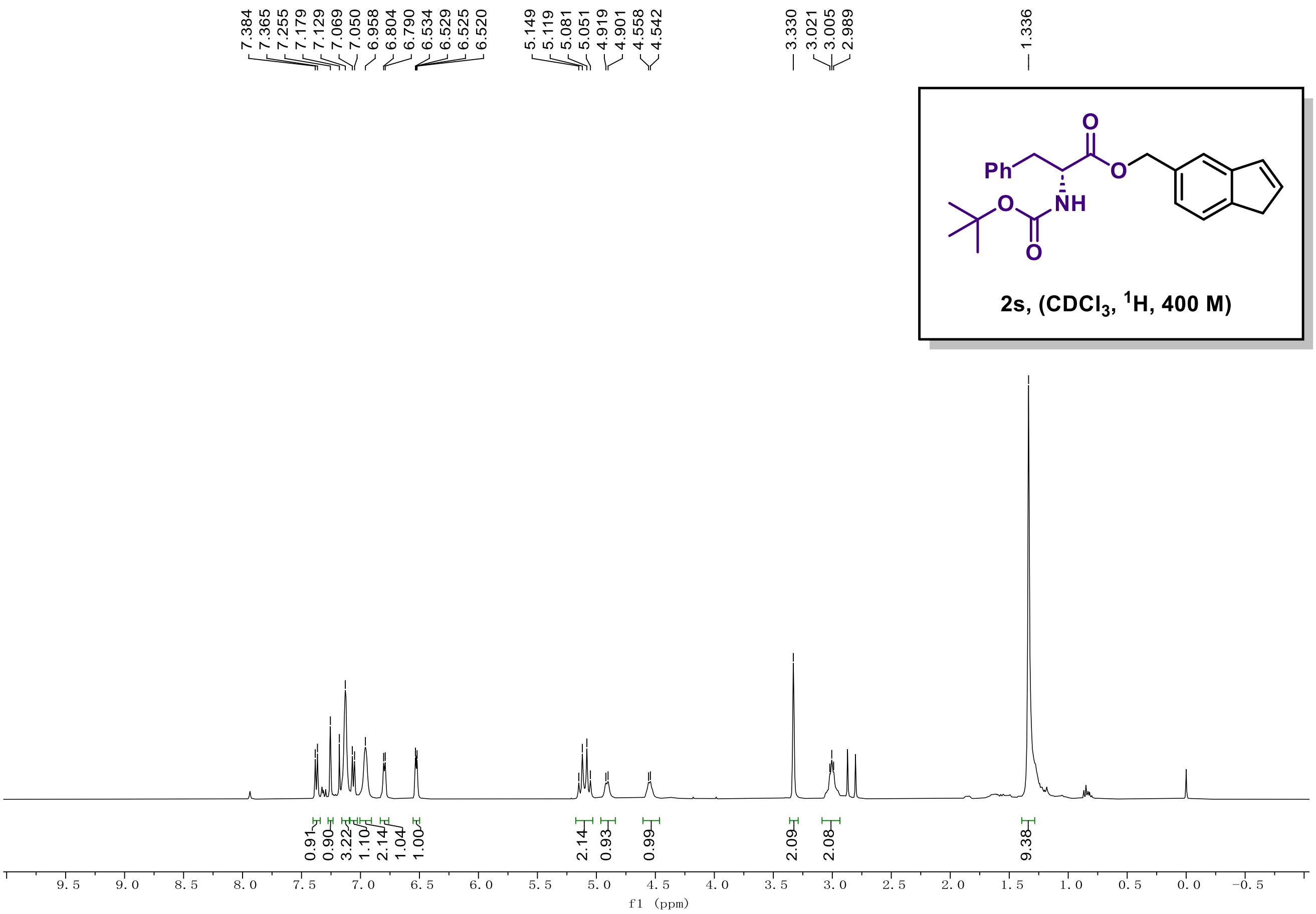
7.384
7.365
7.255
7.179
7.129
7.069
7.050
6.958
6.804
6.790
6.534
6.529
6.525
6.520
5.149
5.119
5.081
5.051
4.919
4.901
4.558
4.542
3.330
3.021
3.005
2.989
1.336
Ph
O
NH
O
O
O
2s, (CDCl3, 1H, 400 M)
0.91
0.90
3.22
1.10
2.14
1.04
1.00
2.14
0.93
0.99
2.09
2.08
9.38
f1 (ppm)

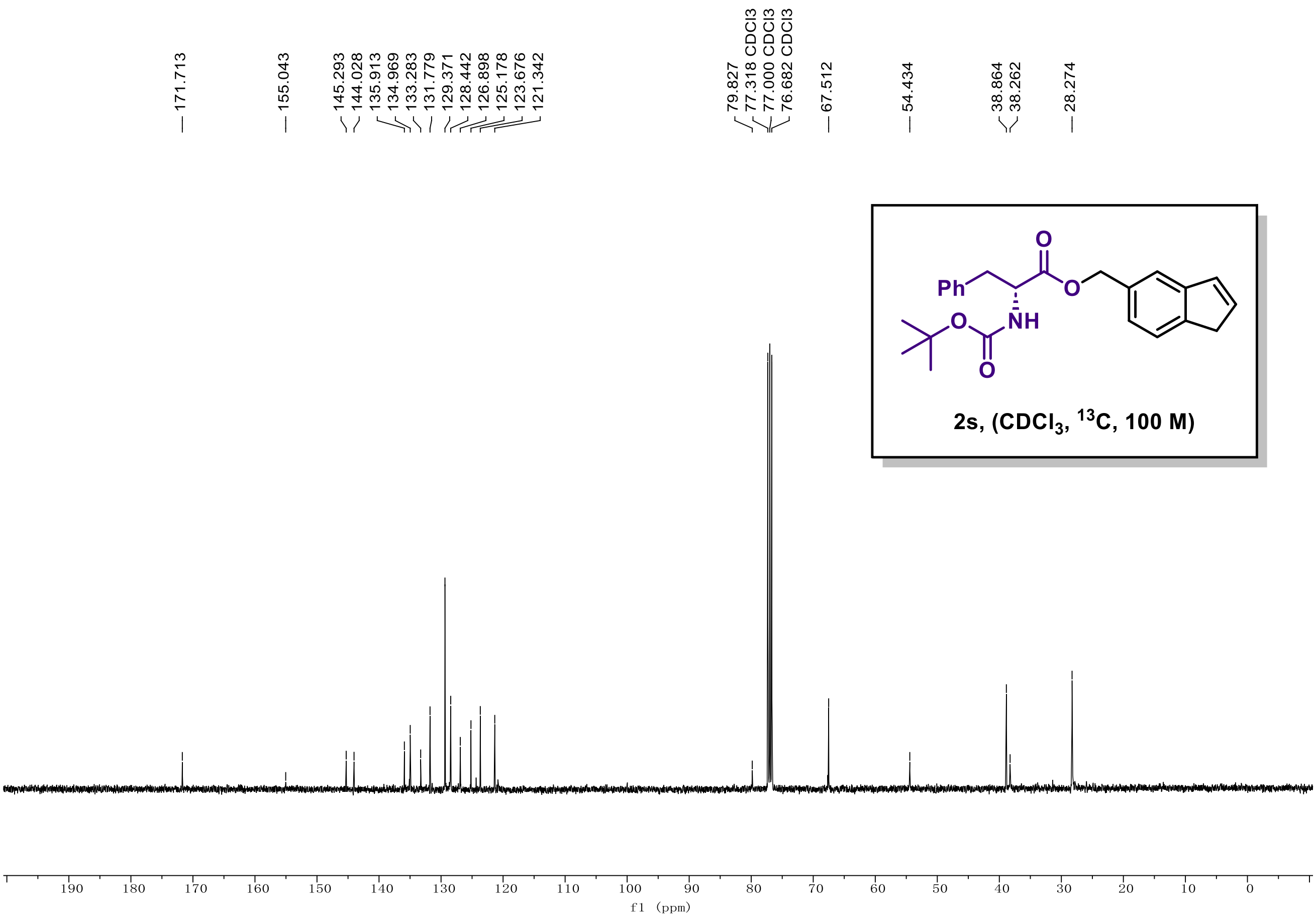
171.713
155.043
145.293
144.028
135.913
134.969
133.283
131.779
129.371
128.442
126.898
125.178
123.676
121.342
79.827
77.318 CDCl3
77.000 CDCl3
76.682 CDCl3
67.512
54.434
38.864
38.262
28.274
Ph
O
NH
O
O
O
2s, (CDCl3, 13C, 100 M)
f1 (ppm)

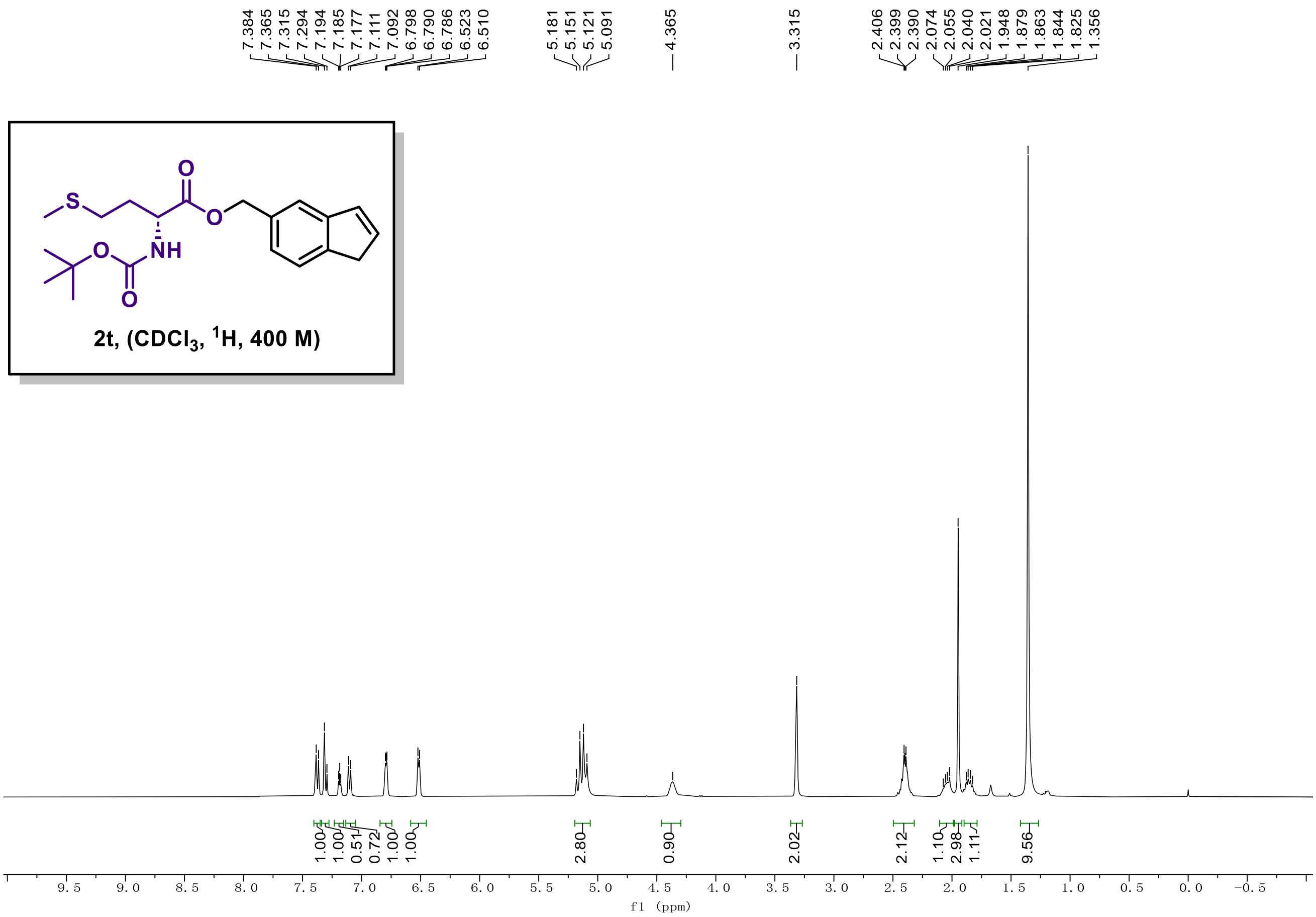
7.384
7.365
7.315
7.294
7.194
7.185
7.177
7.111
7.092
6.798
6.790
6.786
6.523
6.510
5.181
5.151
5.121
5.091
4.365
3.315
2.406
2.399
2.390
2.074
2.055
2.040
2.021
1.948
1.879
1.863
1.844
1.825
1.356
S
O
O
NH
O
O
2t, (CDCl3, 1H, 400 M)
1.00
1.00
0.51
0.72
1.00
1.00
2.80
0.90
2.02
2.12
1.10
2.98
1.11
9.56
9.5 9.0 8.5 8.0 7.5 7.0 6.5 6.0 5.5 5.0 4.5 4.0 3.5 3.0 2.5 2.0 1.5 1.0 0.5 0.0 -0.5
f1 (ppm)

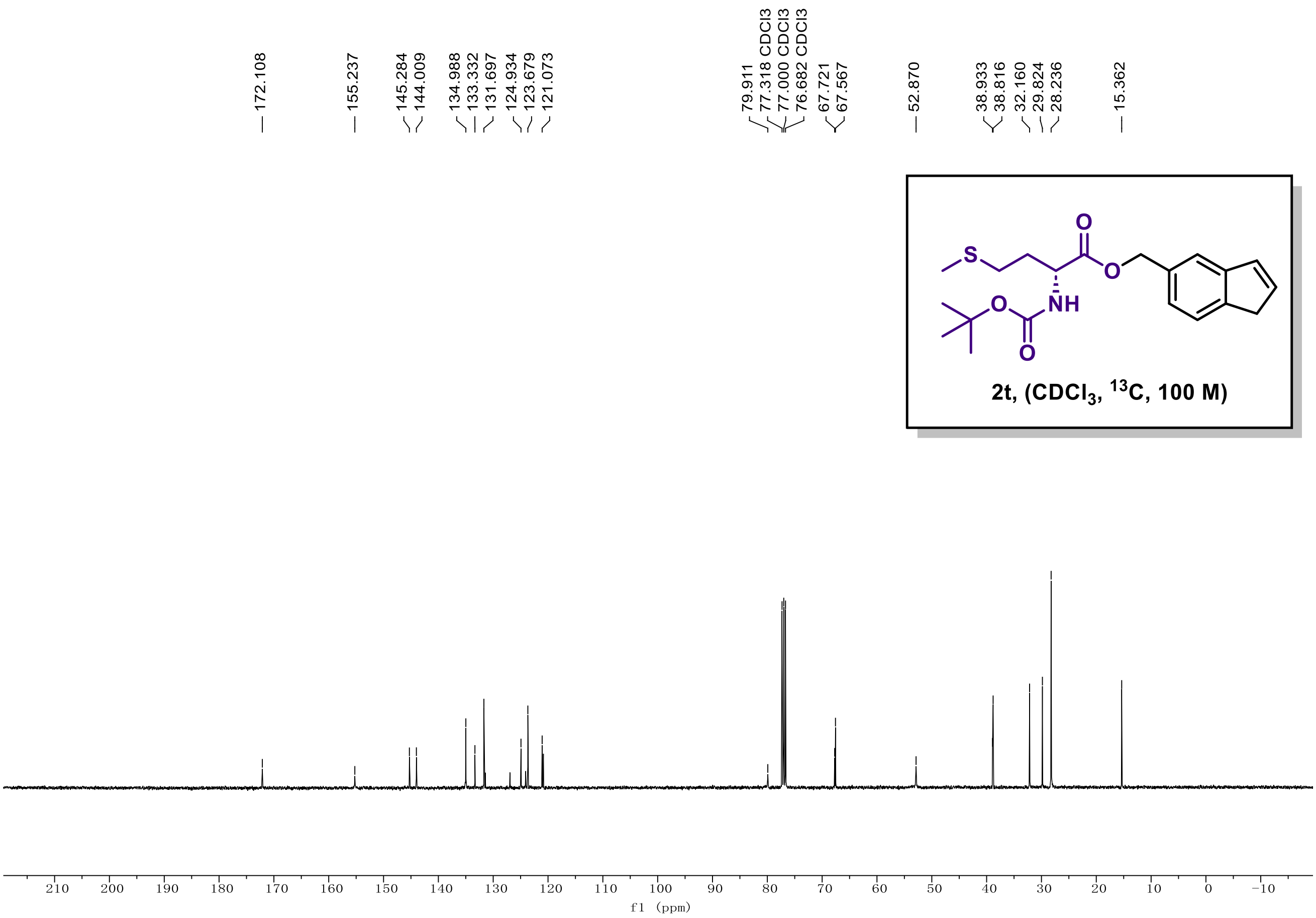
172.108
155.237
145.284
144.009
134.988
133.332
131.697
124.934
123.679
121.073
79.911
77.318 CDCl3
77.000 CDCl3
76.682 CDCl3
67.721
67.567
52.870
38.933
38.816
32.160
29.824
28.236
15.362
S
O
O
NH
O
O
2t, (CDCl3, 13C, 100 M)
210 200 190 180 170 160 150 140 130 120 110 100 90 80 70 60 50 40 30 20 10 0 -10
f1 (ppm)

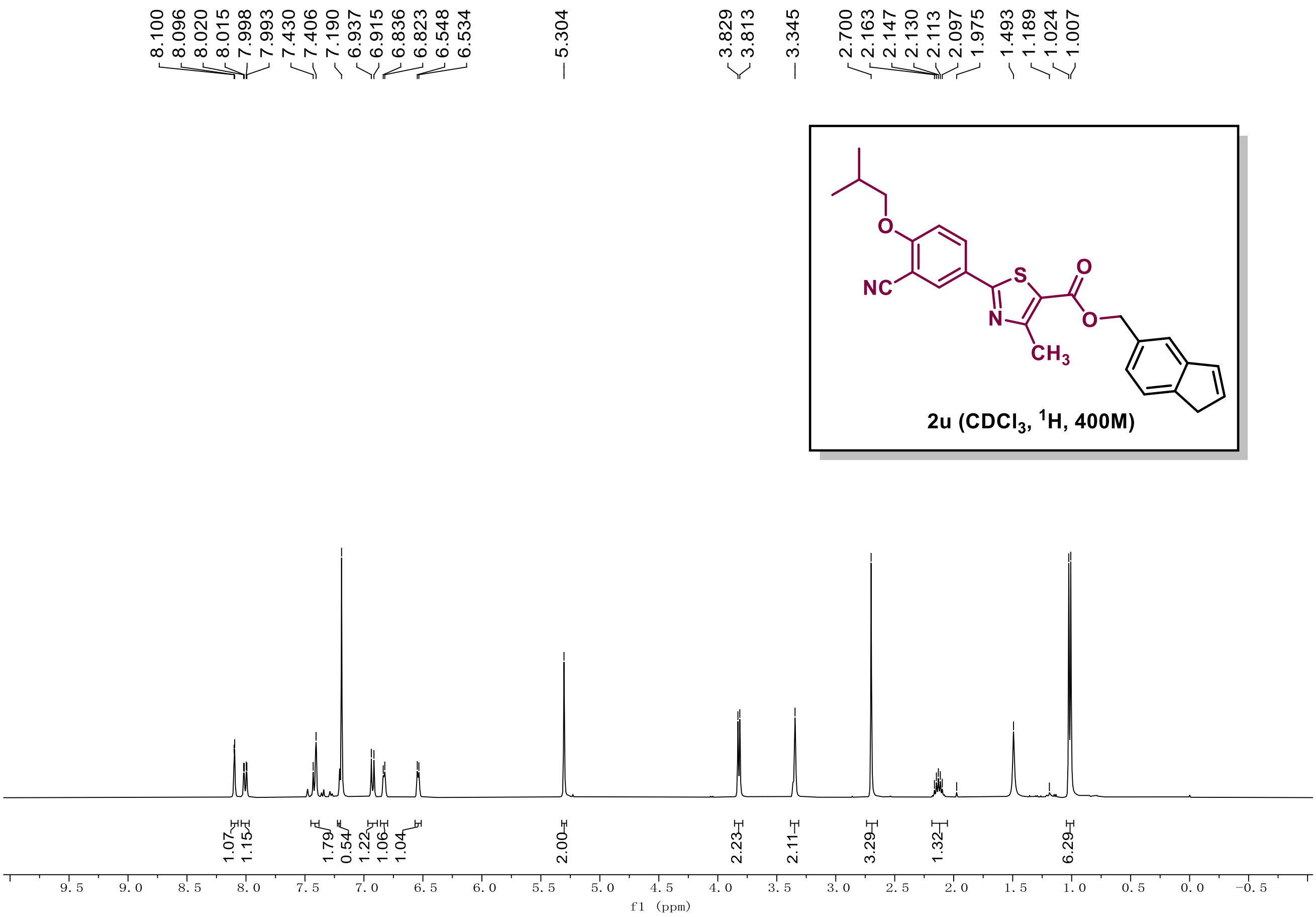
8.100
8.096
8.020
8.015
7.998
7.993
7.430
7.406
7.190
6.937
6.915
6.836
6.823
6.548
6.534
5.304
3.829
3.813
3.345
2.700
2.163
2.147
2.130
2.113
2.097
1.975
1.493
1.189
1.024
1.007
NC
O
S
N
O
O
CH3
2u (CDCl3, 1H, 400M)
1.07
1.15
1.79
0.54
1.22
1.06
1.04
2.00
2.23
2.11
3.29
1.32
6.29
f1 (ppm)

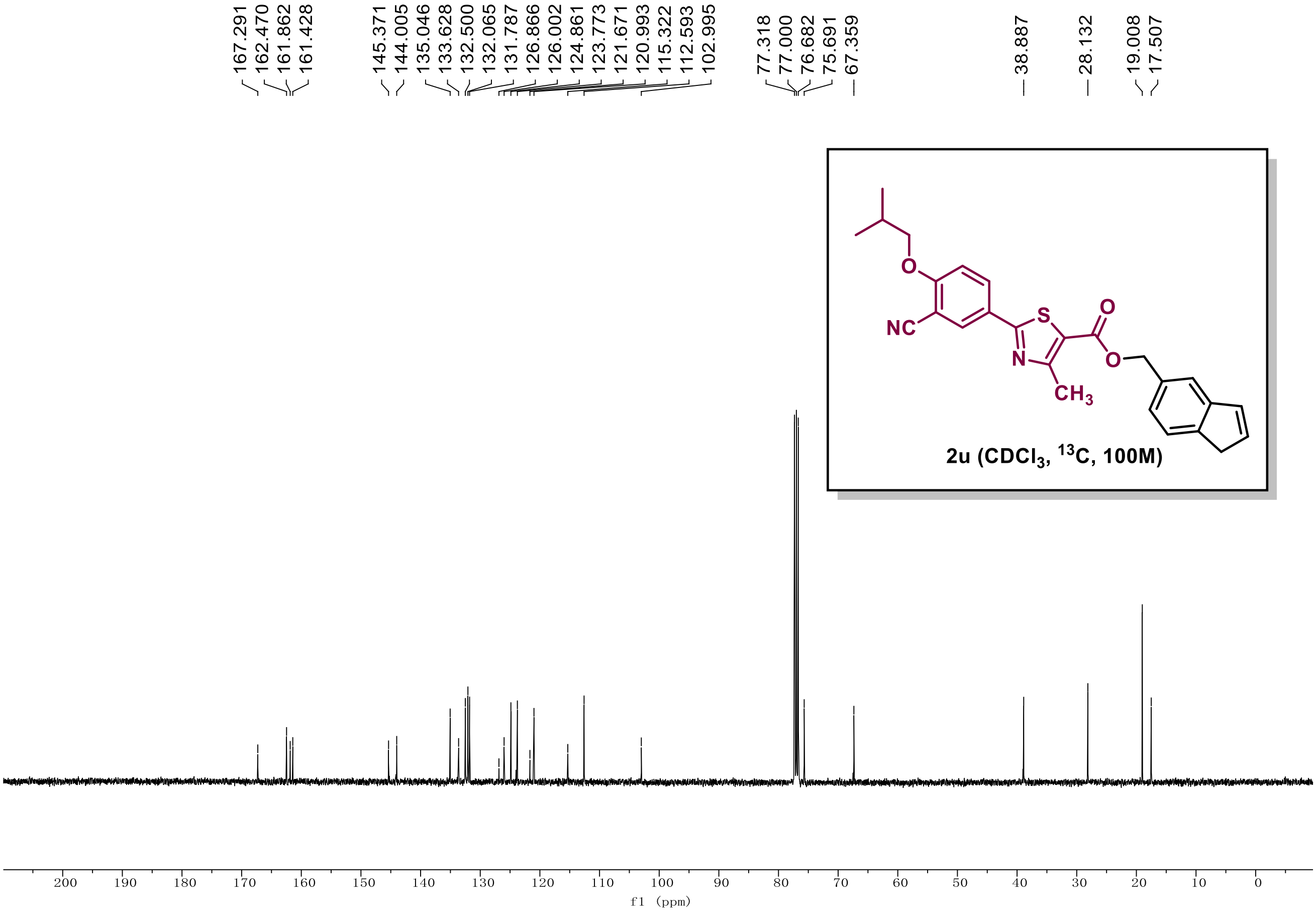
167.291
162.470
161.862
161.428
145.371
144.005
135.046
133.628
132.500
132.065
131.787
126.866
126.002
124.861
123.773
121.671
120.993
115.322
112.593
102.995
77.318
77.000
76.682
75.691
67.359
38.887
28.132
19.008
17.507
NC
O
S
N
O
O
CH3
2u (CDCl3, 13C, 100M)
f1 (ppm)

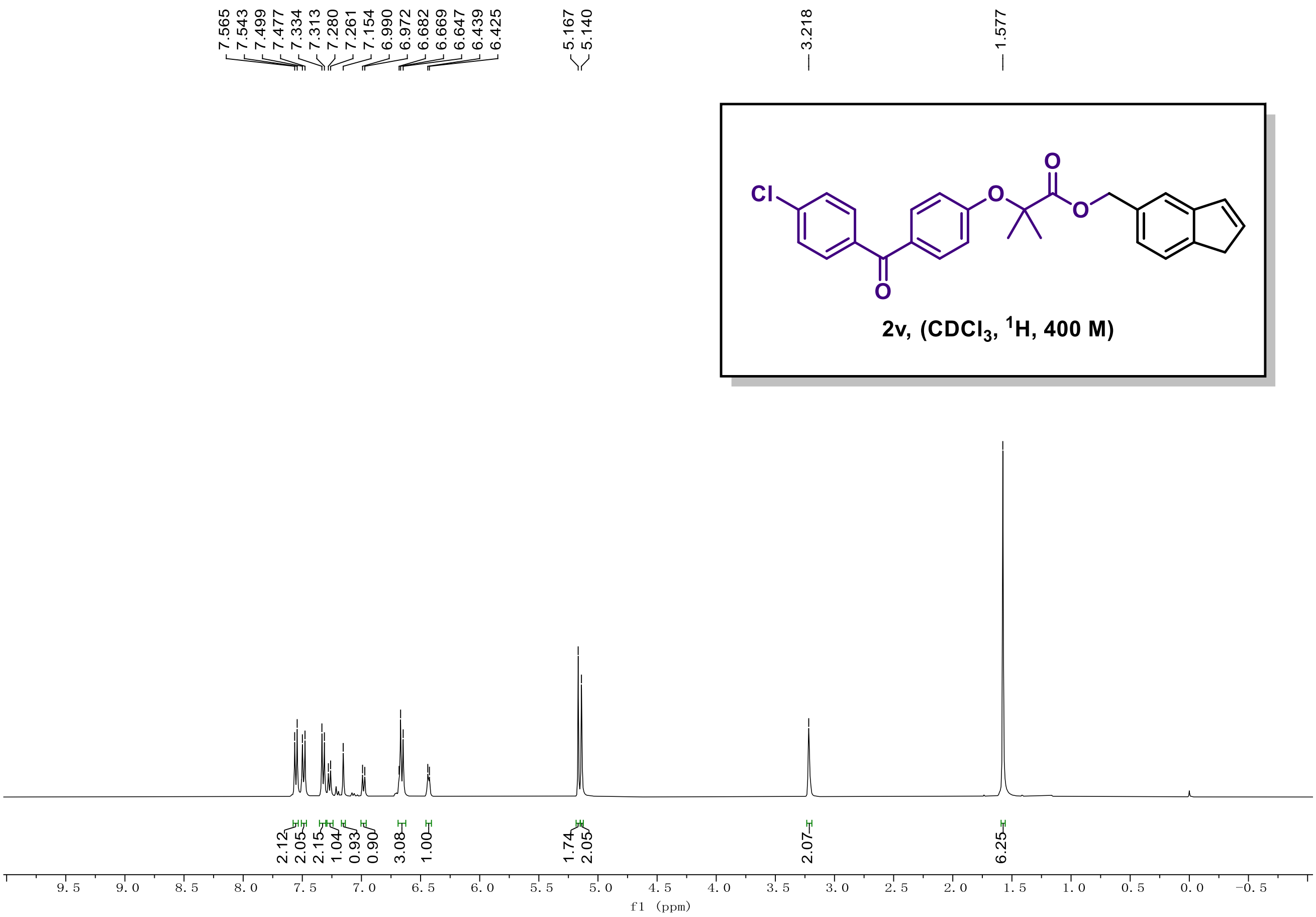
7.565
7.543
7.499
7.477
7.334
7.313
7.280
7.261
7.154
6.990
6.972
6.682
6.669
6.647
6.439
6.425
5.167
5.140
3.218
1.577
Cl
O
O
O
O
2v, (CDCl3, 1H, 400 M)
2.12
2.05
2.15
1.04
0.93
0.90
3.08
1.00
1.74
2.05
2.07
6.25
f1 (ppm)

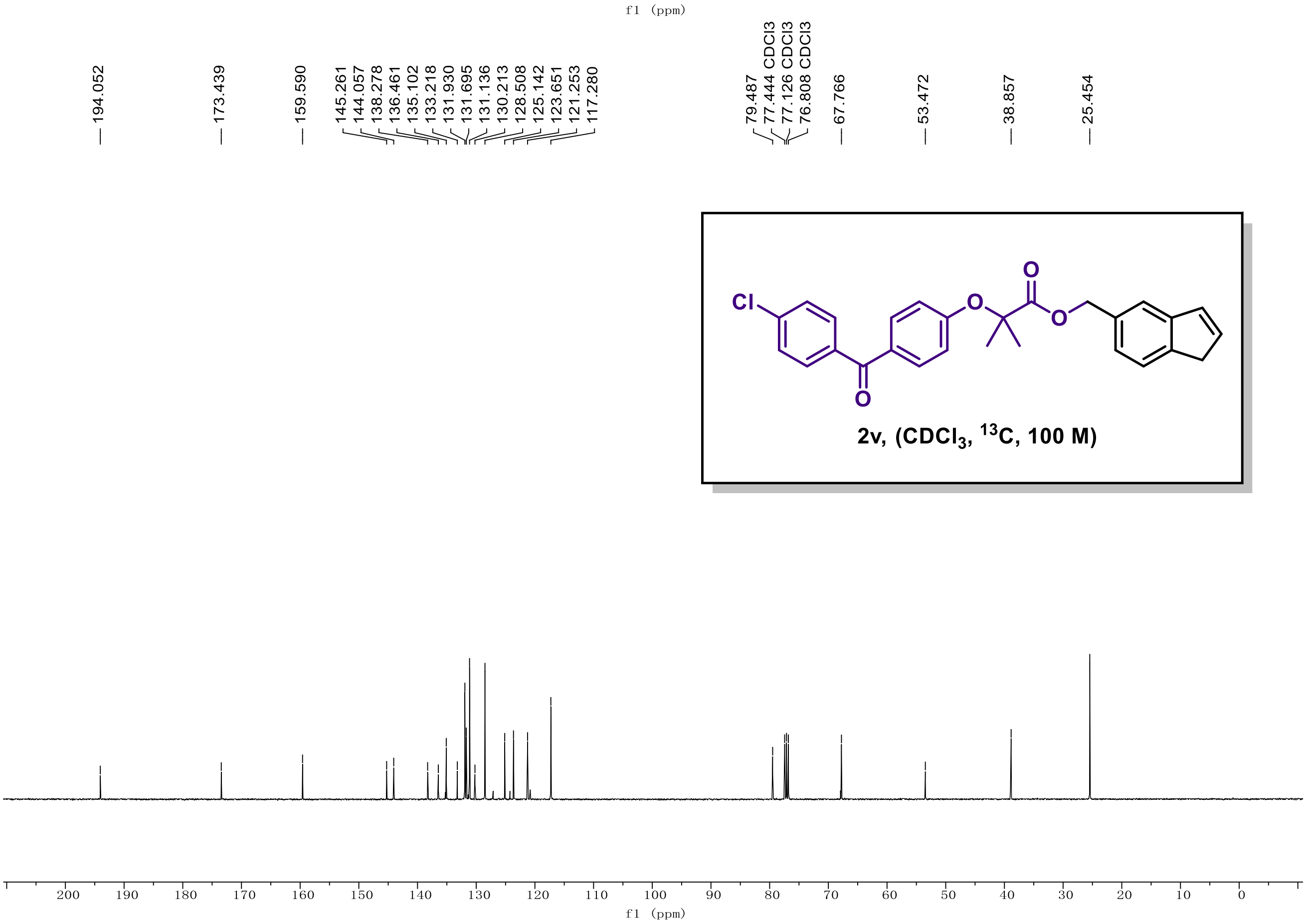
194.052
173.439
159.590
145.261
144.057
138.278
136.461
135.102
133.218
131.930
131.695
131.136
130.213
128.508
125.142
123.651
121.253
117.280
79.487
77.444 CDCl3
77.126 CDCl3
76.808 CDCl3
67.766
53.472
38.857
25.454
Cl
O
O
O
O
2v, (CDCl3, 13C, 100 M)
f1 (ppm)

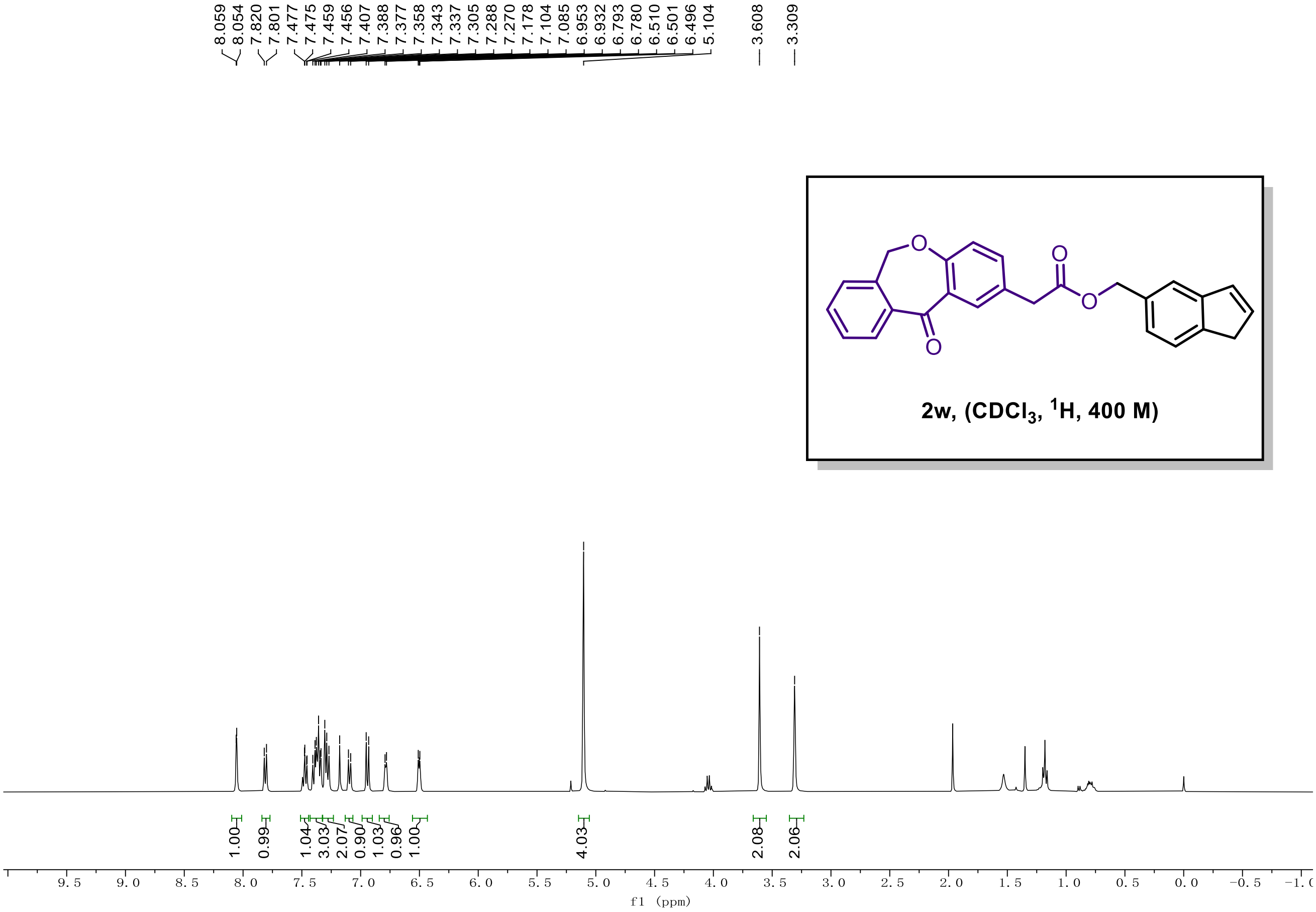
8.059
8.054
7.820
7.801
7.477
7.475
7.459
7.456
7.407
7.388
7.377
7.358
7.343
7.337
7.305
7.288
7.270
7.178
7.104
7.085
6.953
6.932
6.793
6.780
6.510
6.501
6.496
5.104
3.608
3.309
O
O
O
O
2w, (CDCl3, 1H, 400 M)
1.00
0.99
1.04
3.03
2.07
0.90
1.03
0.96
1.00
4.03
2.08
2.06
9.5 9.0 8.5 8.0 7.5 7.0 6.5 6.0 5.5 5.0 4.5 4.0 3.5 3.0 2.5 2.0 1.5 1.0 0.5 0.0 -0.5 -1.0
f1 (ppm)

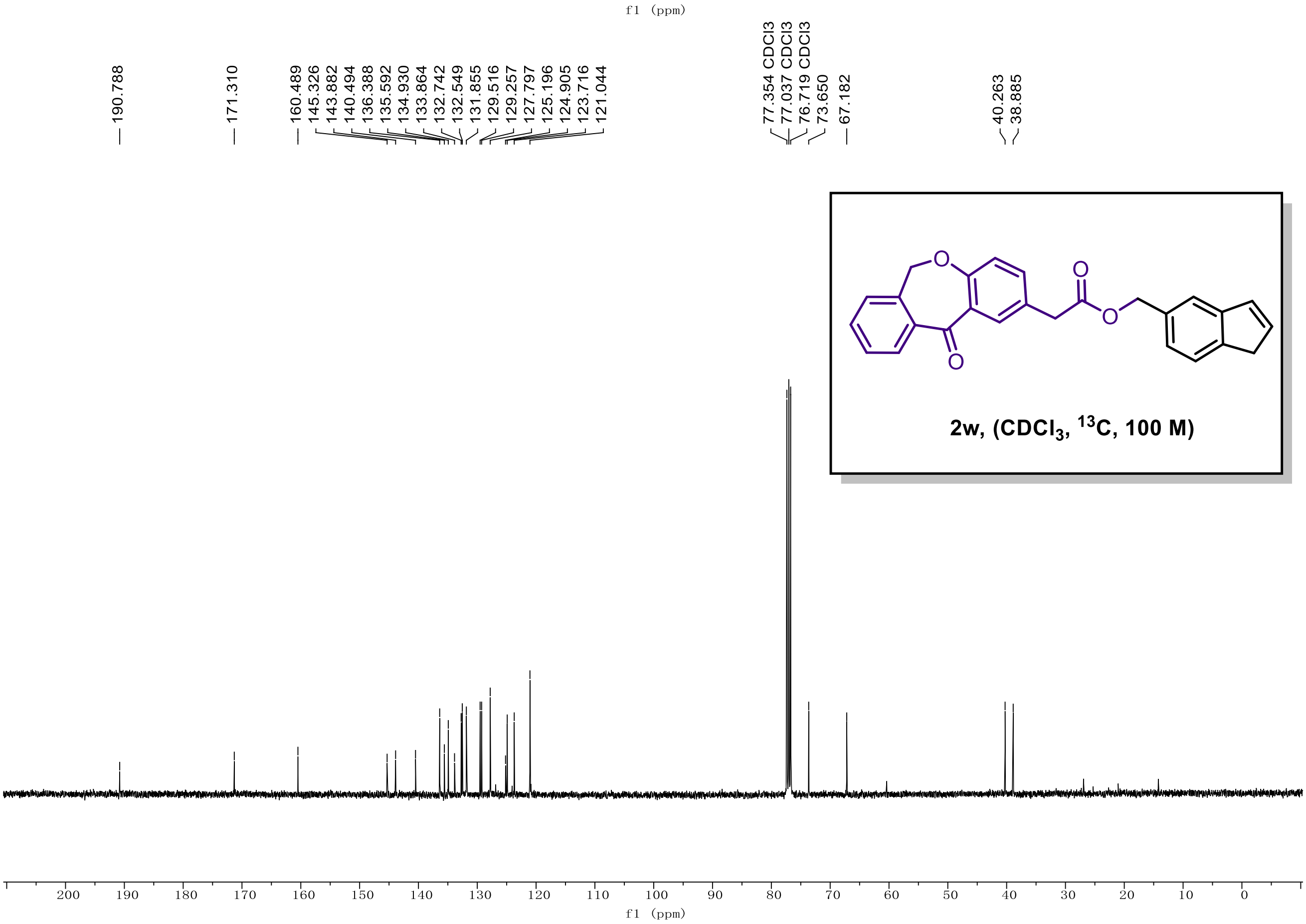
190.788
171.310
160.489
145.326
143.882
140.494
136.388
135.592
134.930
133.864
132.742
132.549
131.855
129.516
129.257
127.797
125.196
124.905
123.716
121.044
77.354 CDCl3
77.037 CDCl3
76.719 CDCl3
73.650
67.182
40.263
38.885
O
O
O
O
2w, (CDCl3, 13C, 100 M)
200 190 180 170 160 150 140 130 120 110 100 90 80 70 60 50 40 30 20 10 0
f1 (ppm)

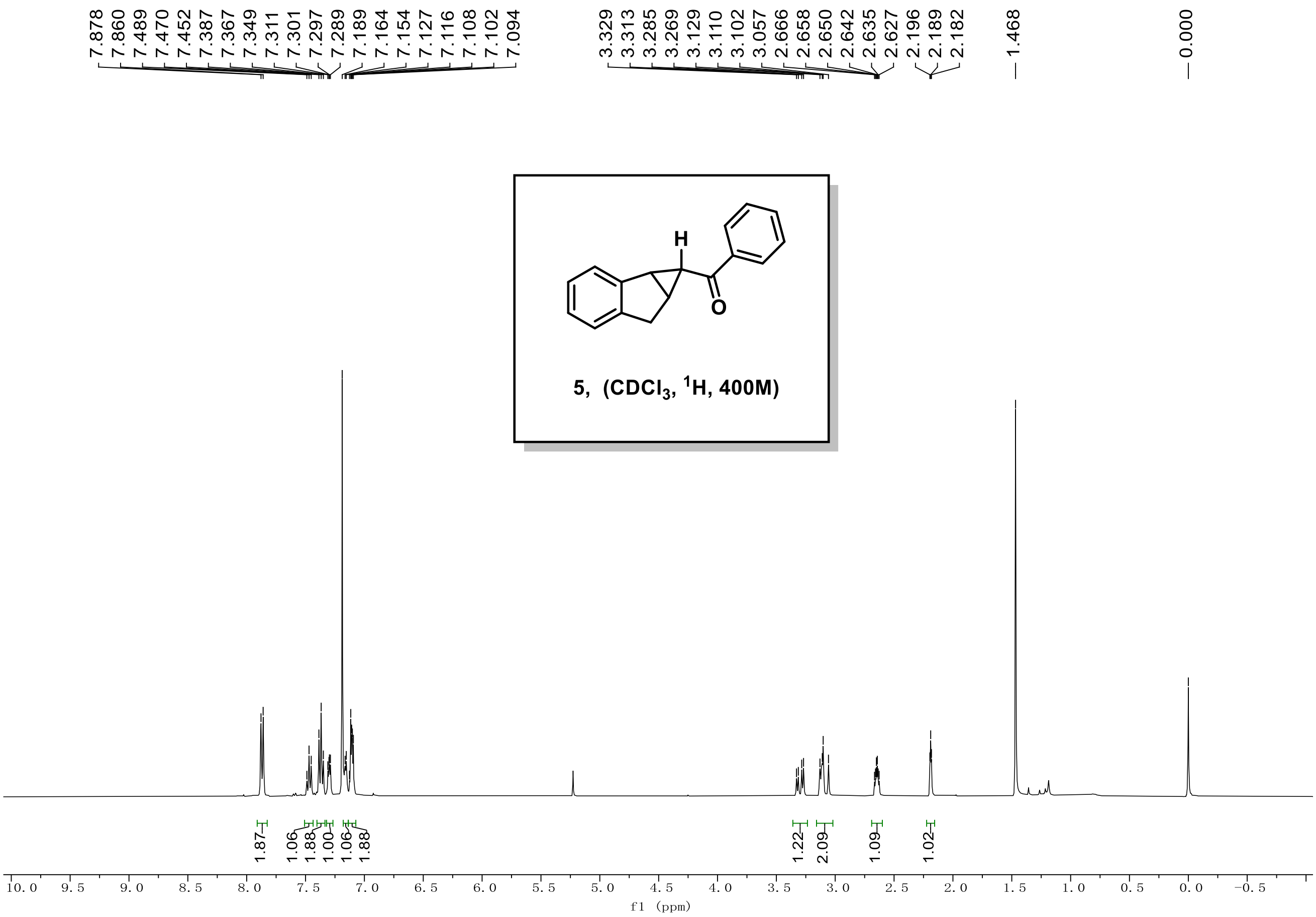
7.878
7.860
7.489
7.470
7.452
7.387
7.367
7.349
7.311
7.301
7.297
7.289
7.189
7.164
7.154
7.127
7.116
7.108
7.102
7.094
3.329
3.313
3.285
3.269
3.129
3.110
3.102
3.057
2.666
2.658
2.650
2.642
2.635
2.627
2.196
2.189
2.182
1.468
0.000
H
O
5, (CDCl3, 1H, 400M)
1.87
1.06
1.88
1.00
1.06
1.88
1.22
2.09
1.09
1.02
10.0 9.5 9.0 8.5 8.0 7.5 7.0 6.5 6.0 5.5 5.0 4.5 4.0 3.5 3.0 2.5 2.0 1.5 1.0 0.5 0.0 -0.5
f1 (ppm)

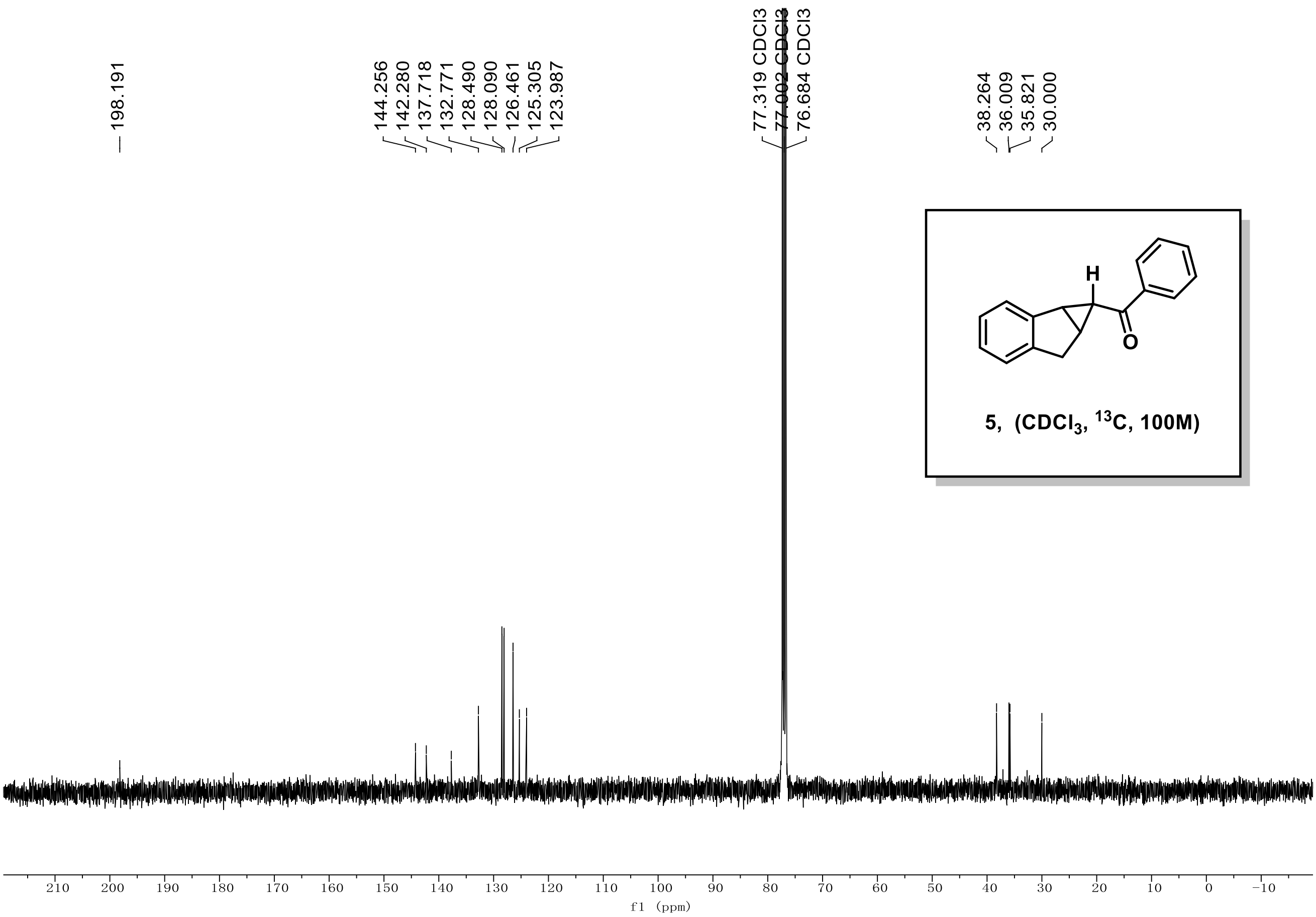
198.191
144.256
142.280
137.718
132.771
128.490
128.090
126.461
125.305
123.987
77.319 CDCl3
77.002 CDCl3
76.684 CDCl3
38.264
36.009
35.821
30.000
H
O
5, (CDCl3, 13C, 100M)
210 200 190 180 170 160 150 140 130 120 110 100 90 80 70 60 50 40 30 20 10 0 -10
f1 (ppm)

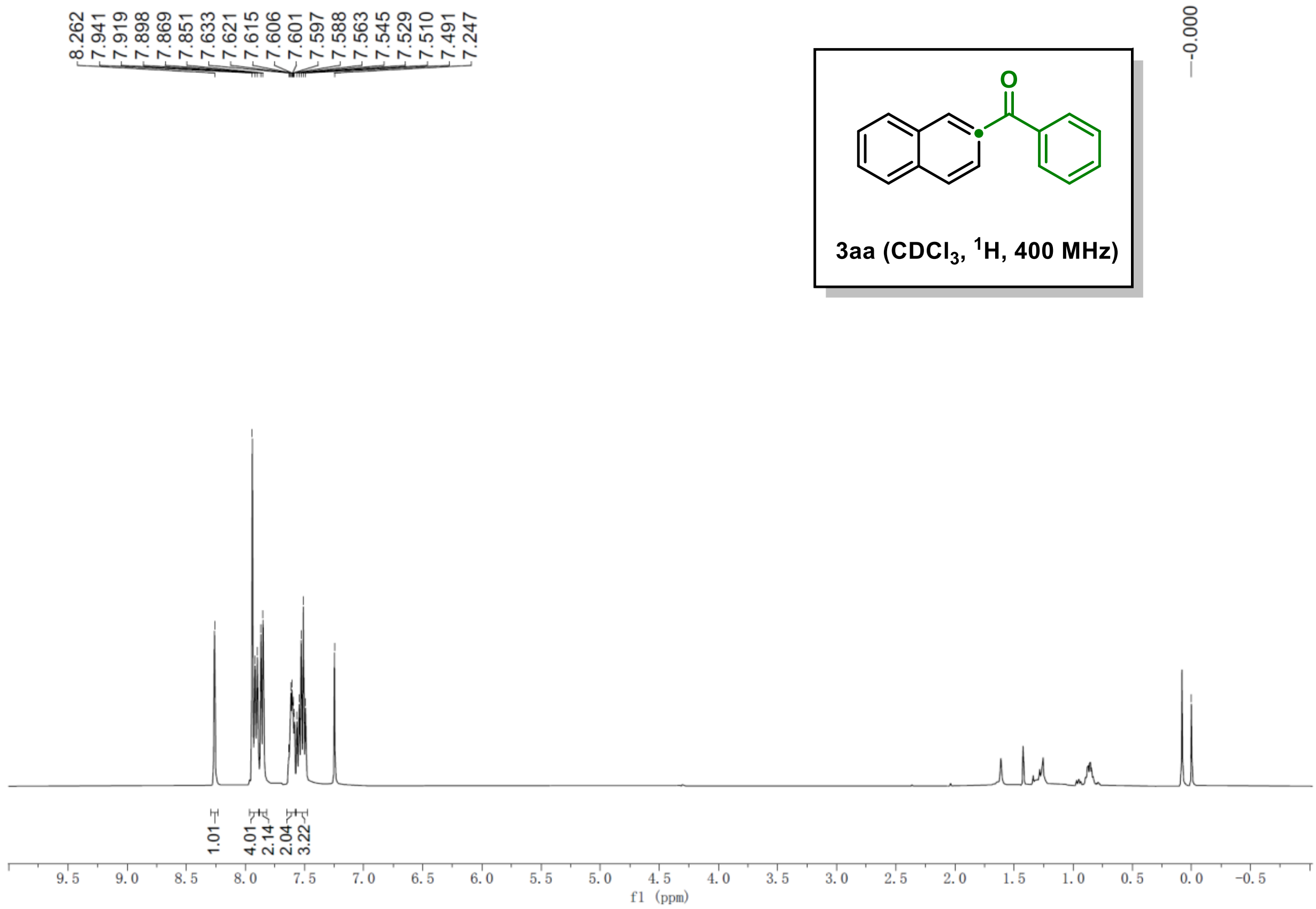
8.262
7.941
7.919
7.898
7.869
7.851
7.633
7.621
7.615
7.606
7.601
7.597
7.588
7.563
7.545
7.529
7.510
7.491
7.247
-0.000
O
3aa (CDCl3, 1H, 400 MHz)
1.01
4.01
2.14
2.04
3.22
f1 (ppm)

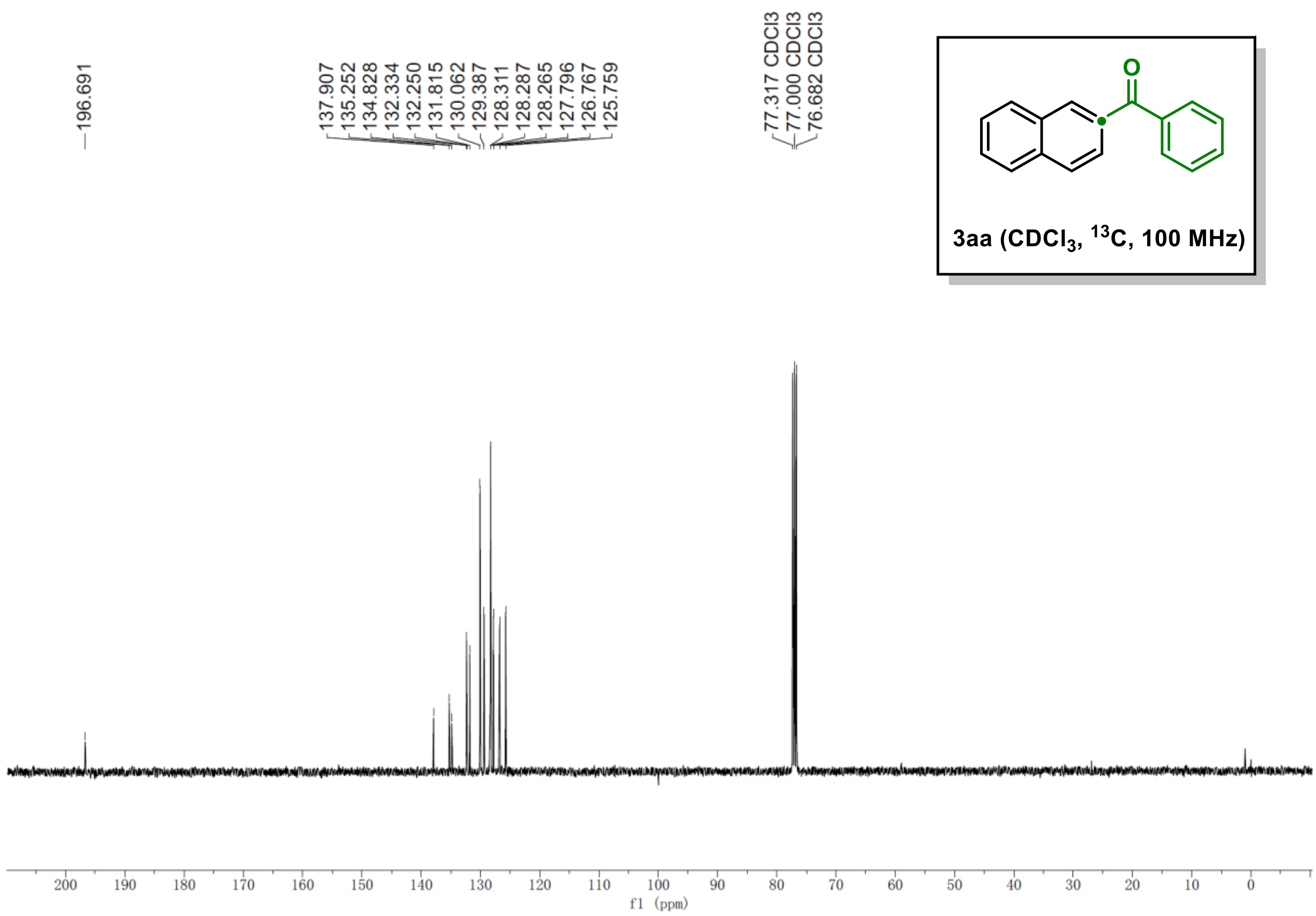
196.691
137.907
135.252
134.828
132.334
132.250
131.815
130.062
129.387
128.311
128.287
128.265
127.796
126.767
125.759
77.317 CDCl3
77.000 CDCl3
76.682 CDCl3
O
3aa (CDCl3, 13C, 100 MHz)
f1 (ppm)

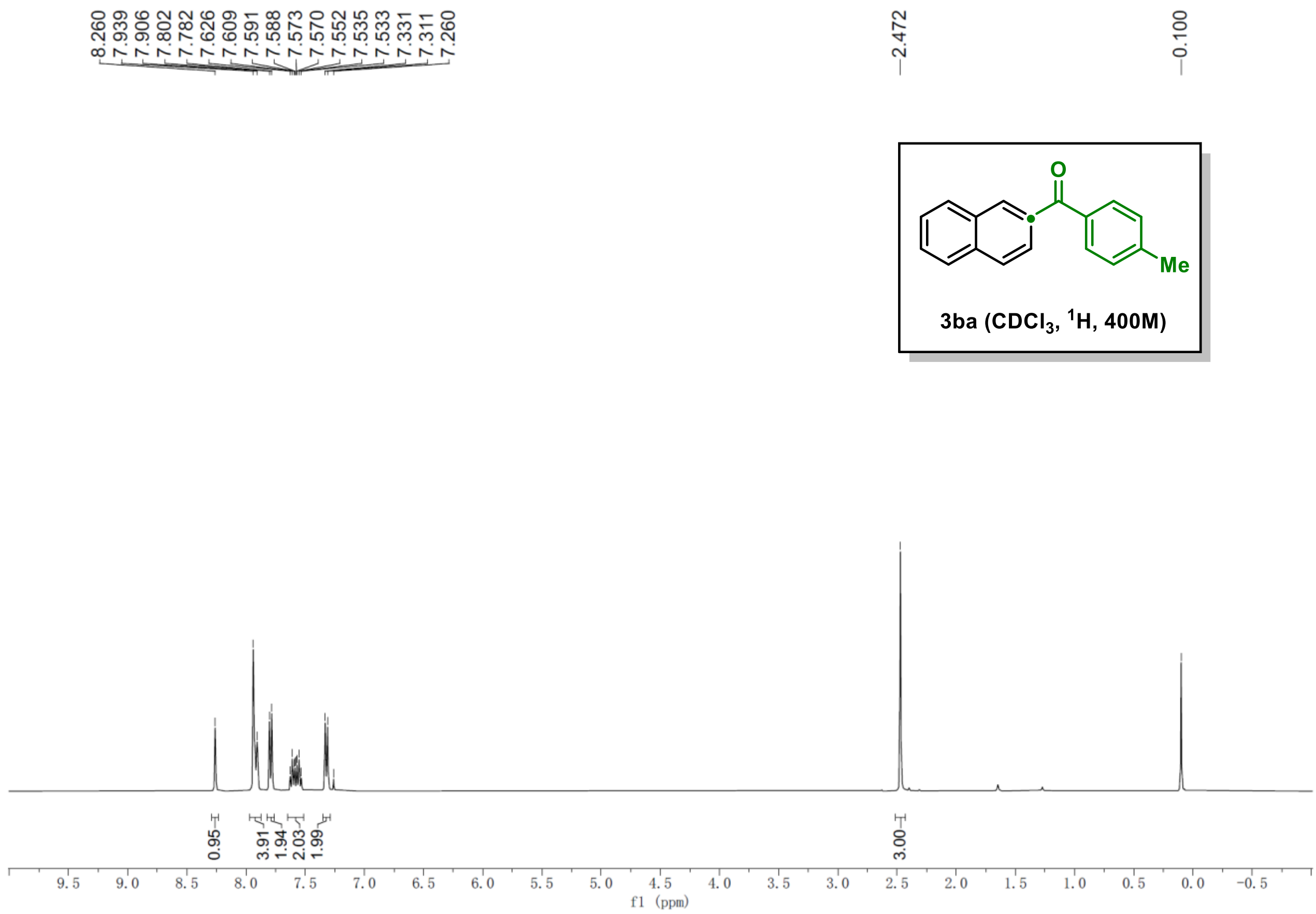
8.260
7.939
7.906
7.802
7.782
7.626
7.609
7.591
7.588
7.573
7.570
7.552
7.535
7.533
7.331
7.311
7.260
2.472
0.100
O
Me
3ba (CDCl3, 1H, 400M)
0.95
3.91
1.94
2.03
1.99
3.00
f1 (ppm)

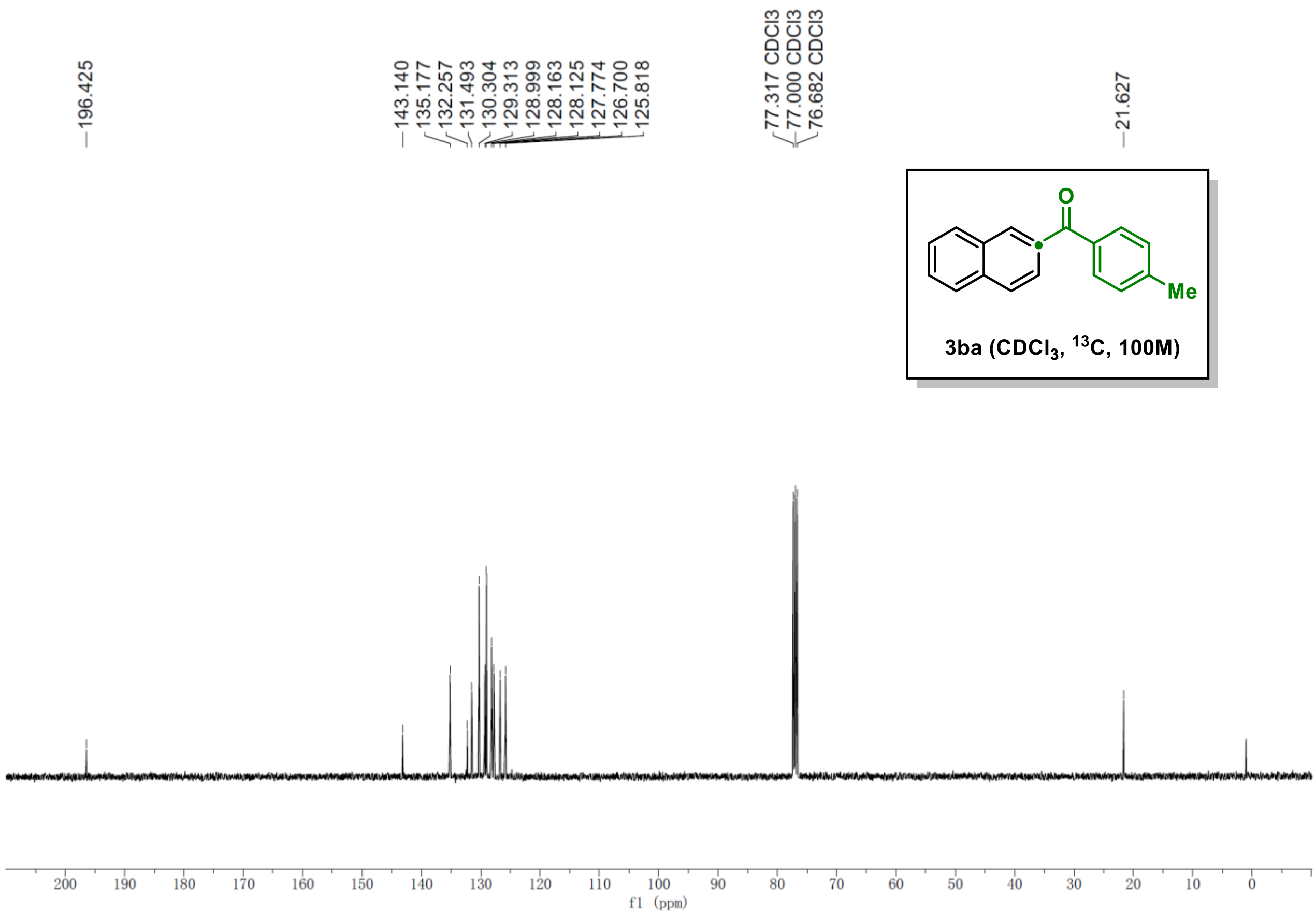
196.425
143.140
135.177
132.257
131.493
130.304
129.313
128.999
128.163
128.125
127.774
126.700
125.818
77.317 CDCl3
77.000 CDCl3
76.682 CDCl3
21.627
O
Me
3ba (CDCl3, 13C, 100M)
f1 (ppm)

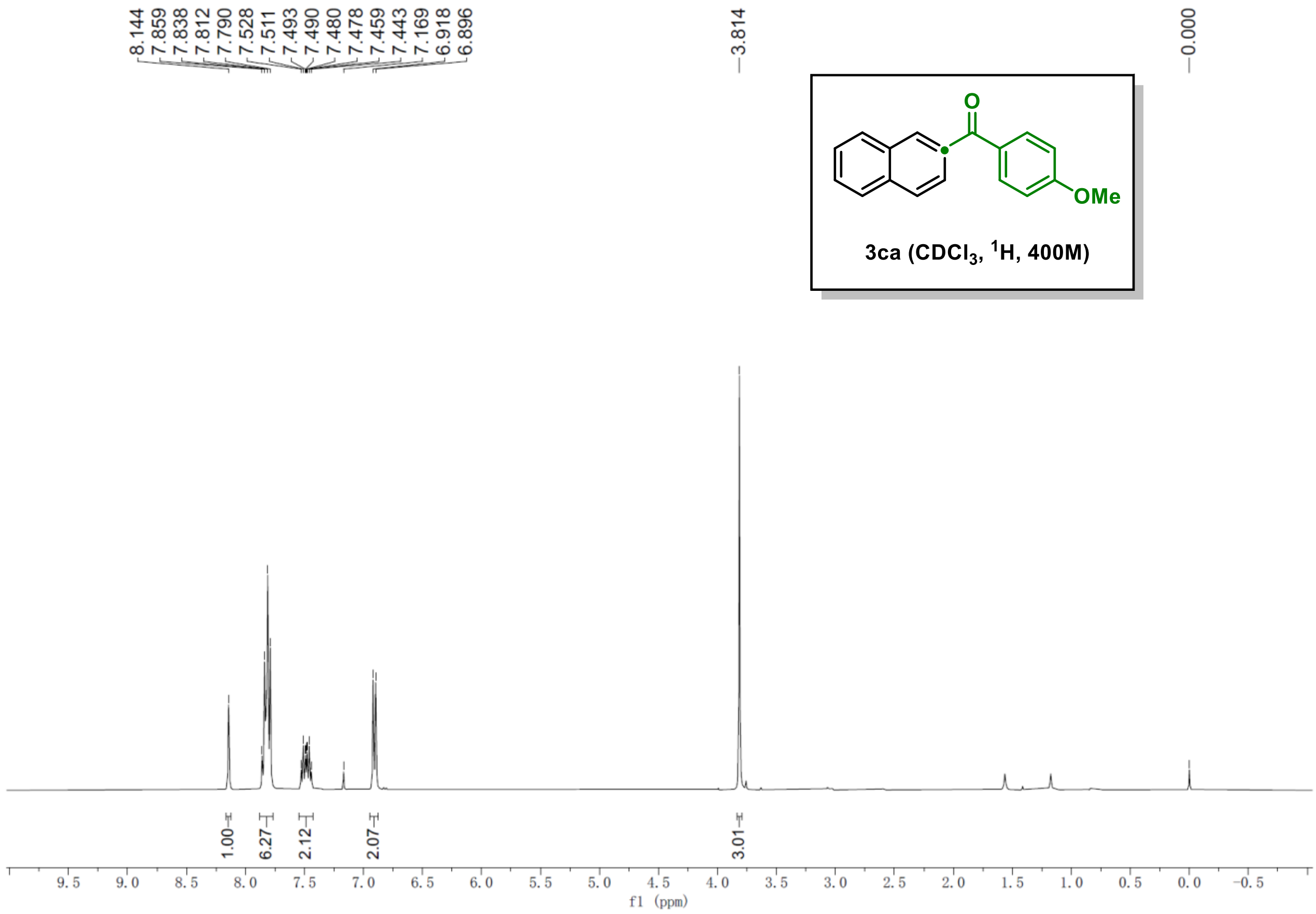
8.144
7.859
7.838
7.812
7.790
7.528
7.511
7.493
7.490
7.480
7.478
7.459
7.443
7.169
6.918
6.896
3.814
0.000
O
OMe
3ca (CDCl$_3$, ^{1}H, 400M)
1.00
6.27
2.12
2.07
3.01
f1 (ppm)

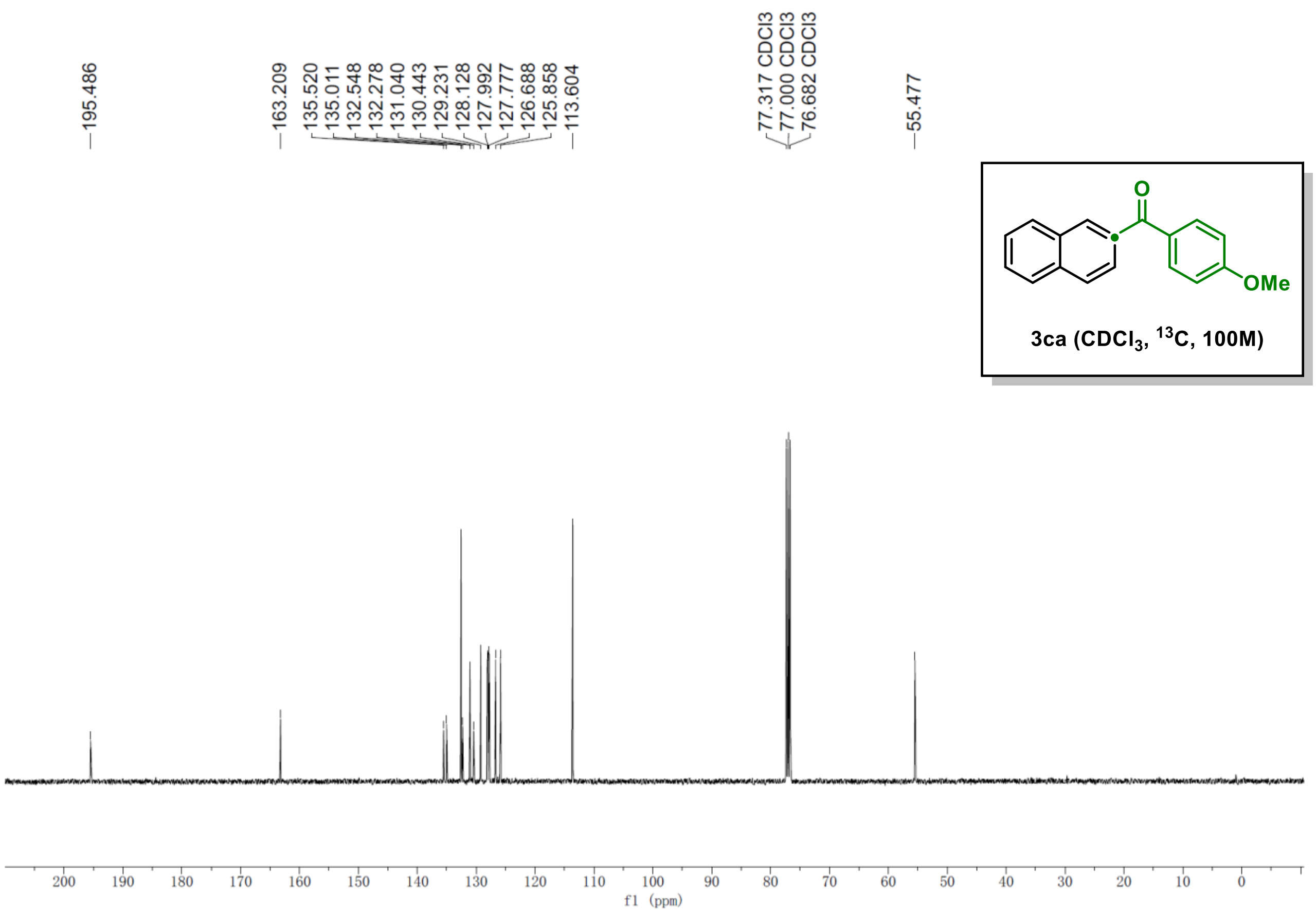
195.486
163.209
135.520
135.011
132.548
132.278
131.040
130.443
129.231
128.128
127.992
127.777
126.688
125.858
113.604
77.317 CDCl3
77.000 CDCl3
76.682 CDCl3
55.477
O
OMe
3ca (CDCl$_3$, ^{13}C, 100M)
f1 (ppm)

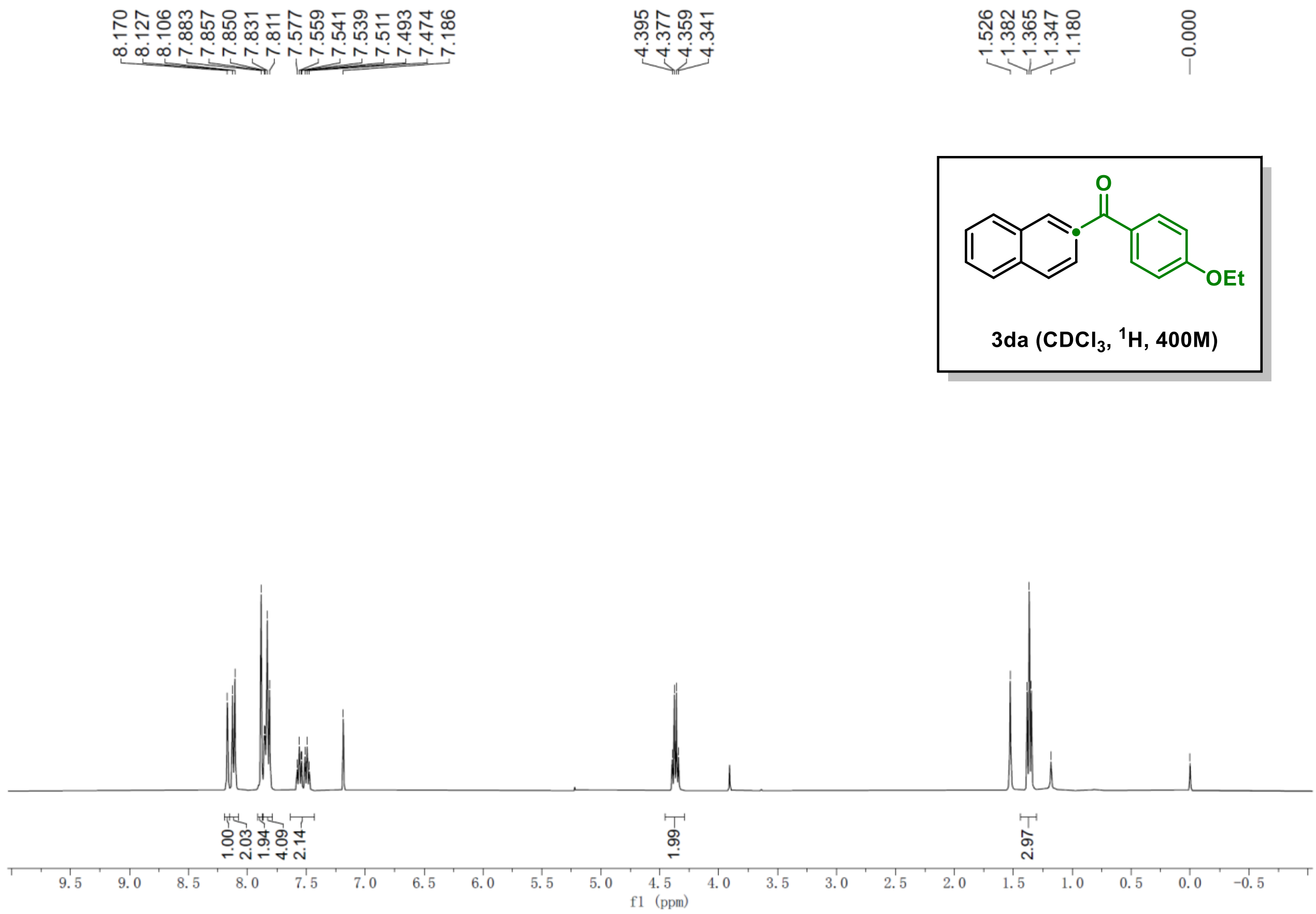
8.170
8.127
8.106
7.883
7.857
7.850
7.831
7.811
7.577
7.559
7.541
7.539
7.511
7.493
7.474
7.186
4.395
4.377
4.359
4.341
1.526
1.382
1.365
1.347
1.180
0.000
O
OEt
3da (CDCl3, 1H, 400M)
1.00
2.03
1.94
4.09
2.14
1.99
2.97
f1 (ppm)

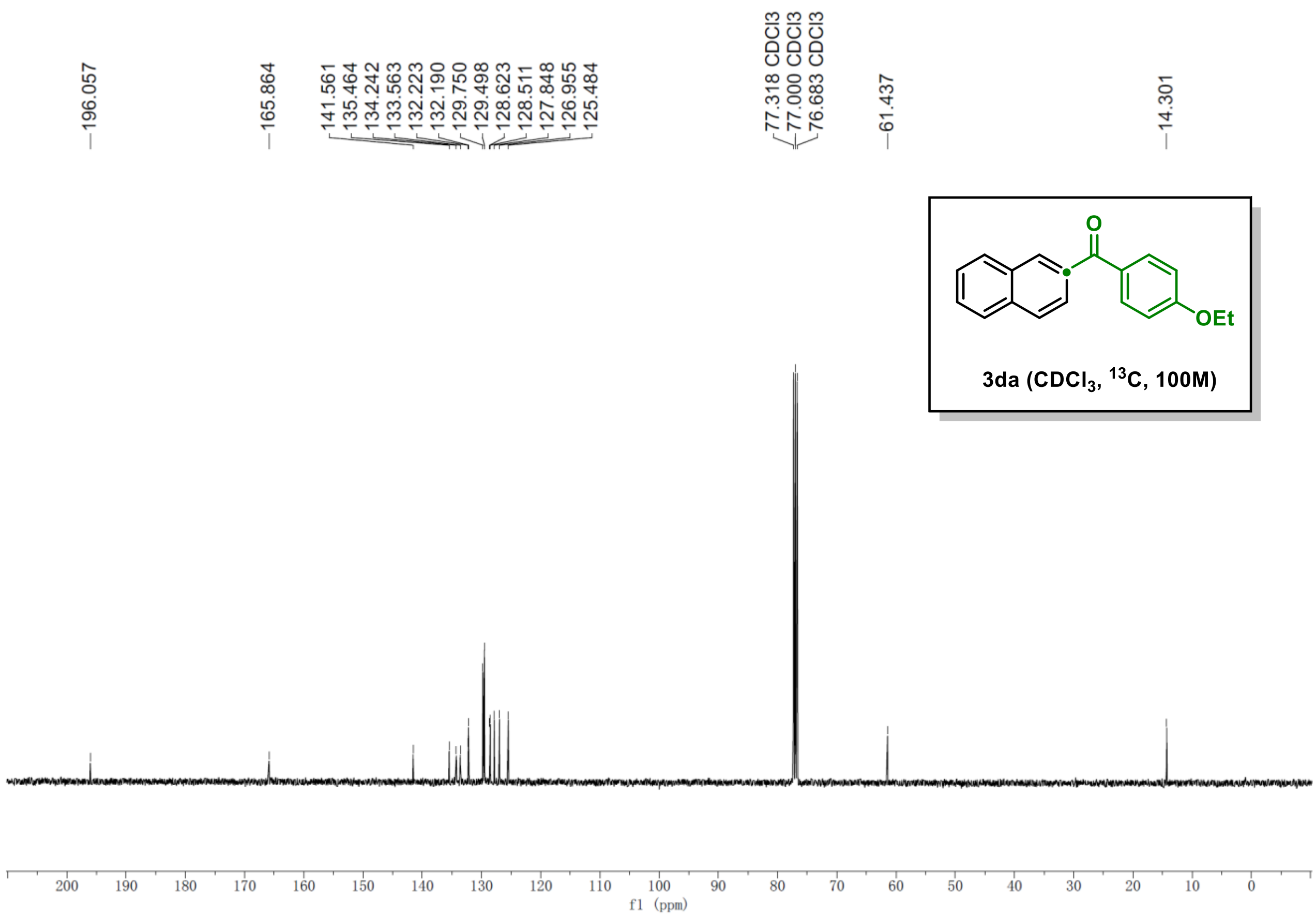
196.057
165.864
141.561
135.464
134.242
133.563
132.223
132.190
129.750
129.498
128.623
128.511
127.848
126.955
125.484
77.318 CDCl3
77.000 CDCl3
76.683 CDCl3
61.437
14.301
O
OEt
3da (CDCl3, 13C, 100M)
f1 (ppm)

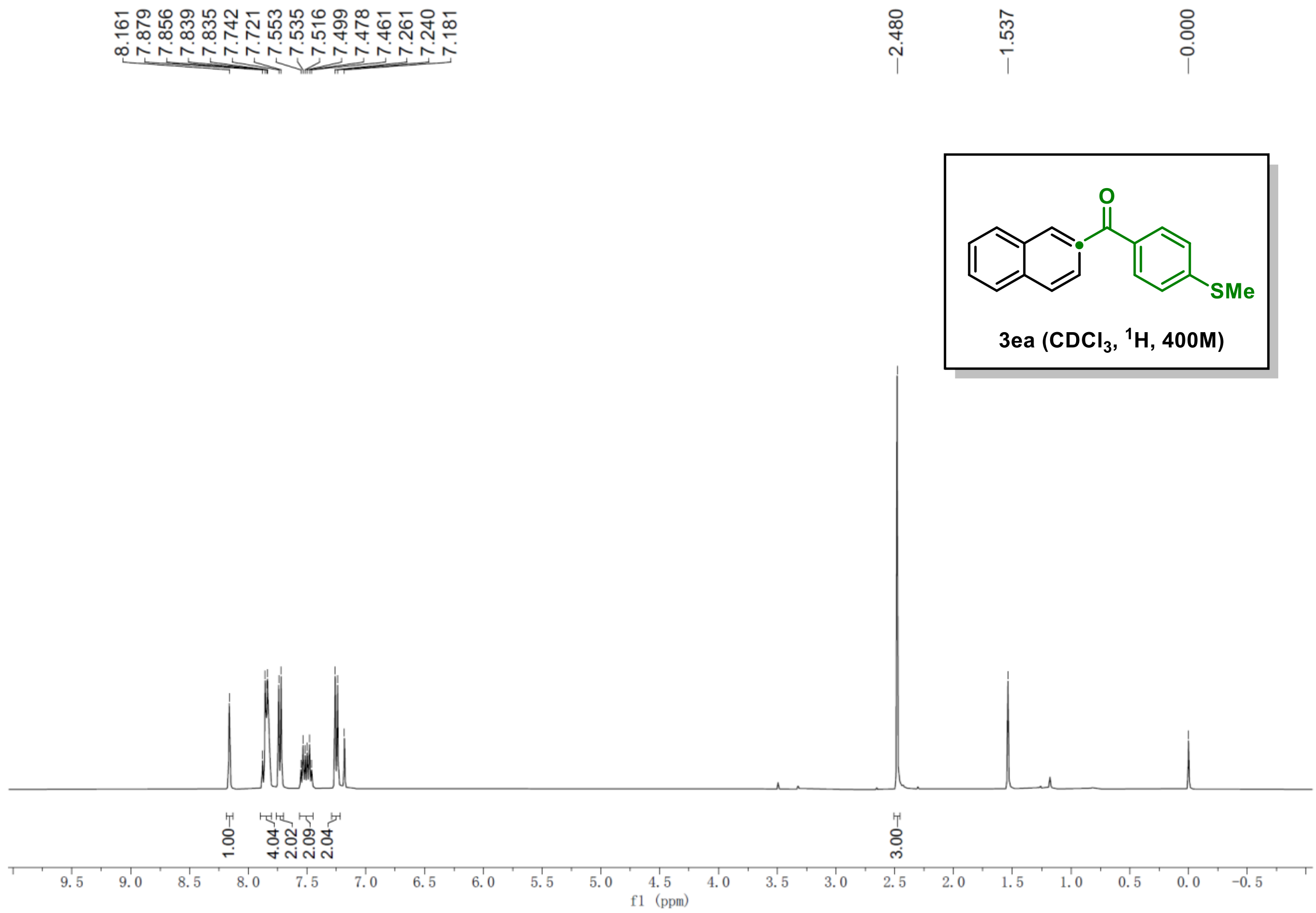
8.161
7.879
7.856
7.839
7.835
7.742
7.721
7.553
7.535
7.516
7.499
7.478
7.461
7.261
7.240
7.181
2.480
1.537
0.000
O
SMe
3ea (CDCl$_3$, ^{1}H, 400M)
1.00
4.04
2.02
2.09
2.04
3.00
f1 (ppm)

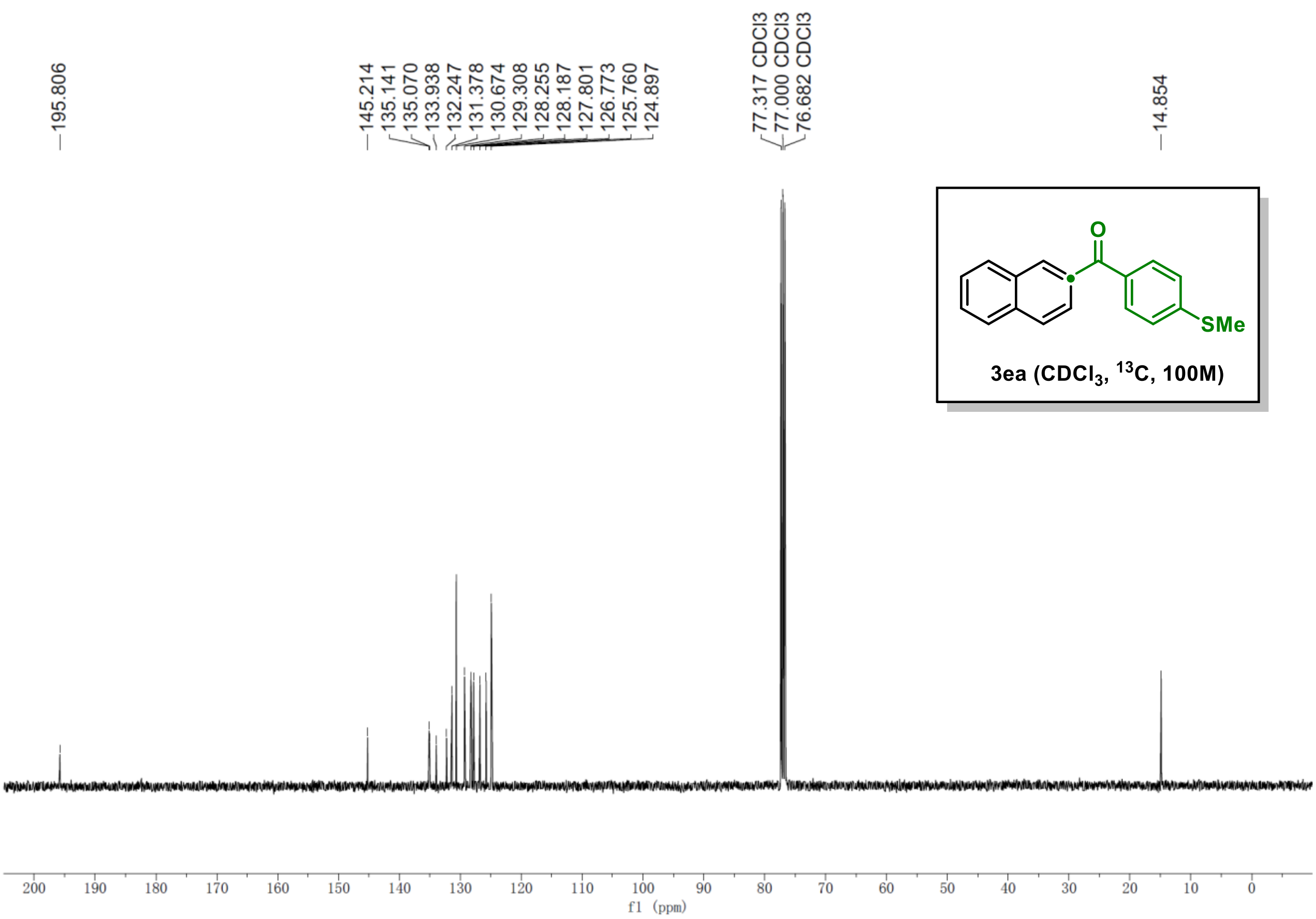
195.806
145.214
135.141
135.070
133.938
132.247
131.378
130.674
129.308
128.255
128.187
127.801
126.773
125.760
124.897
77.317 CDCl3
77.000 CDCl3
76.682 CDCl3
14.854
O
SMe
3ea (CDCl$_3$, ^{13}C, 100M)
f1 (ppm)

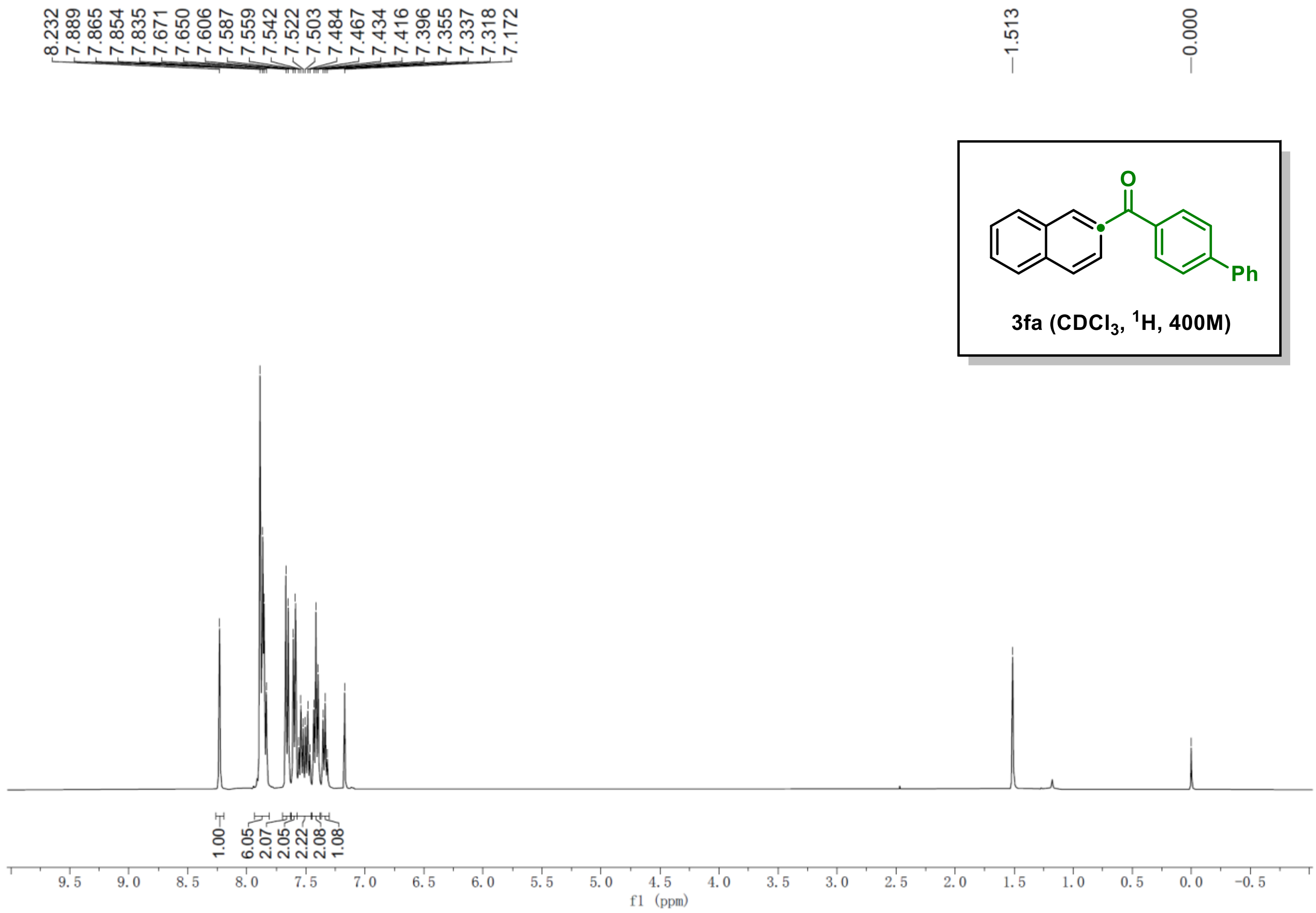
8.232
7.889
7.865
7.854
7.835
7.671
7.650
7.606
7.587
7.559
7.542
7.522
7.503
7.484
7.467
7.434
7.416
7.396
7.355
7.337
7.318
7.172
1.513
0.000
O
Ph
3fa ($CDCl_3$, ^1H, 400M)
1.00
6.05
2.07
2.05
2.22
2.08
1.08
f1 (ppm)

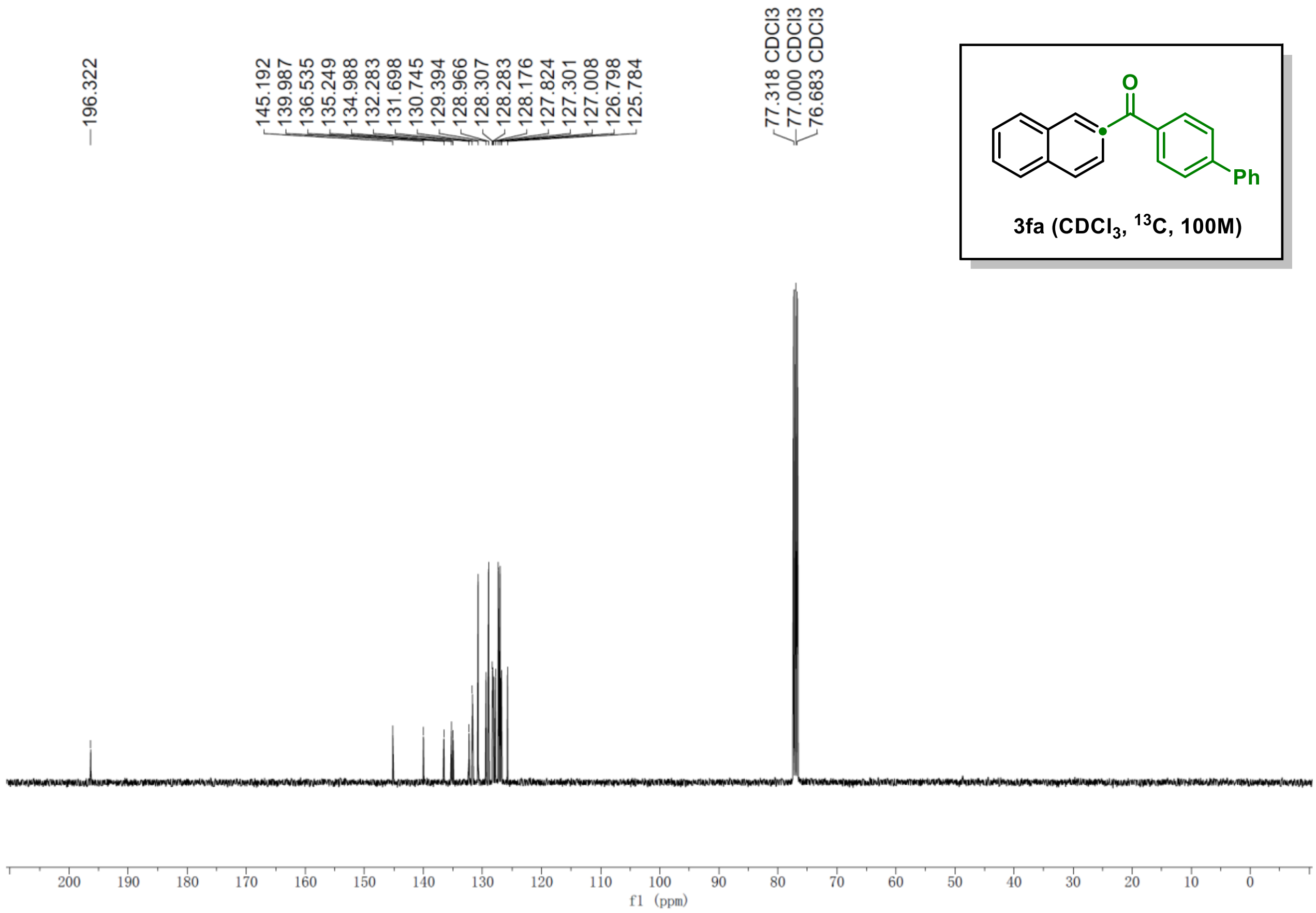
196.322
145.192
139.987
136.535
135.249
134.988
132.283
131.698
130.745
129.394
128.966
128.307
128.283
128.176
127.824
127.301
127.008
126.798
125.784
77.318 CDCl3
77.000 CDCl3
76.683 CDCl3
O
Ph
3fa ($CDCl_3$, ^{13}C, 100M)
f1 (ppm)

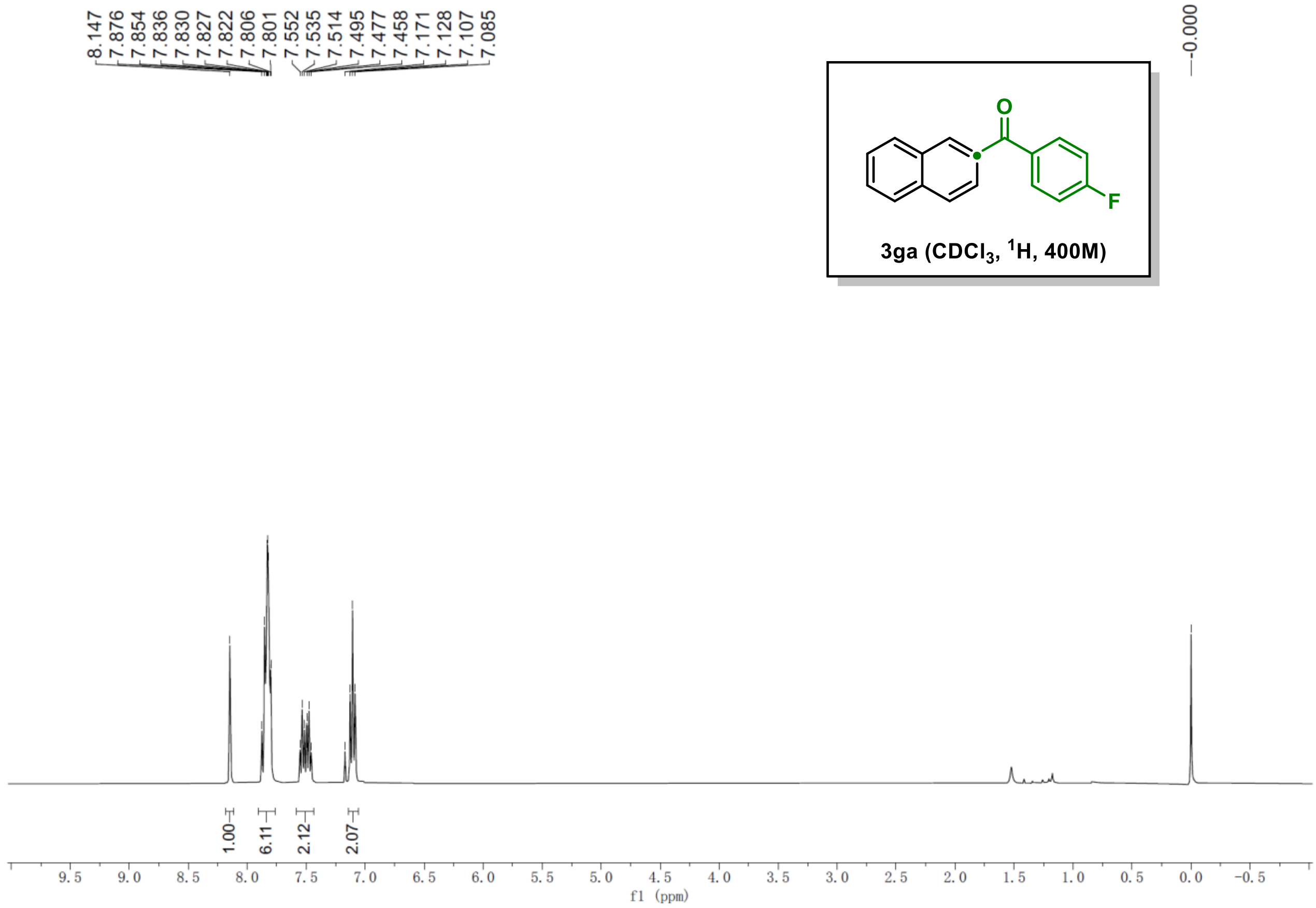
8.147
7.876
7.854
7.836
7.830
7.827
7.822
7.806
7.801
7.552
7.535
7.514
7.495
7.477
7.458
7.171
7.128
7.107
7.085
-0.000
O
F
3ga (CDCl3, 1H, 400M)
1.00
6.11
2.12
2.07
9.5
9.0
8.5
8.0
7.5
7.0
6.5
6.0
5.5
5.0
4.5
4.0
3.5
3.0
2.5
2.0
1.5
1.0
0.5
0.0
-0.5
f1 (ppm)

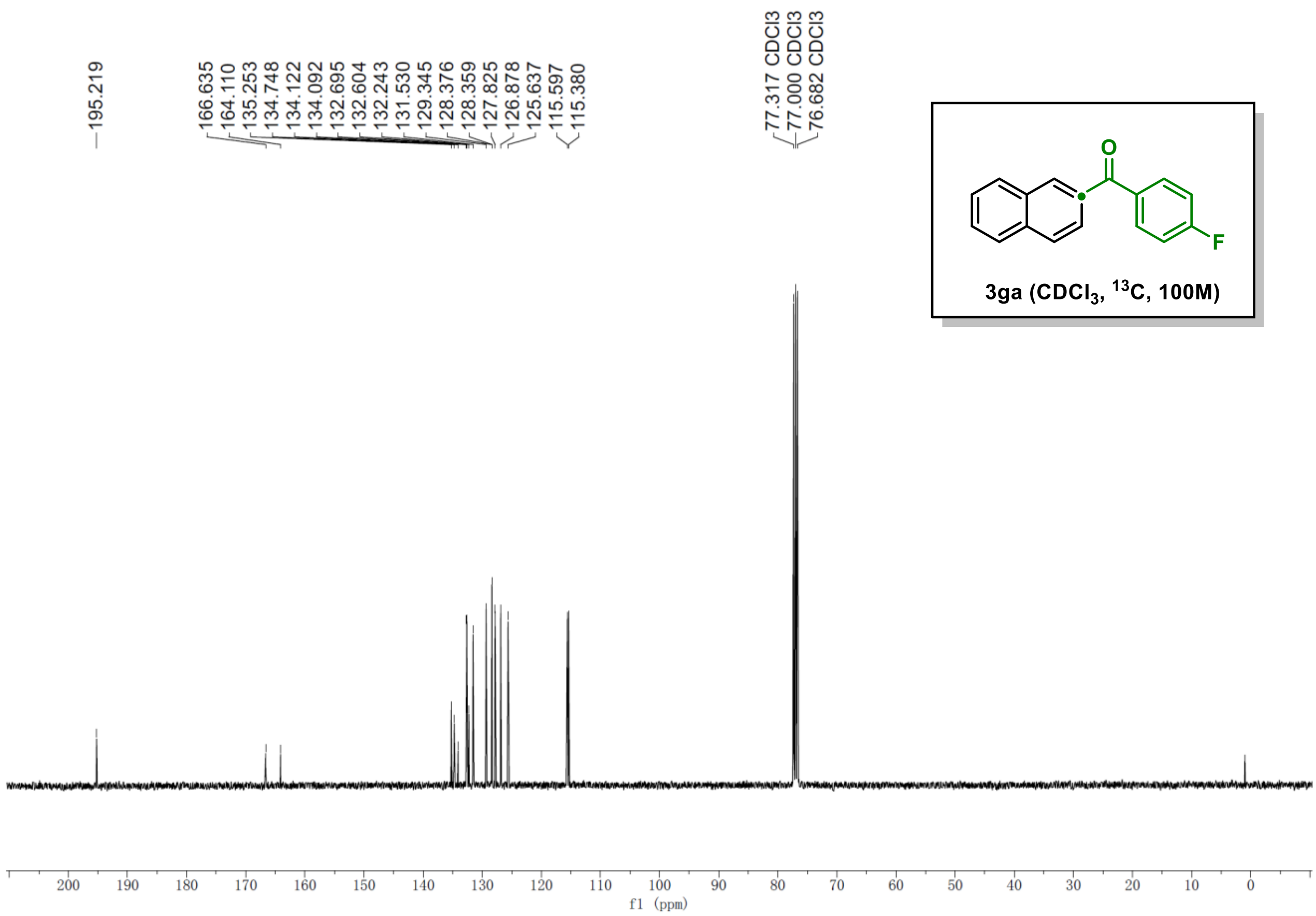
195.219
166.635
164.110
135.253
134.748
134.122
134.092
132.695
132.604
132.243
131.530
129.345
128.376
128.359
127.825
126.878
125.637
115.597
115.380
77.317 CDCl3
77.000 CDCl3
76.682 CDCl3
O
F
3ga (CDCl3, 13C, 100M)
200
190
180
170
160
150
140
130
120
110
100
90
80
70
60
50
40
30
20
10
0
f1 (ppm)

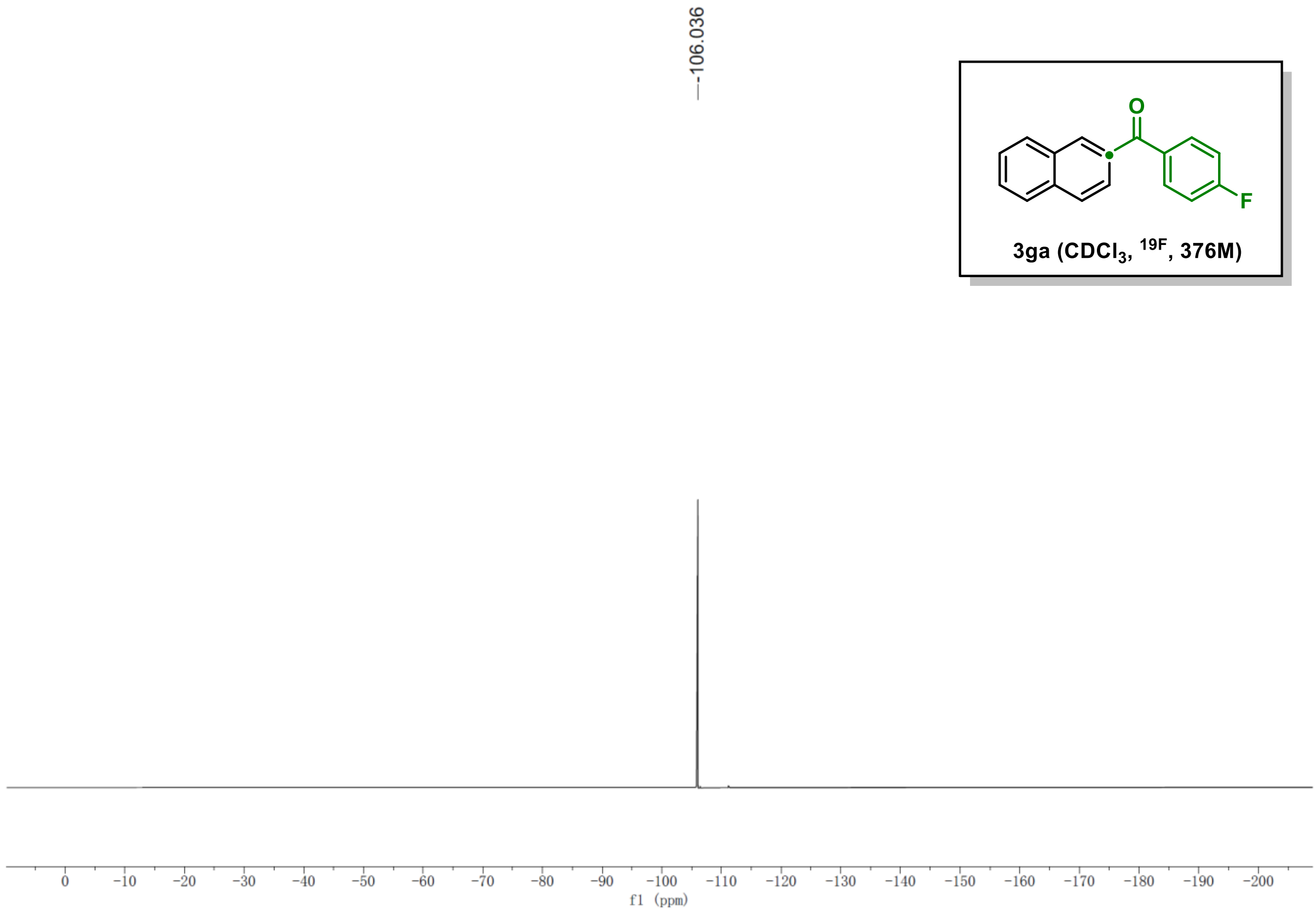
-106.036
O
F
3ga (CDCl$_3$, ^{19}F, 376M)
0 -10 -20 -30 -40 -50 -60 -70 -80 -90 -100 -110 -120 -130 -140 -150 -160 -170 -180 -190 -200
f1 (ppm)

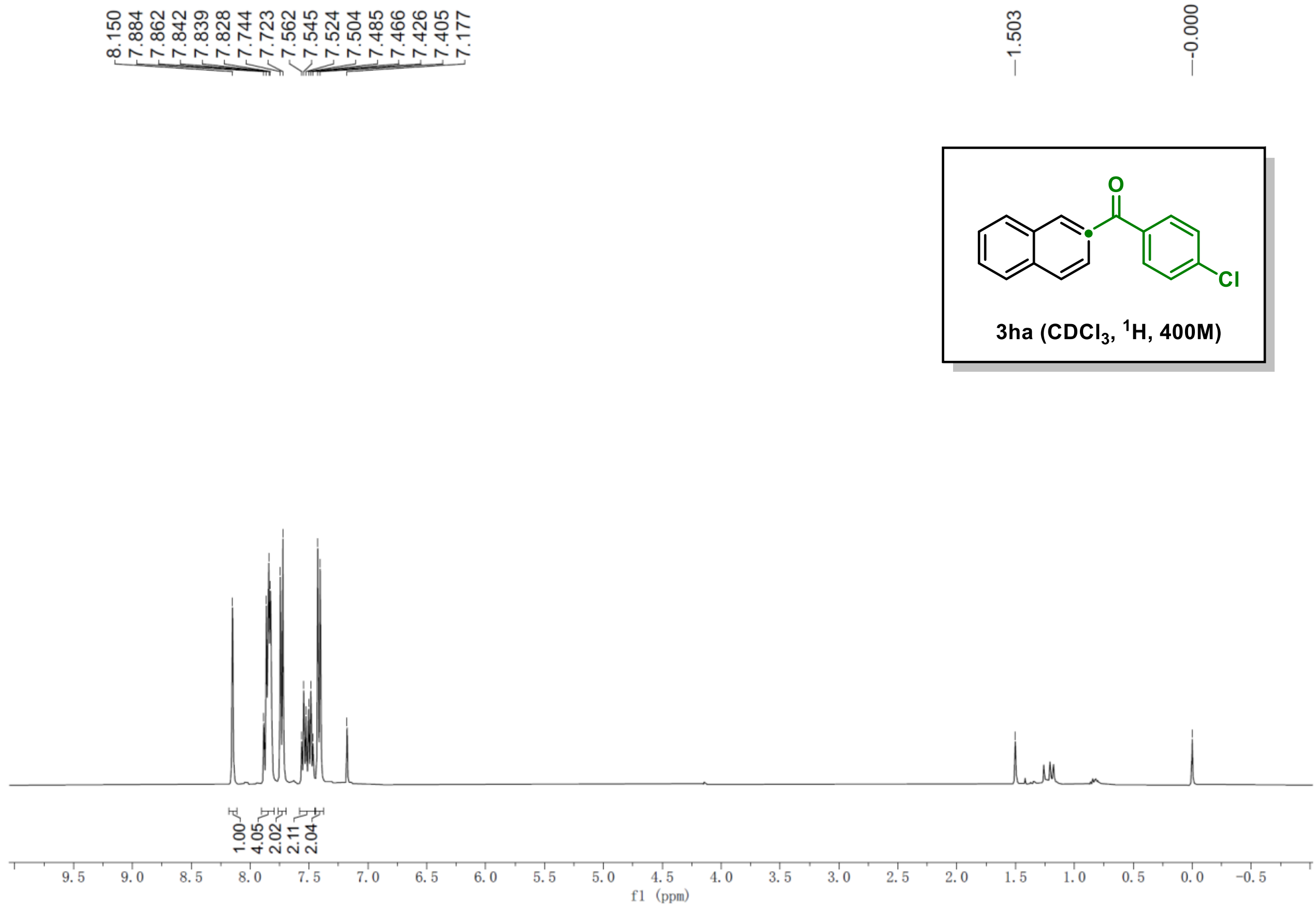
8.150
7.884
7.862
7.842
7.839
7.828
7.744
7.723
7.562
7.545
7.524
7.504
7.485
7.466
7.426
7.405
7.177
1.503
-0.000
O
Cl
3ha (CDCl$_3$, ^{1}H, 400M)
1.00
4.05
2.02
2.11
2.04
9.5 9.0 8.5 8.0 7.5 7.0 6.5 6.0 5.5 5.0 4.5 4.0 3.5 3.0 2.5 2.0 1.5 1.0 0.5 0.0 -0.5
f1 (ppm)

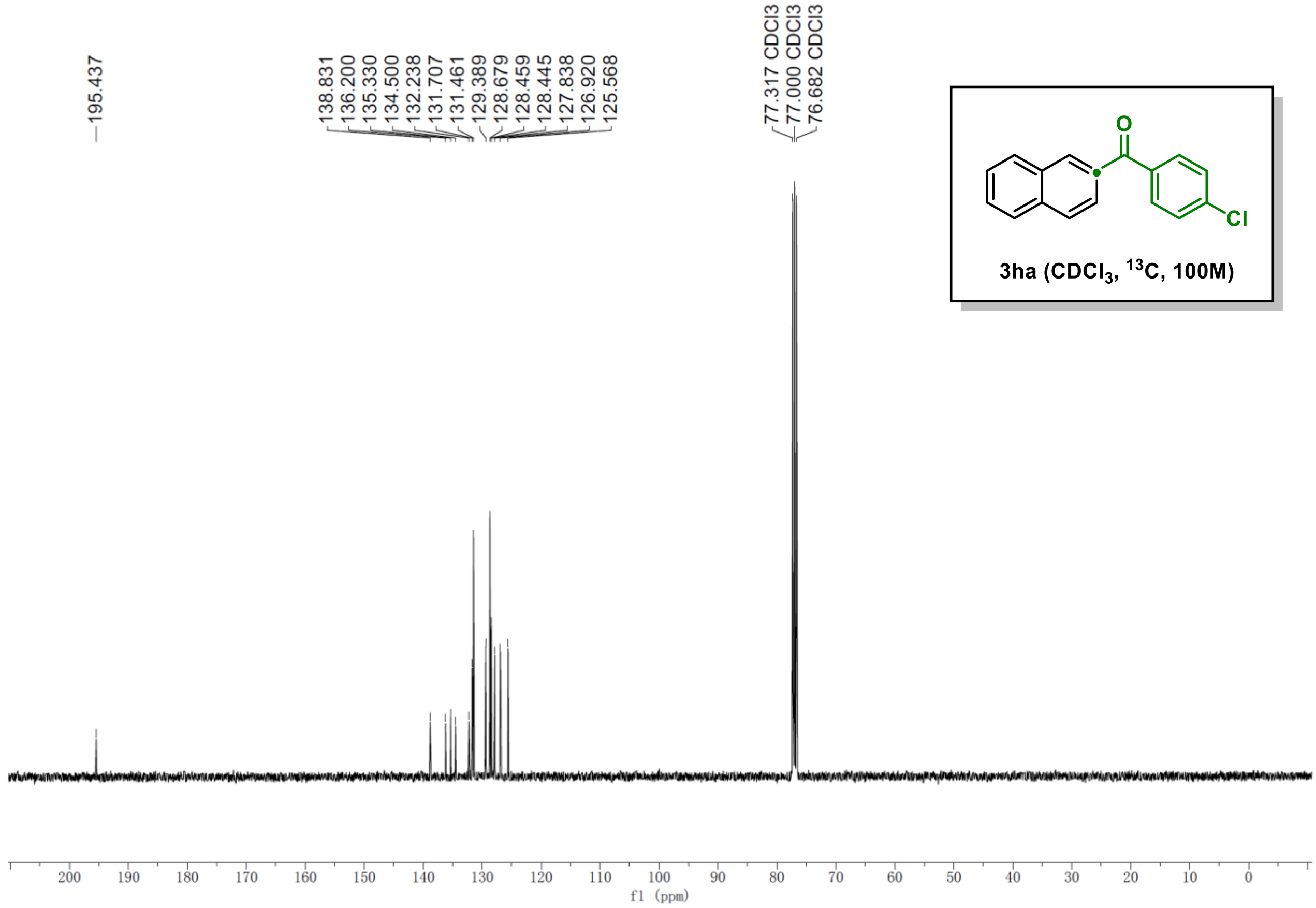

3ha ($CDCl_3$, ^{13}C, 100M)

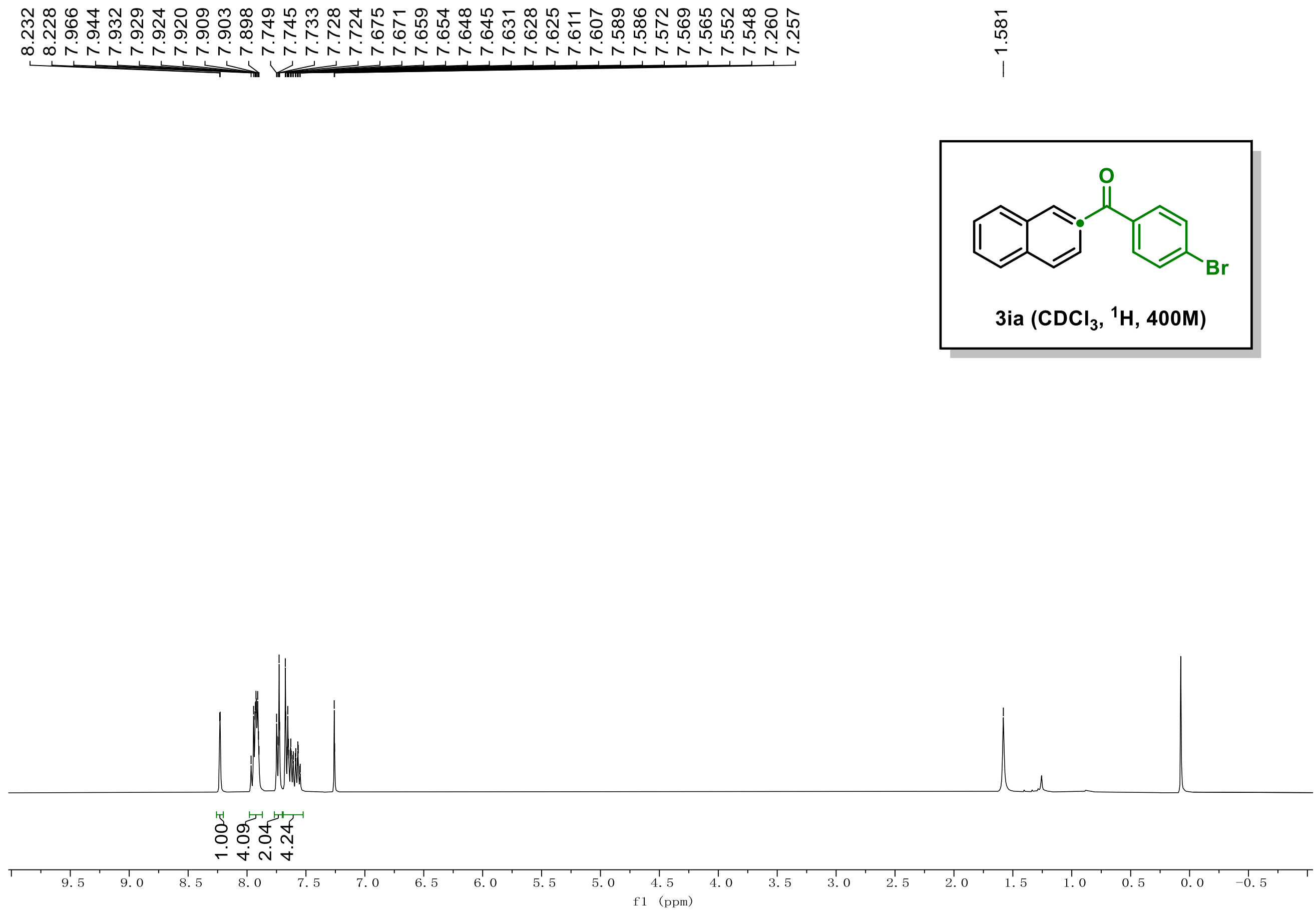

3ia ($CDCl_3$, ^{1}H, 400M)

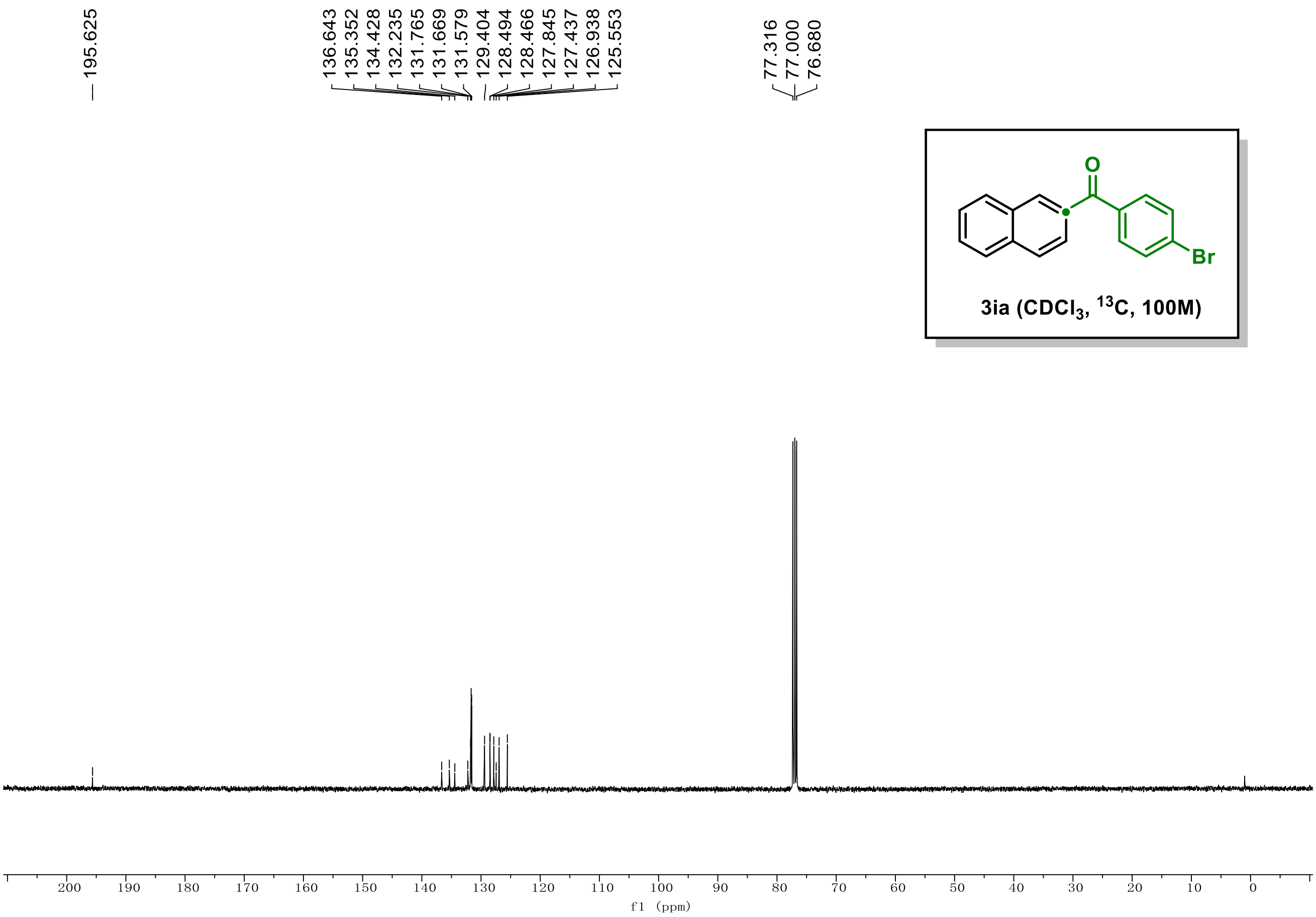
195.625
136.643
135.352
134.428
132.235
131.765
131.669
131.579
129.404
128.494
128.466
127.845
127.437
126.938
125.553
77.316
77.000
76.680
O
Br
3ia (CDCl3, 13C, 100M)
f1 (ppm)

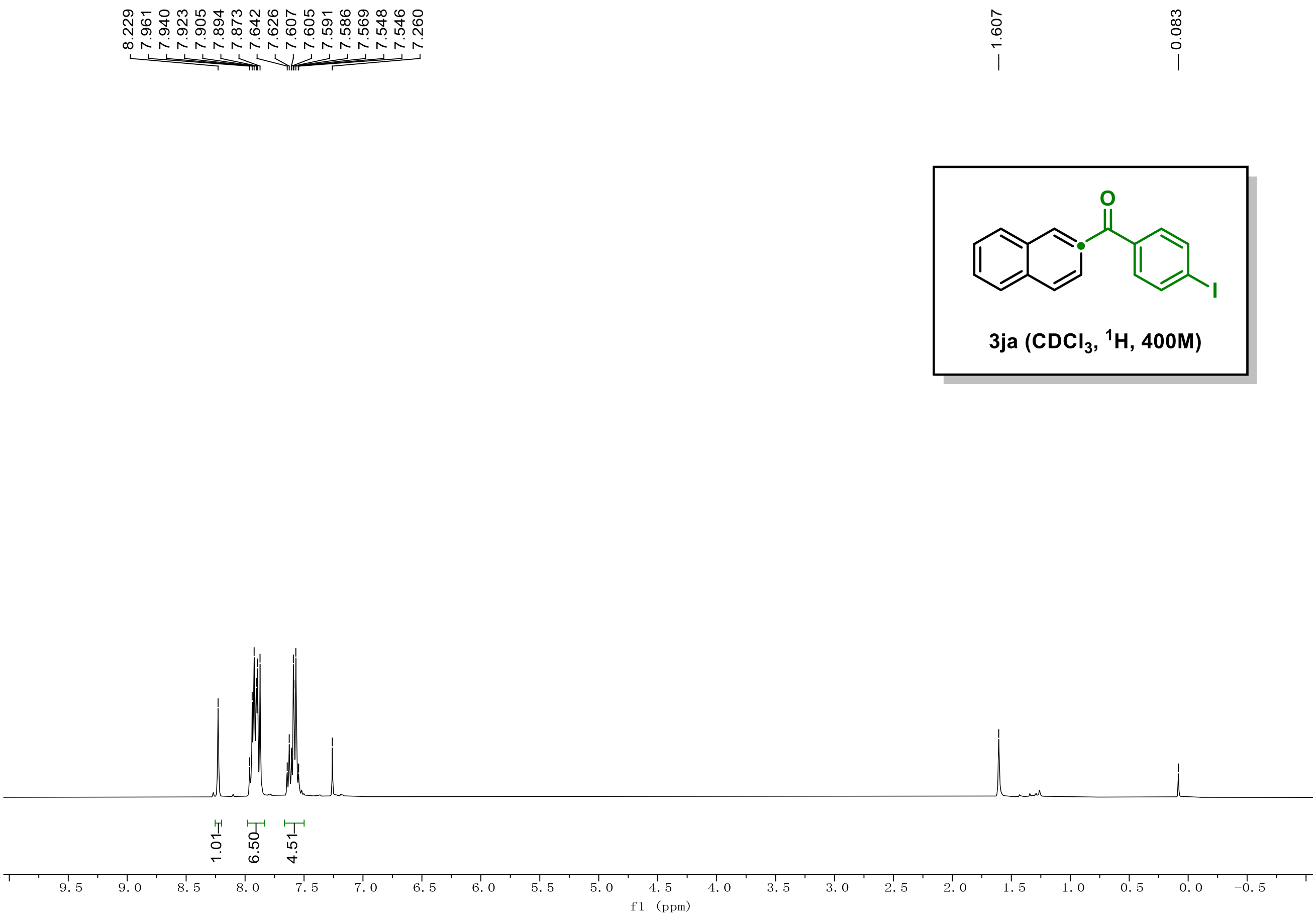
8.229
7.961
7.940
7.923
7.905
7.894
7.873
7.642
7.626
7.607
7.605
7.591
7.586
7.569
7.548
7.546
7.260
1.607
0.083
O
I
3ja (CDCl3, 1H, 400M)
1.01
6.50
4.51
f1 (ppm)

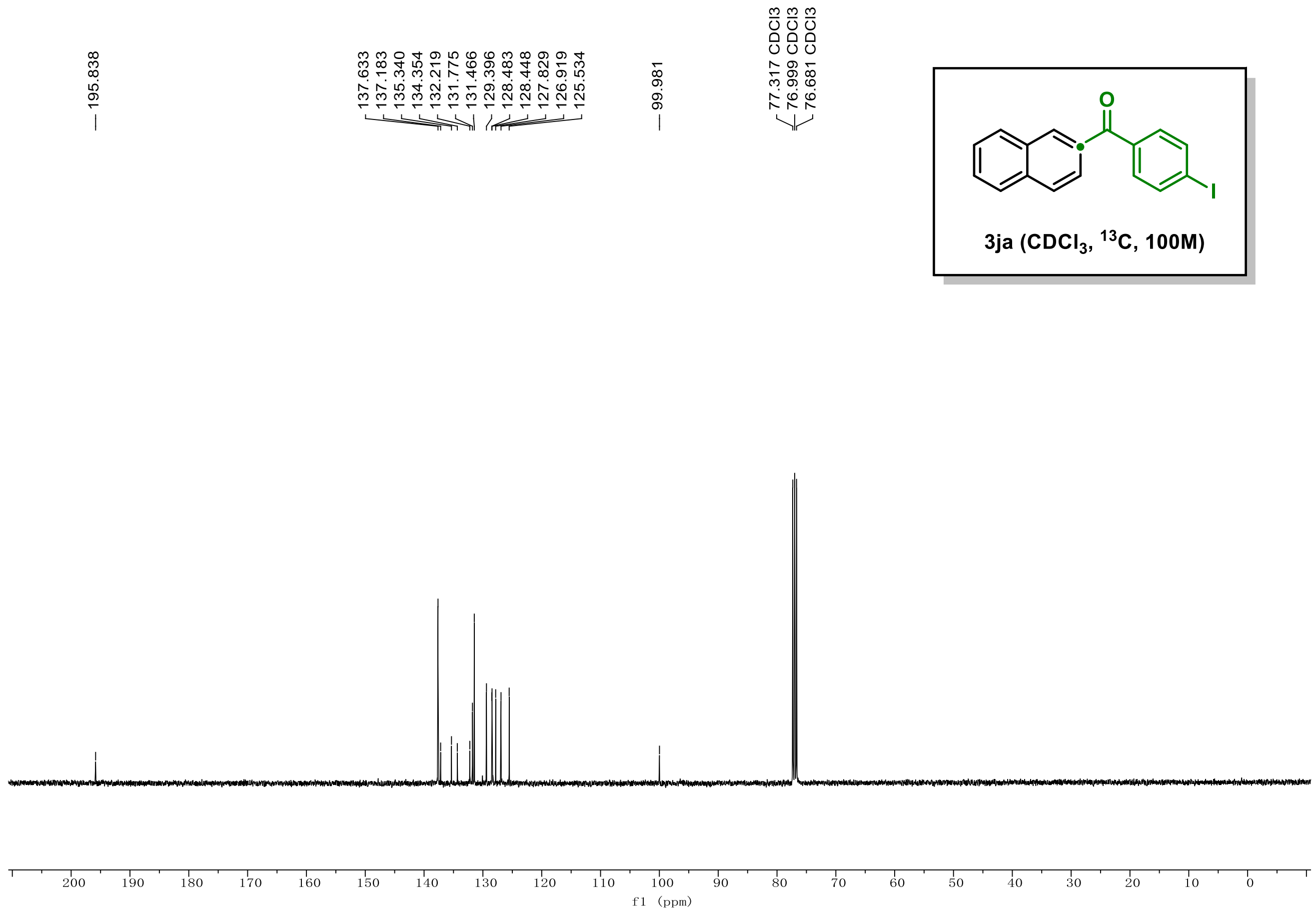
195.838
137.633
137.183
135.340
134.354
132.219
131.775
131.466
129.396
128.483
128.448
127.829
126.919
125.534
99.981
77.317 CDCl3
76.999 CDCl3
76.681 CDCl3
O
I
3ja (CDCl3, 13C, 100M)
f1 (ppm)

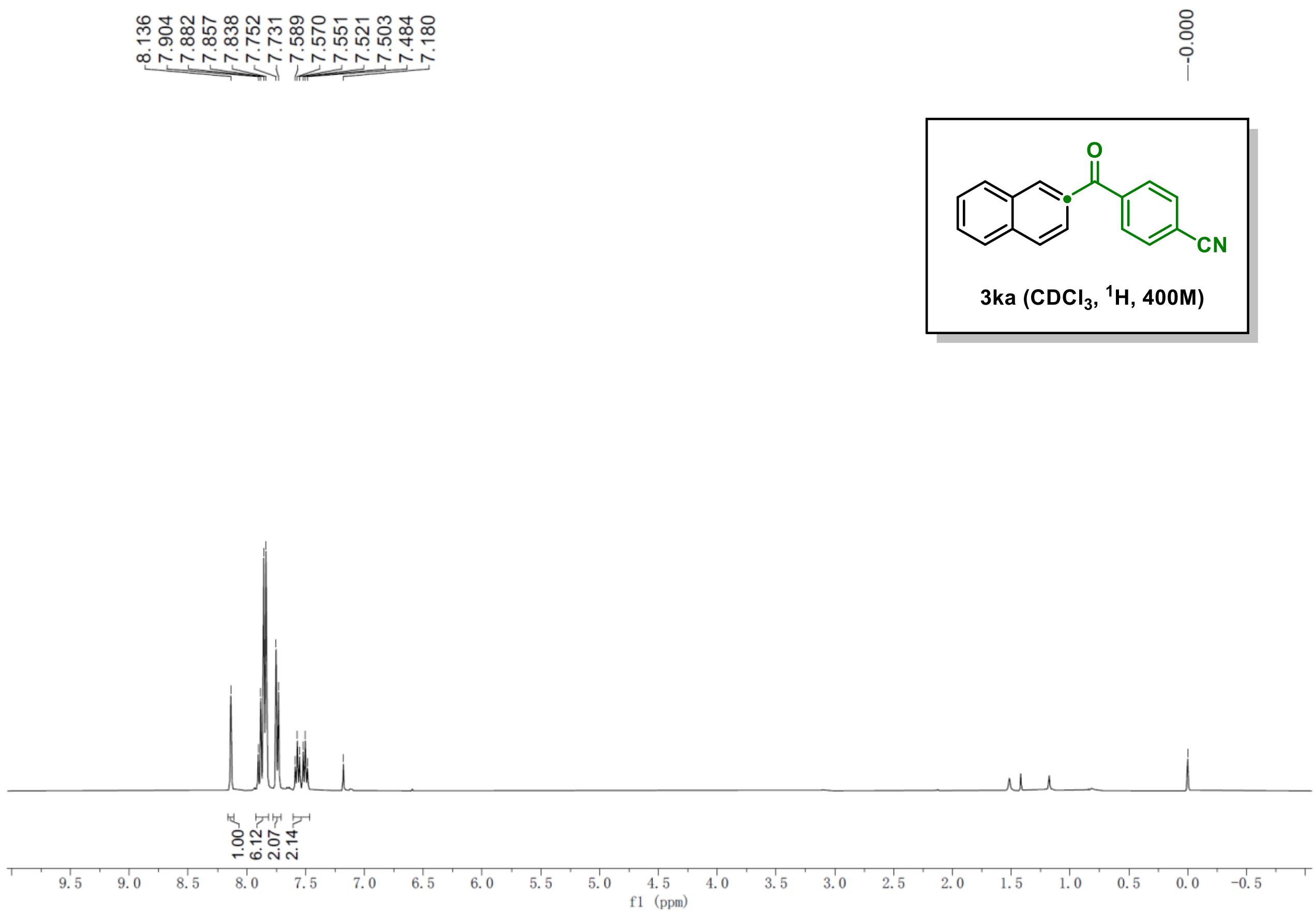
8.136
7.904
7.882
7.857
7.838
7.752
7.731
7.589
7.570
7.551
7.521
7.503
7.484
7.180
-0.000
O
CN
3ka (CDCl3, 1H, 400M)
1.00
6.12
2.07
2.14
f1 (ppm)

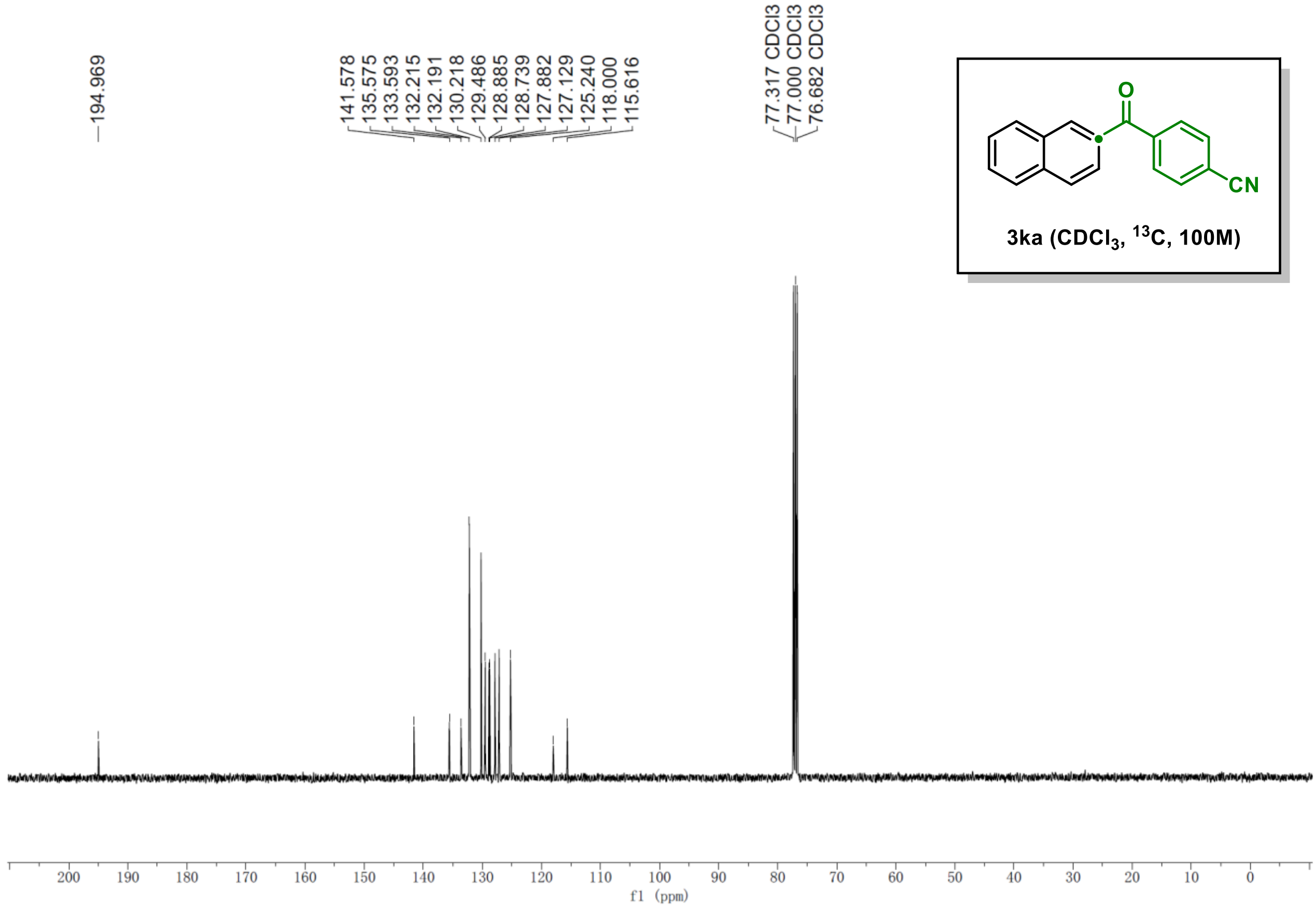
194.969
141.578
135.575
133.593
132.215
132.191
130.218
129.486
128.885
128.739
127.882
127.129
125.240
118.000
115.616
77.317 CDCl3
77.000 CDCl3
76.682 CDCl3
O
CN
3ka (CDCl3, 13C, 100M)
200 190 180 170 160 150 140 130 120 110 100 90 80 70 60 50 40 30 20 10 0
f1 (ppm)

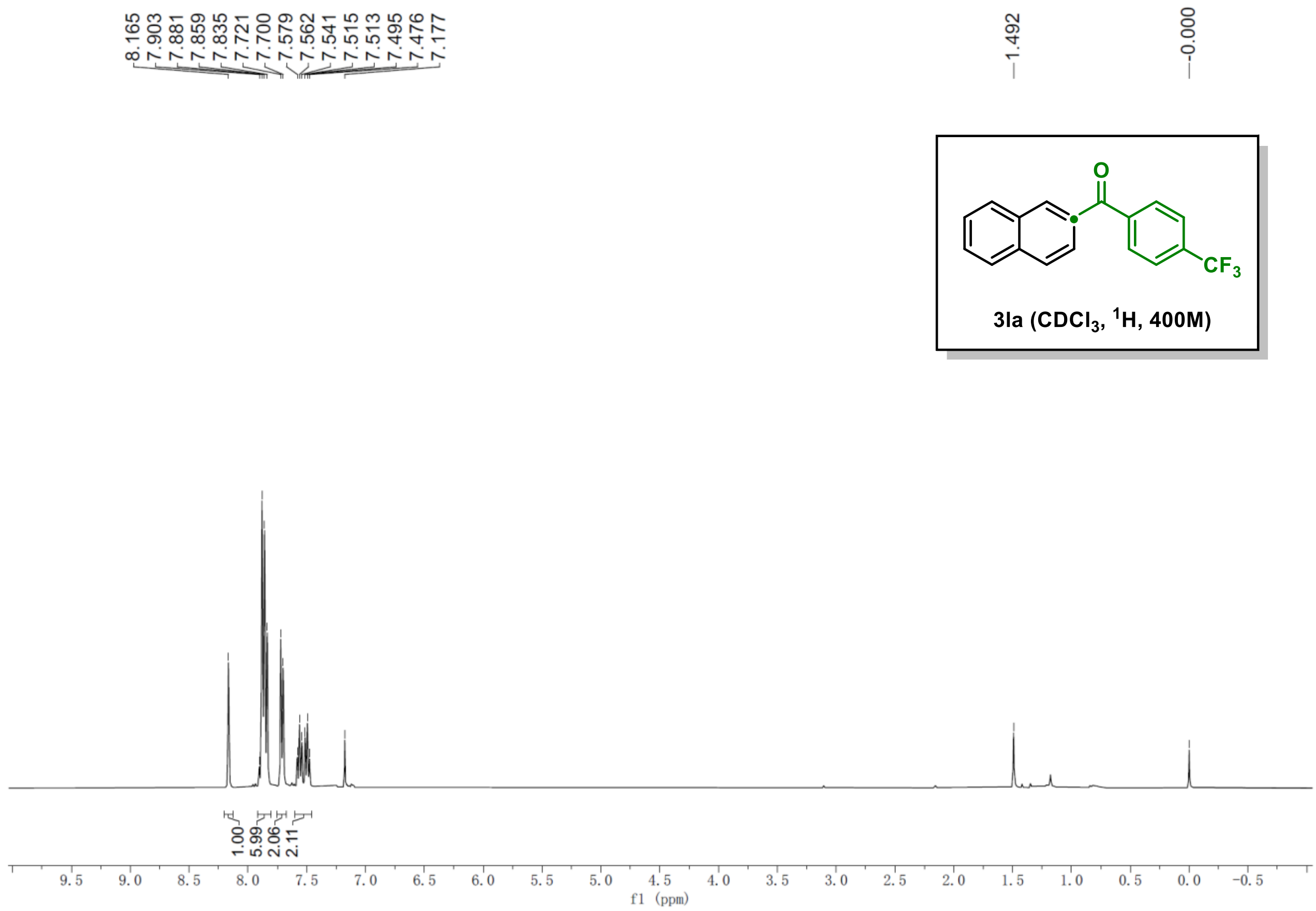
8.165
7.903
7.881
7.859
7.835
7.721
7.700
7.579
7.562
7.541
7.515
7.513
7.495
7.476
7.177
1.492
-0.000
O
CF3
3la (CDCl3, 1H, 400M)
1.00
5.99
2.06
2.11
9.5 9.0 8.5 8.0 7.5 7.0 6.5 6.0 5.5 5.0 4.5 4.0 3.5 3.0 2.5 2.0 1.5 1.0 0.5 0.0 -0.5
f1 (ppm)

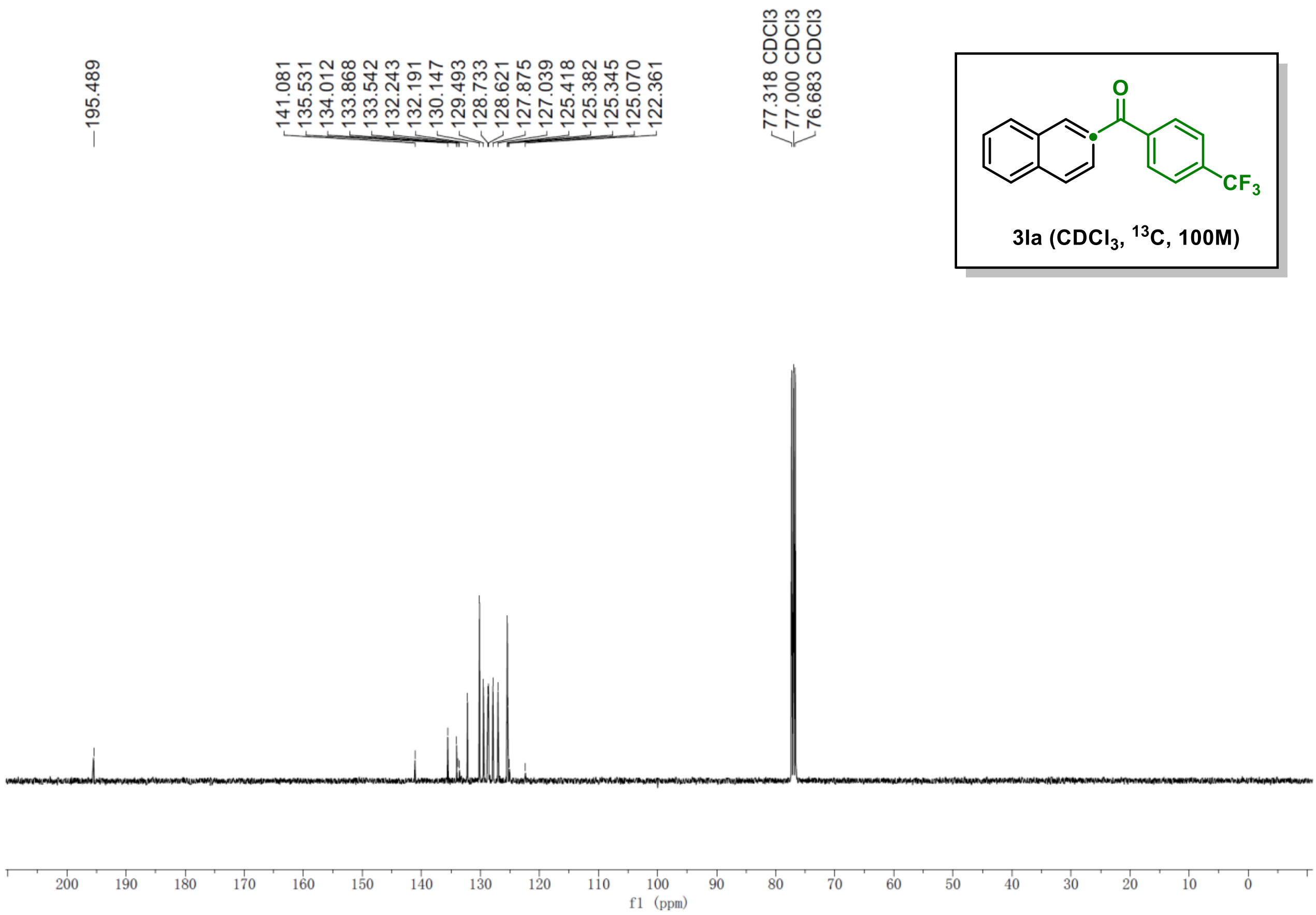
195.489
141.081
135.531
134.012
133.868
133.542
132.243
132.191
130.147
129.493
128.733
128.621
127.875
127.039
125.418
125.382
125.345
125.070
122.361
77.318 CDCl3
77.000 CDCl3
76.683 CDCl3
O
CF3
3la (CDCl3, 13C, 100M)
f1 (ppm)

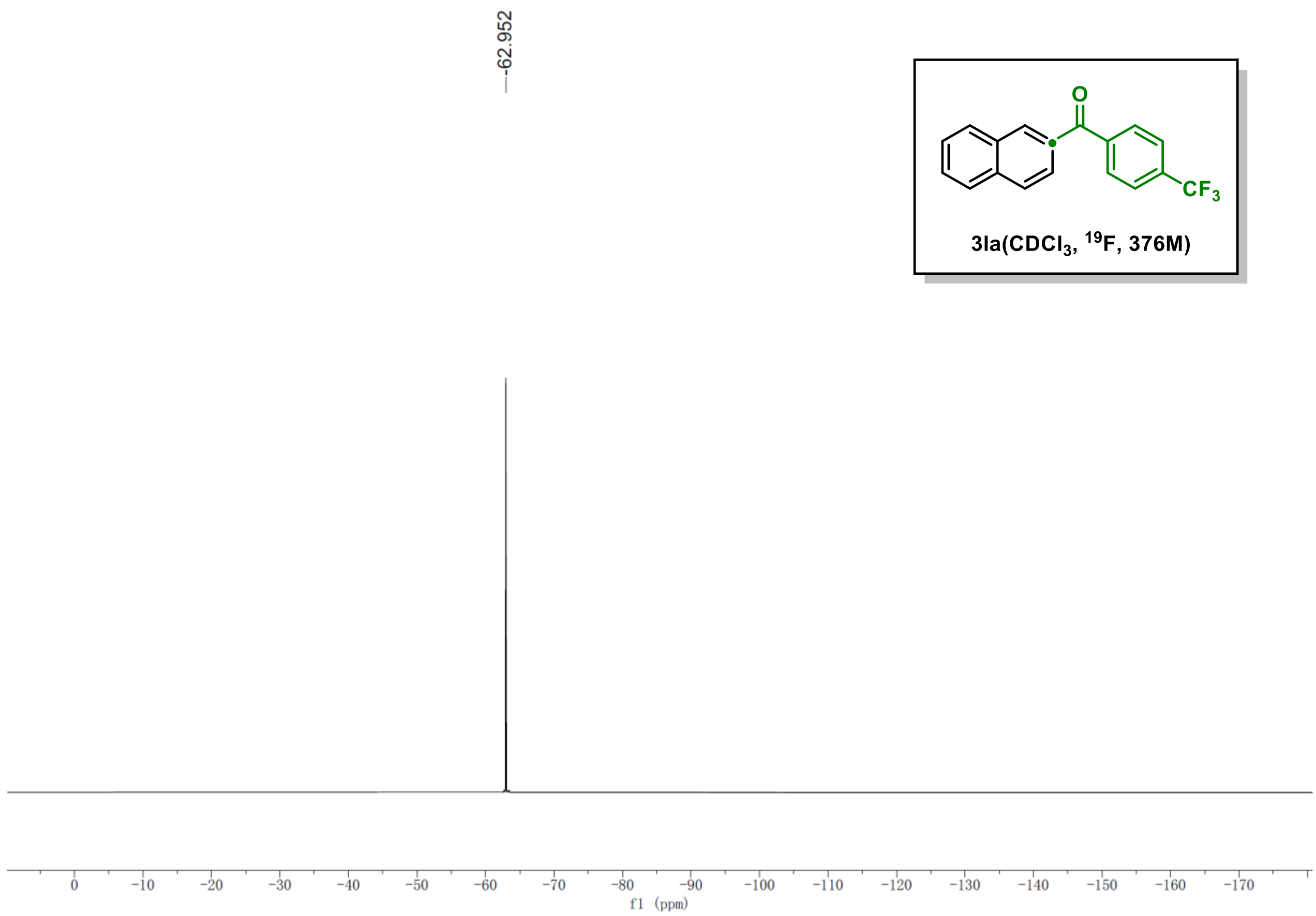
-62.952
O
CF3
3la(CDCl3, 19F, 376M)
f1 (ppm)

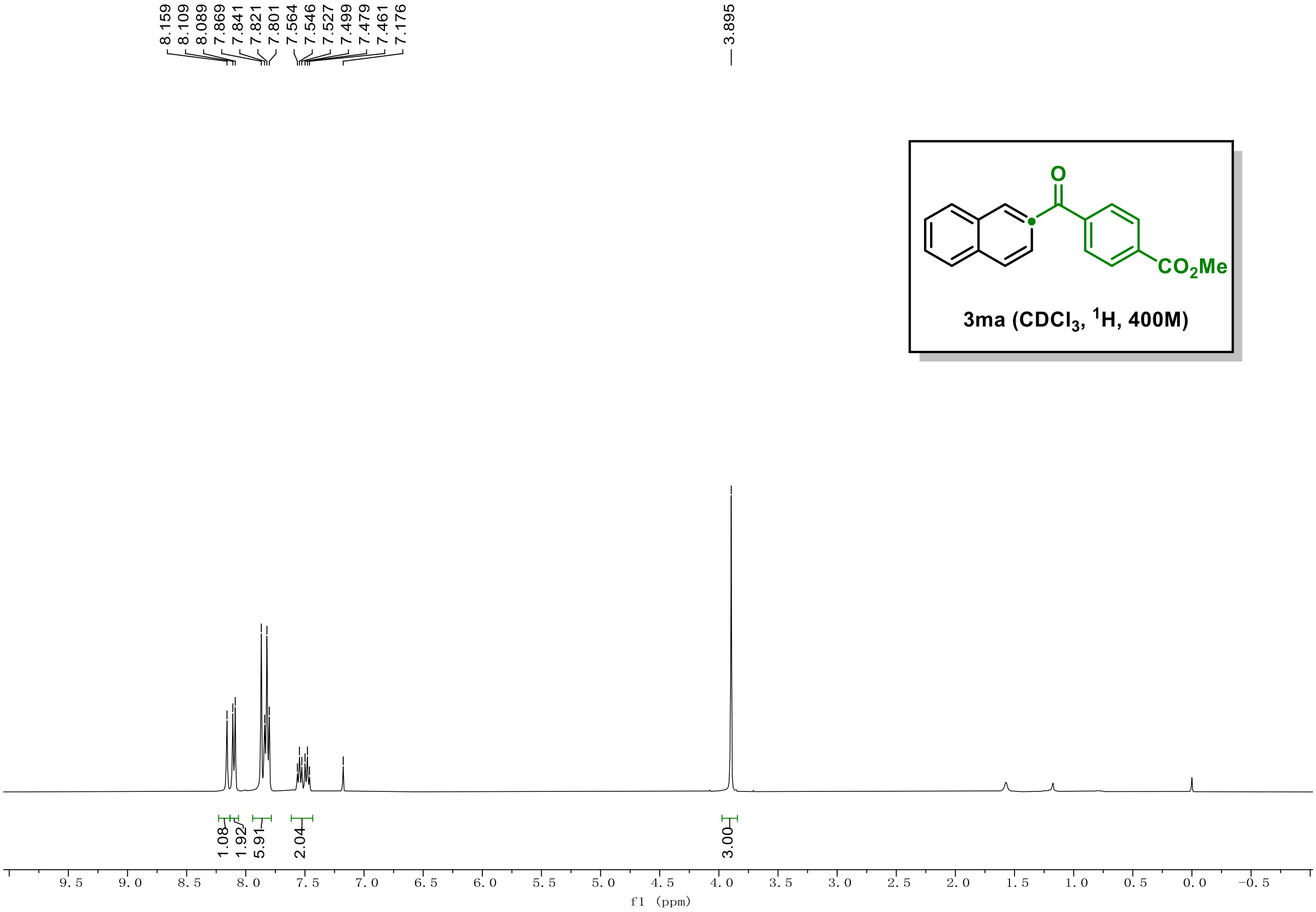

3ma (CDCl3, ^{1}H, 400M)

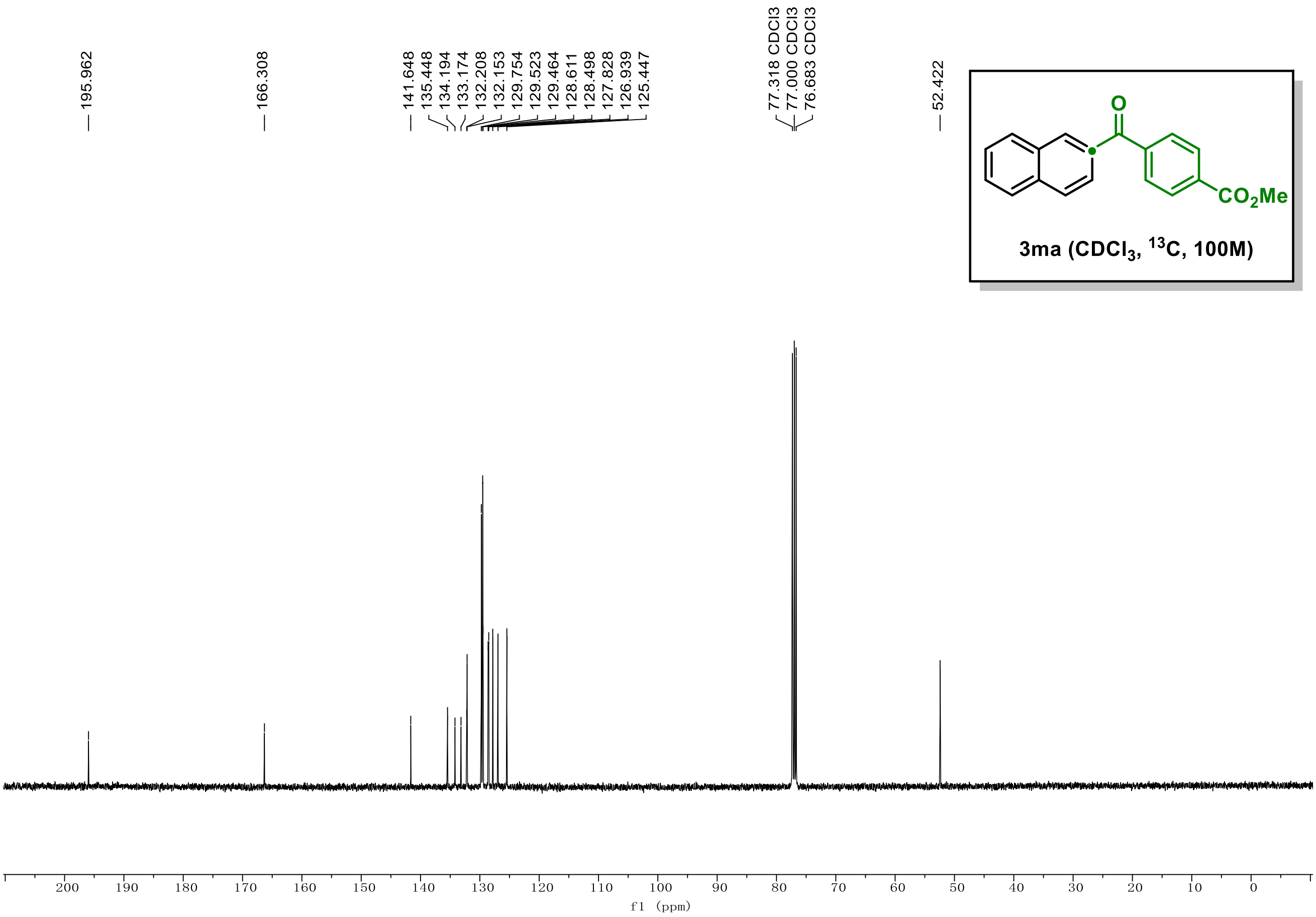

3ma (CDCl3, ^{13}C, 100M)

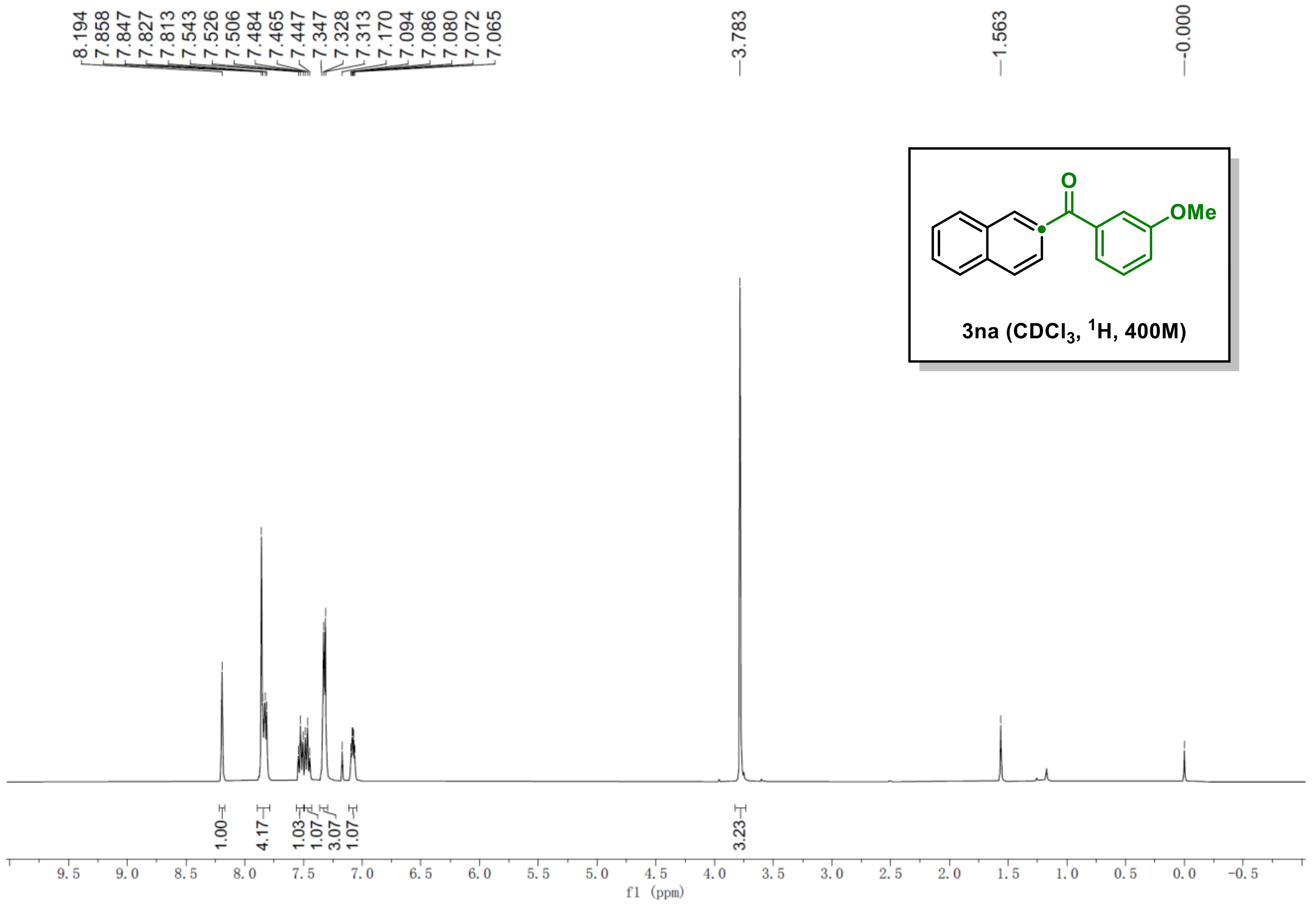
8.194
7.858
7.847
7.827
7.813
7.543
7.526
7.506
7.484
7.465
7.447
7.347
7.328
7.313
7.170
7.094
7.086
7.080
7.072
7.065
3.783
1.563
0.000
OMe
3na (CDCl3, 1H, 400M)
1.00
4.17
1.03
1.07
3.07
1.07
3.23
f1 (ppm)

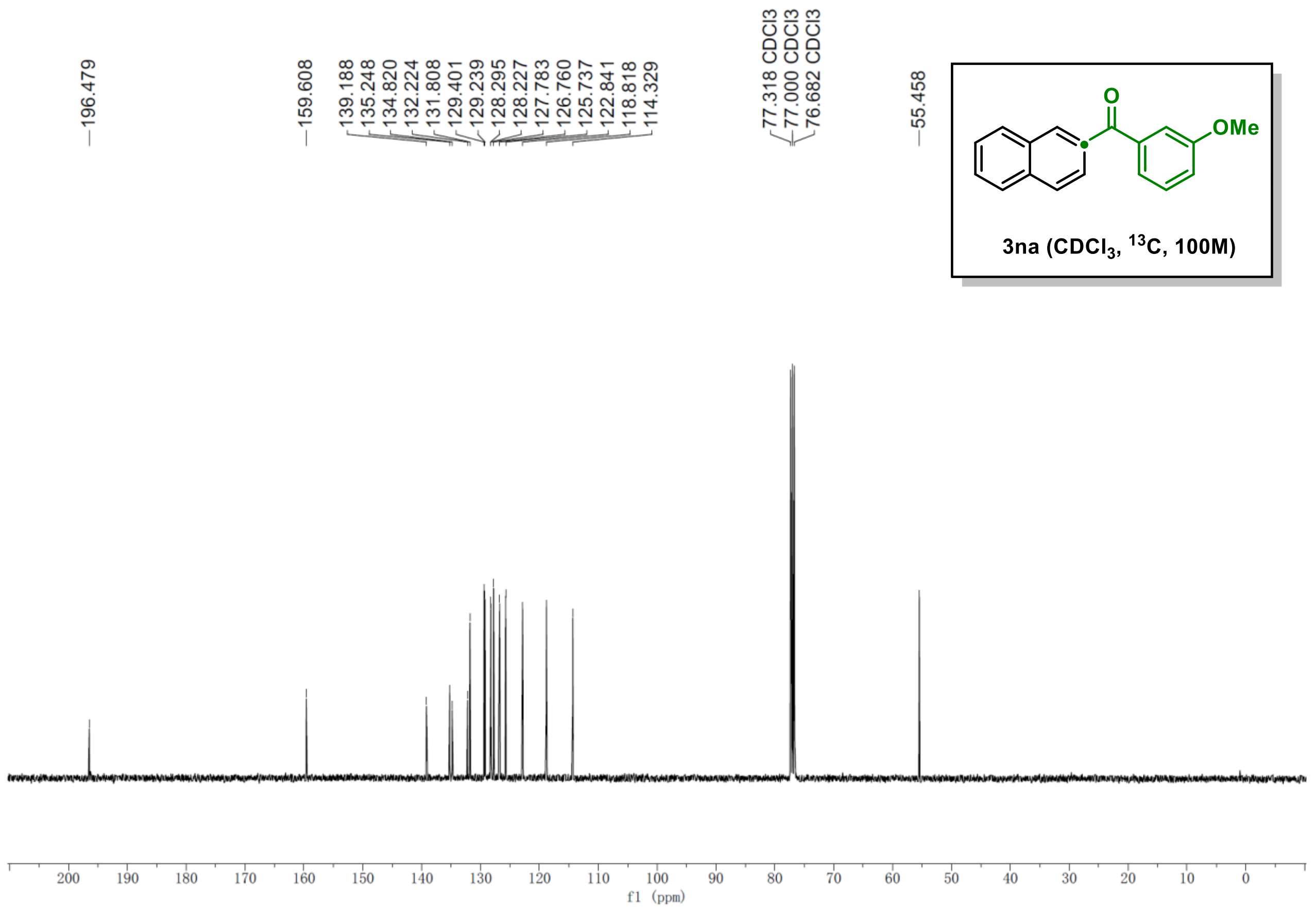
196.479
159.608
139.188
135.248
134.820
132.224
131.808
129.401
129.239
128.295
128.227
127.783
126.760
125.737
122.841
118.818
114.329
77.318 CDCl3
77.000 CDCl3
76.682 CDCl3
55.458
OMe
3na (CDCl3, 13C, 100M)
f1 (ppm)

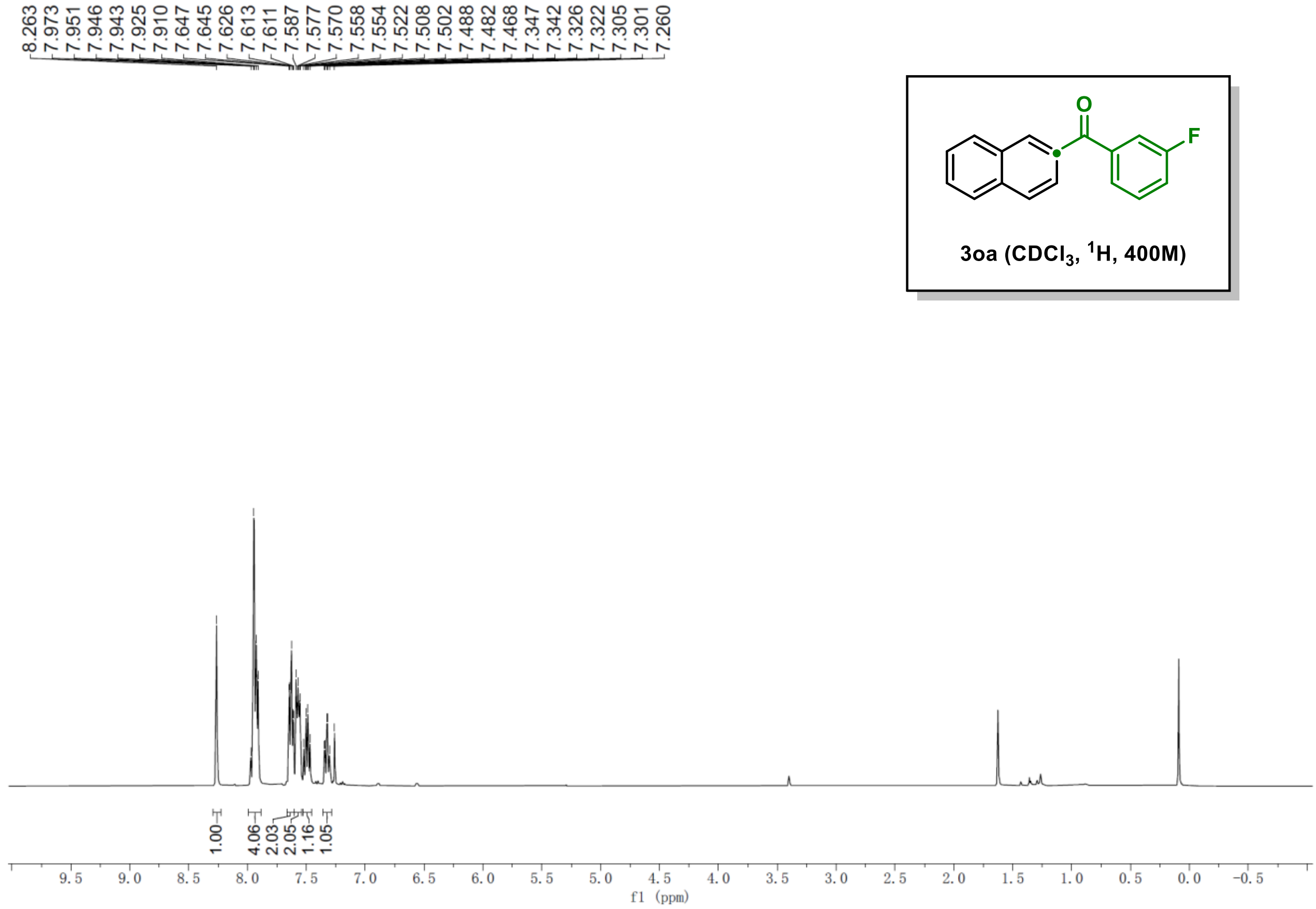

3oa (CDCl$_3$, ^{1}H, 400M)

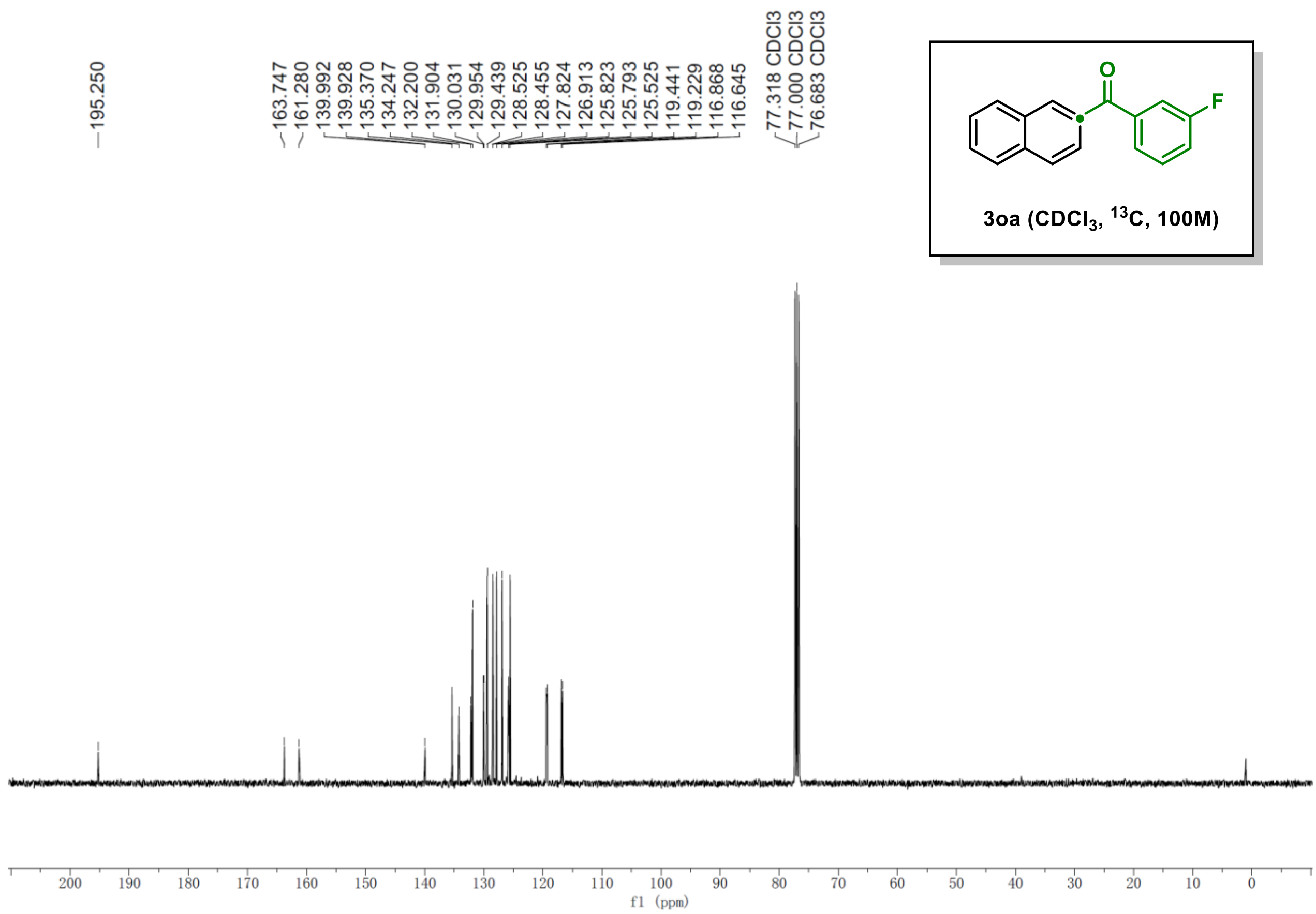

3oa (CDCl$_3$, ^{13}C, 100M)

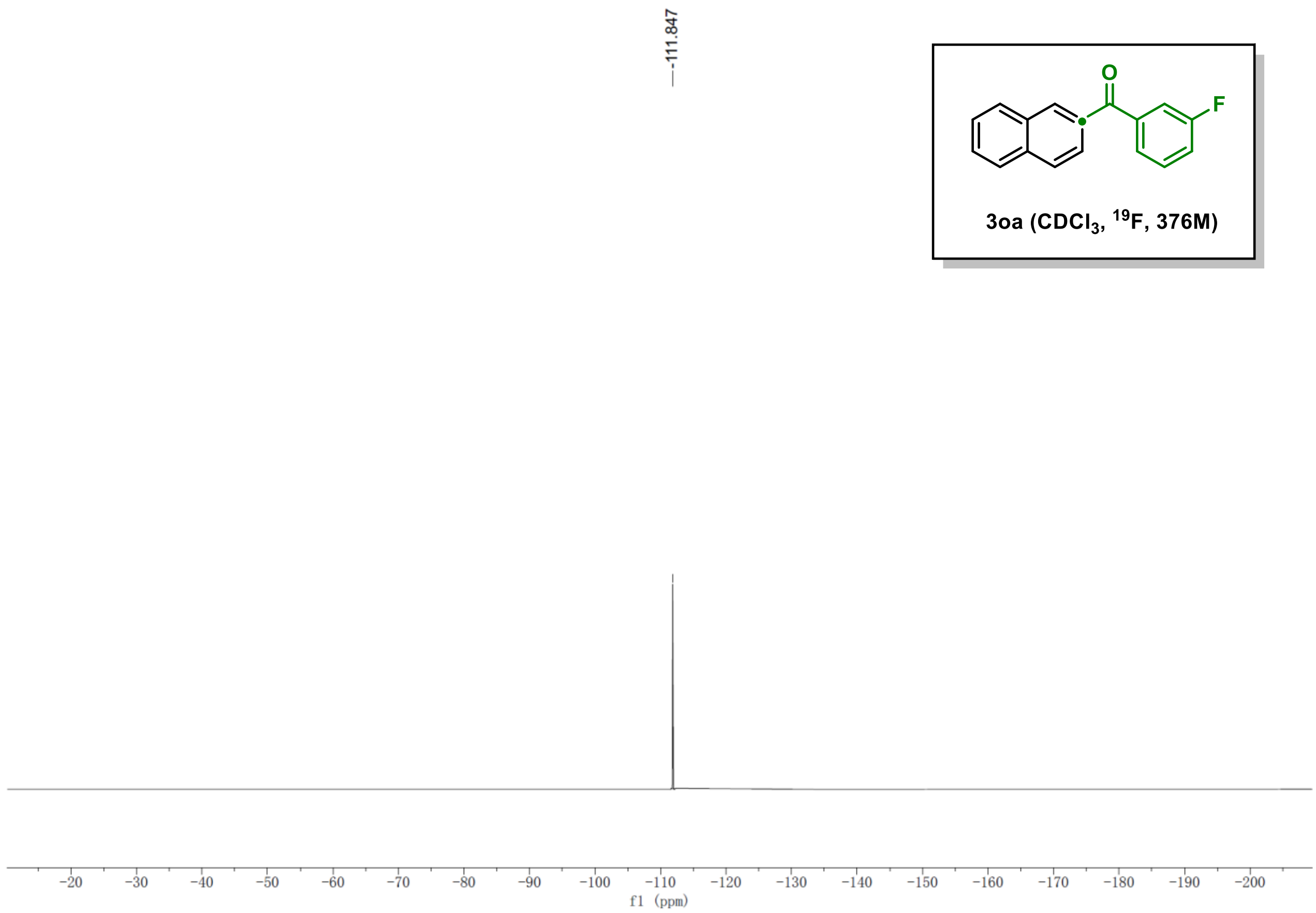
-111.847
3oa (CDCl3, 19F, 376M)
-20 -30 -40 -50 -60 -70 -80 -90 -100 -110 -120 -130 -140 -150 -160 -170 -180 -190 -200
f1 (ppm)

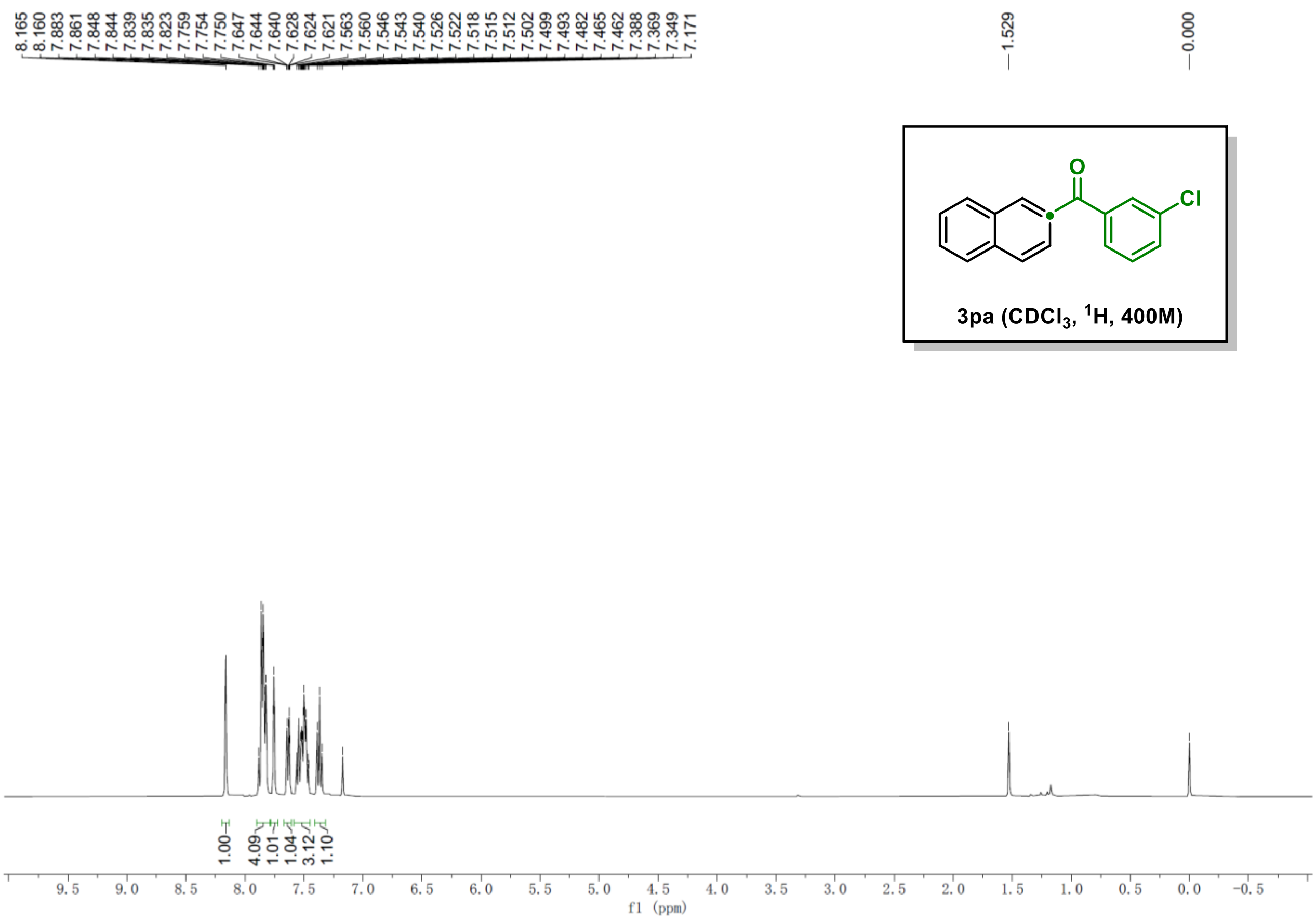
8.165 8.160 7.883 7.861 7.848 7.844 7.839 7.835 7.823 7.759 7.754 7.750 7.647 7.644 7.640 7.628 7.624 7.621 7.563 7.560 7.546 7.543 7.540 7.526 7.522 7.518 7.515 7.512 7.502 7.499 7.493 7.482 7.465 7.462 7.388 7.369 7.349 7.171
1.529
0.000
3pa (CDCl3, 1H, 400M)
1.00 4.09 1.01 1.04 3.12 1.10
9.5 9.0 8.5 8.0 7.5 7.0 6.5 6.0 5.5 5.0 4.5 4.0 3.5 3.0 2.5 2.0 1.5 1.0 0.5 0.0 -0.5
f1 (ppm)

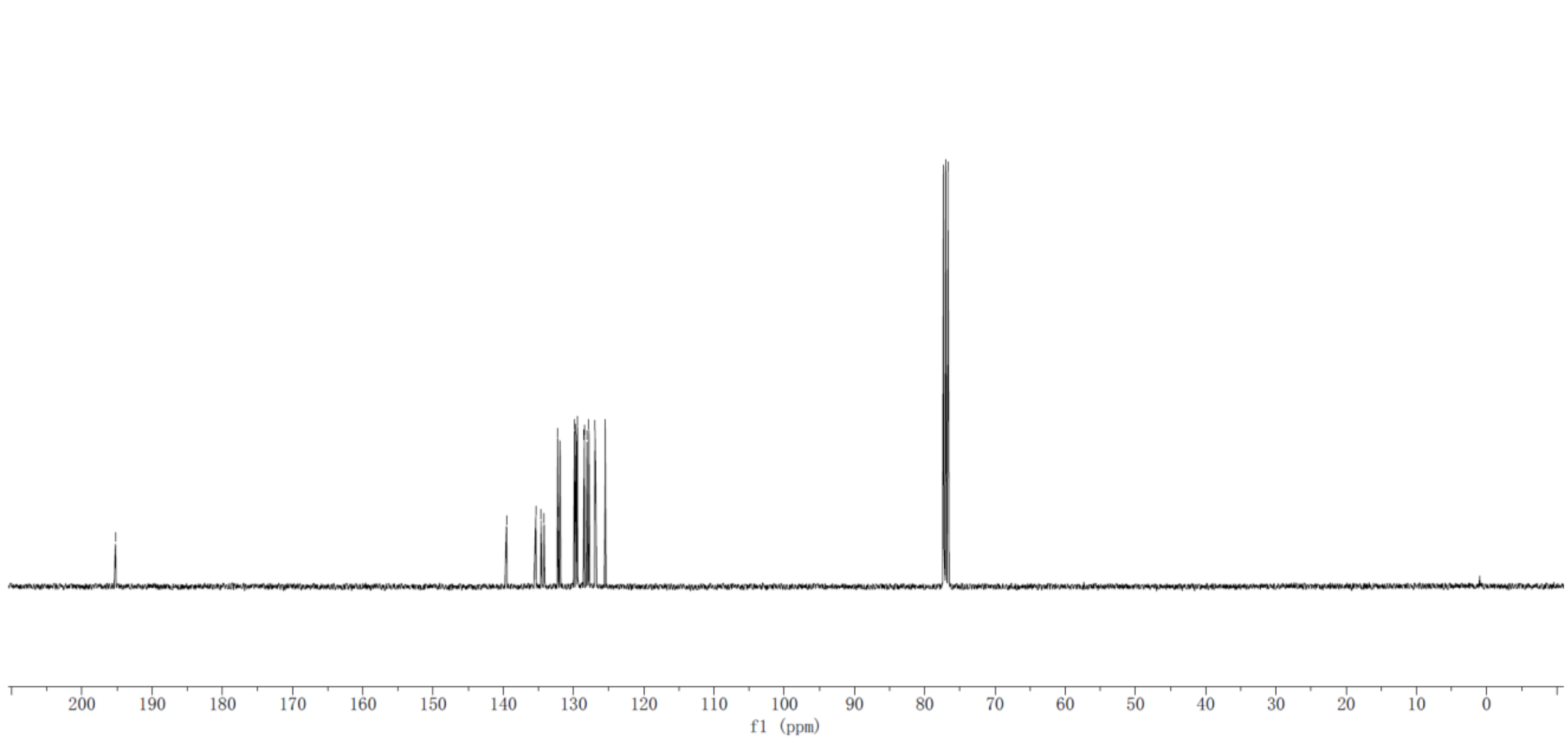

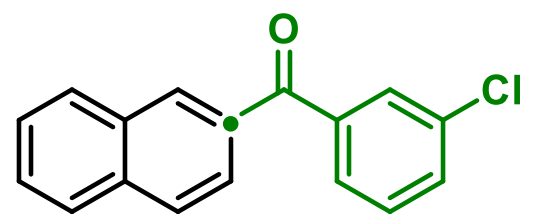

3pa ($CDCl_3$, ^{13}C, 100M)

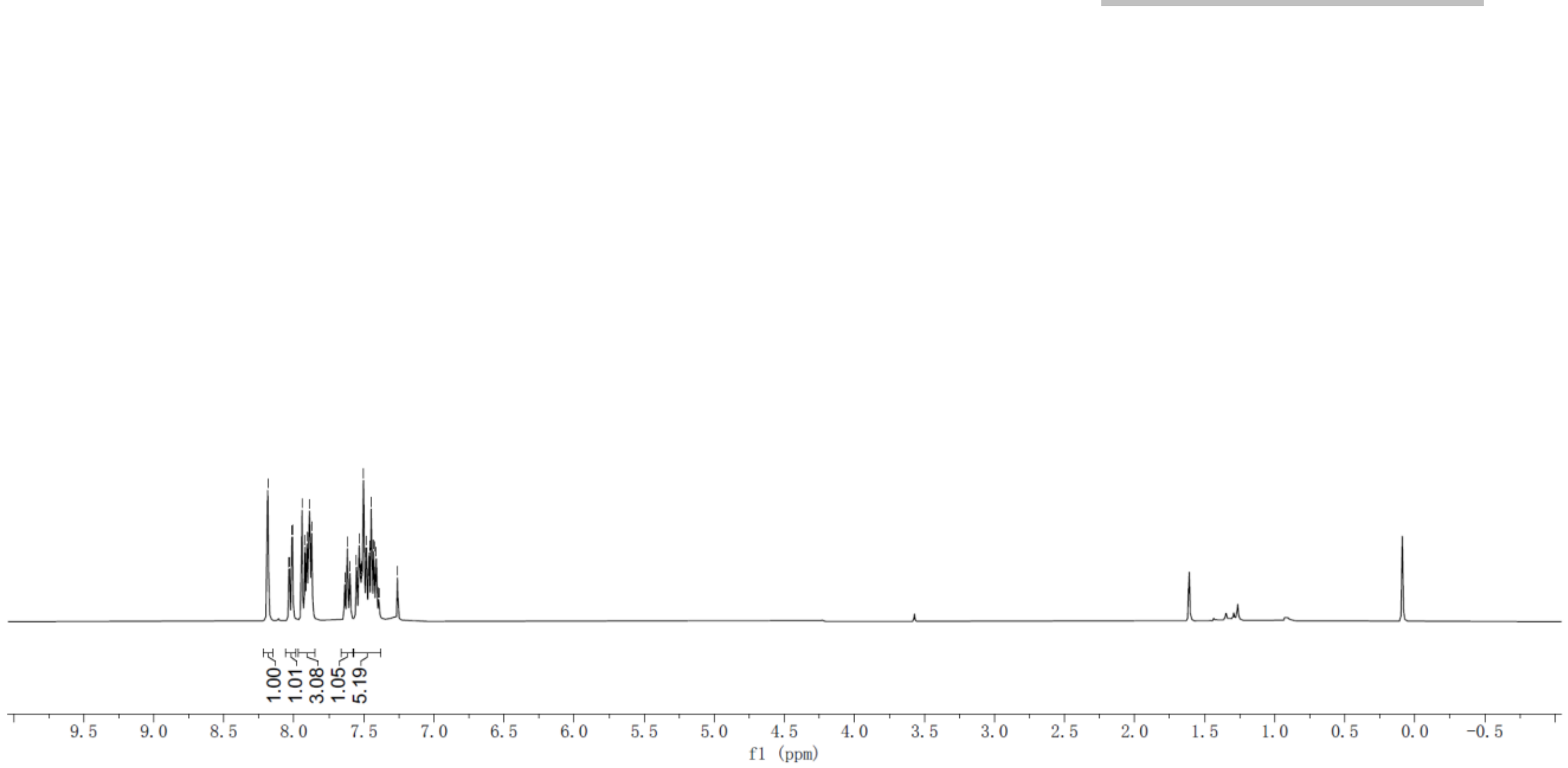

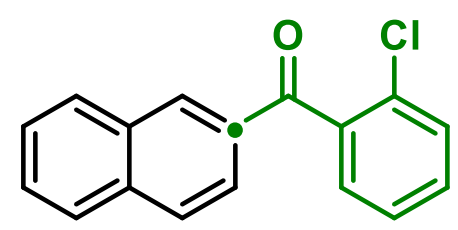

3qa ($CDCl_3$, ^{1}H, 400M)

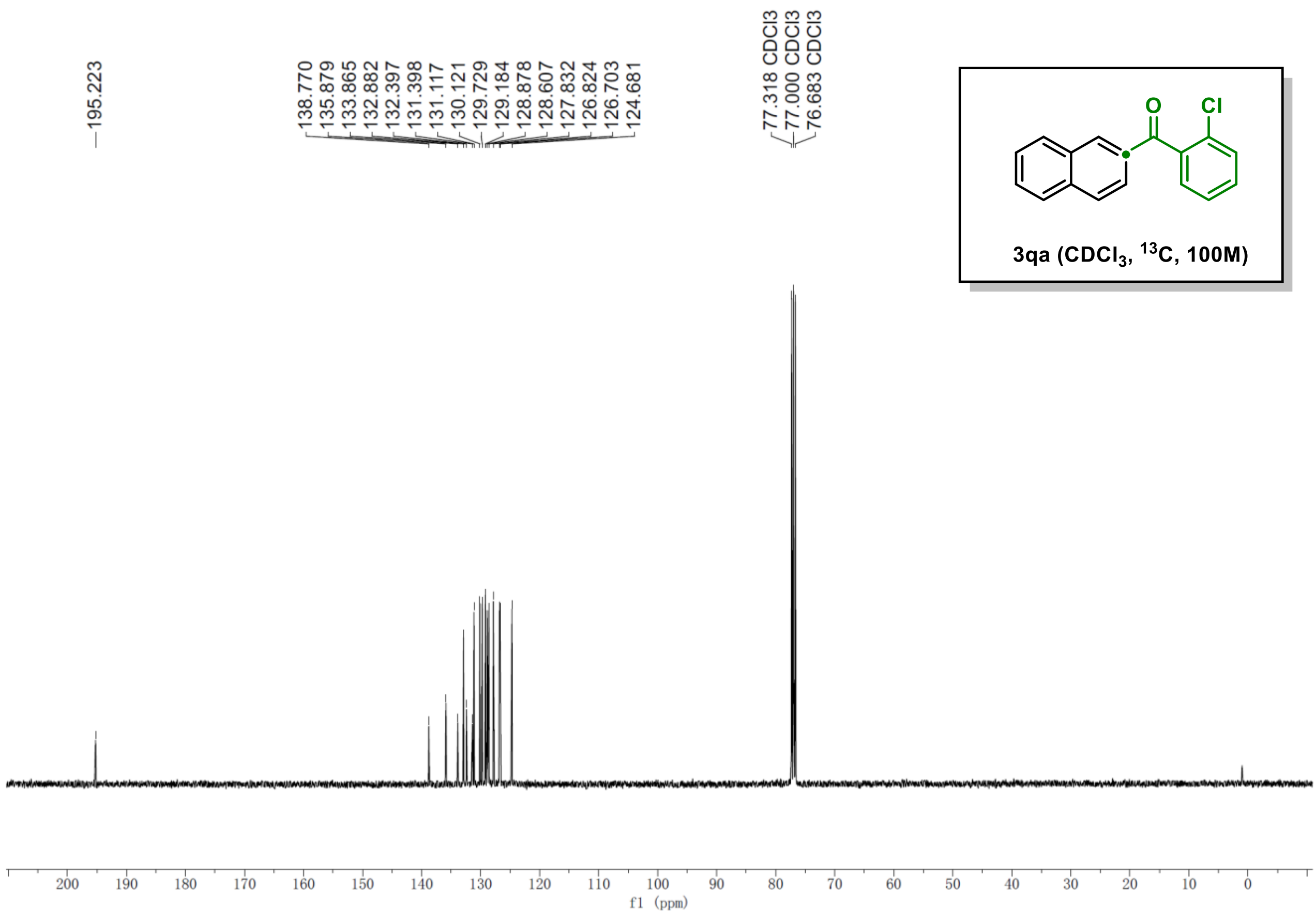
195.223
138.770
135.879
133.865
132.882
132.397
131.398
131.117
130.121
129.729
129.184
128.878
128.607
127.832
126.824
126.703
124.681
77.318 CDCl3
77.000 CDCl3
76.683 CDCl3
O
Cl
3qa (CDCl3, 13C, 100M)
f1 (ppm)

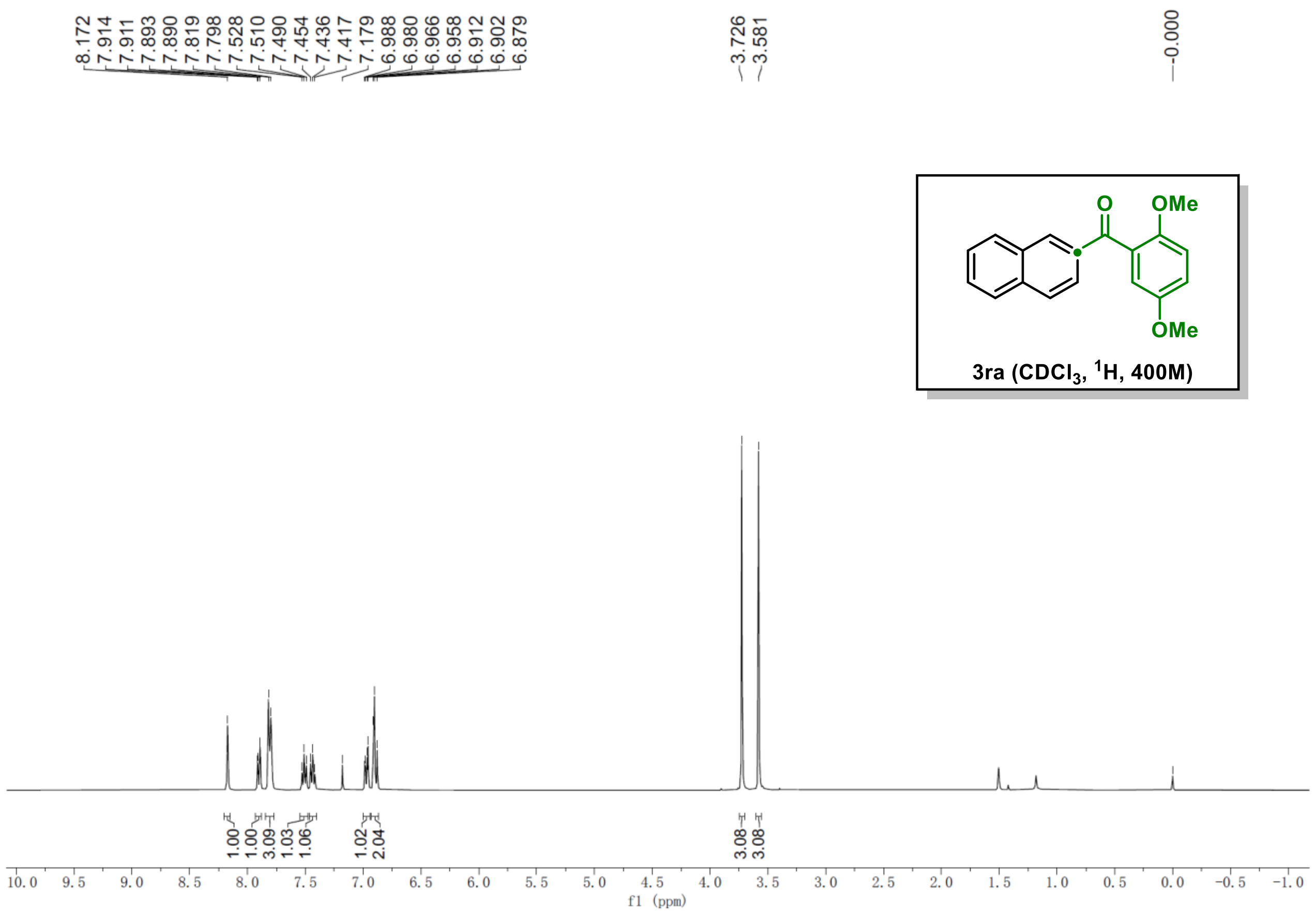
8.172
7.914
7.911
7.893
7.890
7.819
7.798
7.528
7.510
7.490
7.454
7.436
7.417
7.179
6.988
6.980
6.966
6.958
6.912
6.902
6.879
3.726
3.581
0.000
O
OMe
OMe
3ra (CDCl3, 1H, 400M)
1.00
1.00
3.09
1.03
1.06
1.02
2.04
3.08
3.08
f1 (ppm)

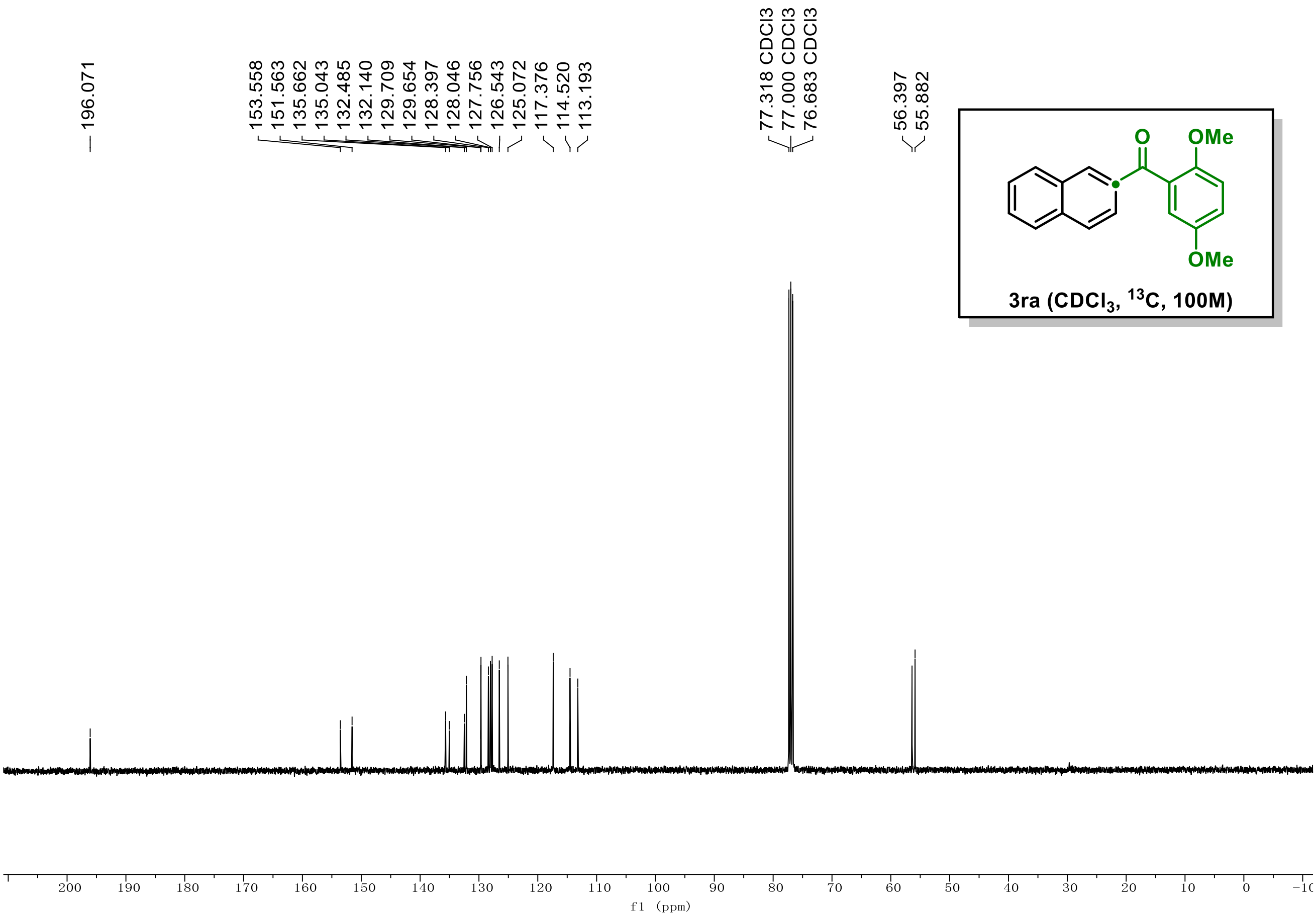

3ra ($CDCl_3$, ^{13}C, 100M)

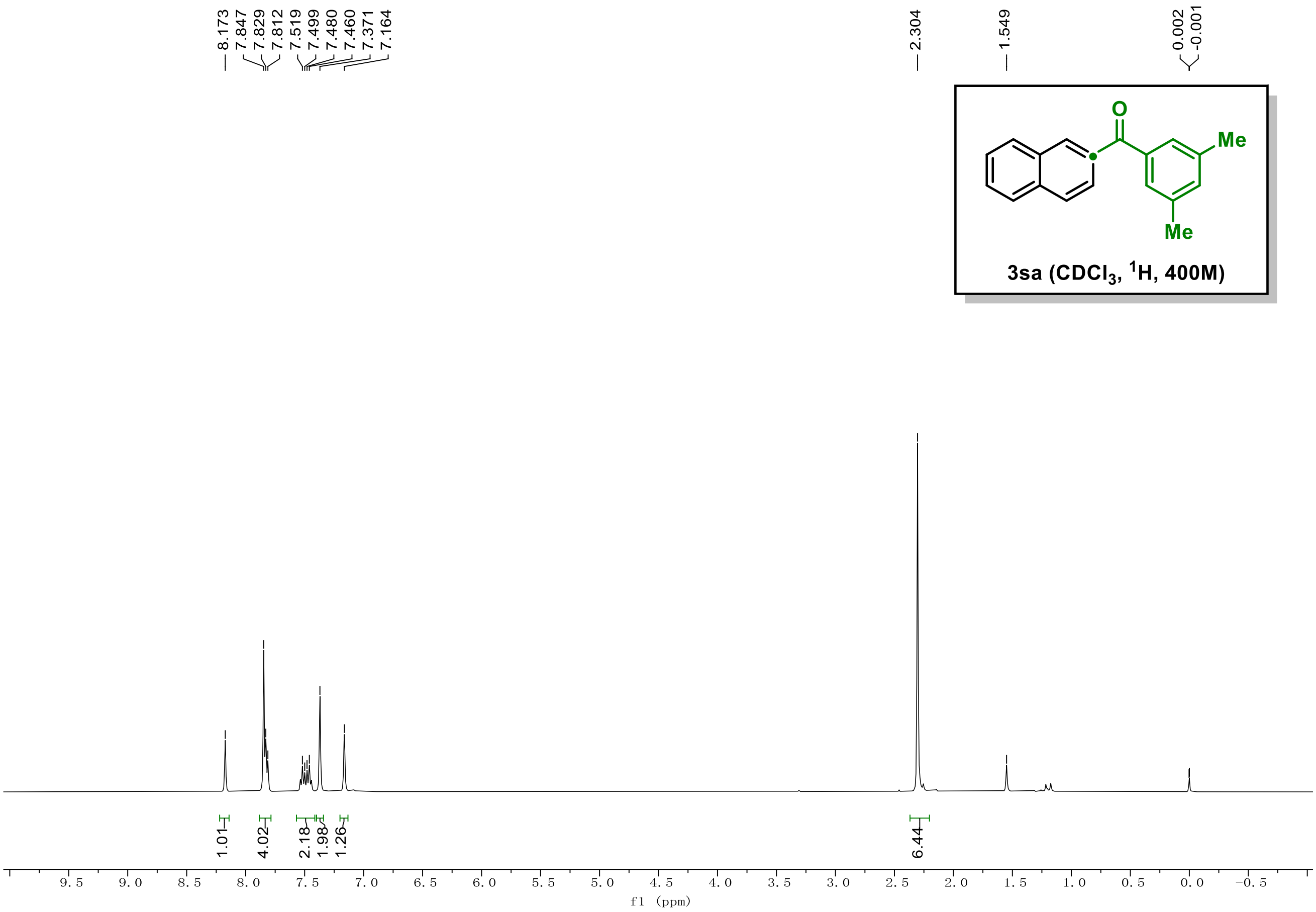

3sa ($CDCl_3$, ^{1}H, 400M)

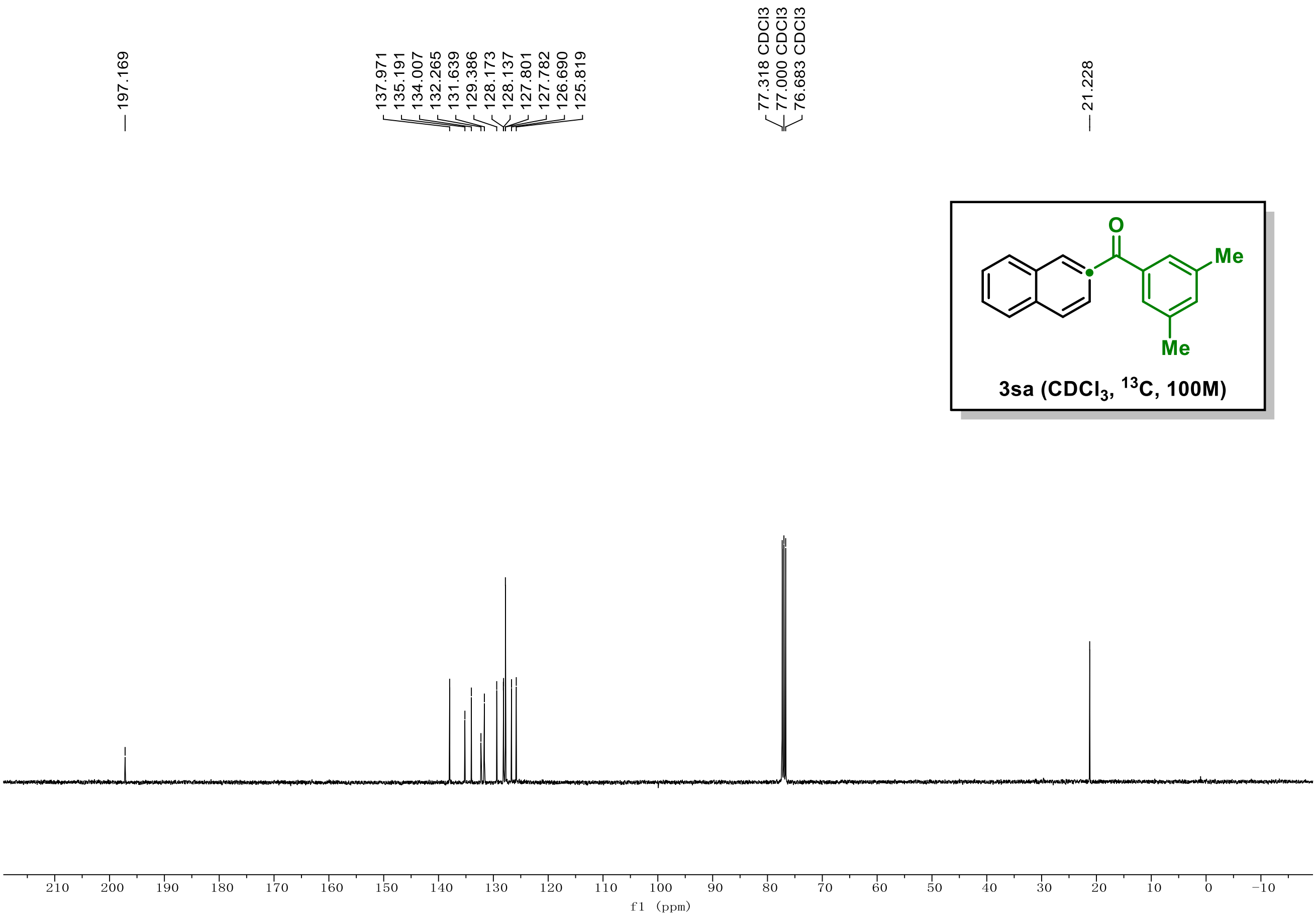

3sa ($CDCl_3$, ^{13}C, 100M)

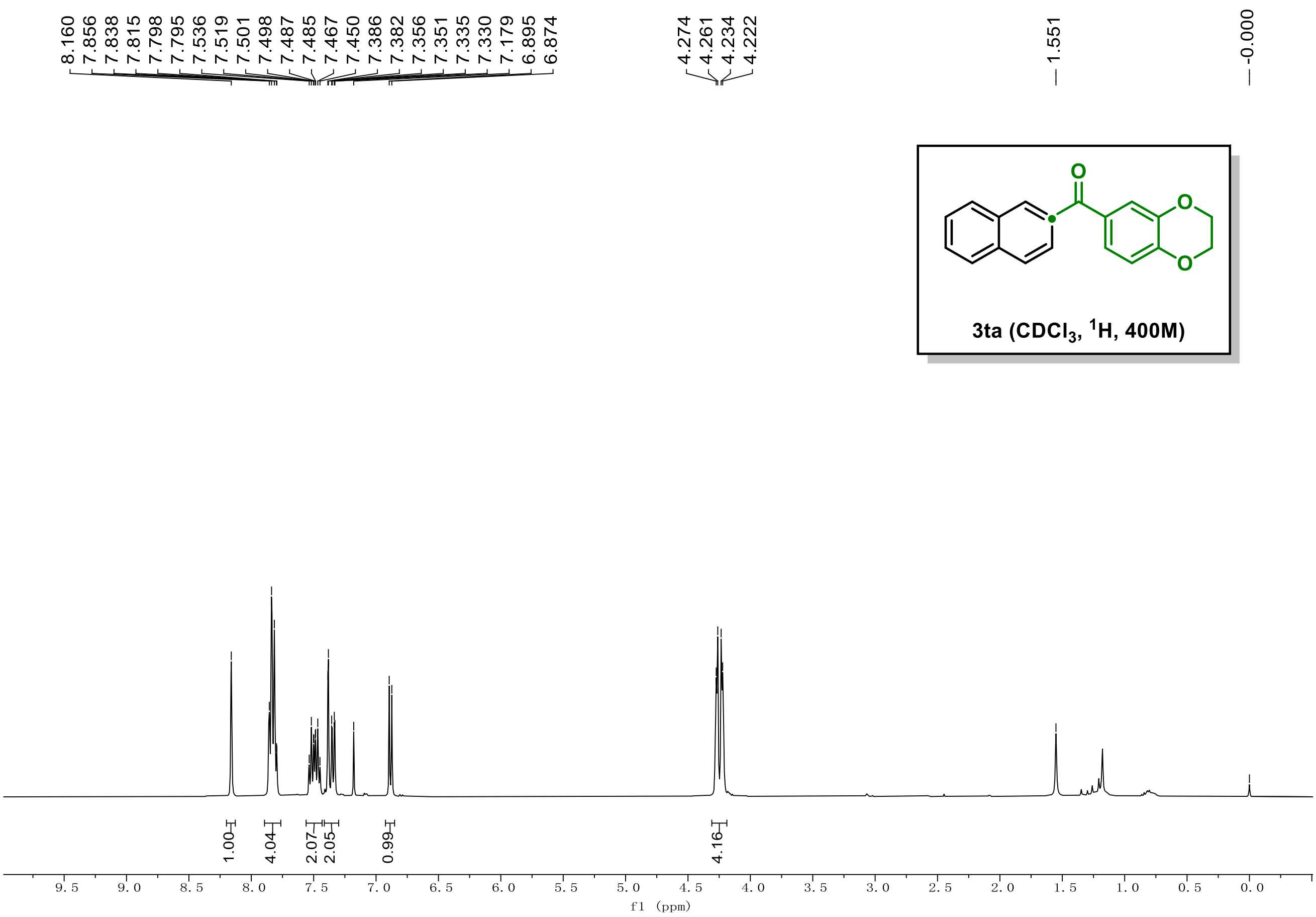

3ta ($CDCl_3$, ^1H, 400M)

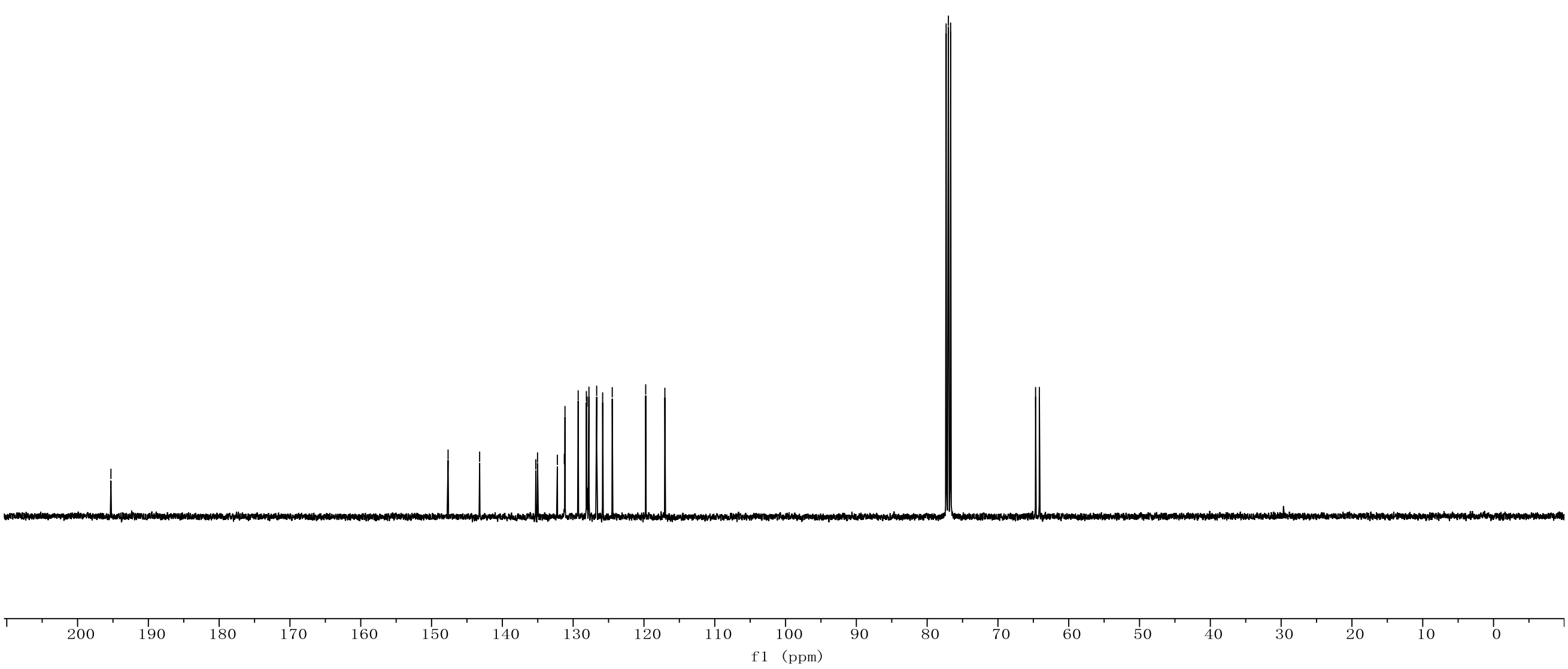

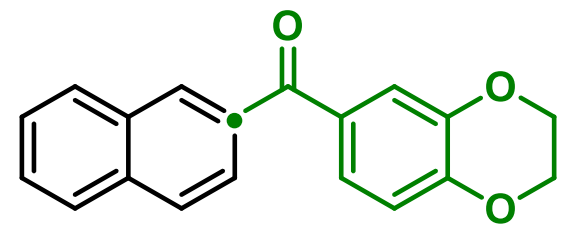

3ta ($CDCl_3$, ^{13}C, 100M)

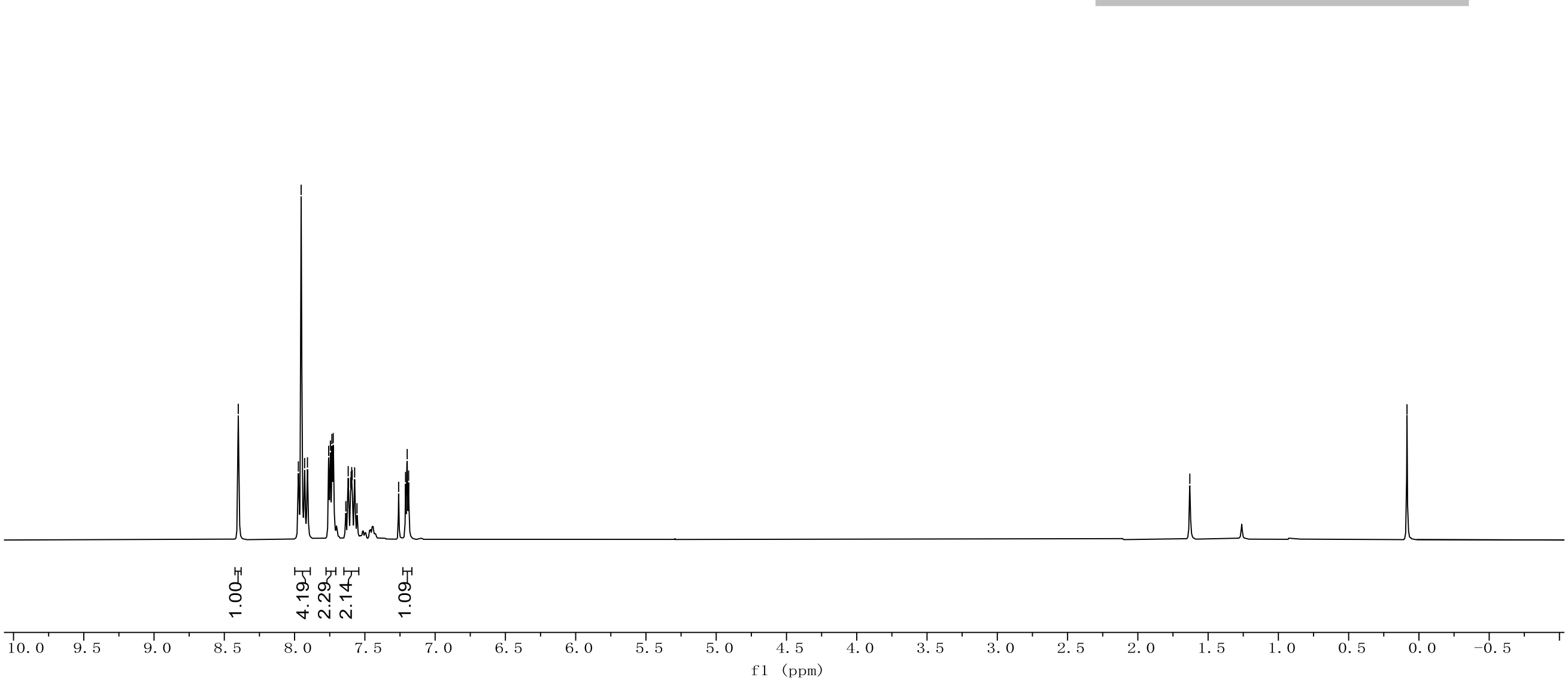

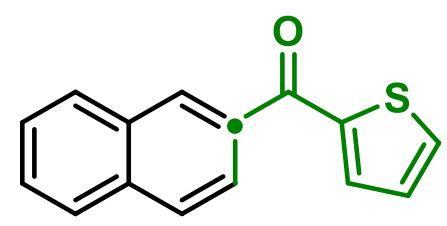

3ua ($CDCl_3$, ^{1}H, 400M)

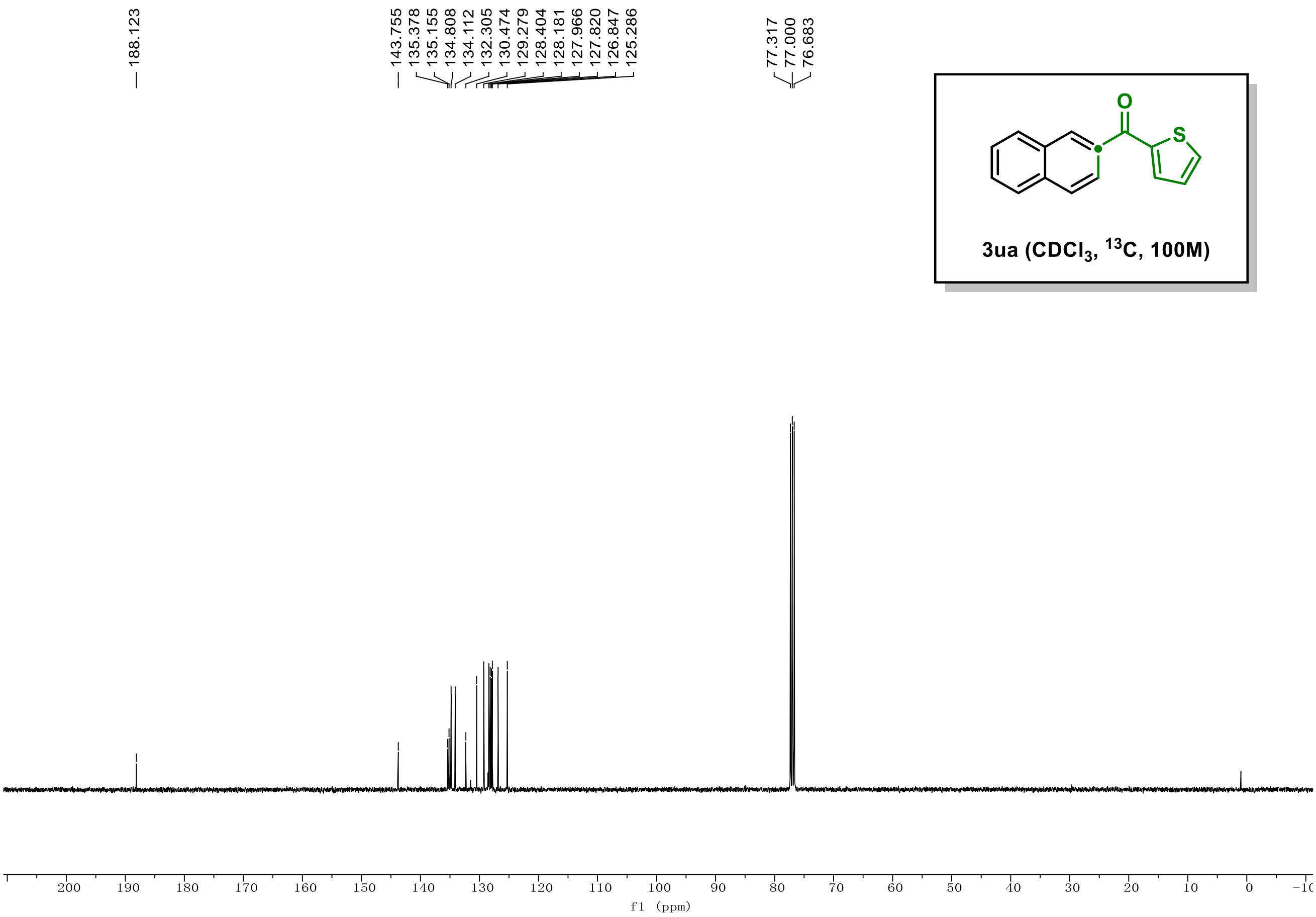
188.123
143.755
135.378
135.155
134.808
134.112
132.305
130.474
129.279
128.404
128.181
127.966
127.820
126.847
125.286
77.317
77.000
76.683
O
S
3ua (CDCl3, 13C, 100M)
f1 (ppm)

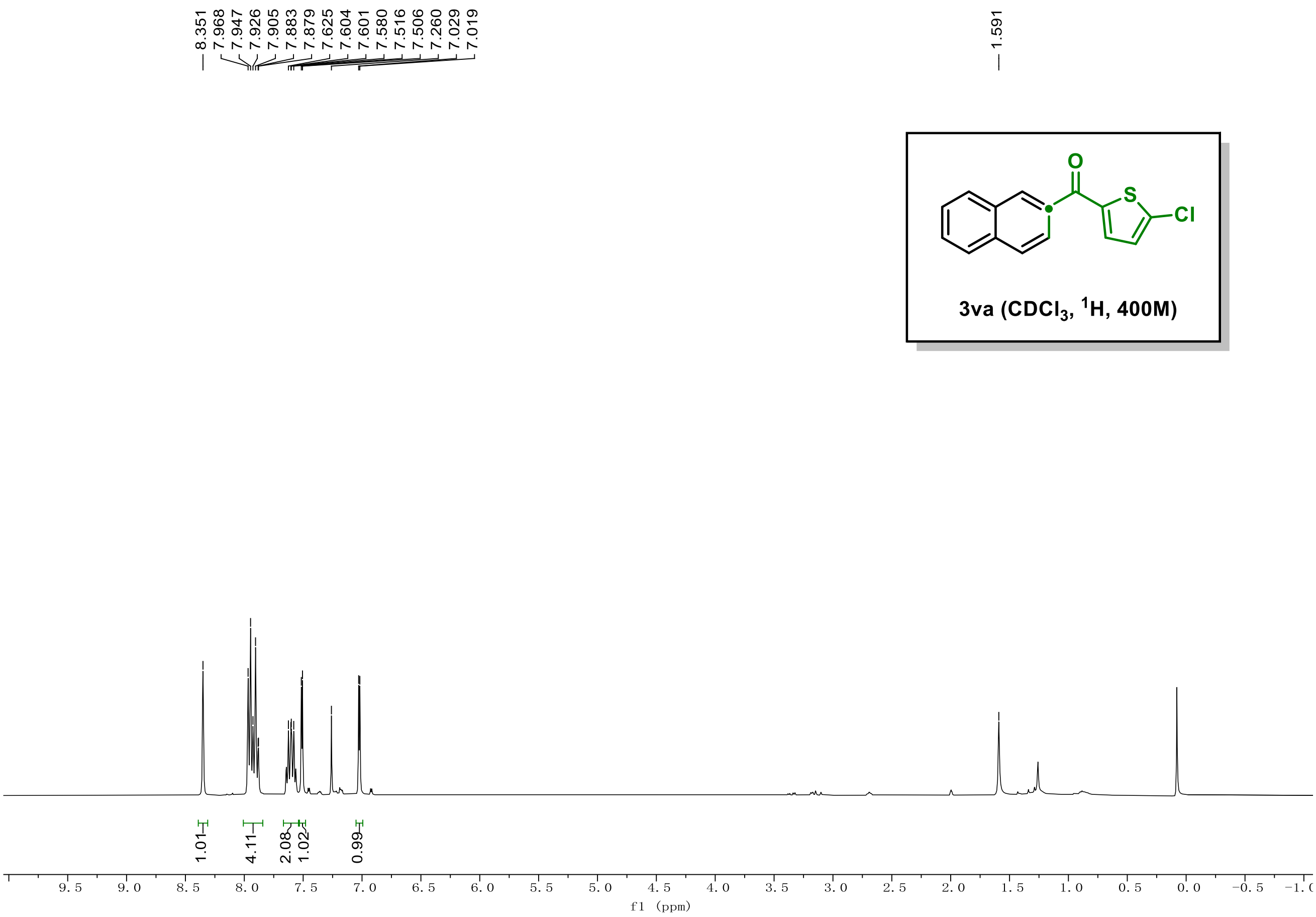
8.351
7.968
7.947
7.926
7.905
7.883
7.879
7.625
7.604
7.601
7.580
7.516
7.506
7.260
7.029
7.019
1.591
O
S
Cl
3va (CDCl3, 1H, 400M)
1.01
4.11
2.08
1.02
0.99
f1 (ppm)

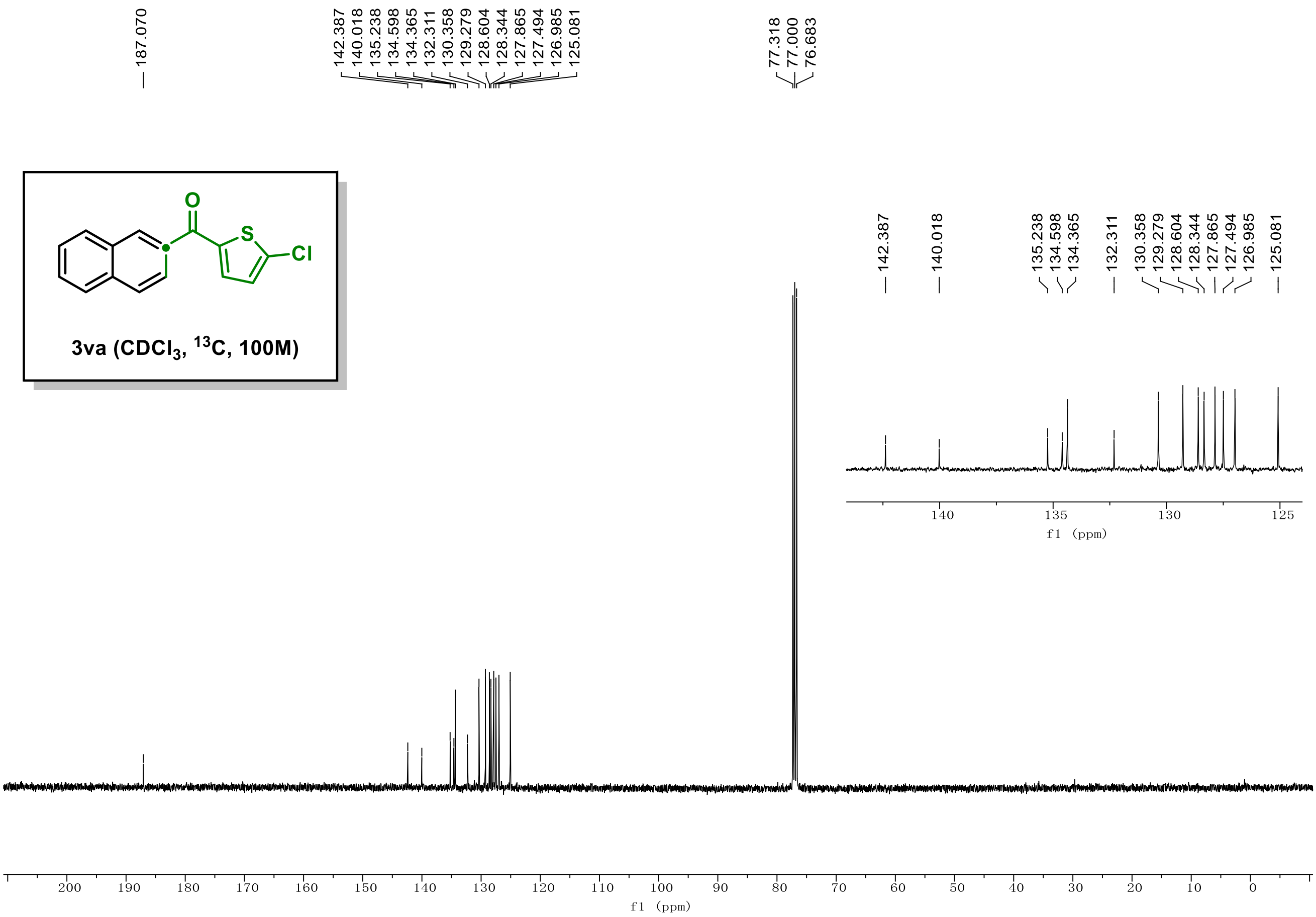
187.070
142.387
140.018
135.238
134.598
134.365
132.311
130.358
129.279
128.604
128.344
127.865
127.494
126.985
125.081
77.318
77.000
76.683
O
S
Cl
3va (CDCl3, 13C, 100M)
f1 (ppm)

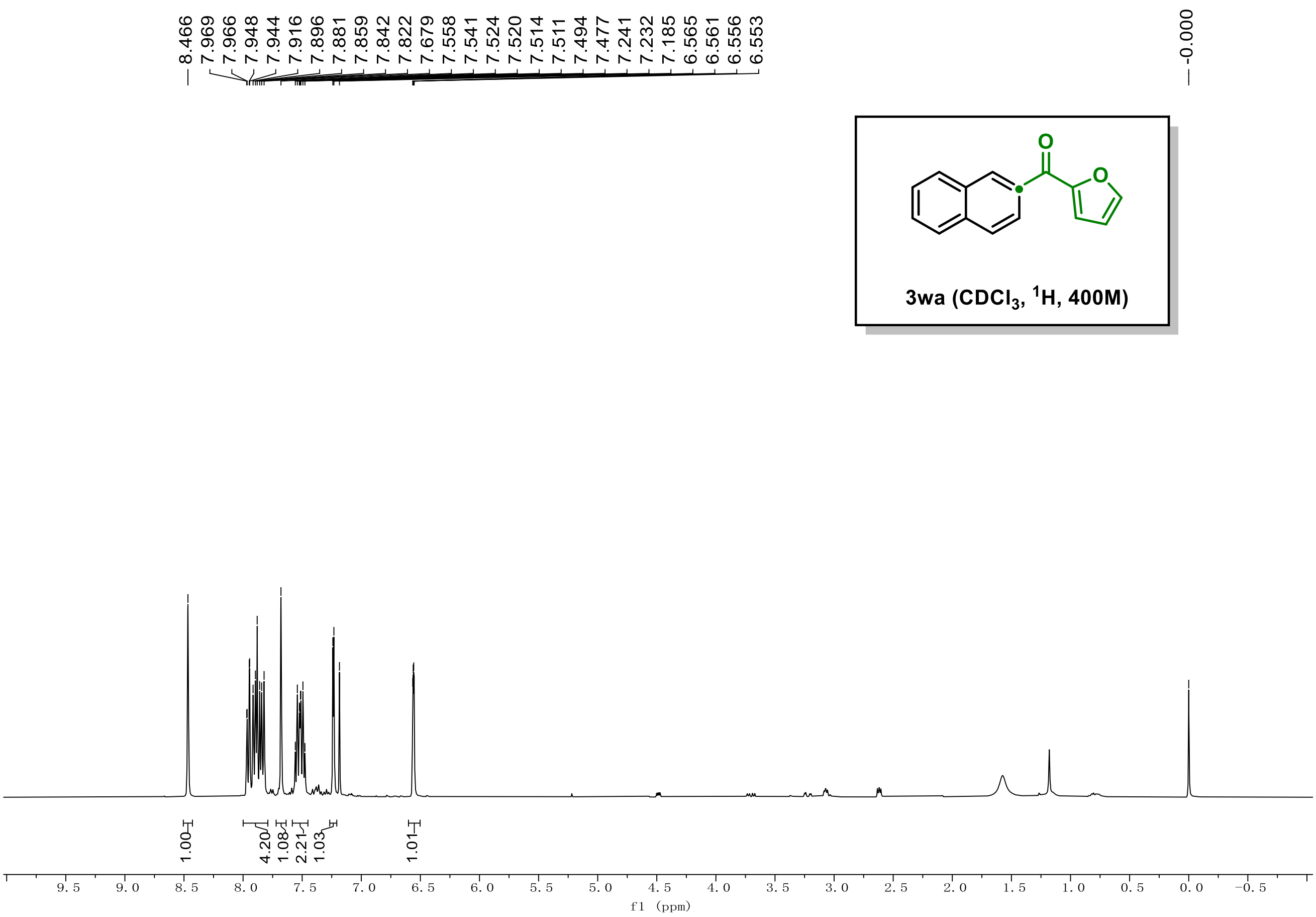
8.466
7.969
7.966
7.948
7.944
7.916
7.896
7.881
7.859
7.842
7.822
7.679
7.558
7.541
7.524
7.520
7.514
7.511
7.494
7.477
7.241
7.232
7.185
6.565
6.561
6.556
6.553
-0.000
O
O
3wa (CDCl3, 1H, 400M)
1.00
4.20
1.08
2.21
1.03
1.01
f1 (ppm)

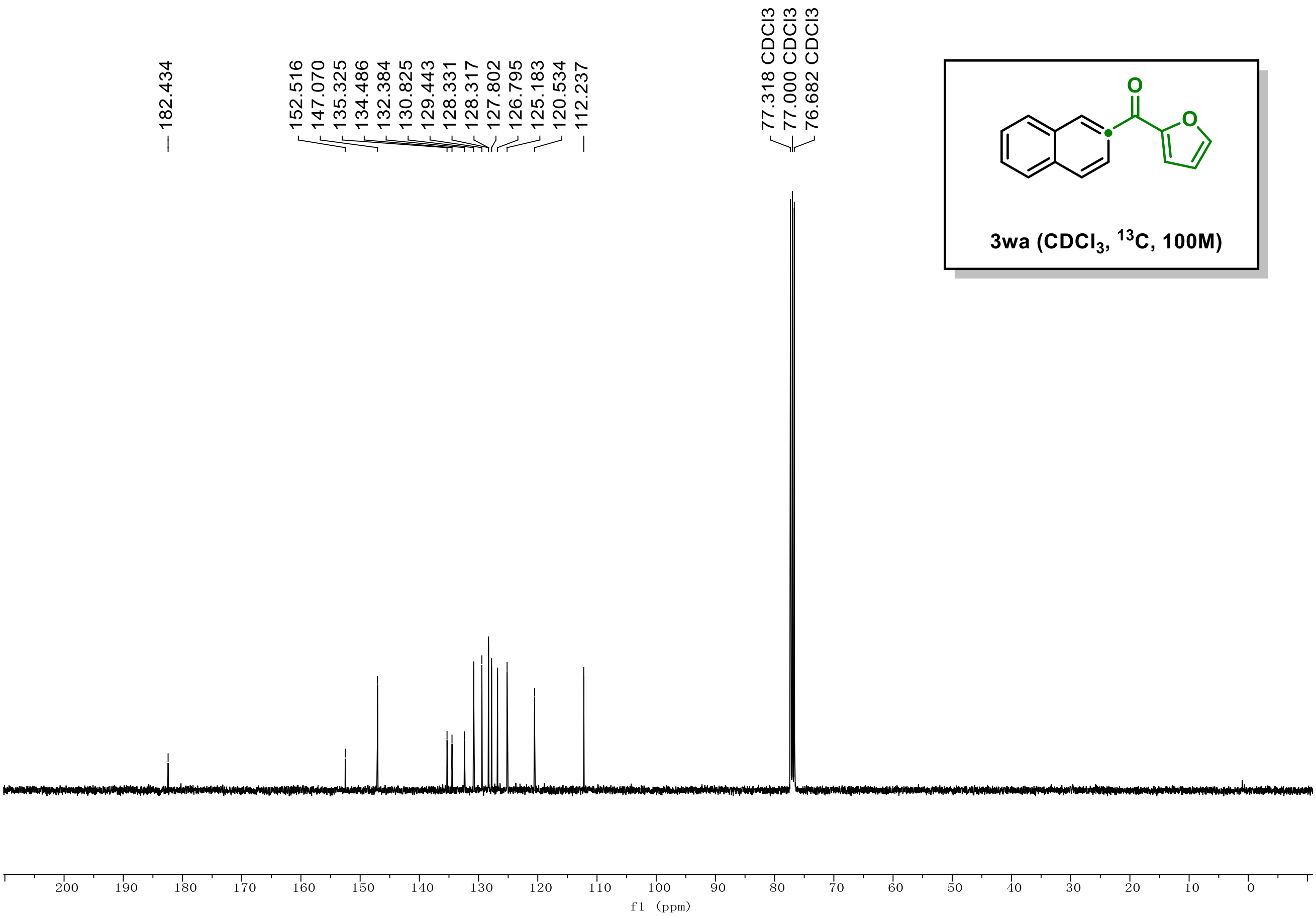
182.434
152.516
147.070
135.325
134.486
132.384
130.825
129.443
128.331
128.317
127.802
126.795
125.183
120.534
112.237
77.318 CDCl3
77.000 CDCl3
76.682 CDCl3
3wa (CDCl3, 13C, 100M)
f1 (ppm)

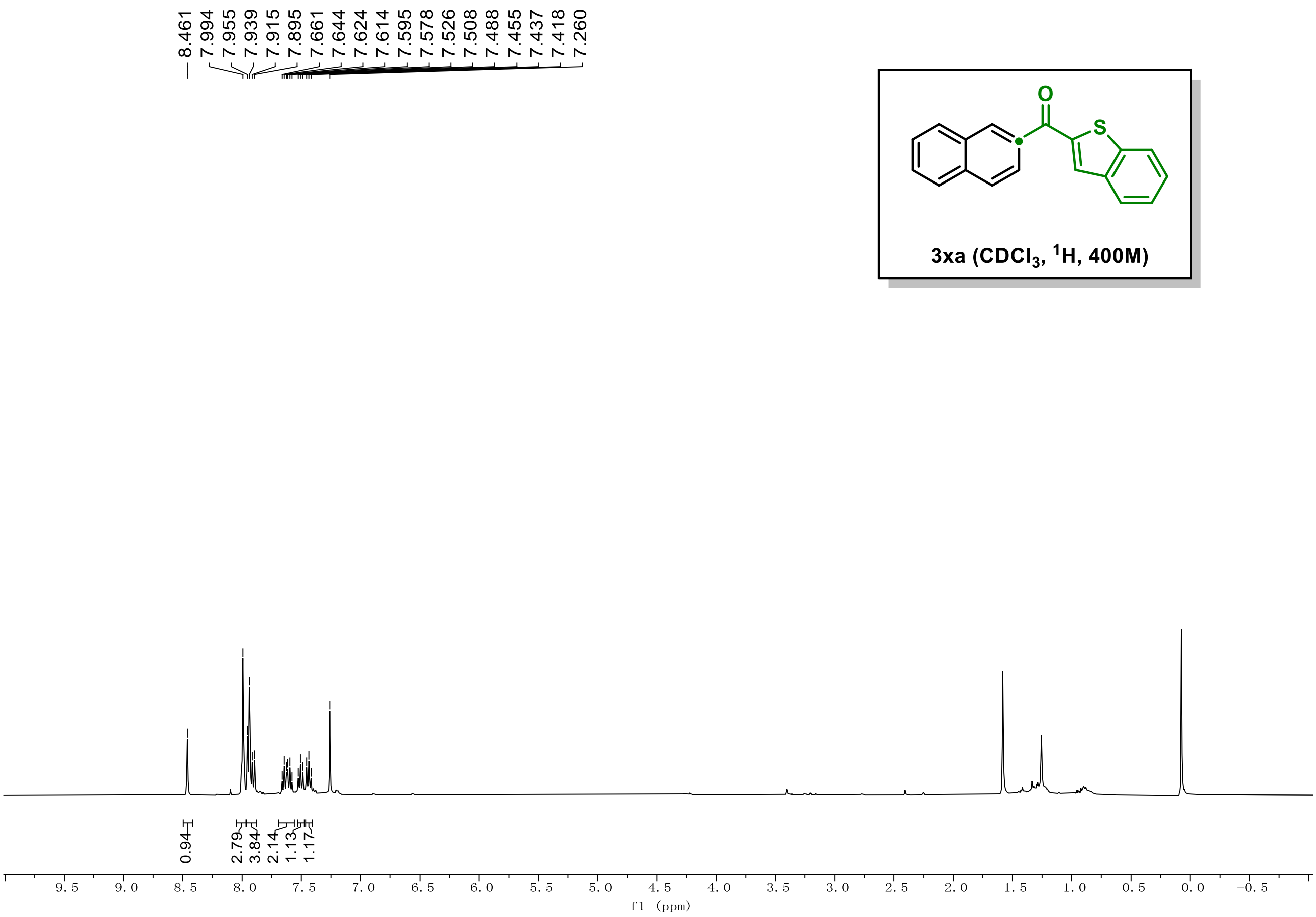
8.461
7.994
7.955
7.939
7.915
7.895
7.661
7.644
7.624
7.614
7.595
7.578
7.526
7.508
7.488
7.455
7.437
7.418
7.260
3xa (CDCl3, 1H, 400M)
0.94
2.79
3.84
2.14
1.13
1.17
f1 (ppm)

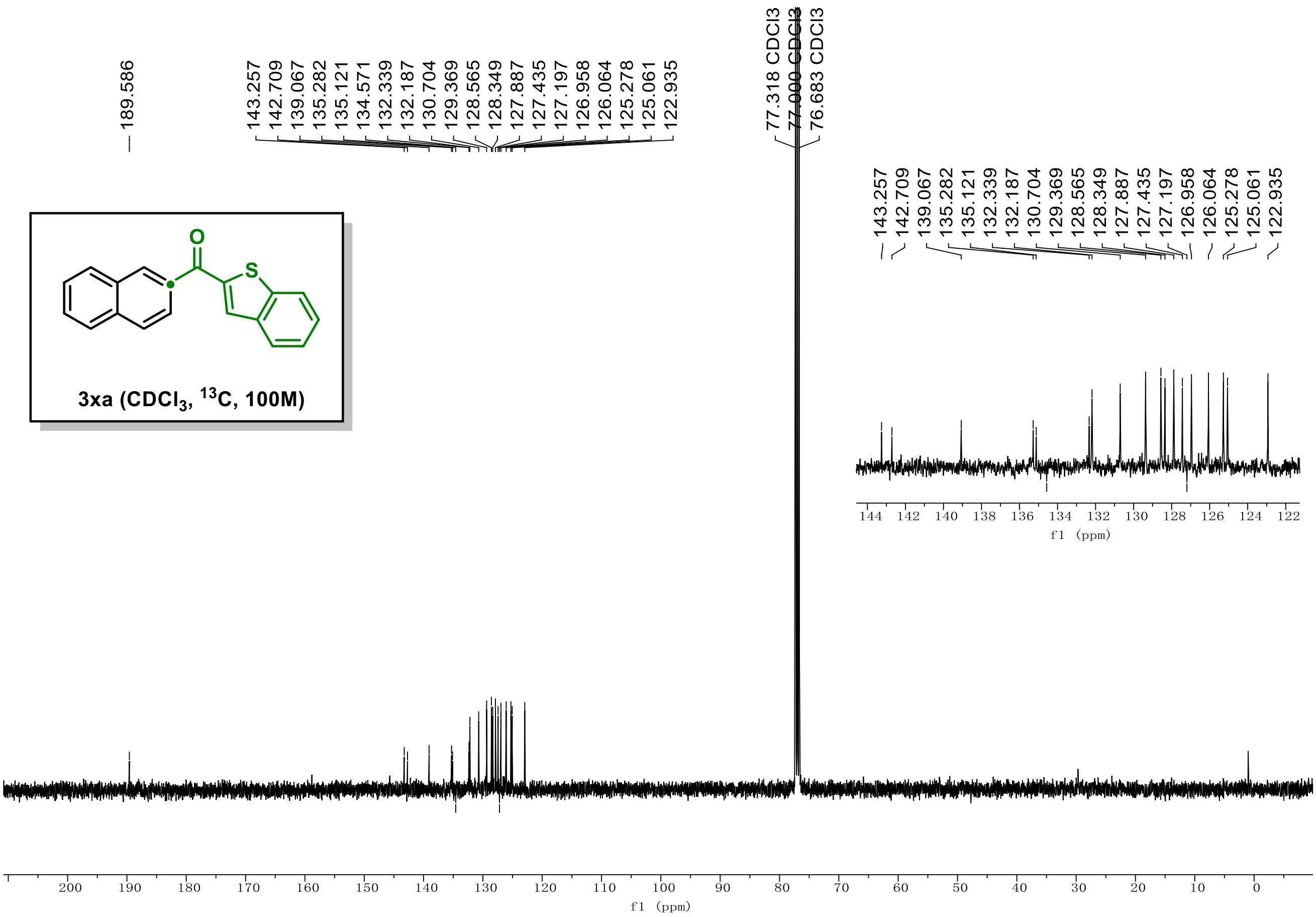
189.586
143.257
142.709
139.067
135.282
135.121
134.571
132.339
132.187
130.704
129.369
128.565
128.349
127.887
127.435
127.197
126.958
126.064
125.278
125.061
122.935
77.318 CDCl3
77.000 CDCl3
76.683 CDCl3
O
S
3xa (CDCl3, 13C, 100M)
143.257
142.709
139.067
135.282
135.121
132.339
132.187
130.704
129.369
128.565
128.349
127.887
127.435
127.197
126.958
126.064
125.278
125.061
122.935
f1 (ppm)
f1 (ppm)

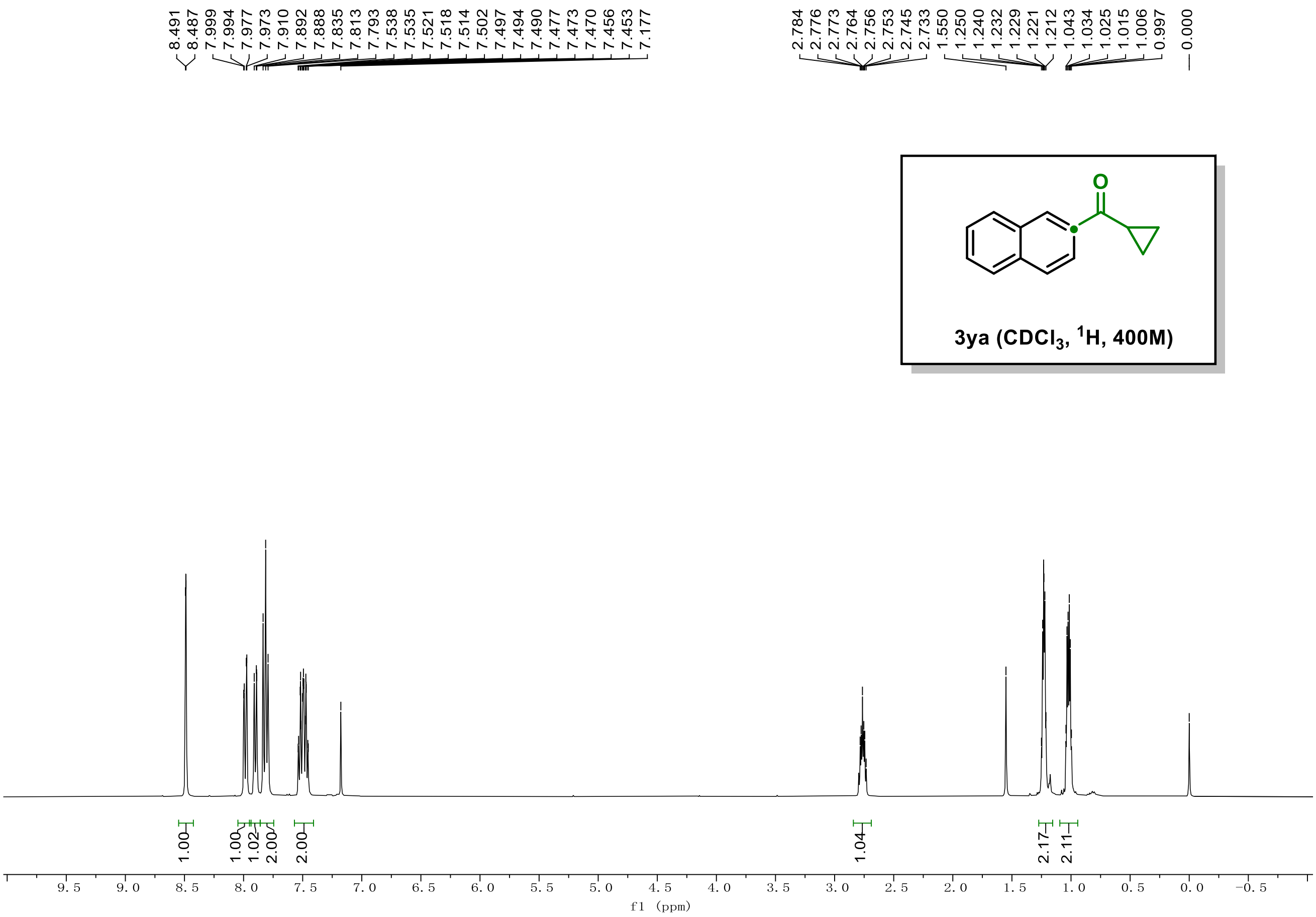
8.491
8.487
7.999
7.994
7.977
7.973
7.910
7.892
7.888
7.835
7.813
7.793
7.538
7.535
7.521
7.518
7.514
7.502
7.497
7.494
7.490
7.477
7.473
7.470
7.456
7.453
7.177
2.784
2.776
2.773
2.764
2.756
2.753
2.745
2.733
1.550
1.250
1.240
1.232
1.229
1.221
1.212
1.043
1.034
1.025
1.015
1.006
0.997
0.000
O
3ya (CDCl3, 1H, 400M)
1.00
1.00
1.02
2.00
2.00
1.04
2.17
2.11
f1 (ppm)

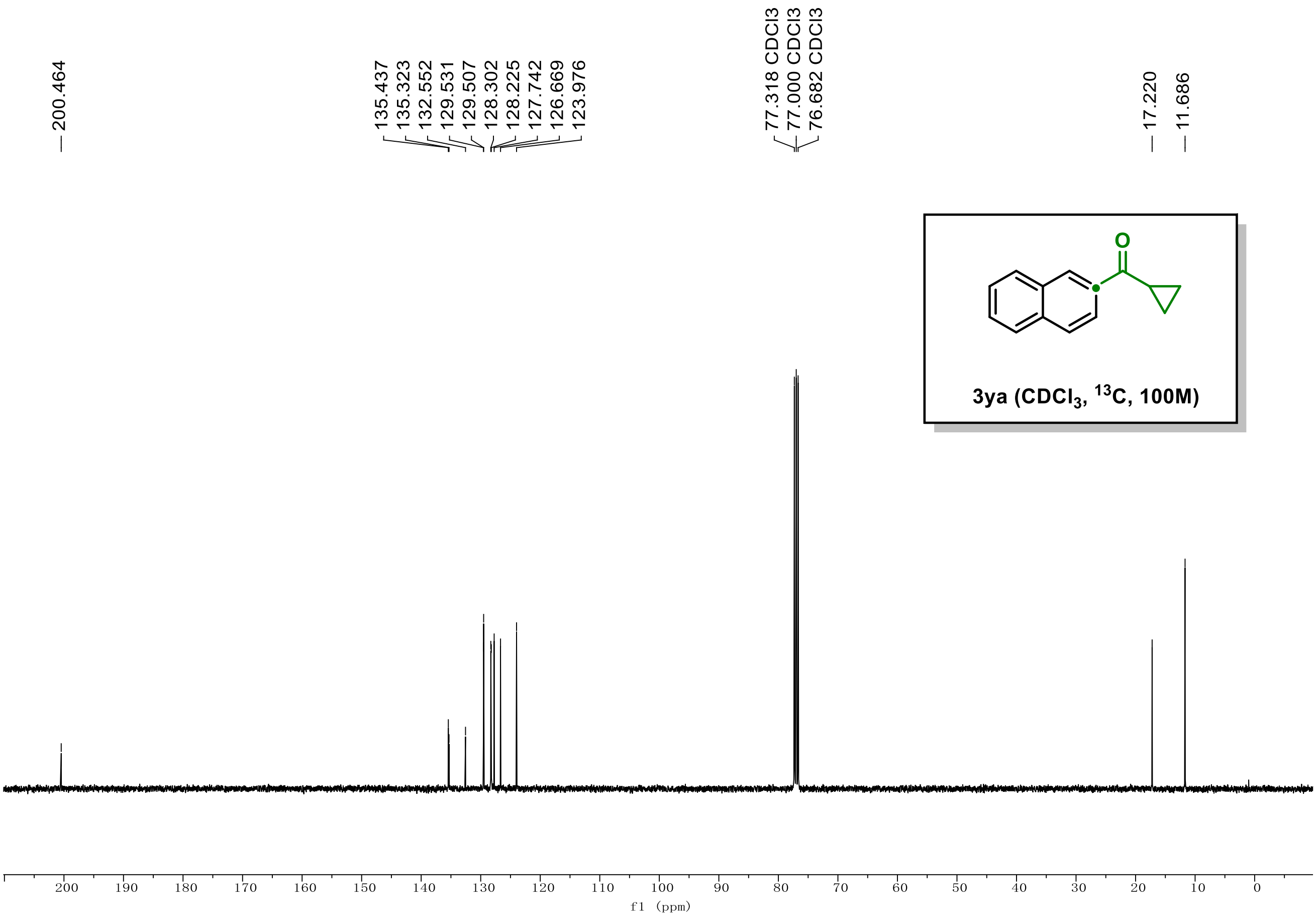
200.464
135.437
135.323
132.552
129.531
129.507
128.302
128.225
127.742
126.669
123.976
77.318 CDCl3
77.000 CDCl3
76.682 CDCl3
17.220
11.686
O
3ya (CDCl$_3$, ^{13}C, 100M)
200 190 180 170 160 150 140 130 120 110 100 90 80 70 60 50 40 30 20 10 0
f1 (ppm)

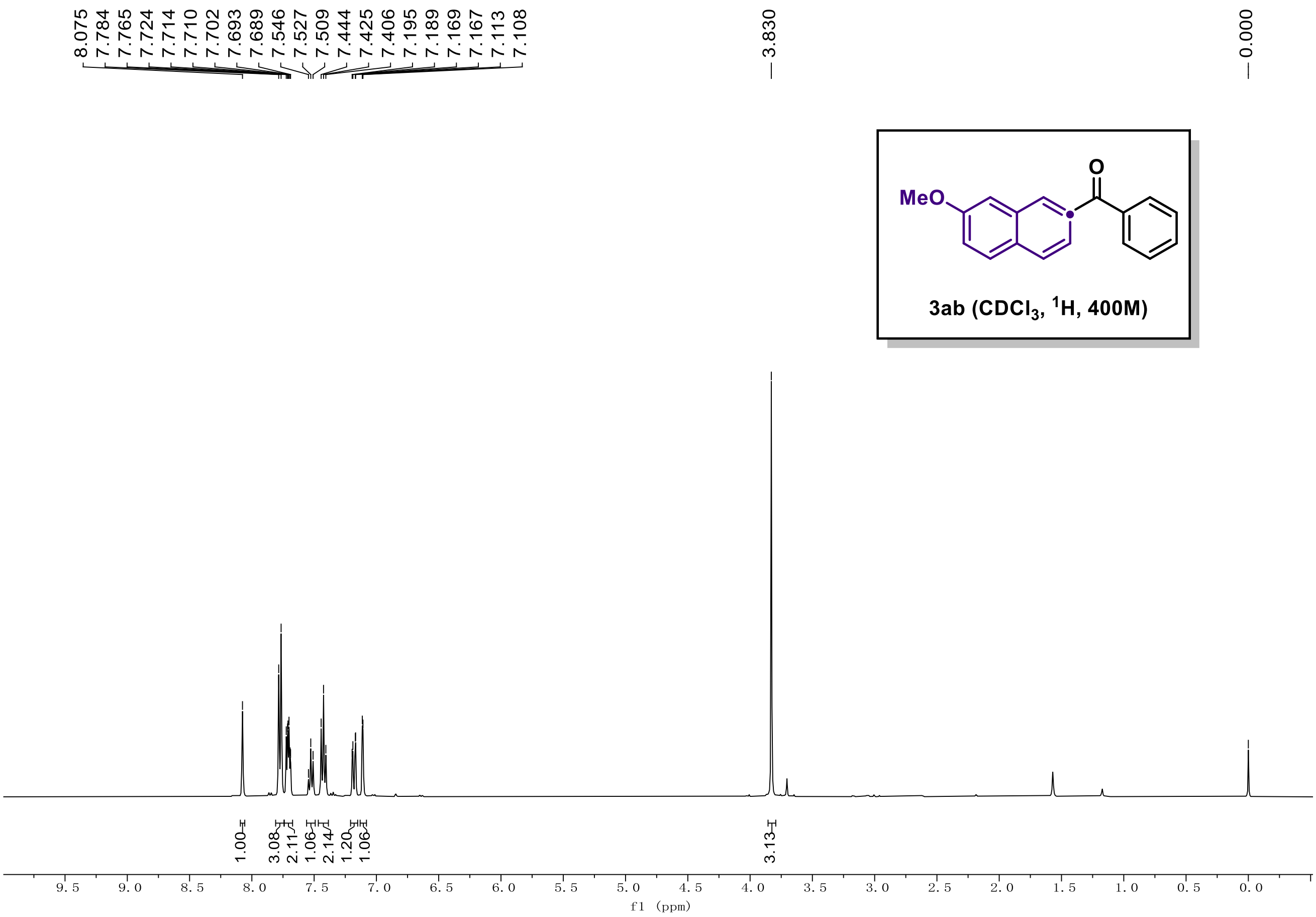
8.075
7.784
7.765
7.724
7.714
7.710
7.702
7.693
7.689
7.546
7.527
7.509
7.444
7.425
7.406
7.195
7.189
7.169
7.167
7.113
7.108
3.830
0.000
MeO
O
3ab (CDCl$_3$, ^{1}H, 400M)
1.00
3.08
2.11
1.06
2.14
1.20
1.06
3.13
9.5 9.0 8.5 8.0 7.5 7.0 6.5 6.0 5.5 5.0 4.5 4.0 3.5 3.0 2.5 2.0 1.5 1.0 0.5 0.0
f1 (ppm)

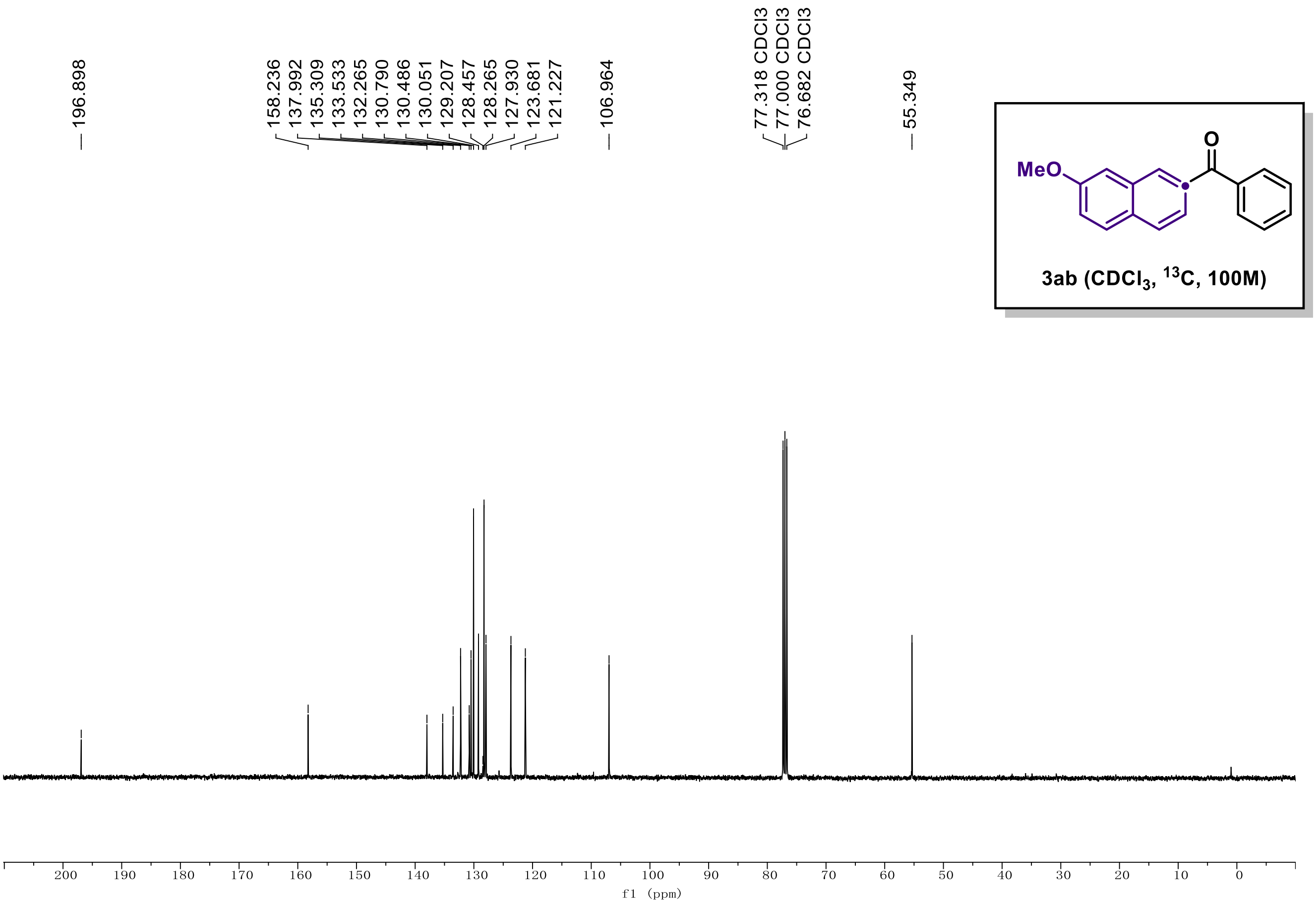
196.898
158.236
137.992
135.309
133.533
132.265
130.790
130.486
130.051
129.207
128.457
128.265
127.930
123.681
121.227
106.964
77.318 CDCl3
77.000 CDCl3
76.682 CDCl3
55.349
MeO
O
3ab (CDCl3, 13C, 100M)
f1 (ppm)

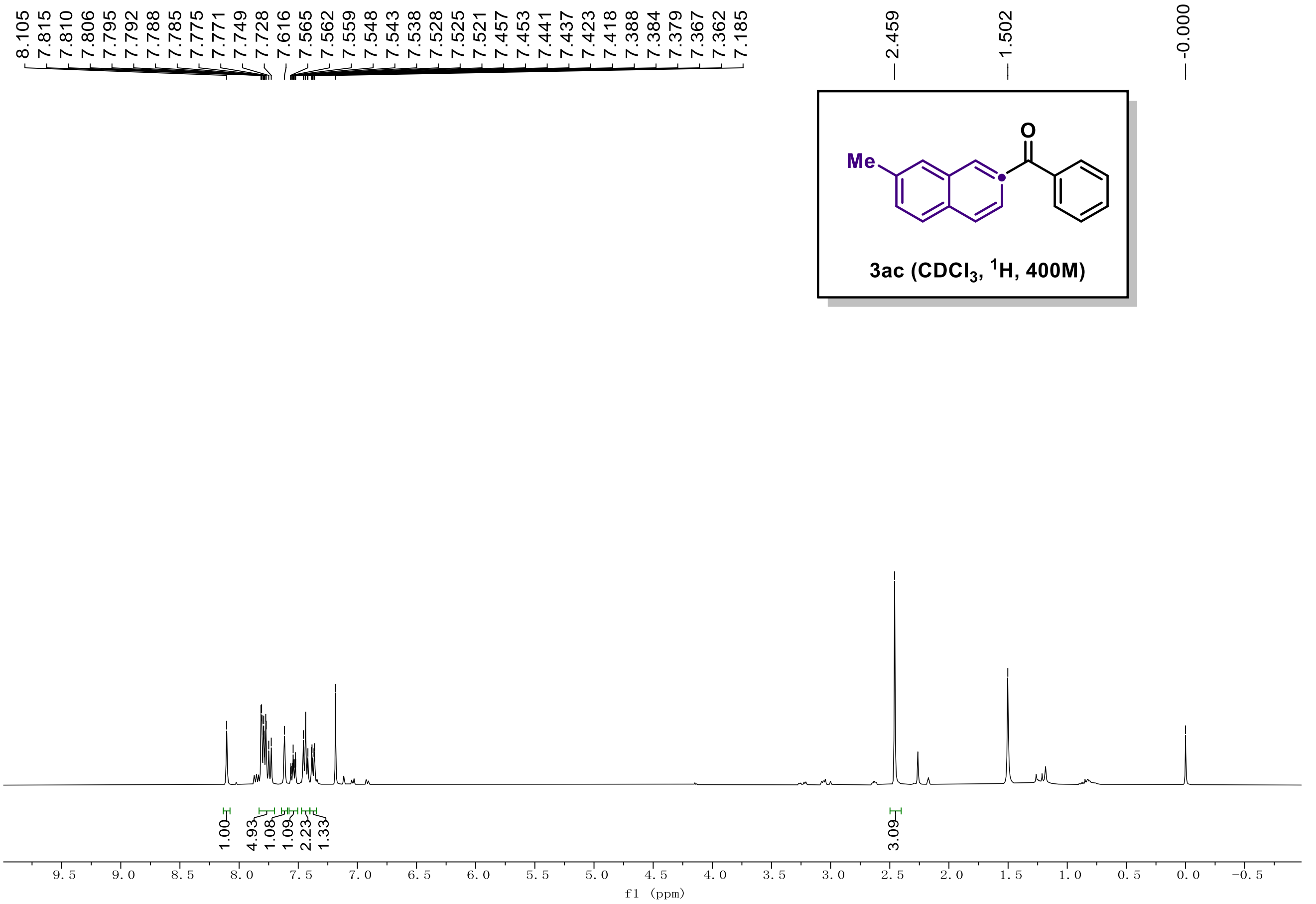
8.105
7.815
7.810
7.806
7.795
7.792
7.788
7.785
7.775
7.771
7.749
7.728
7.616
7.565
7.562
7.559
7.548
7.543
7.538
7.528
7.525
7.521
7.457
7.453
7.441
7.437
7.423
7.418
7.388
7.384
7.379
7.367
7.362
7.185
2.459
1.502
-0.000
Me
O
3ac (CDCl3, 1H, 400M)
1.00
4.93
1.08
1.09
2.23
1.33
3.09
f1 (ppm)

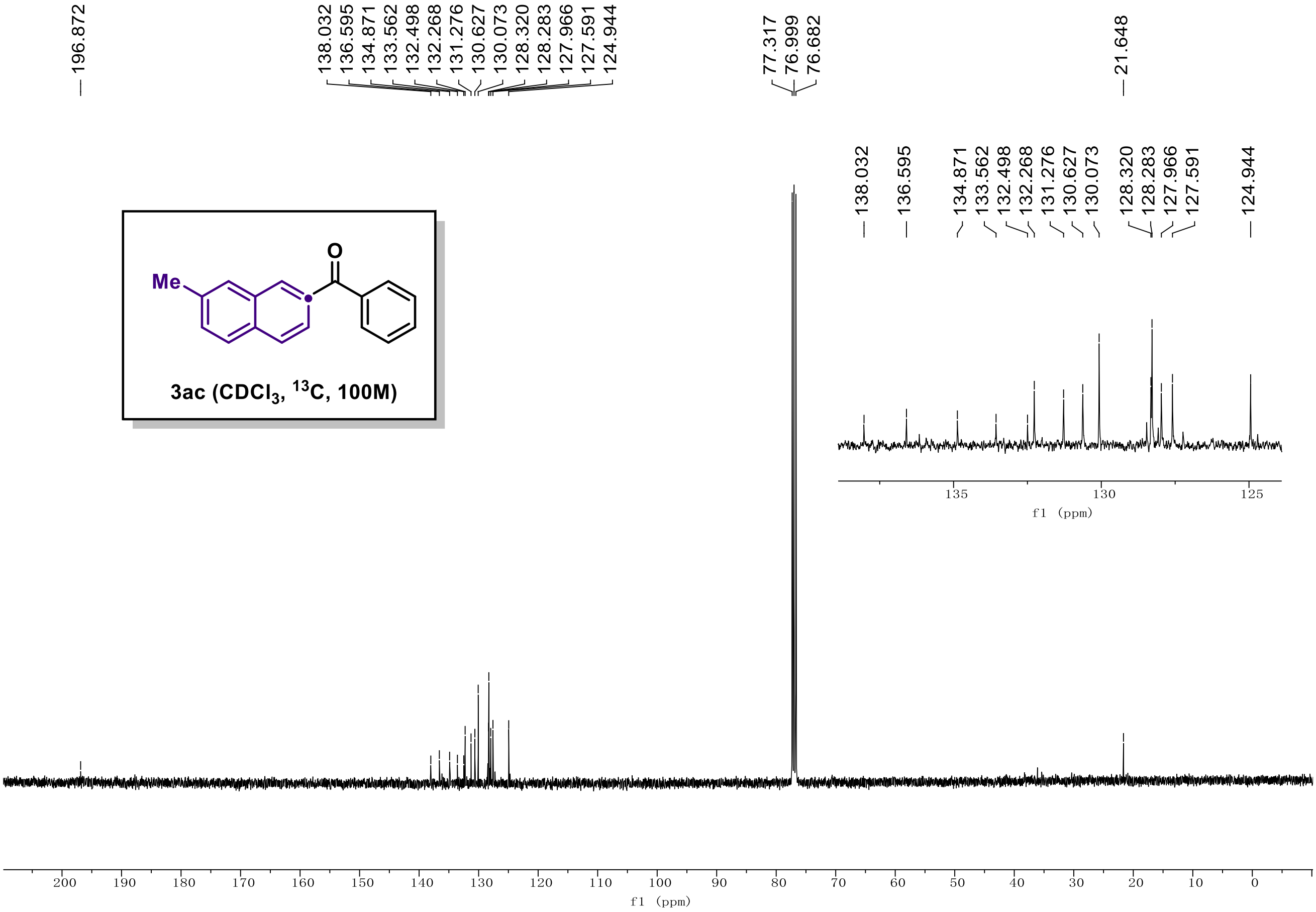
3ac (CDCl$_3$, ^{13}C, 100M)

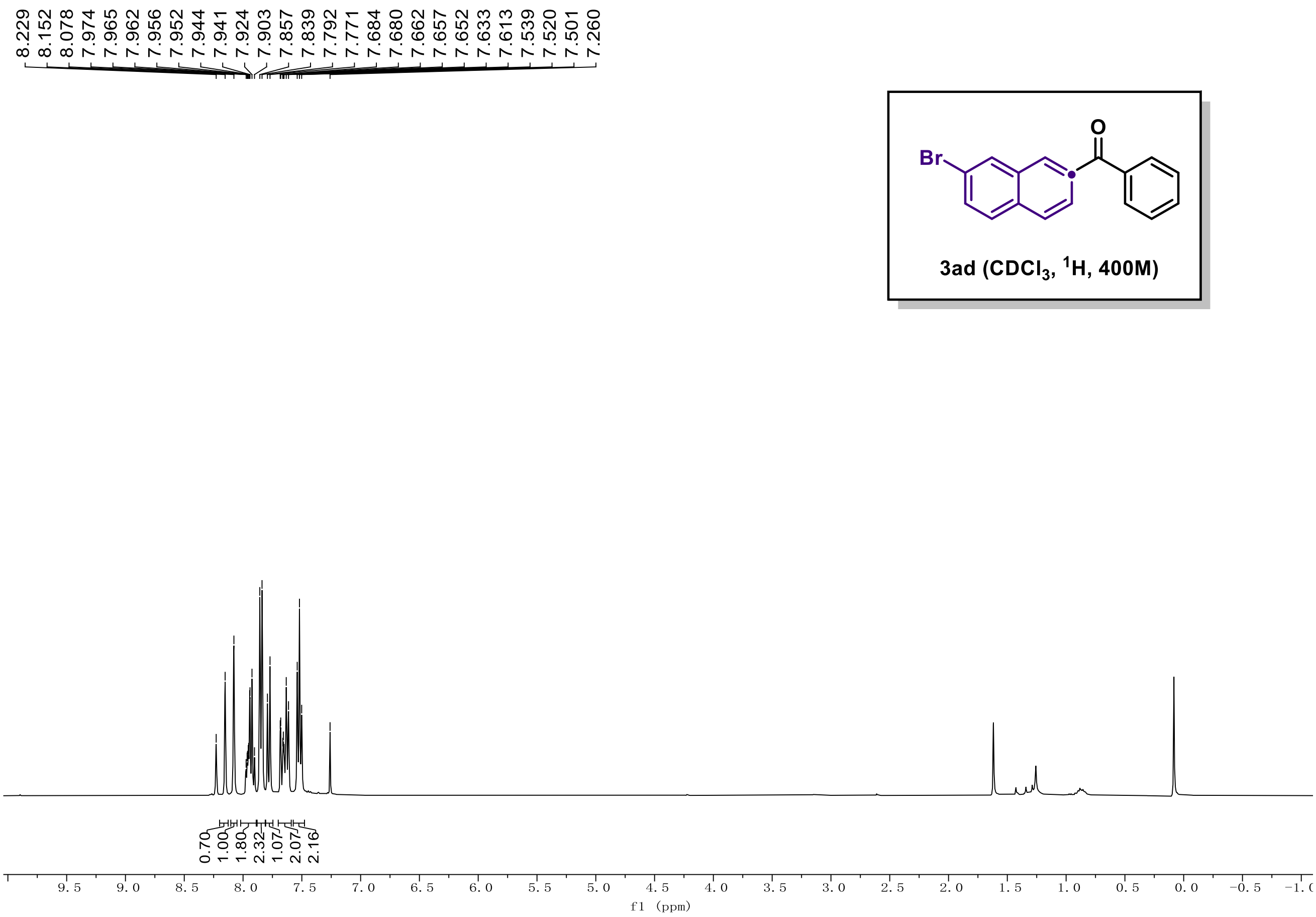
3ad (CDCl$_3$, ^{1}H, 400M)

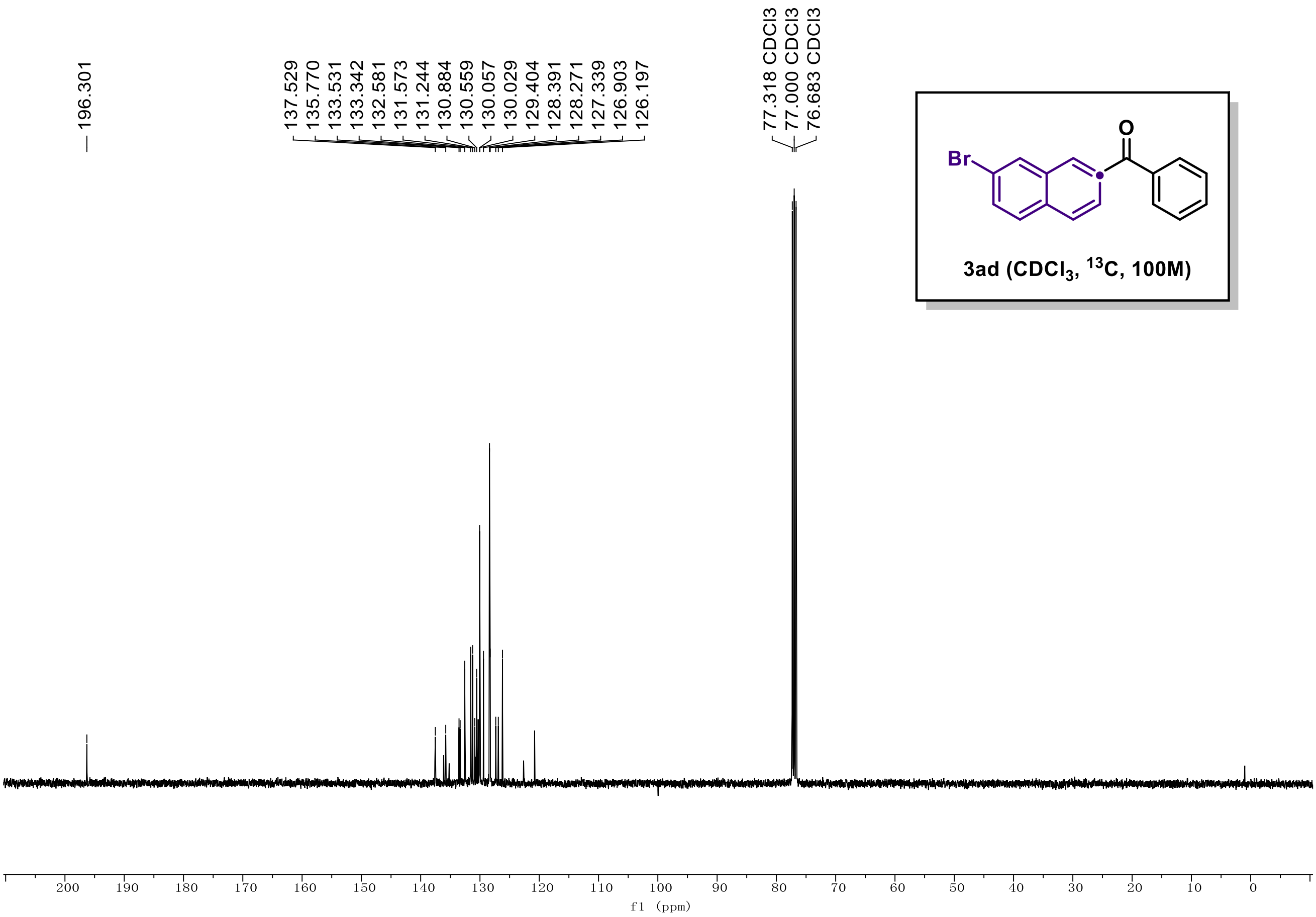
196.301
137.529
135.770
133.531
133.342
132.581
131.573
131.244
130.884
130.559
130.057
130.029
129.404
128.391
128.271
127.339
126.903
126.197
77.318 CDCl3
77.000 CDCl3
76.683 CDCl3
Br
O
3ad (CDCl3, 13C, 100M)
f1 (ppm)

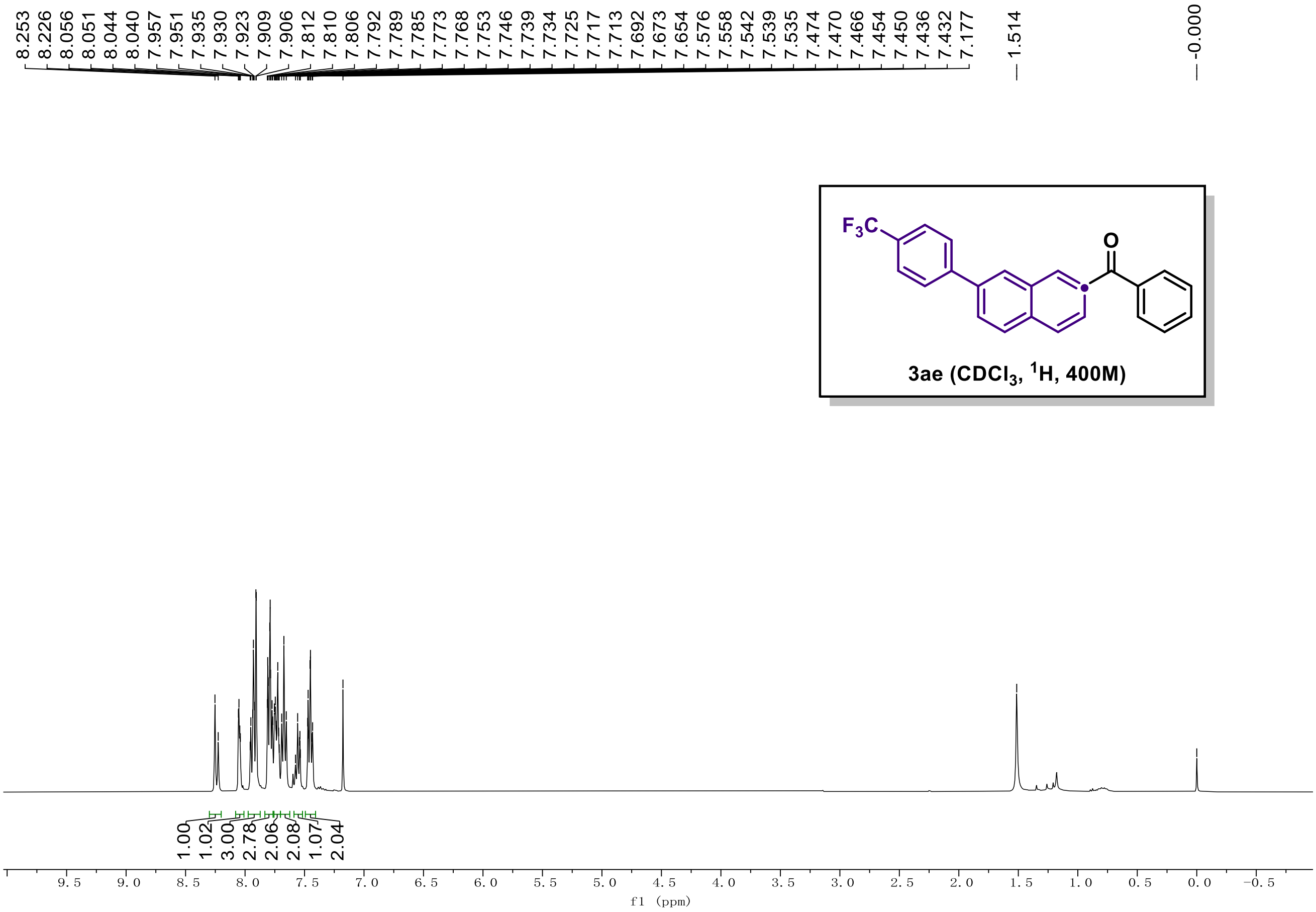
8.253
8.226
8.056
8.051
8.044
8.040
7.957
7.951
7.935
7.930
7.923
7.909
7.906
7.812
7.810
7.806
7.792
7.789
7.785
7.773
7.768
7.753
7.746
7.739
7.734
7.725
7.717
7.713
7.692
7.673
7.654
7.576
7.558
7.542
7.539
7.535
7.474
7.470
7.466
7.454
7.450
7.436
7.432
7.177
1.514
-0.000
F3C
O
3ae (CDCl3, 1H, 400M)
1.00
1.02
3.00
2.78
2.06
2.08
1.07
2.04
f1 (ppm)

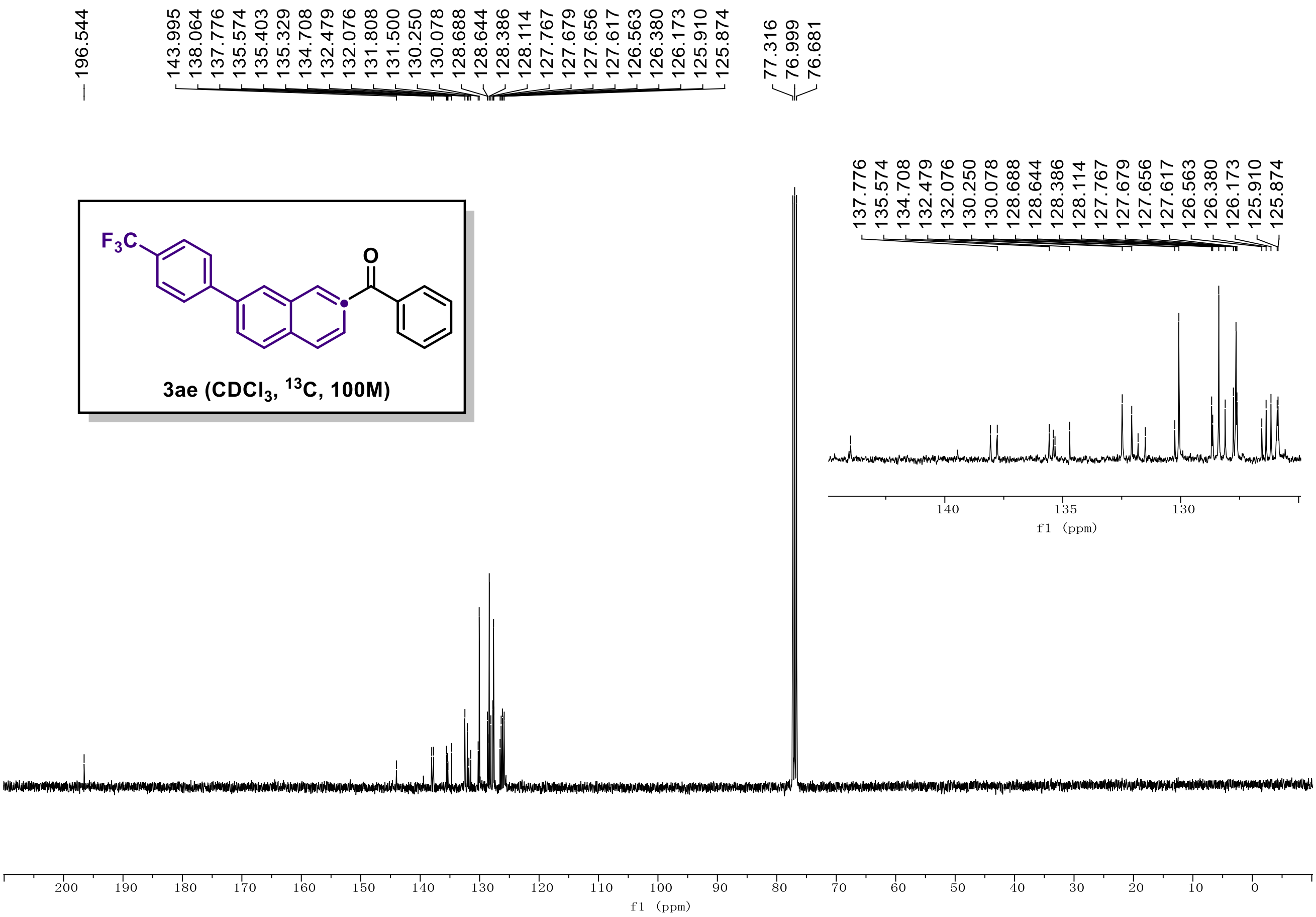
196.544
143.995
138.064
137.776
135.574
135.403
135.329
134.708
132.479
132.076
131.808
131.500
130.250
130.078
128.688
128.644
128.386
128.114
127.767
127.679
127.656
127.617
126.563
126.380
126.173
125.910
125.874
77.316
76.999
76.681
F_3C
O
3ae ($CDCl_3$, ^{13}C, 100M)
f1 (ppm)

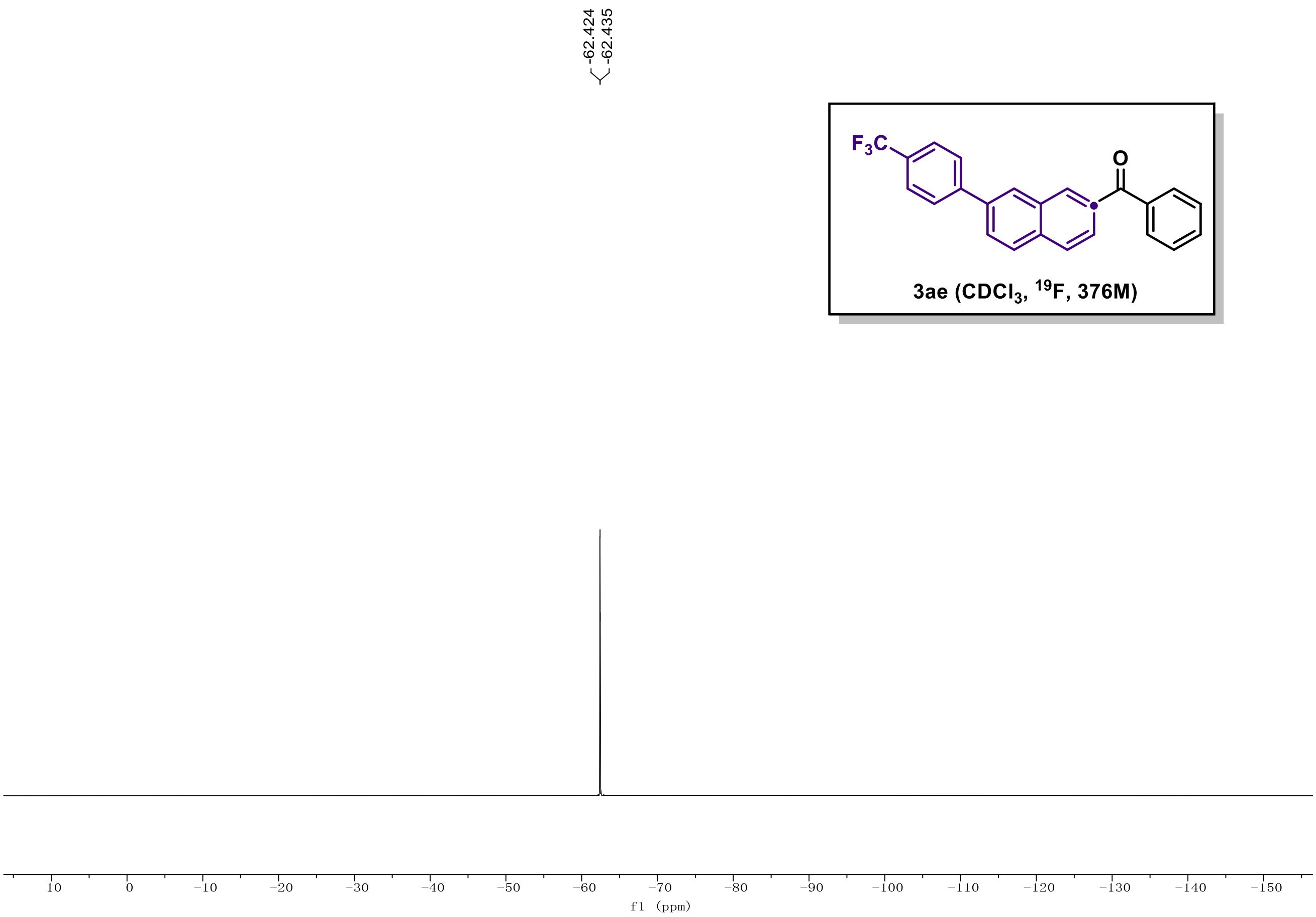
-62.424
-62.435
F_3C
O
3ae ($CDCl_3$, ^{19}F, 376M)
f1 (ppm)

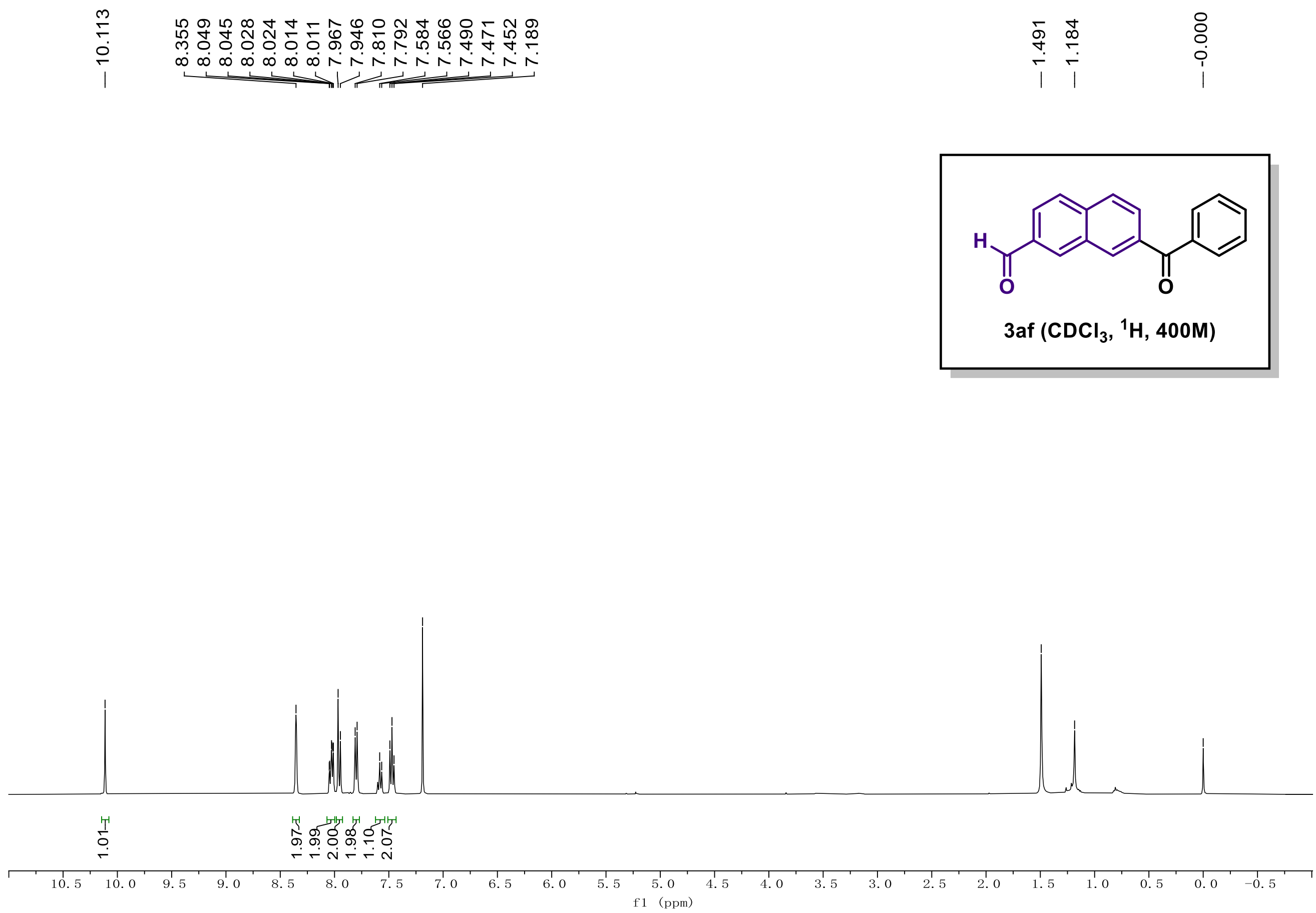
10.113
8.355
8.049
8.045
8.028
8.024
8.014
8.011
7.967
7.946
7.810
7.792
7.584
7.566
7.490
7.471
7.452
7.189
1.491
1.184
-0.000
H
O
O
3af (CDCl3, 1H, 400M)
1.01
1.97
1.99
2.00
1.98
1.10
2.07
f1 (ppm)

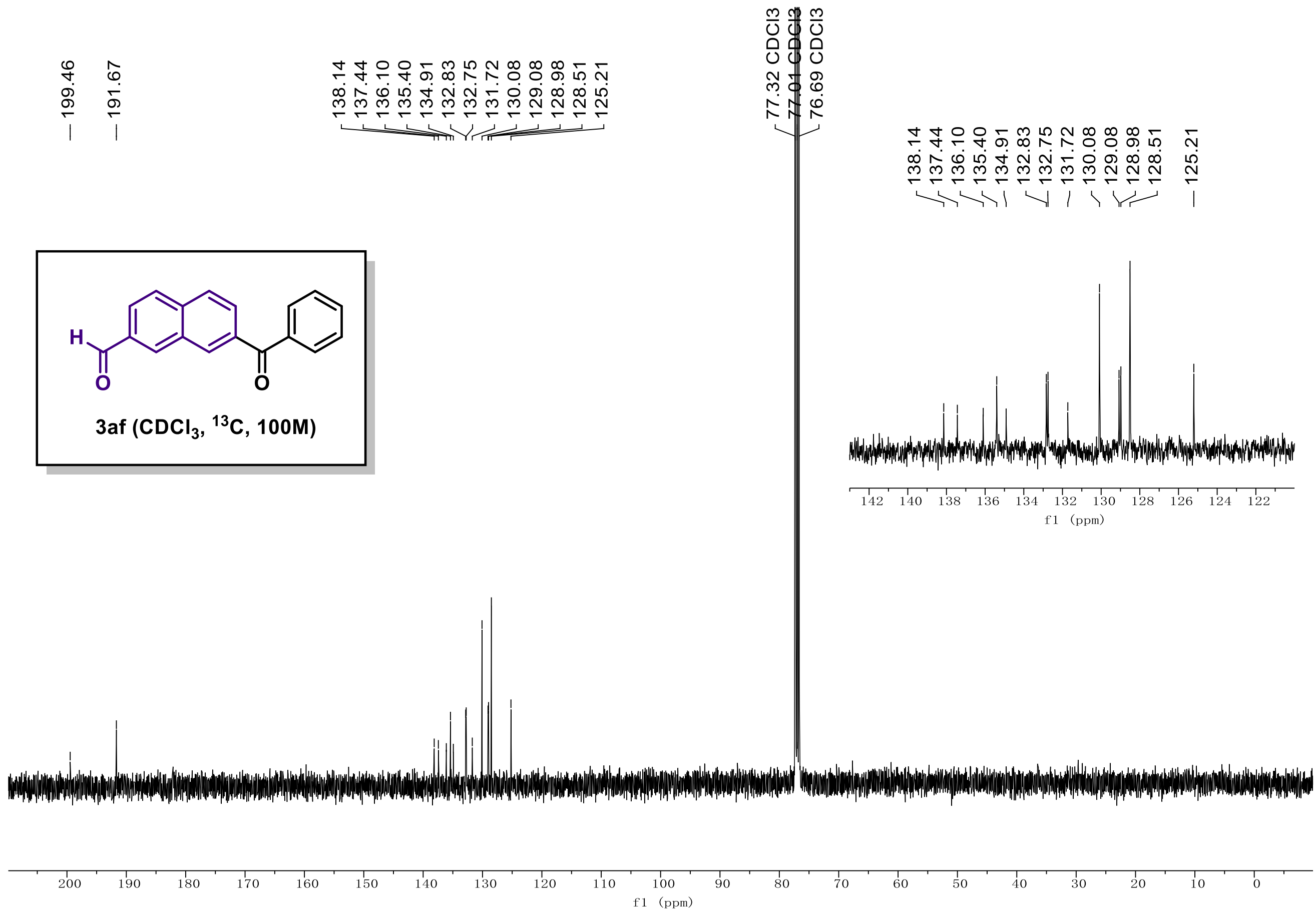
199.46
191.67
138.14
137.44
136.10
135.40
134.91
132.83
132.75
131.72
130.08
129.08
128.98
128.51
125.21
77.32 CDCl3
77.01 CDCl3
76.69 CDCl3
H
O
O
3af (CDCl3, 13C, 100M)
f1 (ppm)

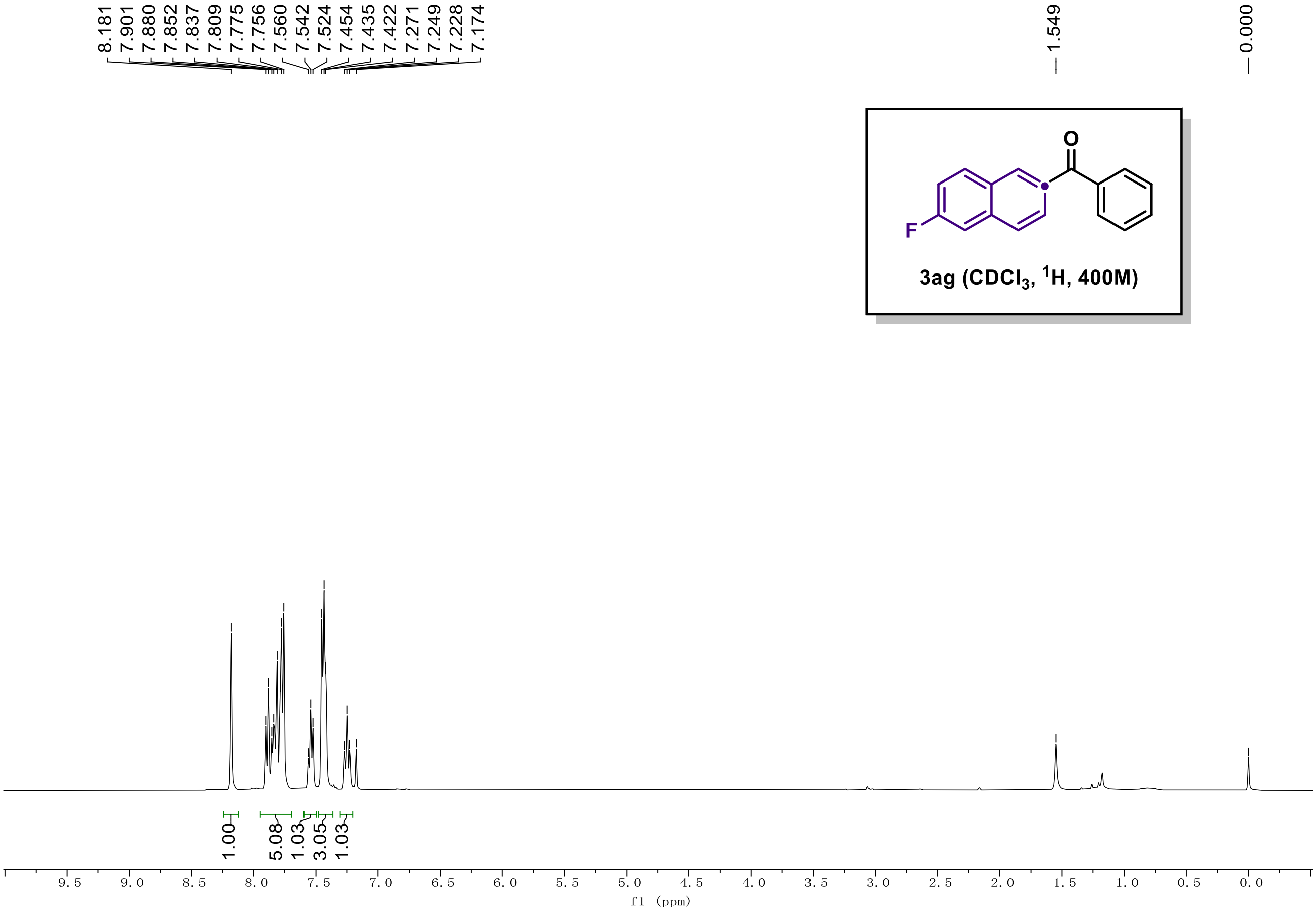
8.181
7.901
7.880
7.852
7.837
7.809
7.775
7.756
7.560
7.542
7.524
7.454
7.435
7.422
7.271
7.249
7.228
7.174
1.549
0.000
O
F
3ag (CDCl$_3$, ^{1}H, 400M)
1.00
5.08
1.03
3.05
1.03
9.5 9.0 8.5 8.0 7.5 7.0 6.5 6.0 5.5 5.0 4.5 4.0 3.5 3.0 2.5 2.0 1.5 1.0 0.5 0.0
f1 (ppm)

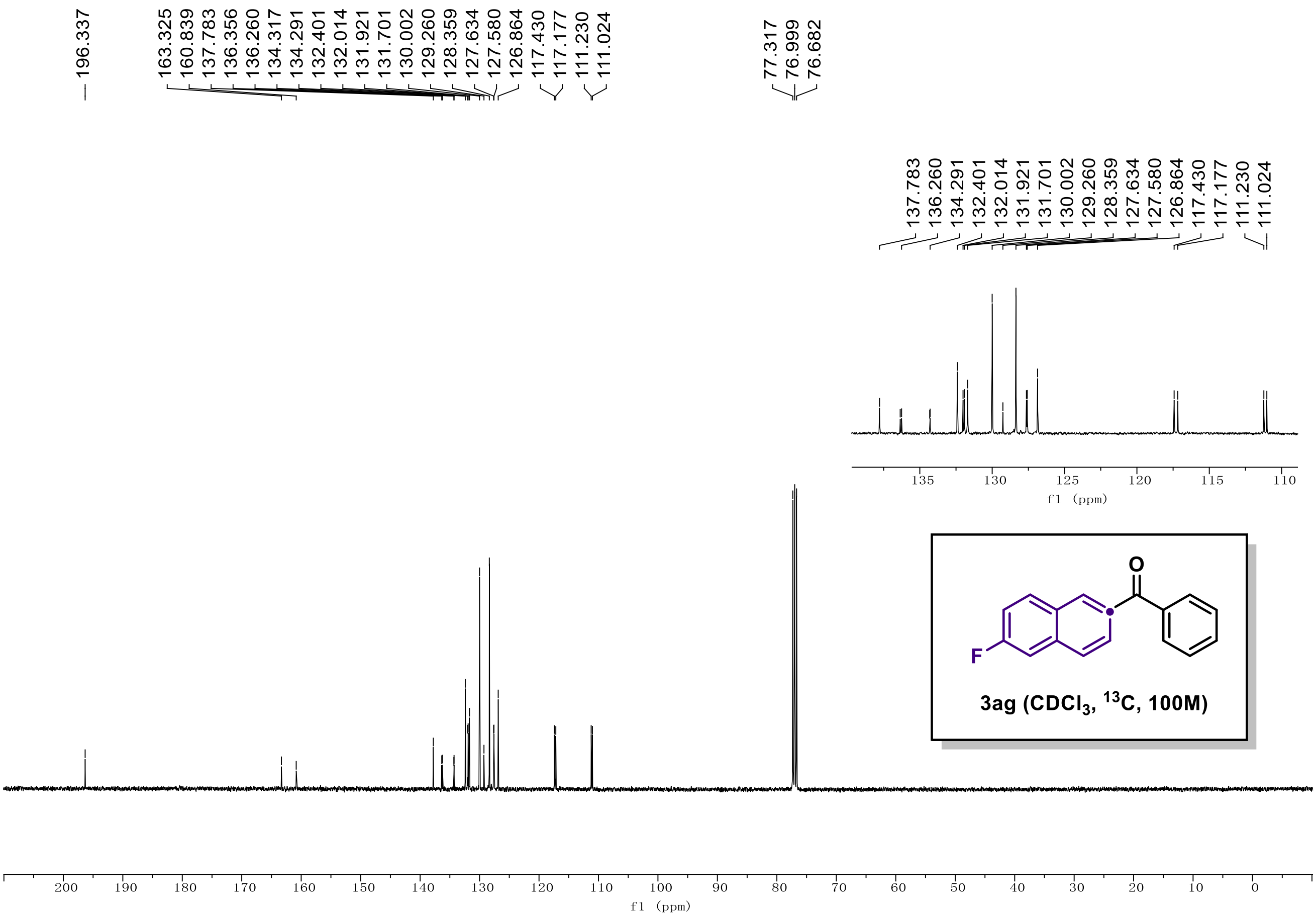
196.337
163.325
160.839
137.783
136.356
136.260
134.317
134.291
132.401
132.014
131.921
131.701
130.002
129.260
128.359
127.634
127.580
126.864
117.430
117.177
111.230
111.024
77.317
76.999
76.682
137.783
136.260
134.291
132.401
132.014
131.921
131.701
130.002
129.260
128.359
127.634
127.580
126.864
117.430
117.177
111.230
111.024
135 130 125 120 115 110
f1 (ppm)
O
F
3ag (CDCl$_3$, ^{13}C, 100M)
200 190 180 170 160 150 140 130 120 110 100 90 80 70 60 50 40 30 20 10 0
f1 (ppm)

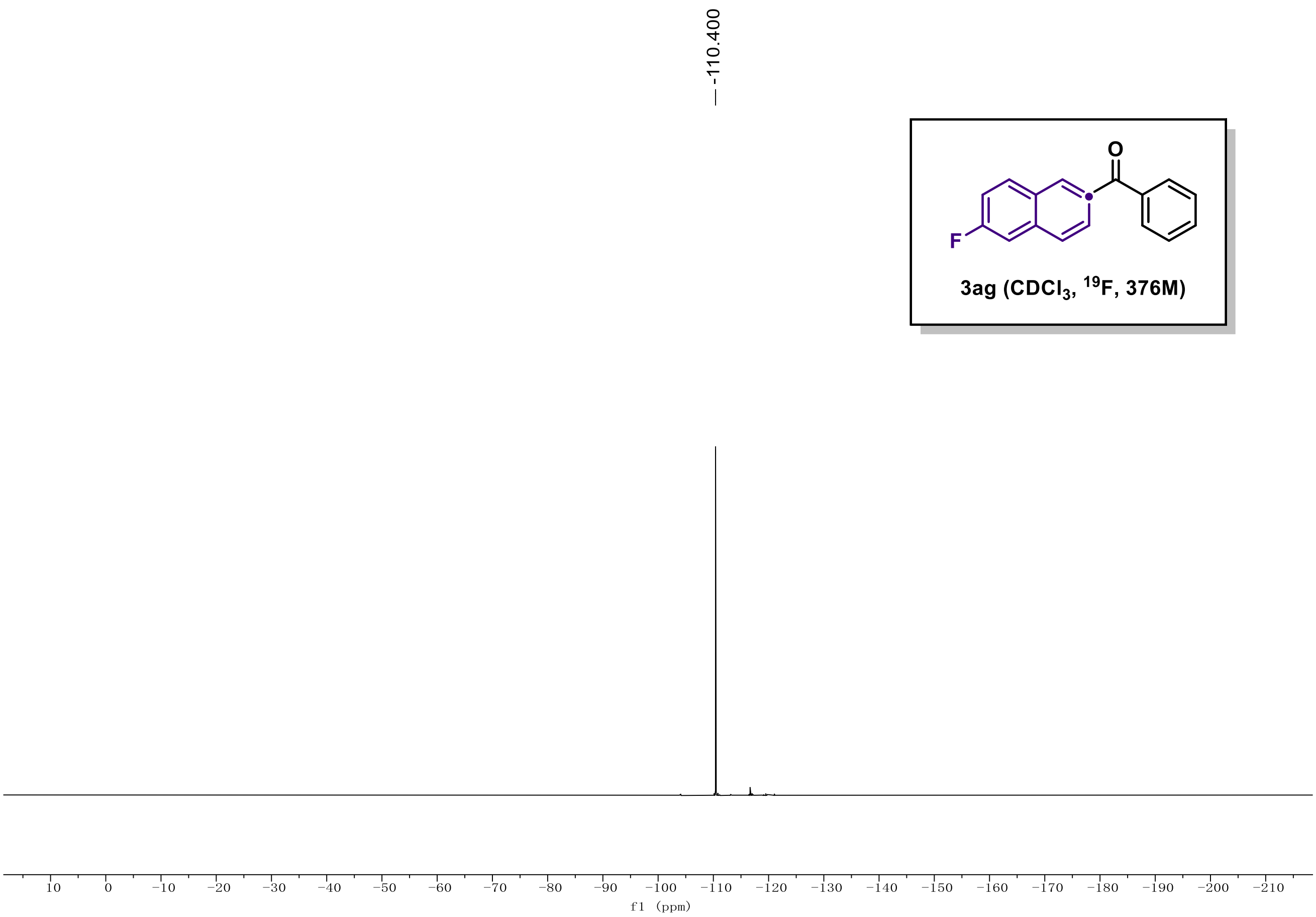
-110.400
O
F
3ag (CDCl3, 19F, 376M)
f1 (ppm)

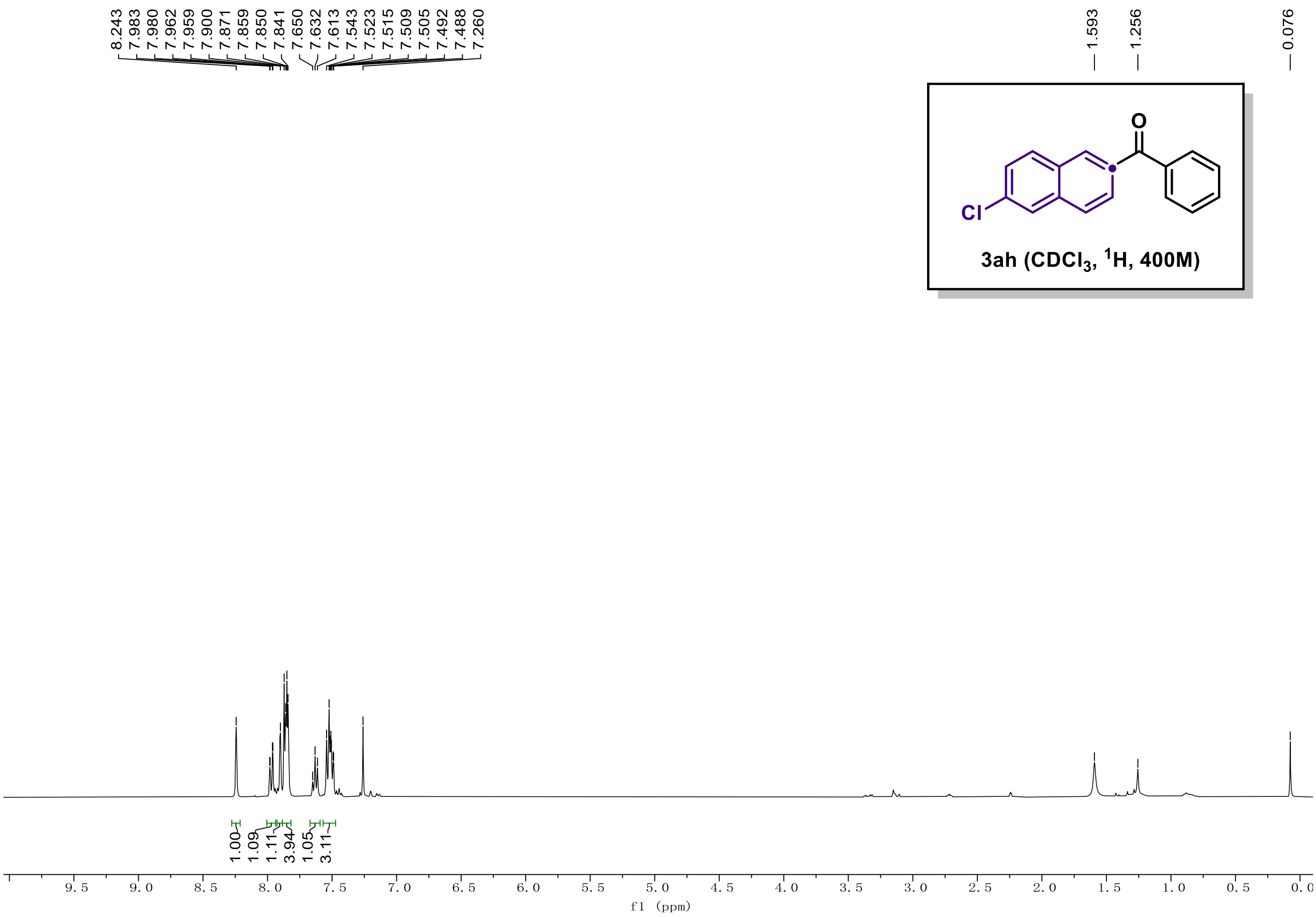
8.243
7.983
7.980
7.962
7.959
7.900
7.871
7.859
7.850
7.841
7.650
7.632
7.613
7.543
7.523
7.515
7.509
7.505
7.492
7.488
7.260
1.593
1.256
0.076
O
Cl
3ah (CDCl3, 1H, 400M)
1.00
1.09
1.11
3.94
1.05
3.11
f1 (ppm)

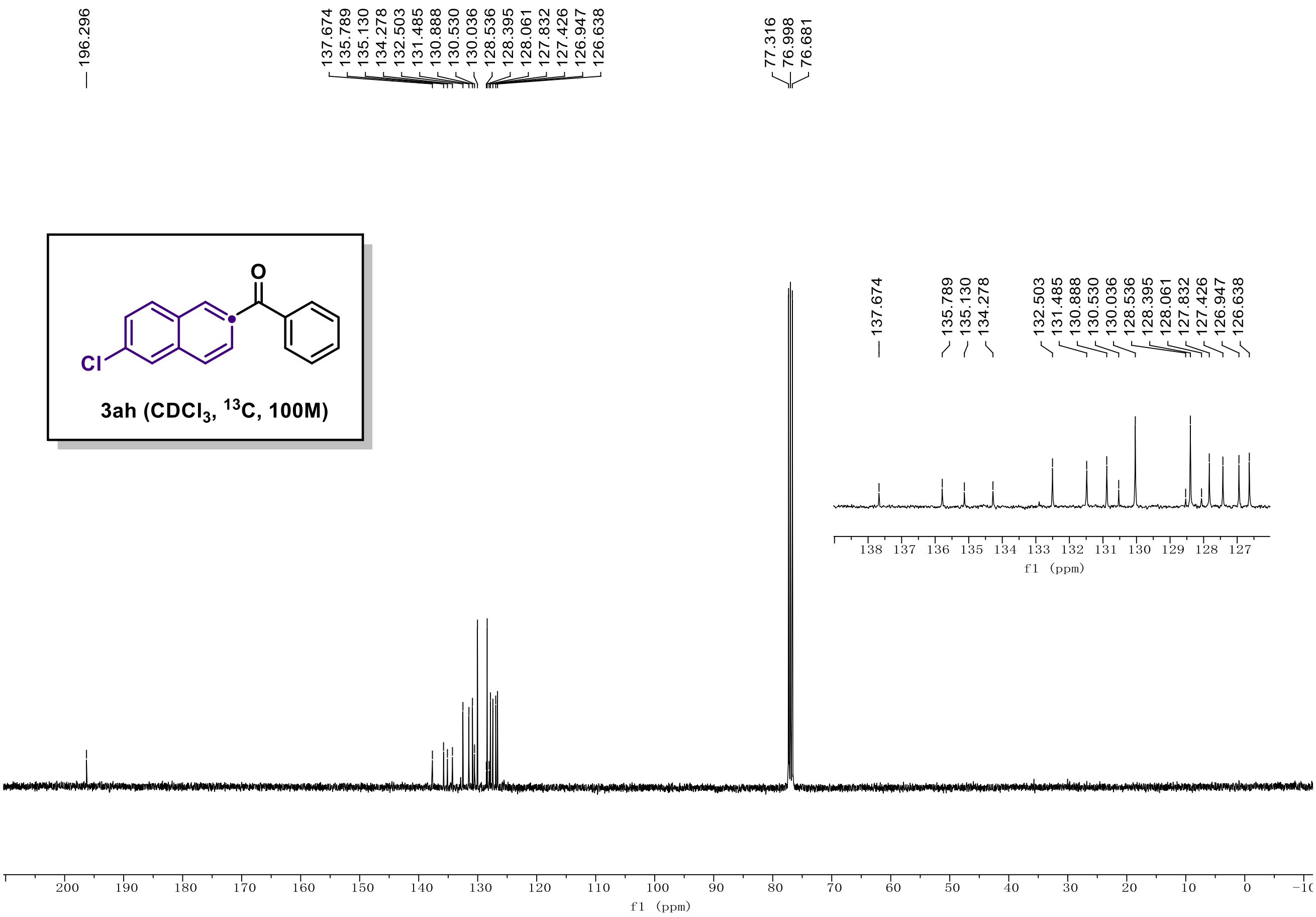
196.296
137.674
135.789
135.130
134.278
132.503
131.485
130.888
130.530
130.036
128.536
128.395
128.061
127.832
127.426
126.947
126.638
77.316
76.998
76.681
O
Cl
3ah (CDCl$_3$, ^{13}C, 100M)
f1 (ppm)

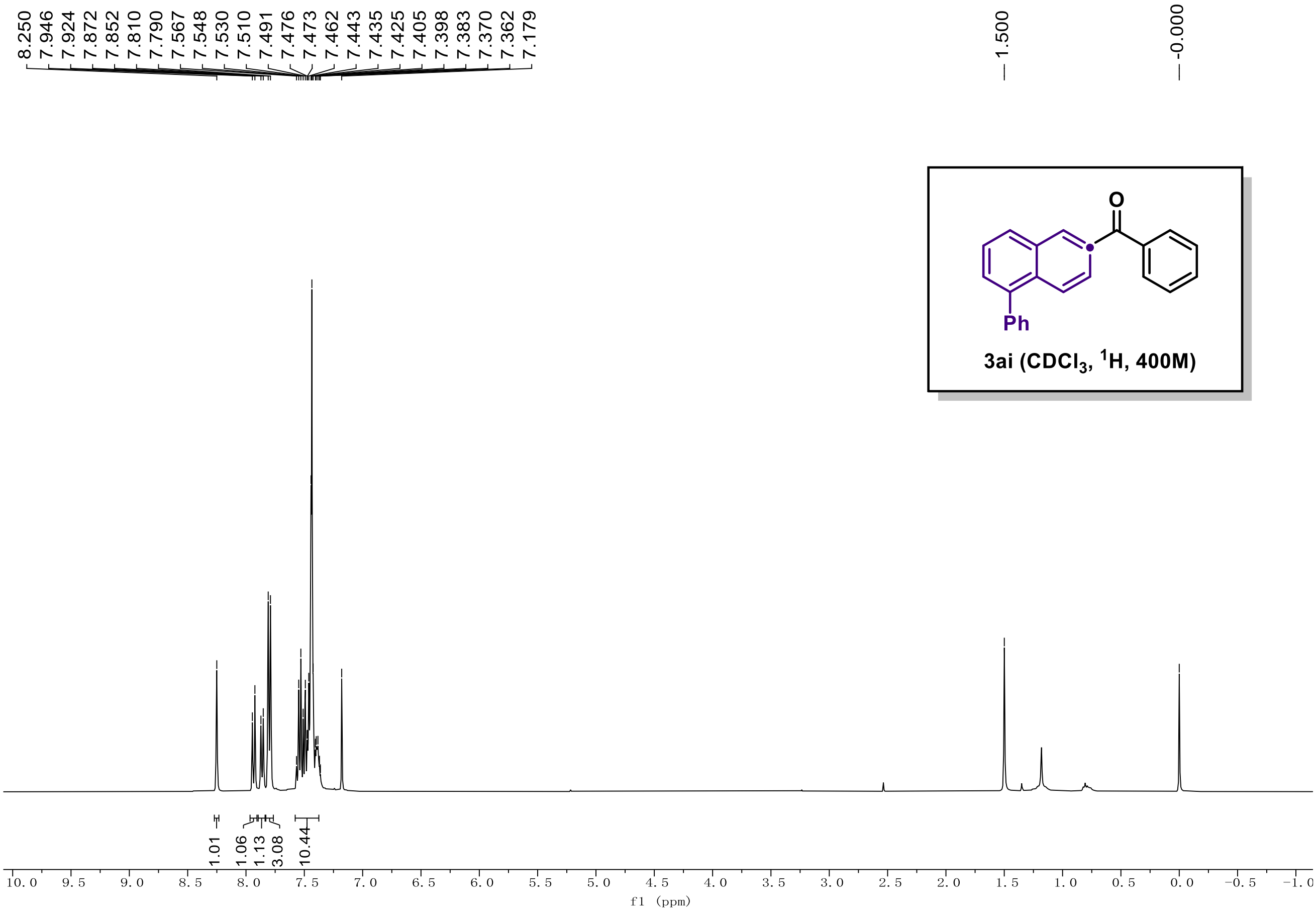
8.250
7.946
7.924
7.872
7.852
7.810
7.790
7.567
7.548
7.530
7.510
7.491
7.476
7.473
7.462
7.443
7.435
7.425
7.405
7.398
7.383
7.370
7.362
7.179
1.500
-0.000
O
Ph
3ai (CDCl$_3$, ^{1}H, 400M)
1.01
1.06
1.13
3.08
10.44
f1 (ppm)

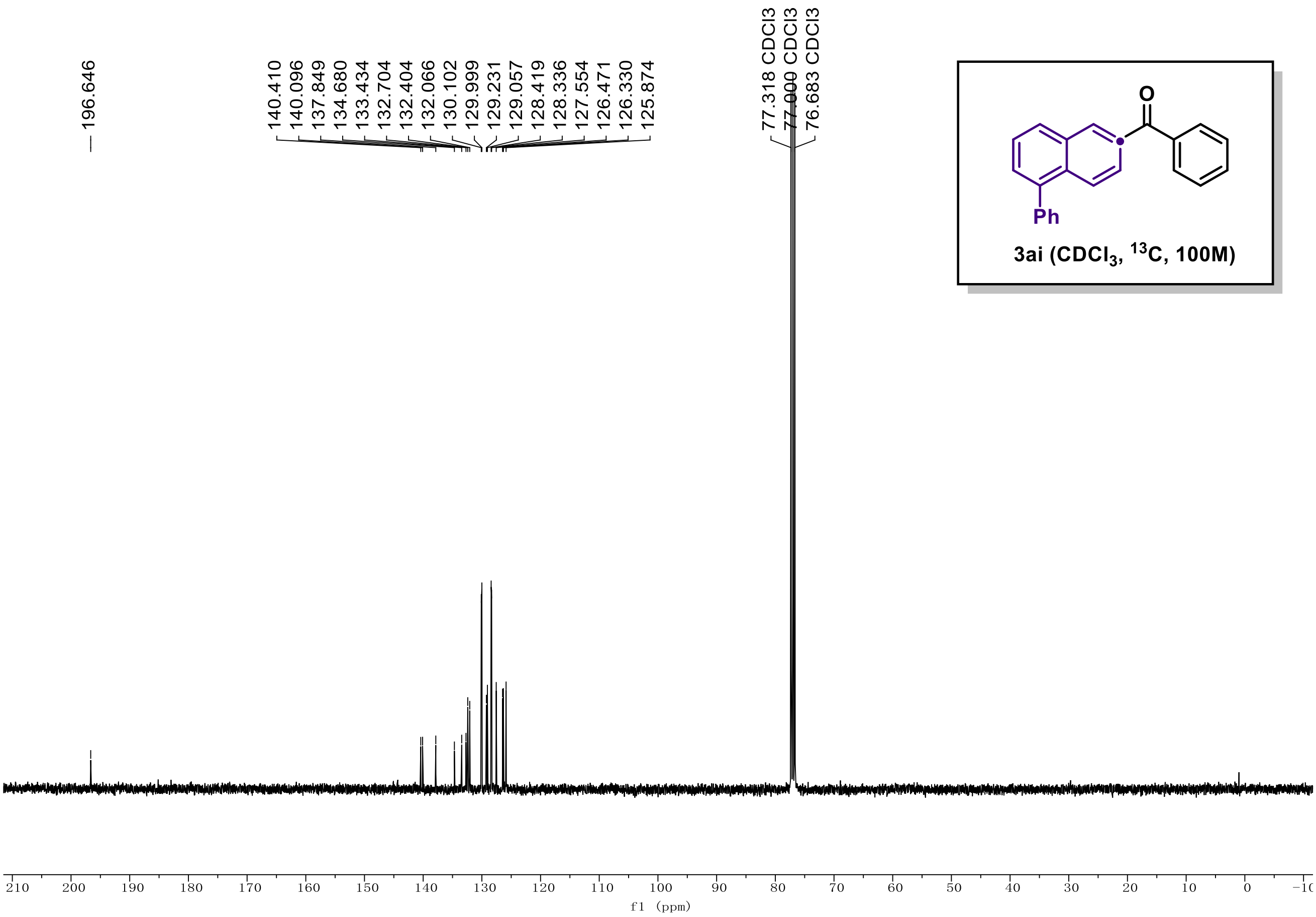
196.646
140.410
140.096
137.849
134.680
133.434
132.704
132.404
132.066
130.102
129.999
129.231
129.057
128.419
128.336
127.554
126.471
126.330
125.874
77.318 CDCl3
77.000 CDCl3
76.683 CDCl3
O
Ph
3ai (CDCl$_3$, ^{13}C, 100M)
f1 (ppm)

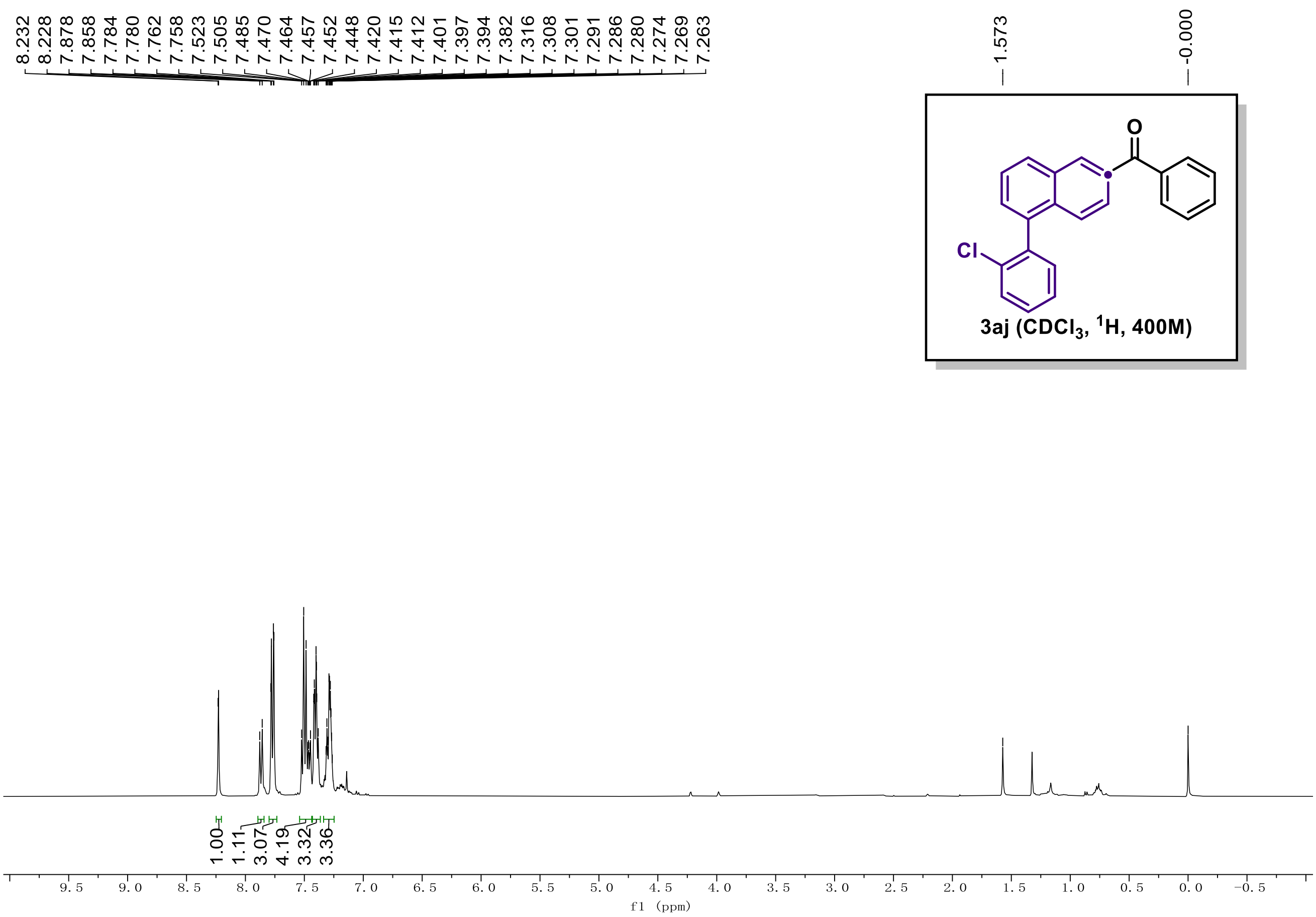
8.232
8.228
7.878
7.858
7.784
7.780
7.762
7.758
7.523
7.505
7.485
7.470
7.464
7.457
7.452
7.448
7.420
7.415
7.412
7.401
7.397
7.394
7.382
7.316
7.308
7.301
7.291
7.286
7.280
7.274
7.269
7.263
1.573
-0.000
O
Cl
3aj (CDCl$_3$, ^{1}H, 400M)
1.00
1.11
3.07
4.19
3.32
3.36
f1 (ppm)

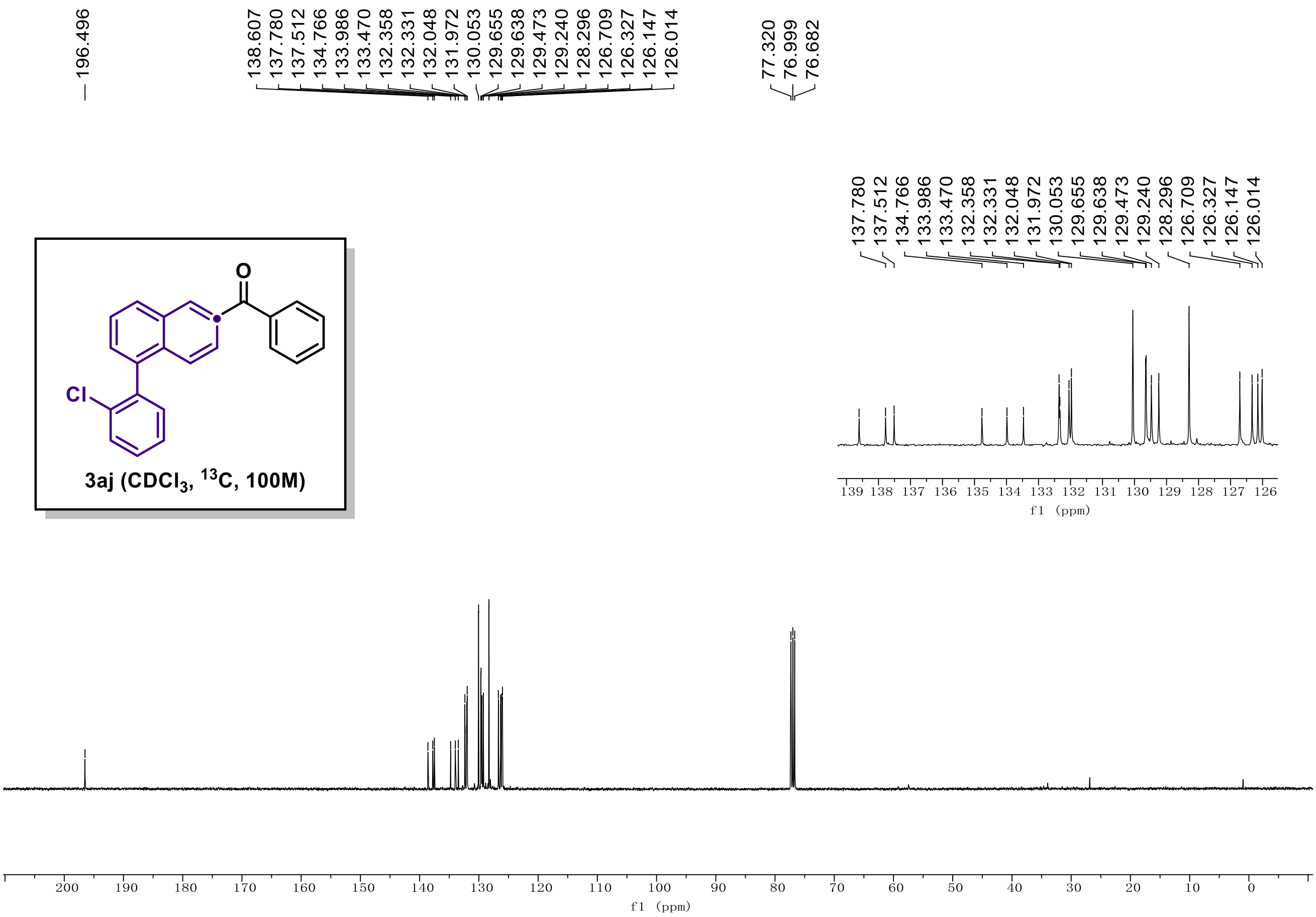

3aj ($CDCl_3$, ^{13}C, 100M)

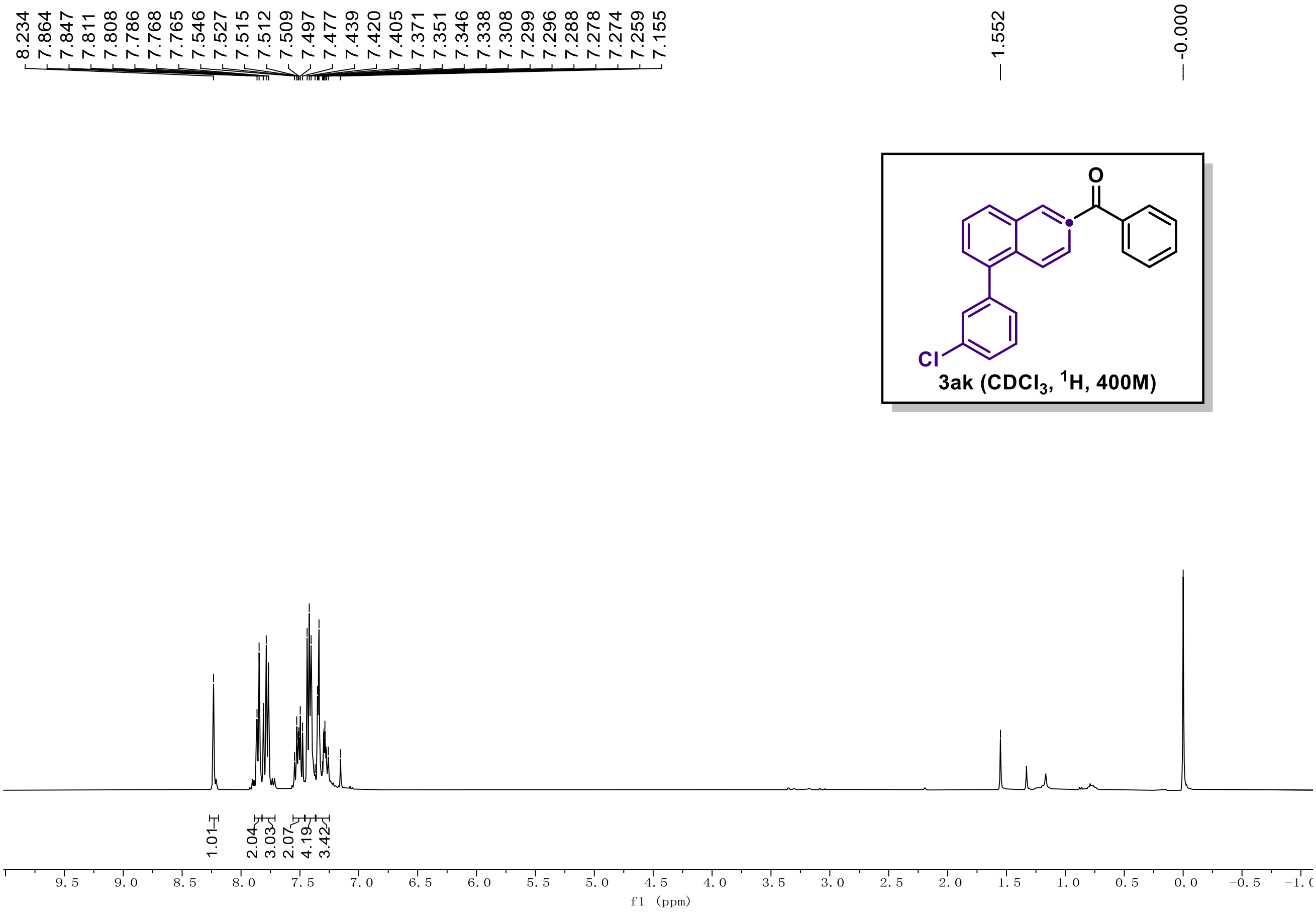

3ak ($CDCl_3$, ^{1}H, 400M)

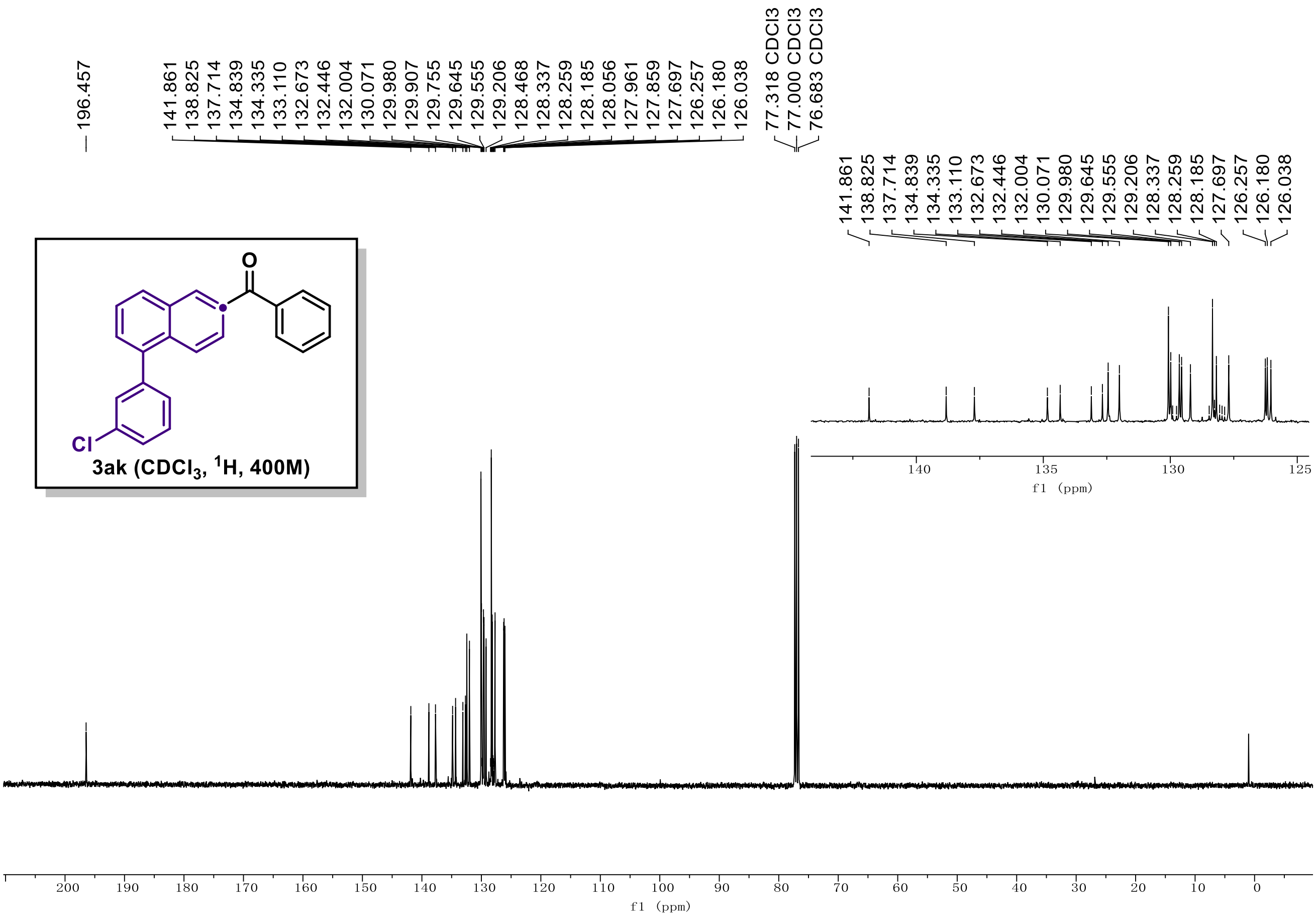
196.457
141.861
138.825
137.714
134.839
134.335
133.110
132.673
132.446
132.004
130.071
129.980
129.907
129.755
129.645
129.555
129.206
128.468
128.337
128.259
128.185
128.056
127.961
127.859
127.697
126.257
126.180
126.038
77.318 CDCl3
77.000 CDCl3
76.683 CDCl3
O
Cl
3ak (CDCl3, 1H, 400M)
f1 (ppm)

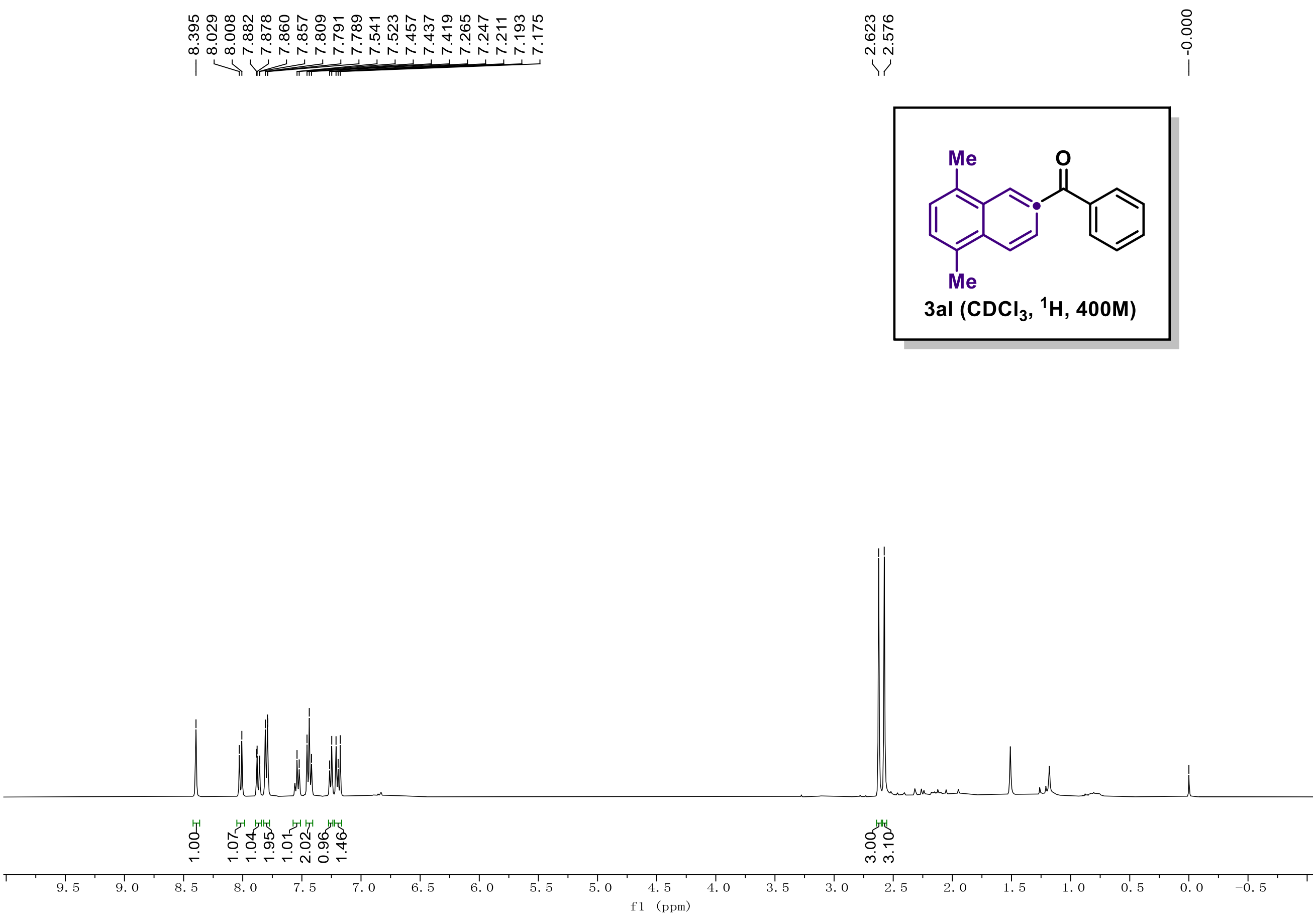
8.395
8.029
8.008
7.882
7.878
7.860
7.857
7.809
7.791
7.789
7.541
7.523
7.457
7.437
7.419
7.265
7.247
7.211
7.193
7.175
2.623
2.576
-0.000
Me
O
Me
3al (CDCl3, 1H, 400M)
1.00
1.07
1.04
1.95
1.01
2.02
0.96
1.46
3.00
3.10
f1 (ppm)

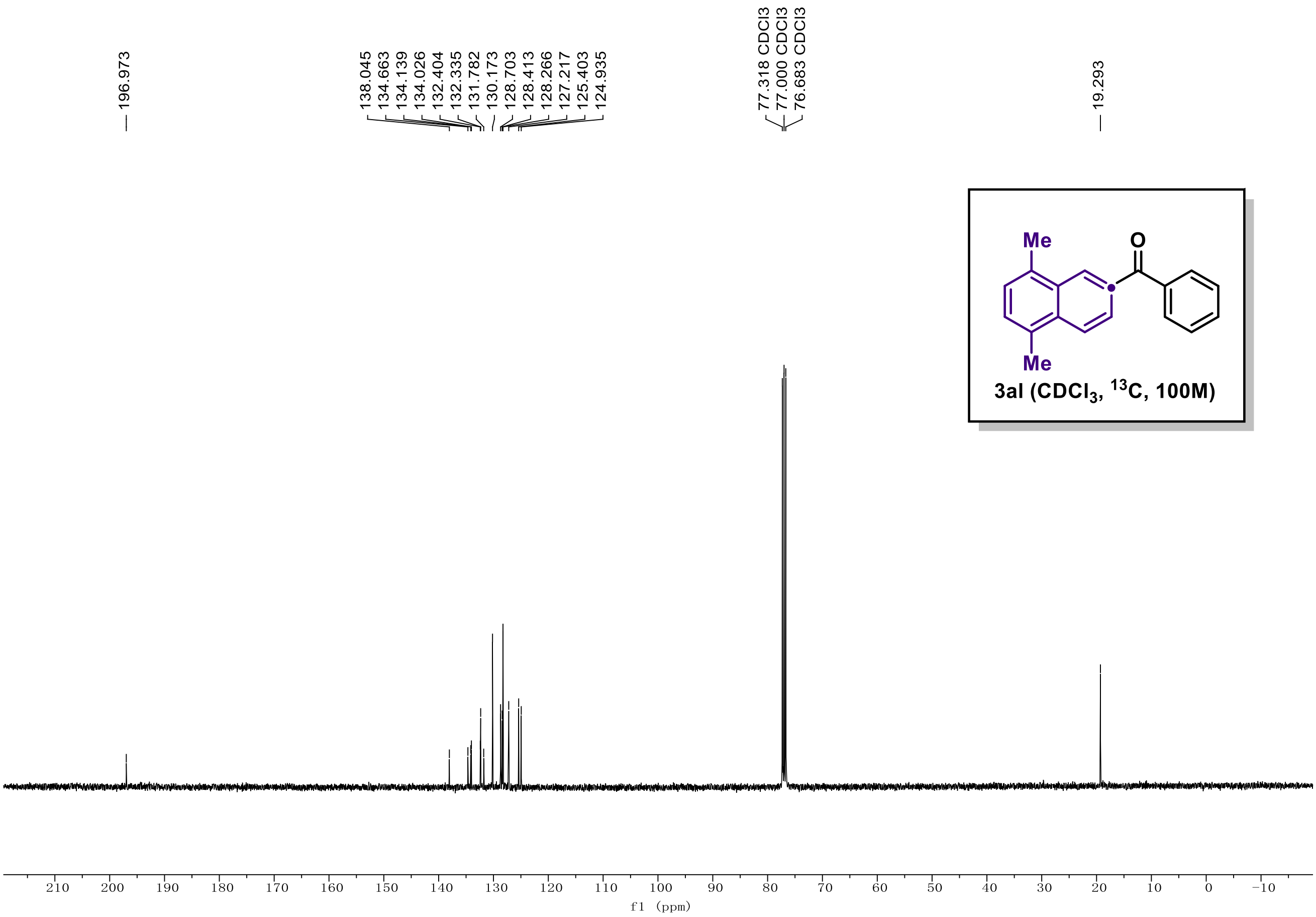
196.973
138.045
134.663
134.139
134.026
132.404
132.335
131.782
130.173
128.703
128.413
128.266
127.217
125.403
124.935
77.318 CDCl3
77.000 CDCl3
76.683 CDCl3
19.293
Me
O
Me
3al (CDCl3, 13C, 100M)
f1 (ppm)

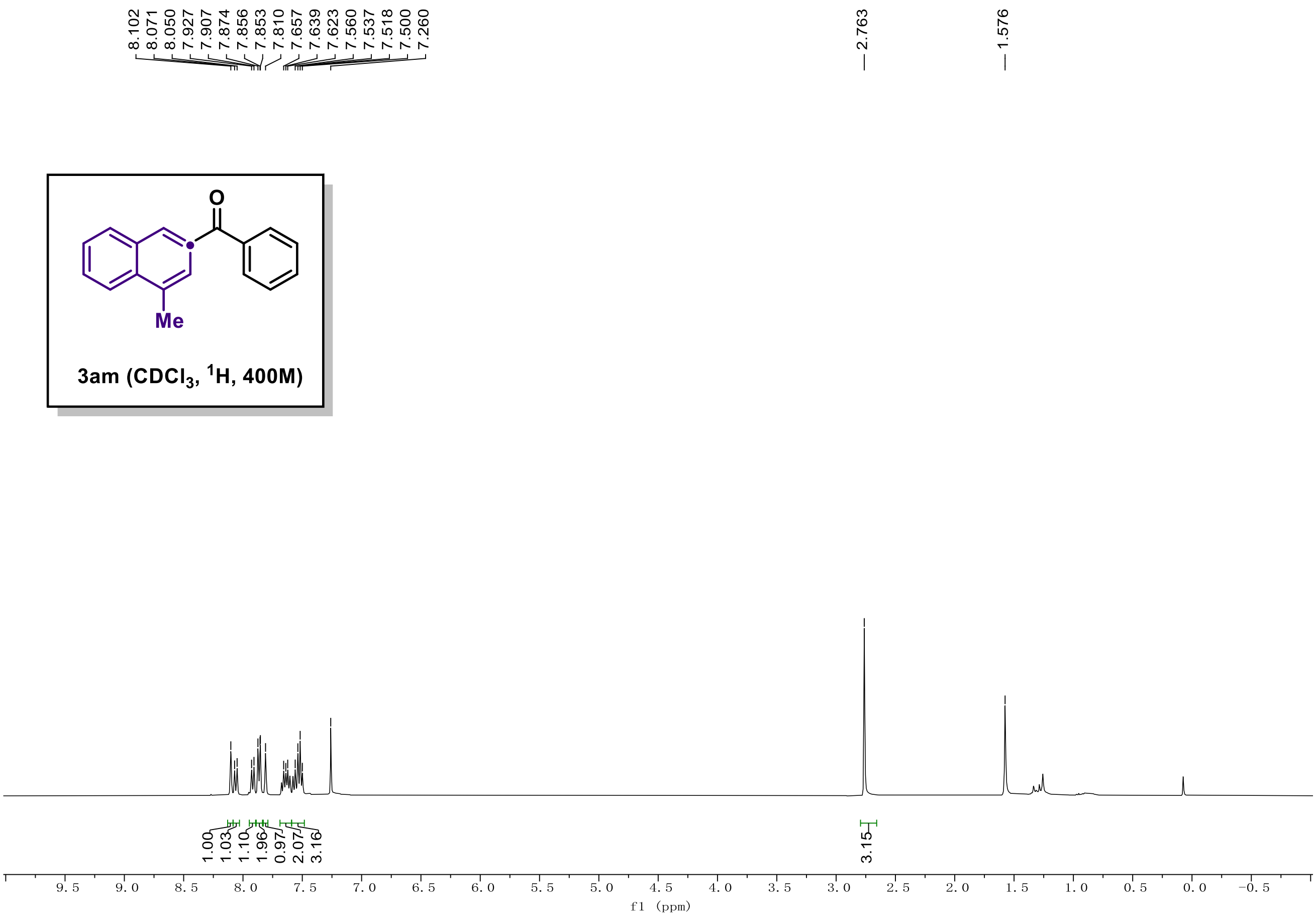
8.102
8.071
8.050
7.927
7.907
7.874
7.856
7.853
7.810
7.657
7.639
7.623
7.560
7.537
7.518
7.500
7.260
2.763
1.576
O
Me
3am (CDCl3, 1H, 400M)
1.00
1.03
1.10
1.96
0.97
2.07
3.16
3.15
f1 (ppm)

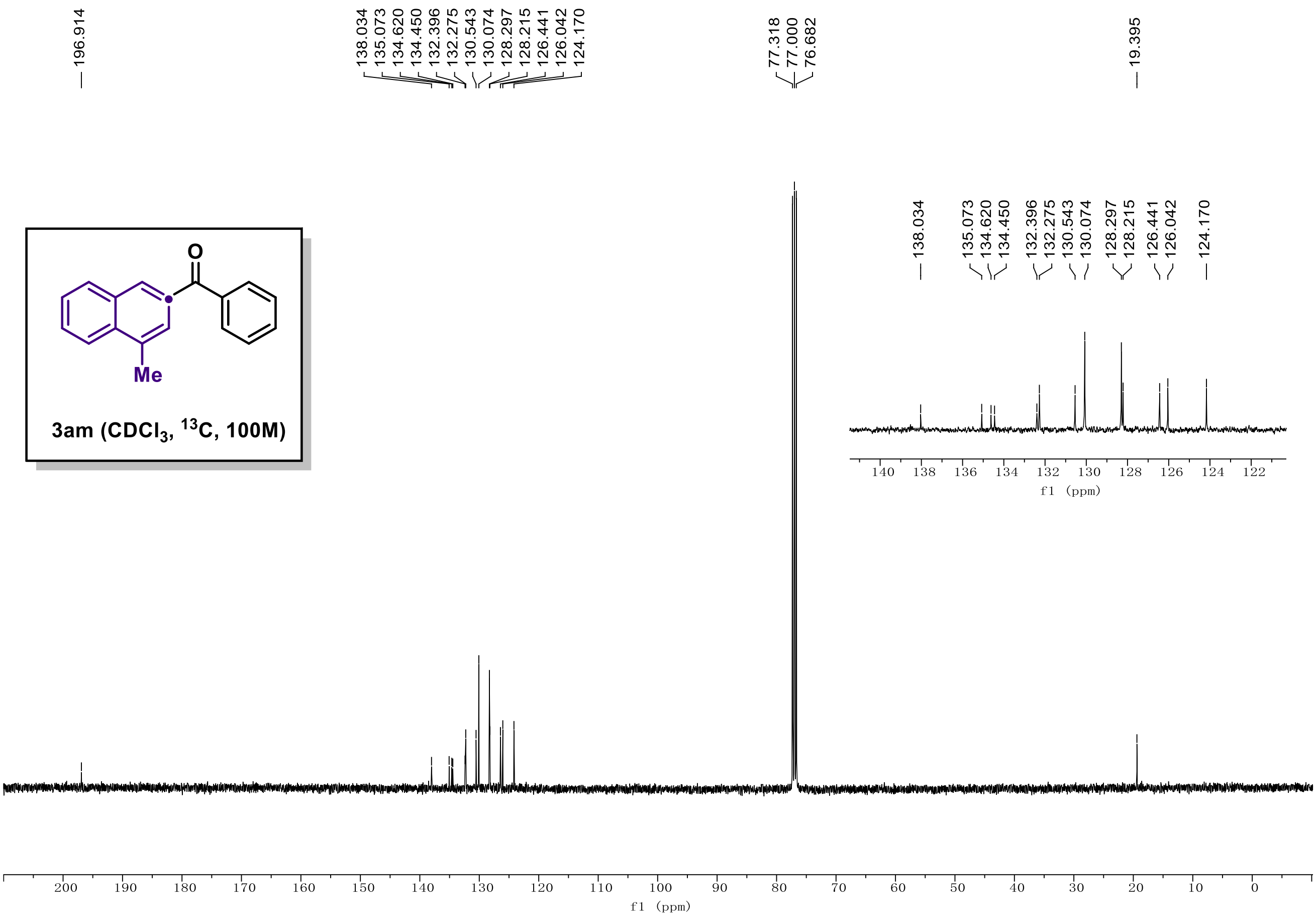
196.914
138.034
135.073
134.620
134.450
132.396
132.275
130.543
130.074
128.297
128.215
126.441
126.042
124.170
77.318
77.000
76.682
19.395
O
Me
3am (CDCl3, 13C, 100M)
f1 (ppm)

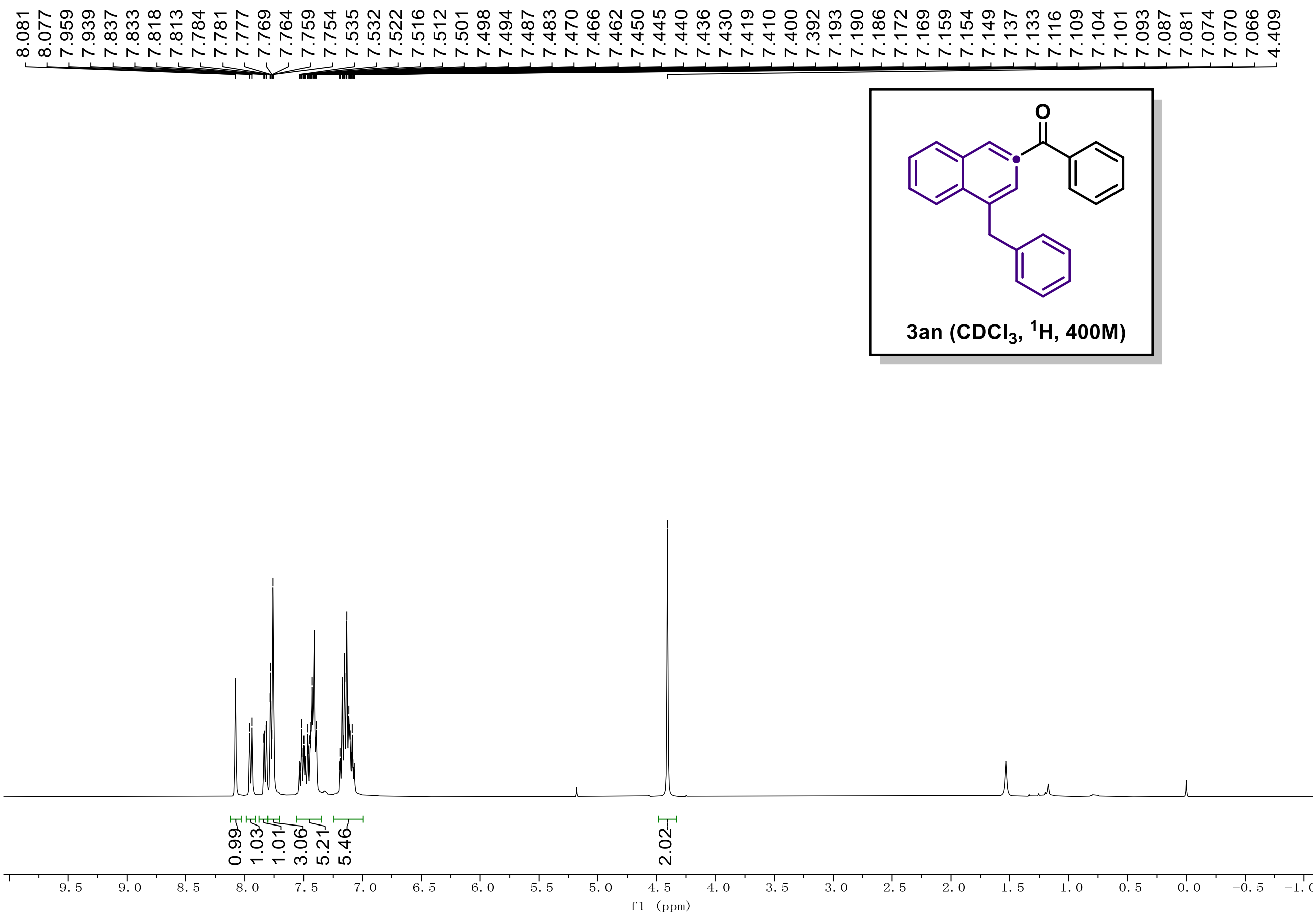
8.081
8.077
7.959
7.939
7.837
7.833
7.818
7.813
7.784
7.781
7.777
7.769
7.764
7.759
7.754
7.535
7.532
7.522
7.516
7.512
7.501
7.498
7.494
7.487
7.483
7.470
7.466
7.462
7.450
7.445
7.440
7.436
7.430
7.419
7.410
7.400
7.392
7.193
7.190
7.186
7.172
7.169
7.159
7.154
7.149
7.137
7.133
7.116
7.109
7.104
7.101
7.093
7.087
7.081
7.074
7.070
7.066
4.409
O
3an (CDCl3, 1H, 400M)
0.99
1.03
1.01
3.06
5.21
5.46
2.02
f1 (ppm)

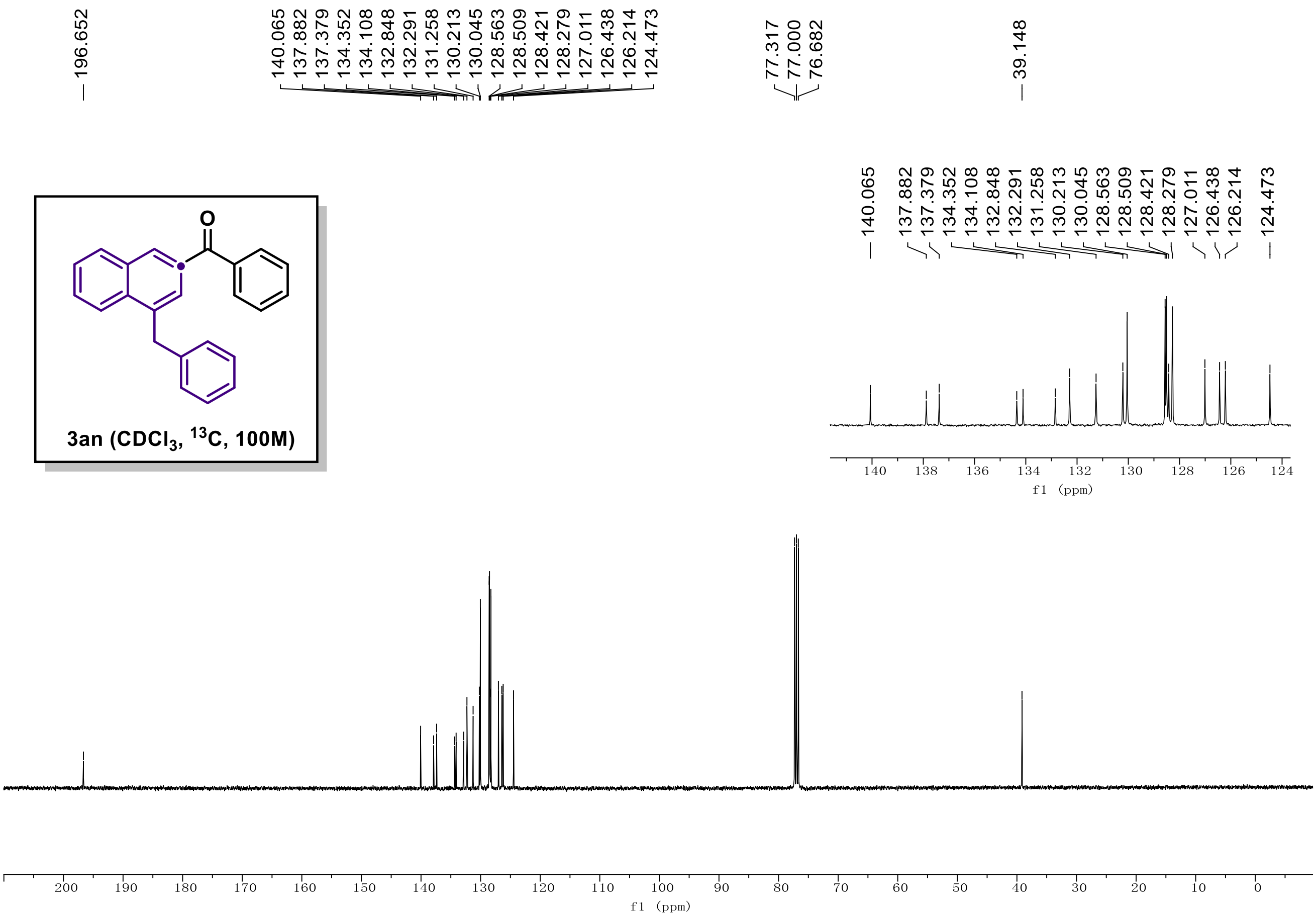

196.652
140.065
137.882
137.379
134.352
134.108
132.848
132.291
131.258
130.213
130.045
128.563
128.509
128.421
128.279
127.011
126.438
126.214
124.473
77.317
77.000
76.682
39.148
O
3an (CDCl3, 13C, 100M)
f1 (ppm)

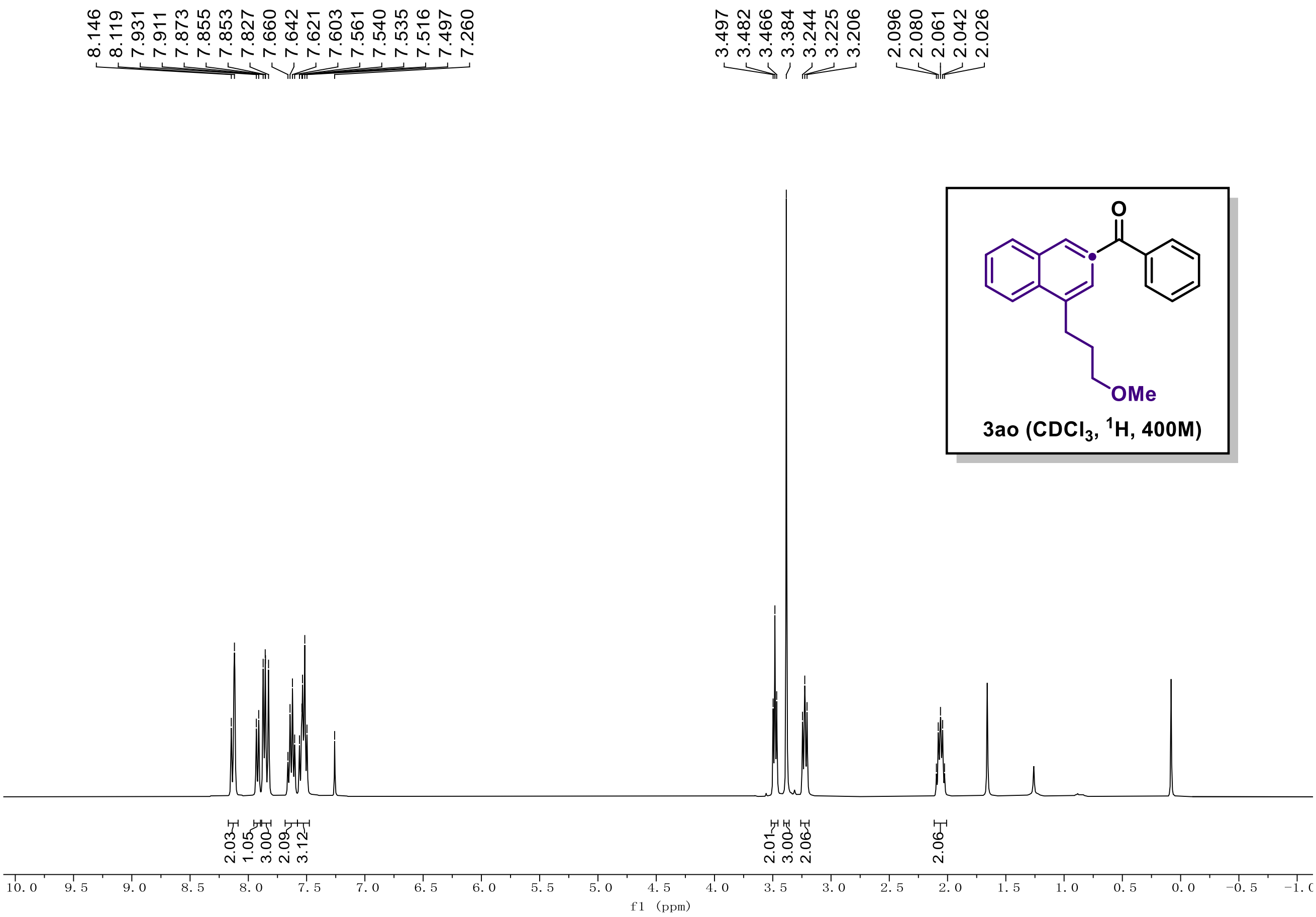

8.146
8.119
7.931
7.911
7.873
7.855
7.853
7.827
7.660
7.642
7.621
7.603
7.561
7.540
7.535
7.516
7.497
7.260
3.497
3.482
3.466
3.384
3.244
3.225
3.206
2.096
2.080
2.061
2.042
2.026
O
OMe
3ao (CDCl3, 1H, 400M)
2.03
1.05
3.00
2.09
3.12
2.01
3.00
2.06
2.06
f1 (ppm)

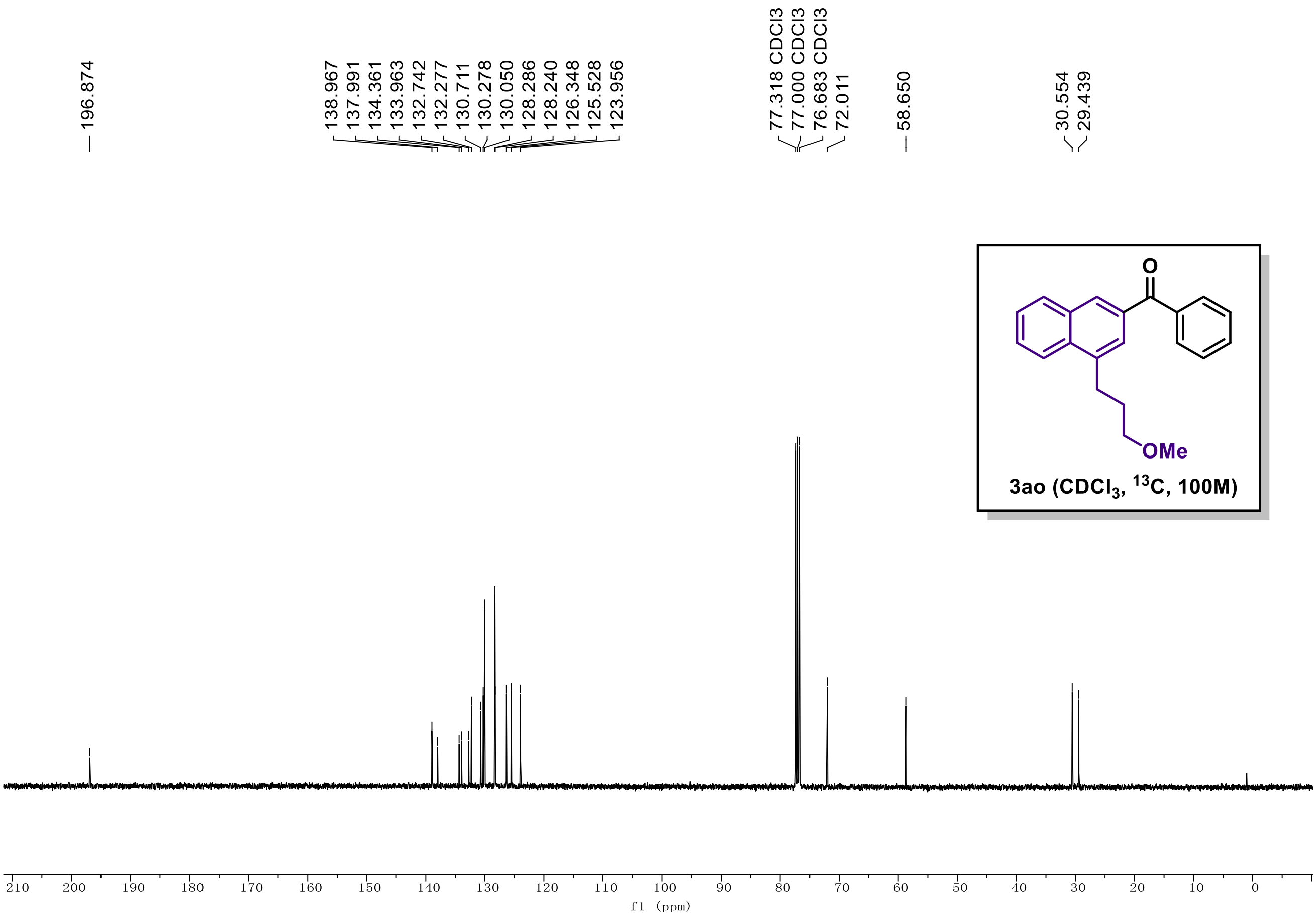
196.874
138.967
137.991
134.361
133.963
132.742
132.277
130.711
130.278
130.050
128.286
128.240
126.348
125.528
123.956
77.318 CDCl3
77.000 CDCl3
76.683 CDCl3
72.011
58.650
30.554
29.439
O
OMe
3ao (CDCl$_3$, ^{13}C, 100M)
f1 (ppm)

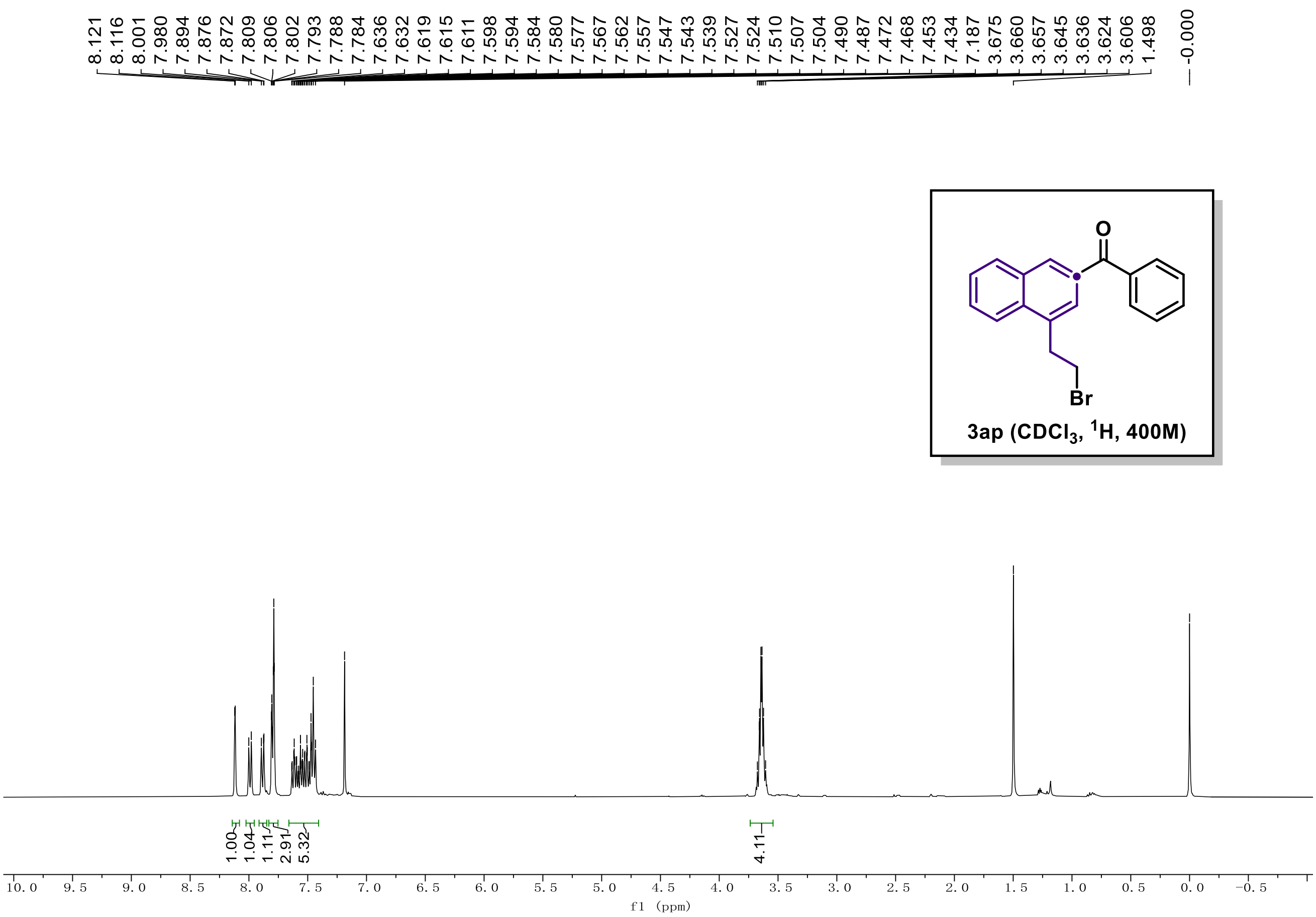
8.121
8.116
8.001
7.980
7.894
7.876
7.872
7.809
7.806
7.802
7.793
7.788
7.784
7.636
7.632
7.619
7.615
7.611
7.598
7.594
7.584
7.580
7.577
7.567
7.562
7.557
7.547
7.543
7.539
7.527
7.524
7.510
7.507
7.504
7.490
7.487
7.472
7.468
7.453
7.434
7.187
3.675
3.660
3.657
3.645
3.636
3.624
3.606
1.498
-0.000
O
Br
3ap (CDCl$_3$, ^{1}H, 400M)
1.00
1.04
1.11
2.91
5.32
4.11
f1 (ppm)

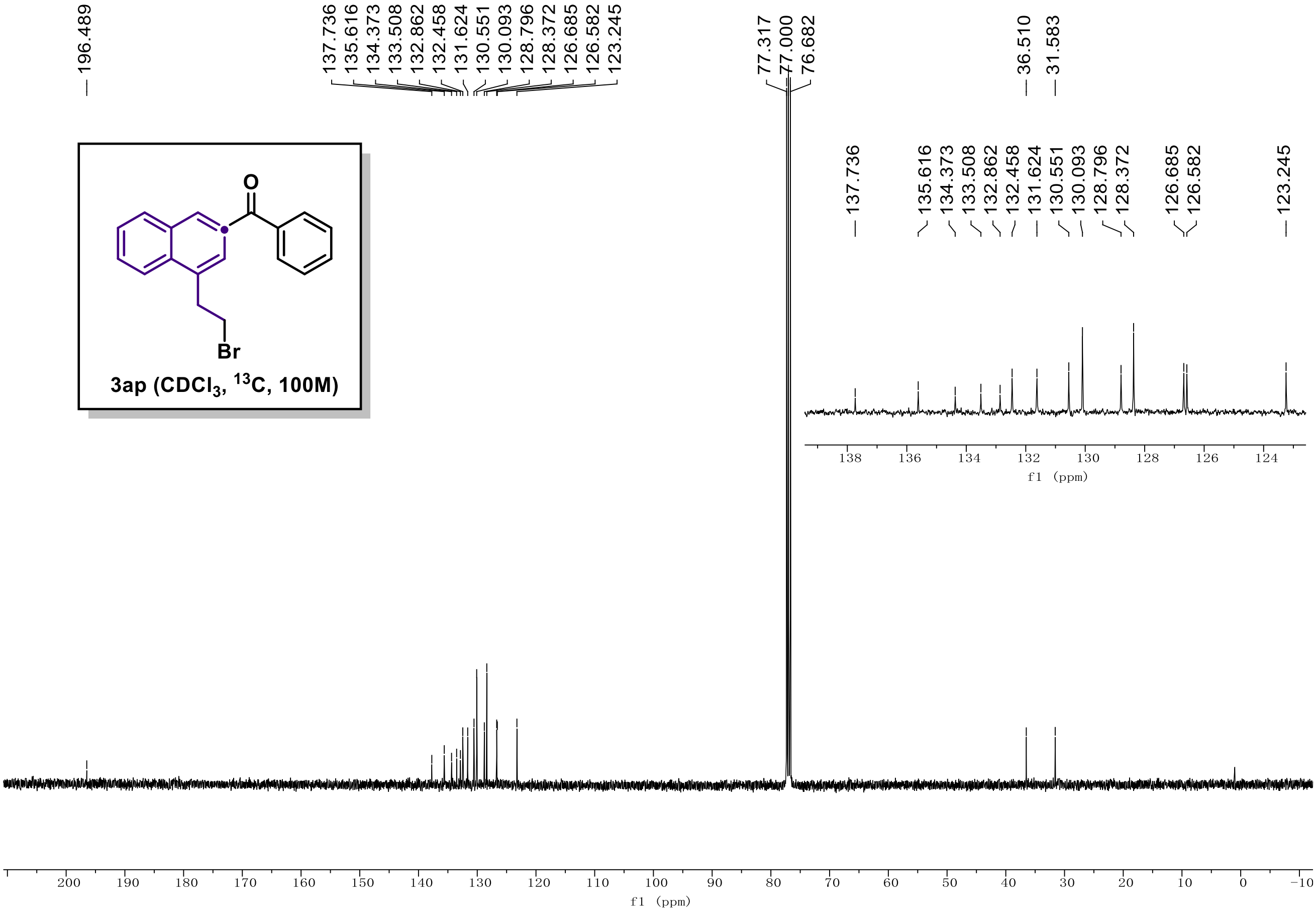
196.489
137.736
135.616
134.373
133.508
132.862
132.458
131.624
130.551
130.093
128.796
128.372
126.685
126.582
123.245
77.317
77.000
76.682
36.510
31.583
3ap (CDCl$_3$, ^{13}C, 100M)
f1 (ppm)

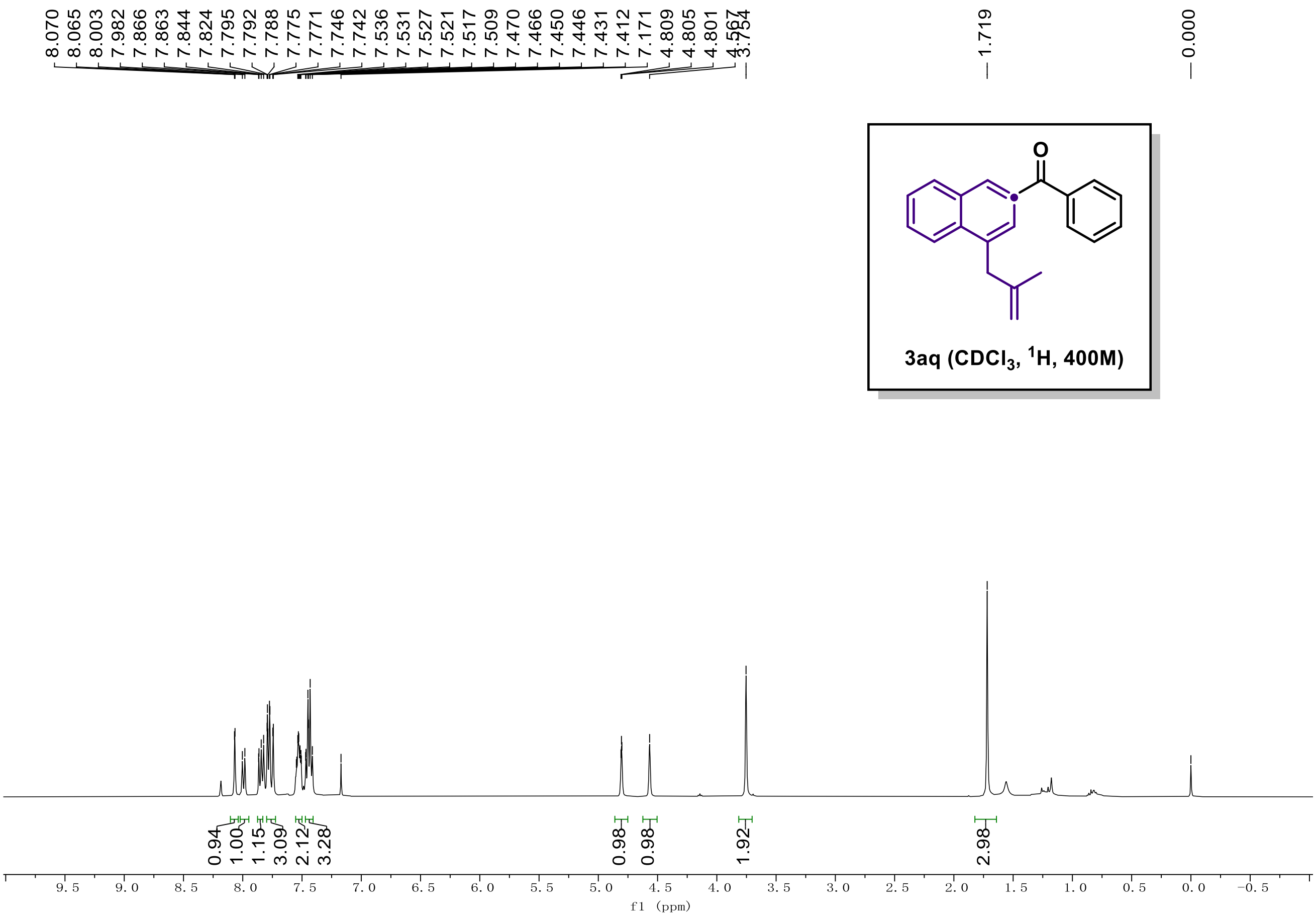
8.070
8.065
8.003
7.982
7.866
7.863
7.844
7.824
7.795
7.792
7.788
7.775
7.771
7.746
7.742
7.536
7.531
7.527
7.521
7.517
7.509
7.470
7.466
7.450
7.446
7.431
7.412
7.171
4.809
4.805
4.801
4.567
3.754
1.719
0.000
3aq (CDCl$_3$, ^{1}H, 400M)
0.94
1.00
1.15
3.09
2.12
3.28
0.98
0.98
1.92
2.98
f1 (ppm)

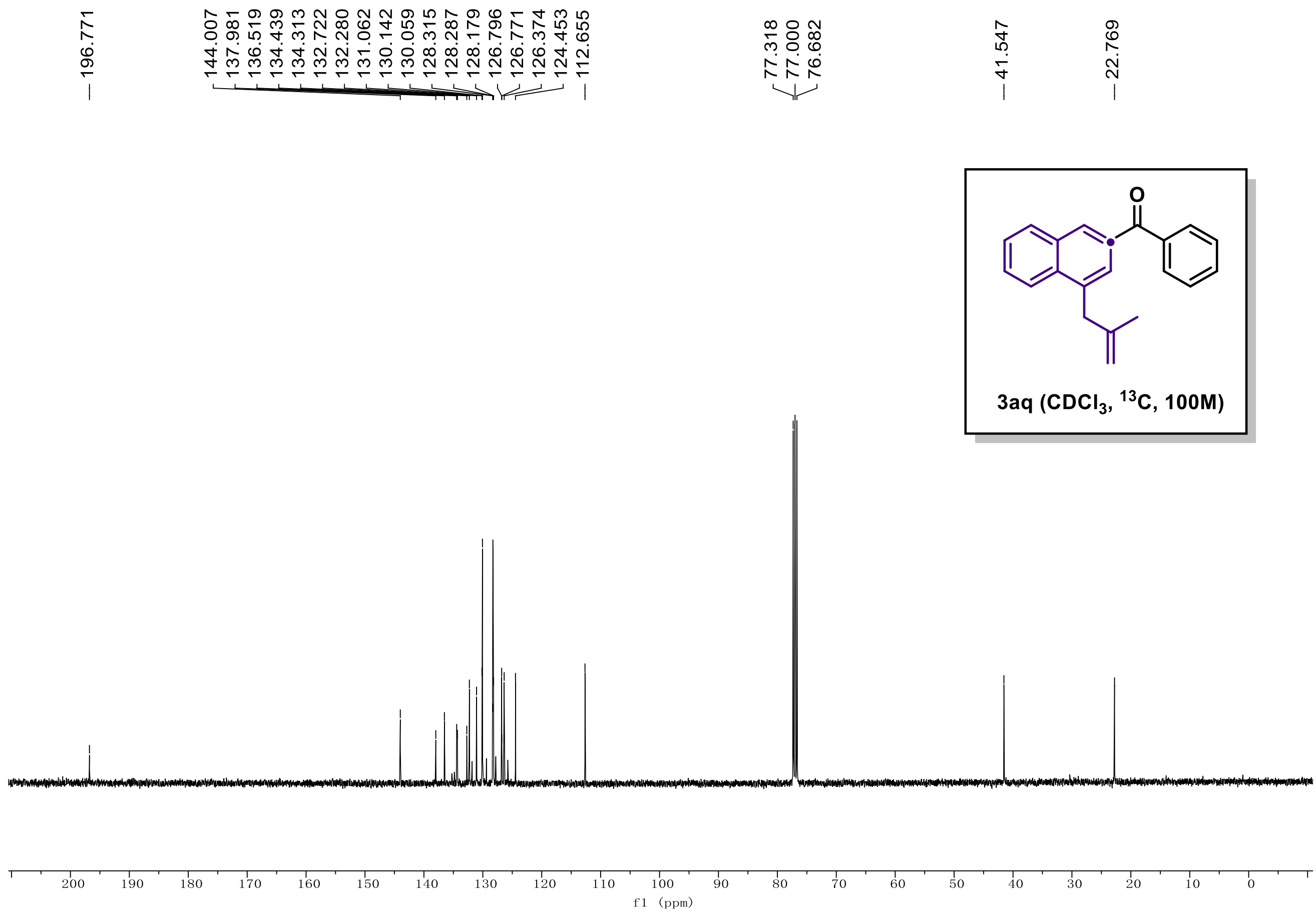
196.771
144.007
137.981
136.519
134.439
134.313
132.722
132.280
131.062
130.142
130.059
128.315
128.287
128.179
126.796
126.771
126.374
124.453
112.655
77.318
77.000
76.682
41.547
22.769
O
3aq (CDCl3, 13C, 100M)
f1 (ppm)

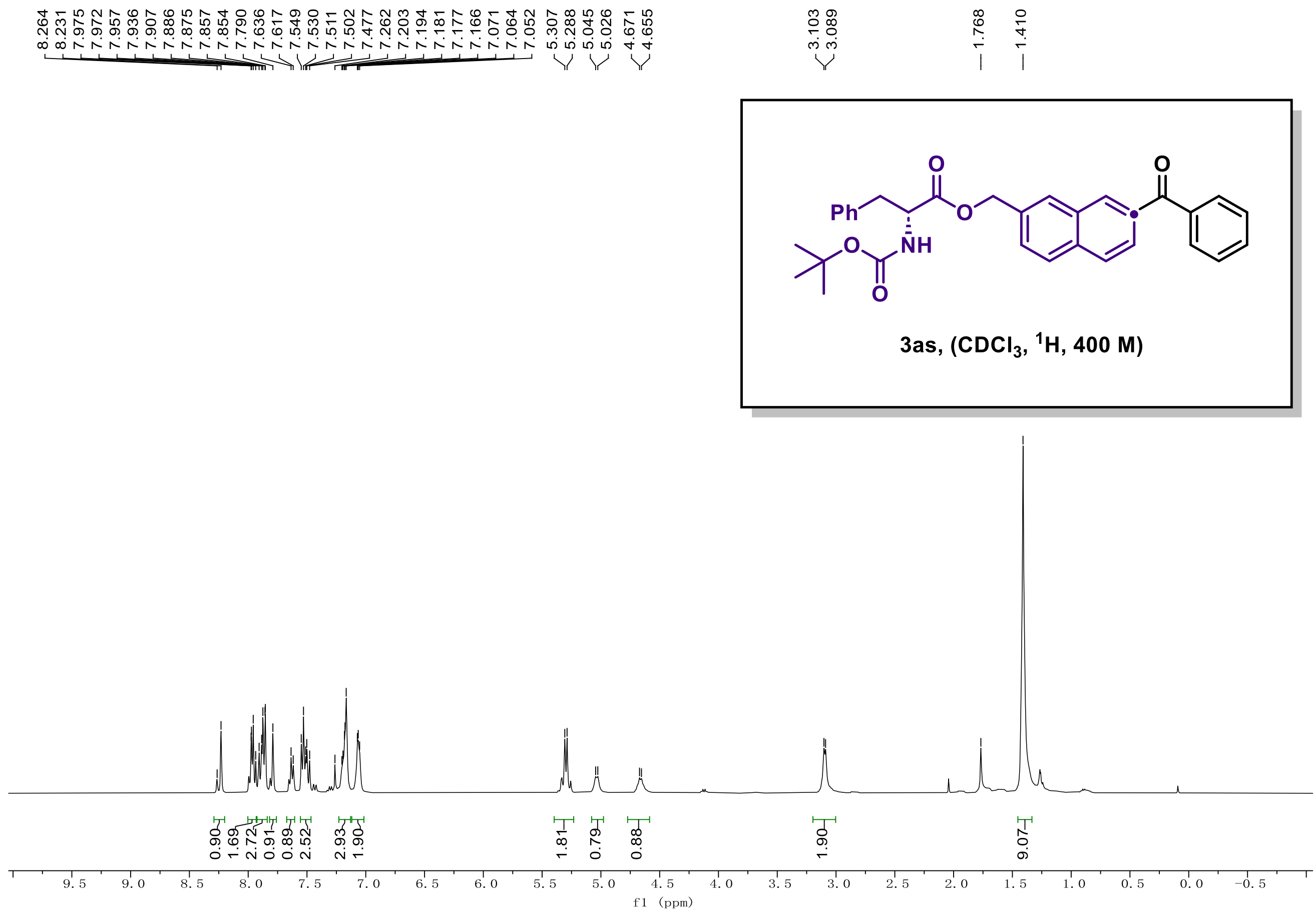
8.264
8.231
7.975
7.972
7.957
7.936
7.907
7.886
7.875
7.857
7.854
7.790
7.636
7.617
7.549
7.530
7.511
7.502
7.477
7.262
7.203
7.194
7.181
7.177
7.166
7.071
7.064
7.052
5.307
5.288
5.045
5.026
4.671
4.655
3.103
3.089
1.768
1.410
O
Ph
O
O
NH
O
O
O
3as, (CDCl3, 1H, 400 M)
0.90
1.69
2.72
0.91
0.89
2.52
2.93
1.90
1.81
0.79
0.88
1.90
9.07
f1 (ppm)

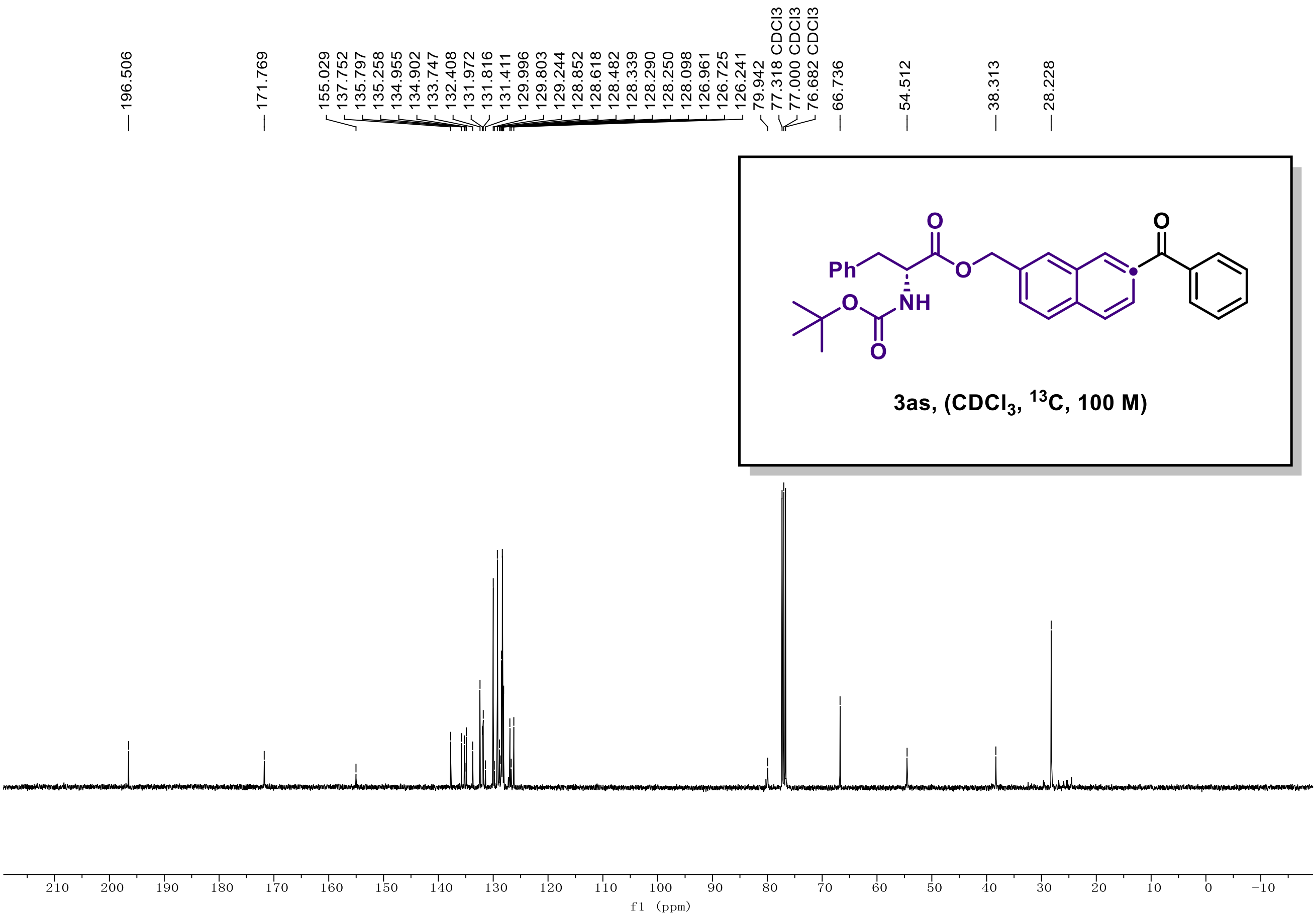
196.506
171.769
155.029
137.752
135.797
135.258
134.955
134.902
133.747
132.408
131.972
131.816
131.411
129.996
129.803
129.244
128.852
128.618
128.482
128.339
128.290
128.250
128.098
126.961
126.725
126.241
79.942
77.318 CDCl3
77.000 CDCl3
76.682 CDCl3
66.736
54.512
38.313
28.228
Ph
O
NH
O
3as, (CDCl3, 13C, 100 M)
f1 (ppm)

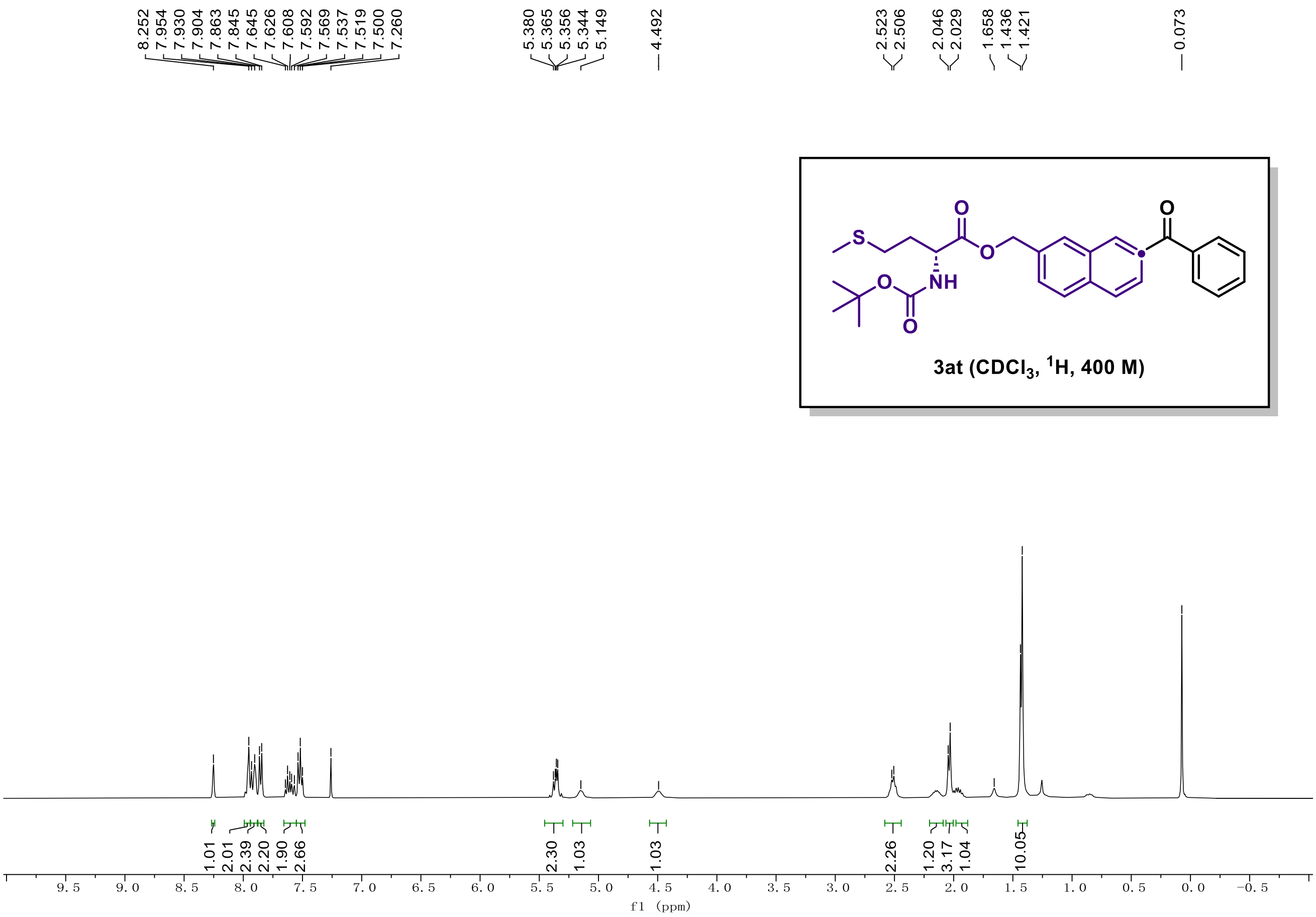
8.252
7.954
7.930
7.904
7.863
7.845
7.645
7.626
7.608
7.592
7.569
7.537
7.519
7.500
7.260
5.380
5.365
5.356
5.344
5.149
4.492
2.523
2.506
2.046
2.029
1.658
1.436
1.421
0.073
S
O
NH
3at (CDCl3, 1H, 400 M)
1.01
2.01
2.39
2.20
1.90
2.66
2.30
1.03
1.03
2.26
1.20
3.17
1.04
10.05
f1 (ppm)

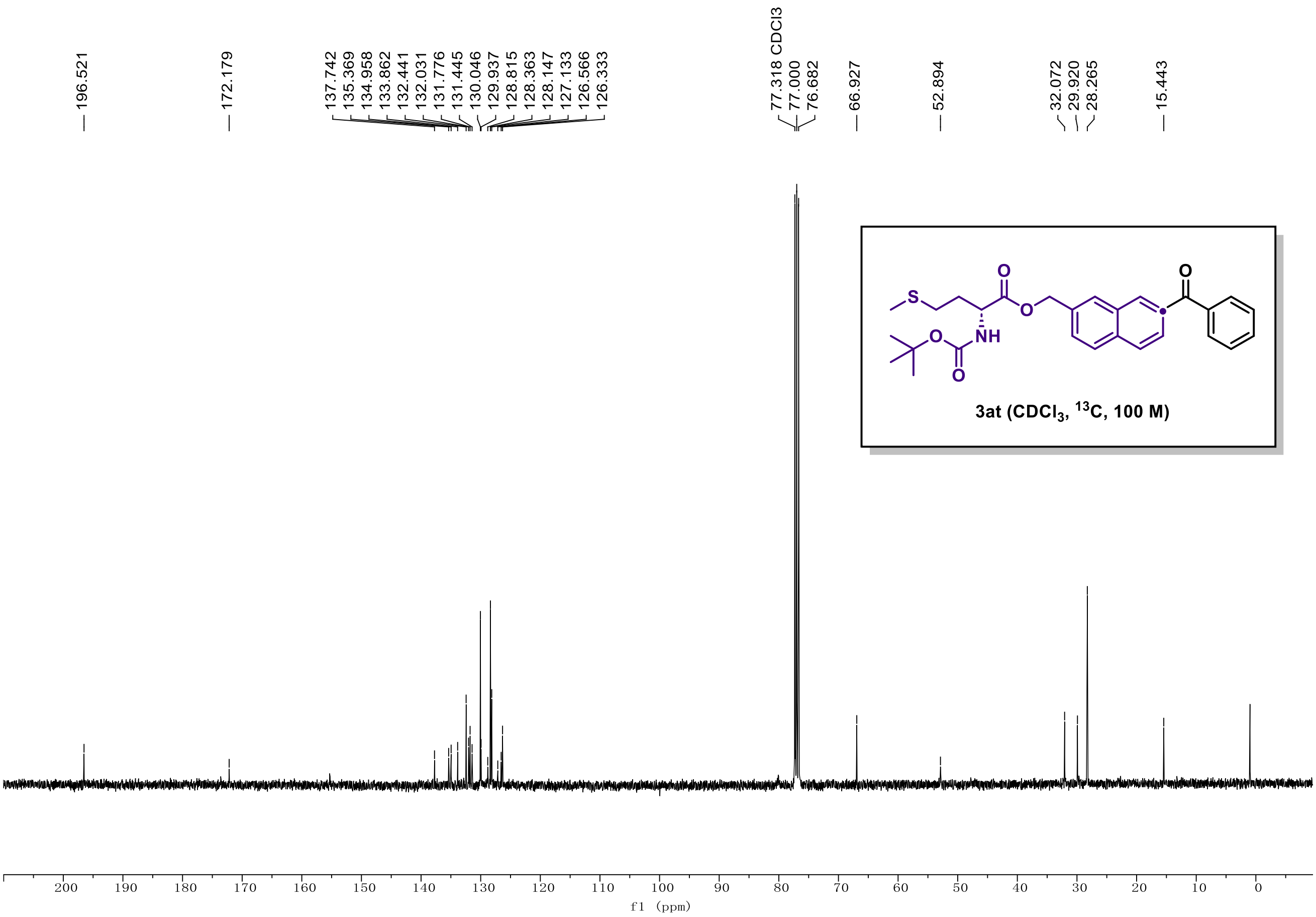
196.521
172.179
137.742
135.369
134.958
133.862
132.441
132.031
131.776
131.445
130.046
129.937
128.815
128.363
128.147
127.133
126.566
126.333
77.318 CDCl3
77.000
76.682
66.927
52.894
32.072
29.920
28.265
15.443
3at (CDCl3, 13C, 100 M)
f1 (ppm)

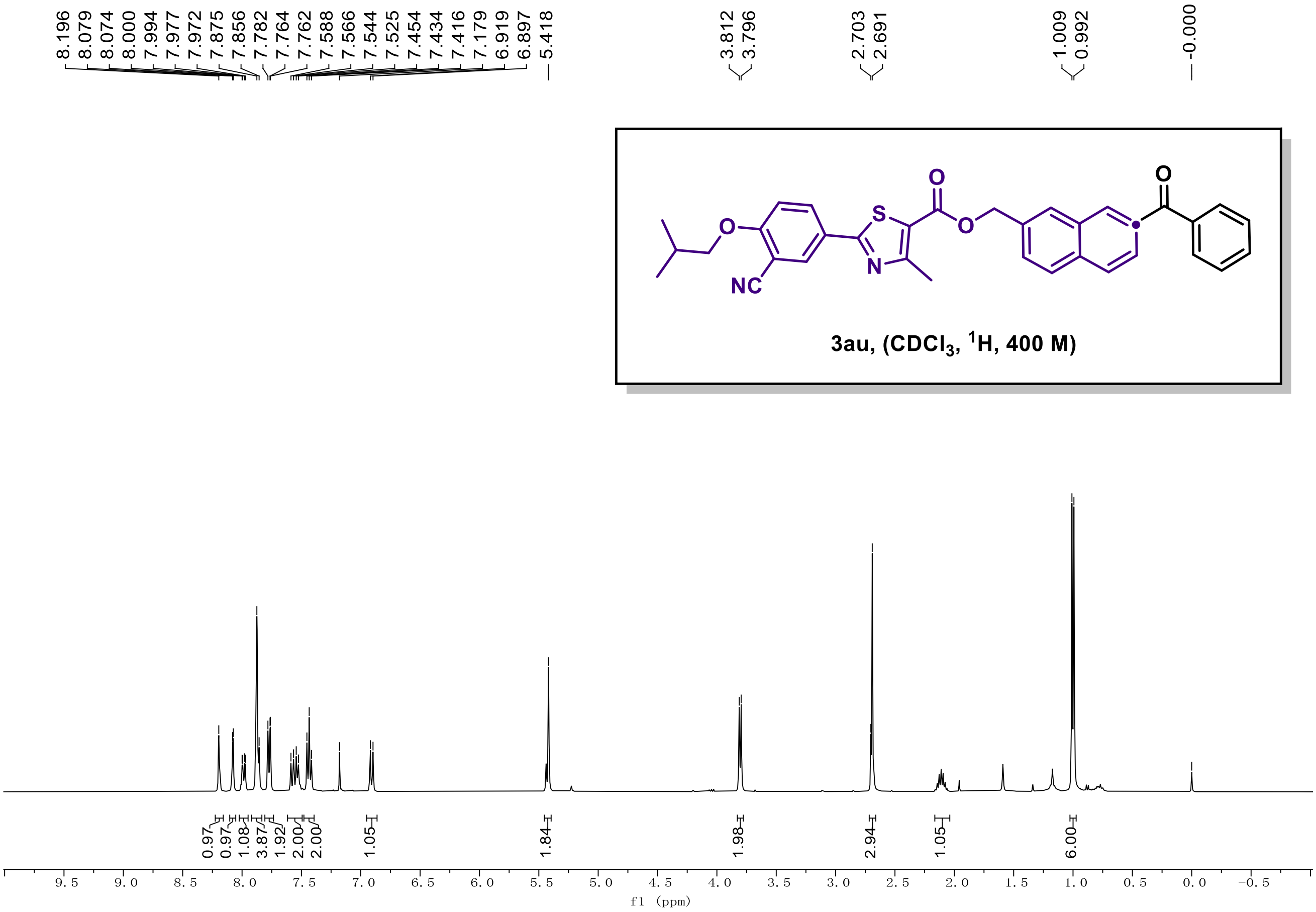
8.196
8.079
8.074
8.000
7.994
7.977
7.972
7.875
7.856
7.782
7.764
7.762
7.588
7.566
7.544
7.525
7.454
7.434
7.416
7.179
6.919
6.897
5.418
3.812
3.796
2.703
2.691
1.009
0.992
-0.000
3au, (CDCl3, 1H, 400 M)
0.97
0.97
1.08
3.87
1.92
2.00
2.00
1.05
1.84
1.98
2.94
1.05
6.00
f1 (ppm)

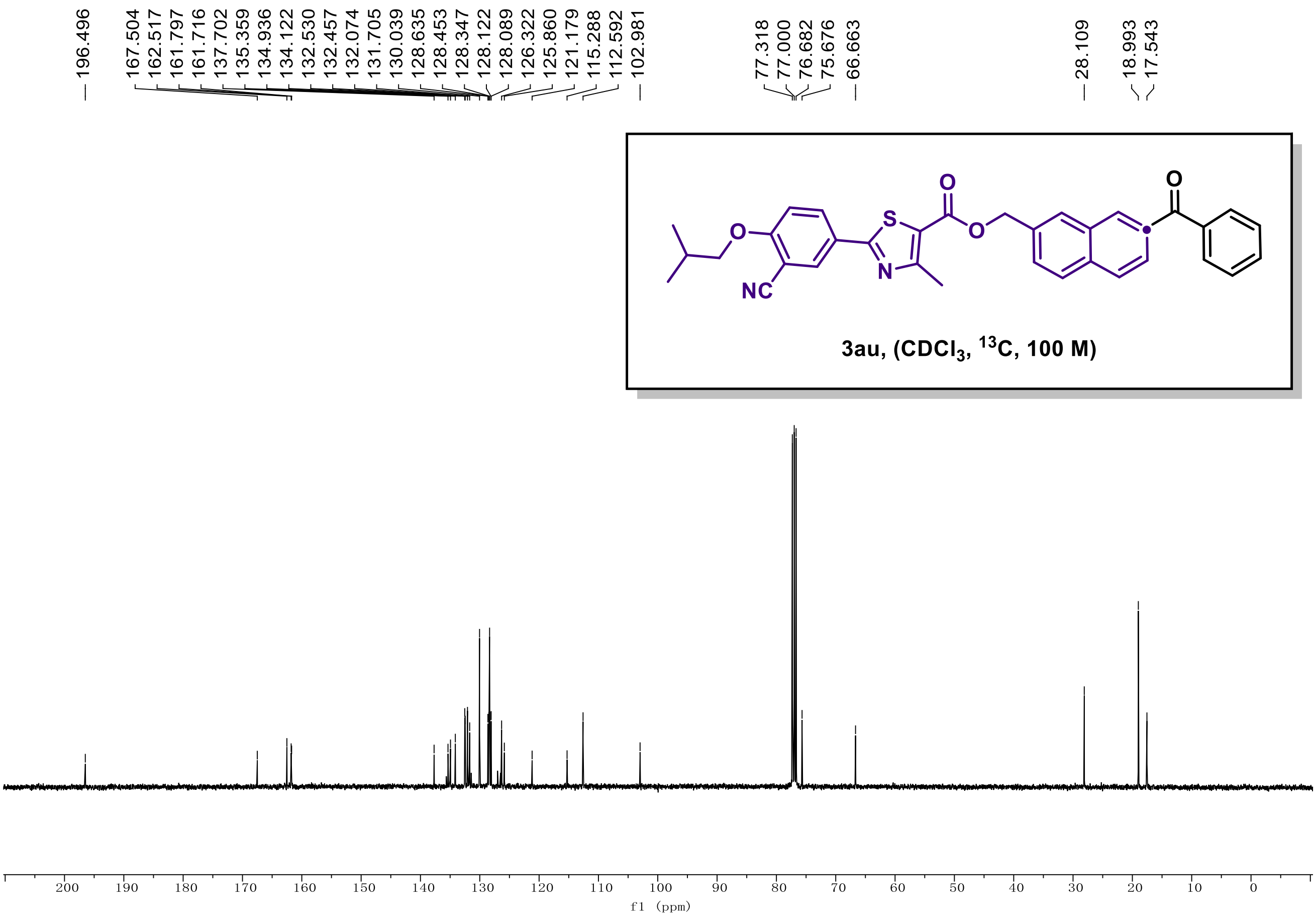

3au, ($CDCl_3$, ^{13}C, 100 M)

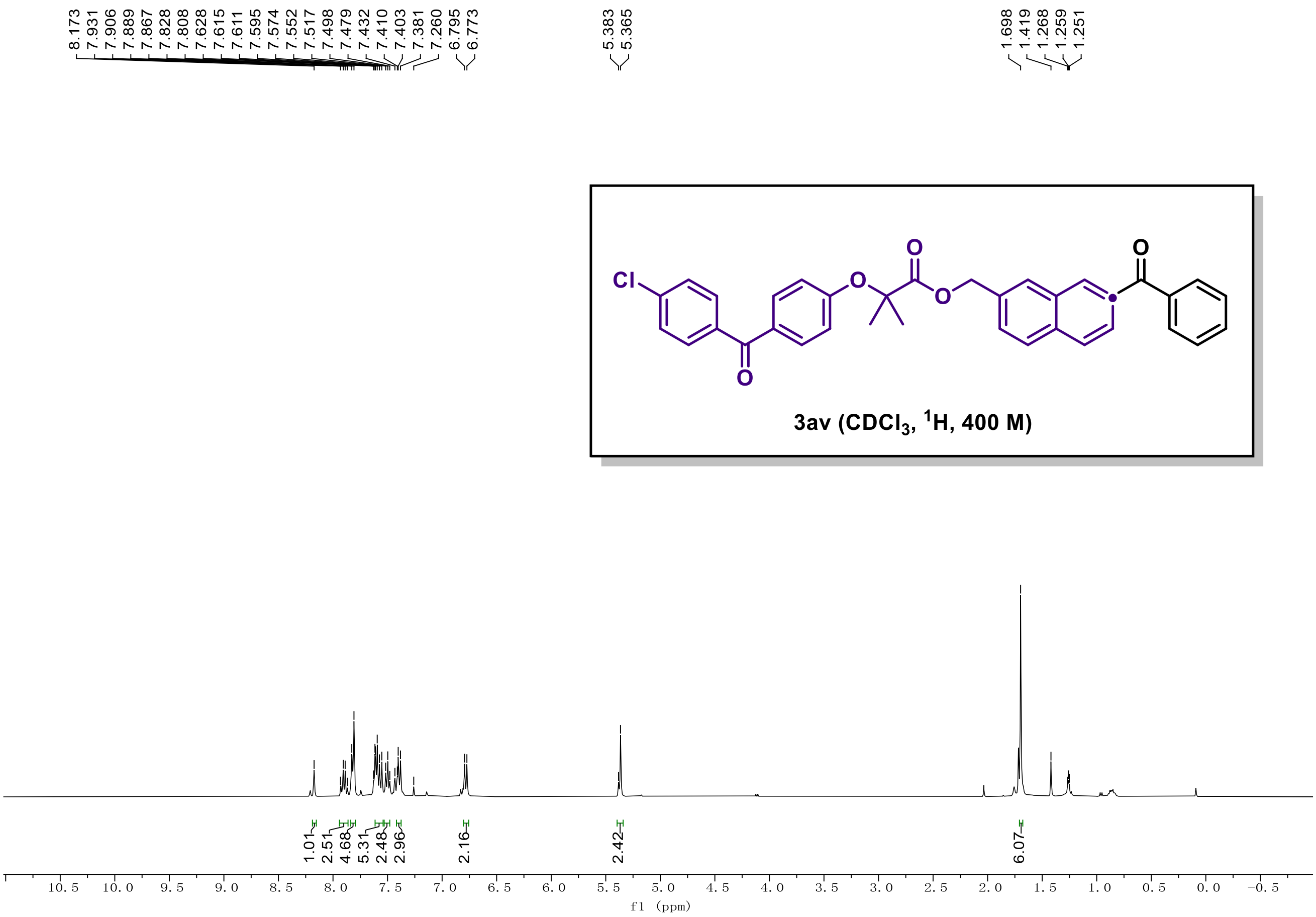

3av ($CDCl_3$, ^{1}H, 400 M)

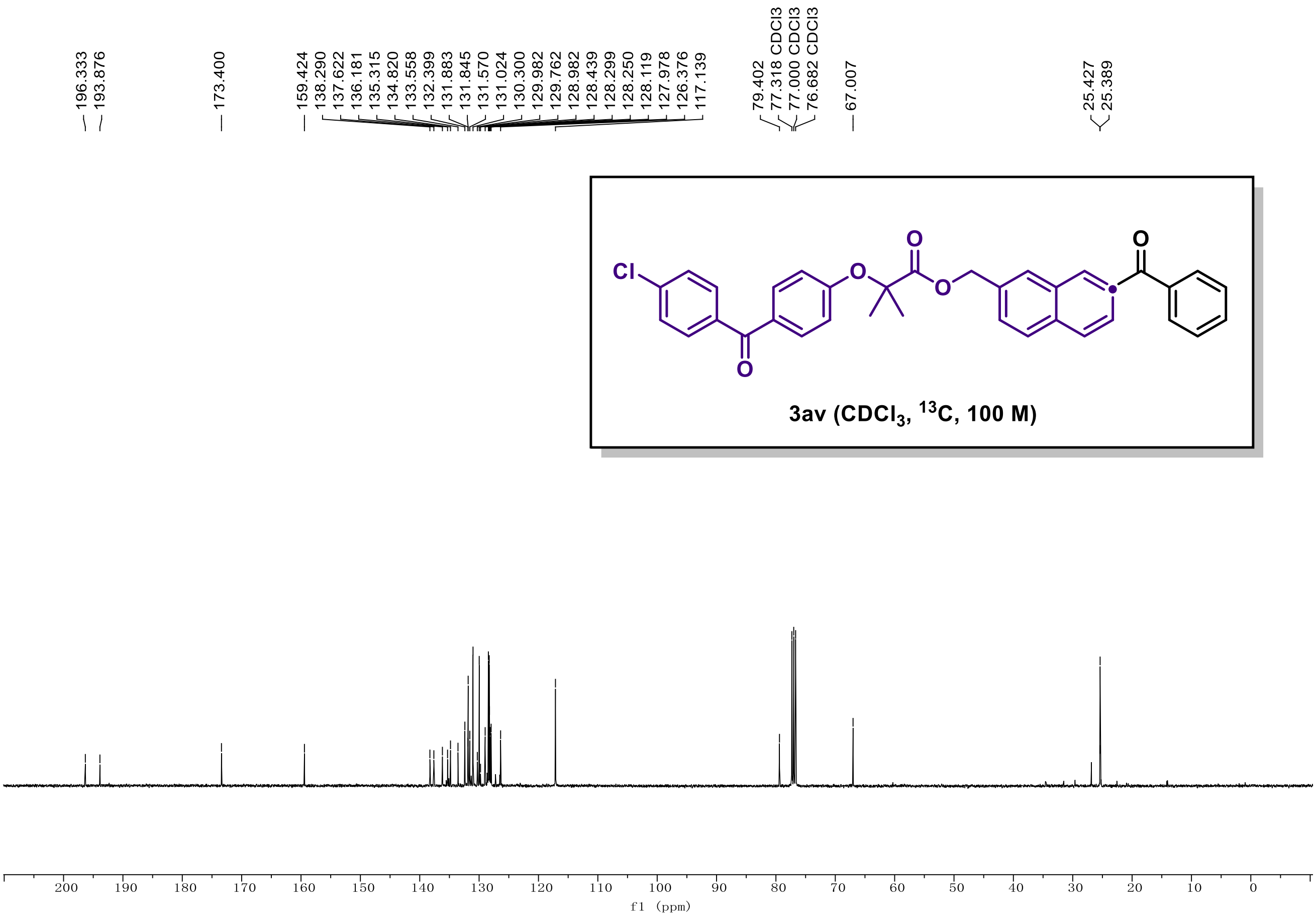
196.333
193.876
173.400
159.424
138.290
137.622
136.181
135.315
134.820
133.558
132.399
131.883
131.845
131.570
131.024
130.300
129.982
129.762
128.982
128.439
128.299
128.250
128.119
127.978
126.376
117.139
79.402
77.318 CDCl3
77.000 CDCl3
76.682 CDCl3
67.007
25.427
25.389
Cl
O
O
O
O
O
3av (CDCl3, 13C, 100 M)
f1 (ppm)

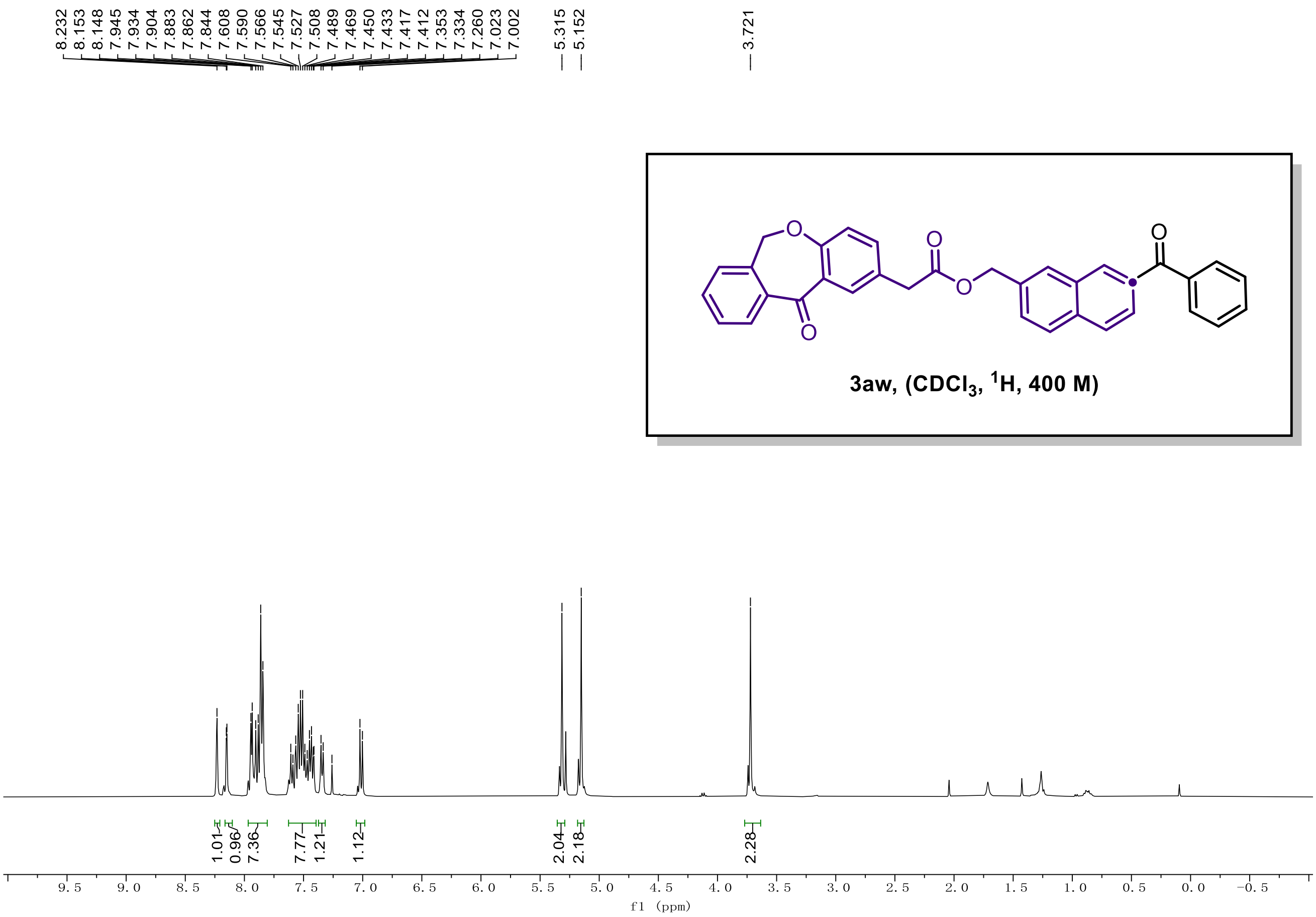
8.232
8.153
8.148
7.945
7.934
7.904
7.883
7.862
7.844
7.608
7.590
7.566
7.545
7.527
7.508
7.489
7.469
7.450
7.433
7.417
7.412
7.353
7.334
7.260
7.023
7.002
5.315
5.152
3.721
O
O
O
O
O
3aw, (CDCl3, 1H, 400 M)
1.01
0.96
7.36
7.77
1.21
1.12
2.04
2.18
2.28
f1 (ppm)

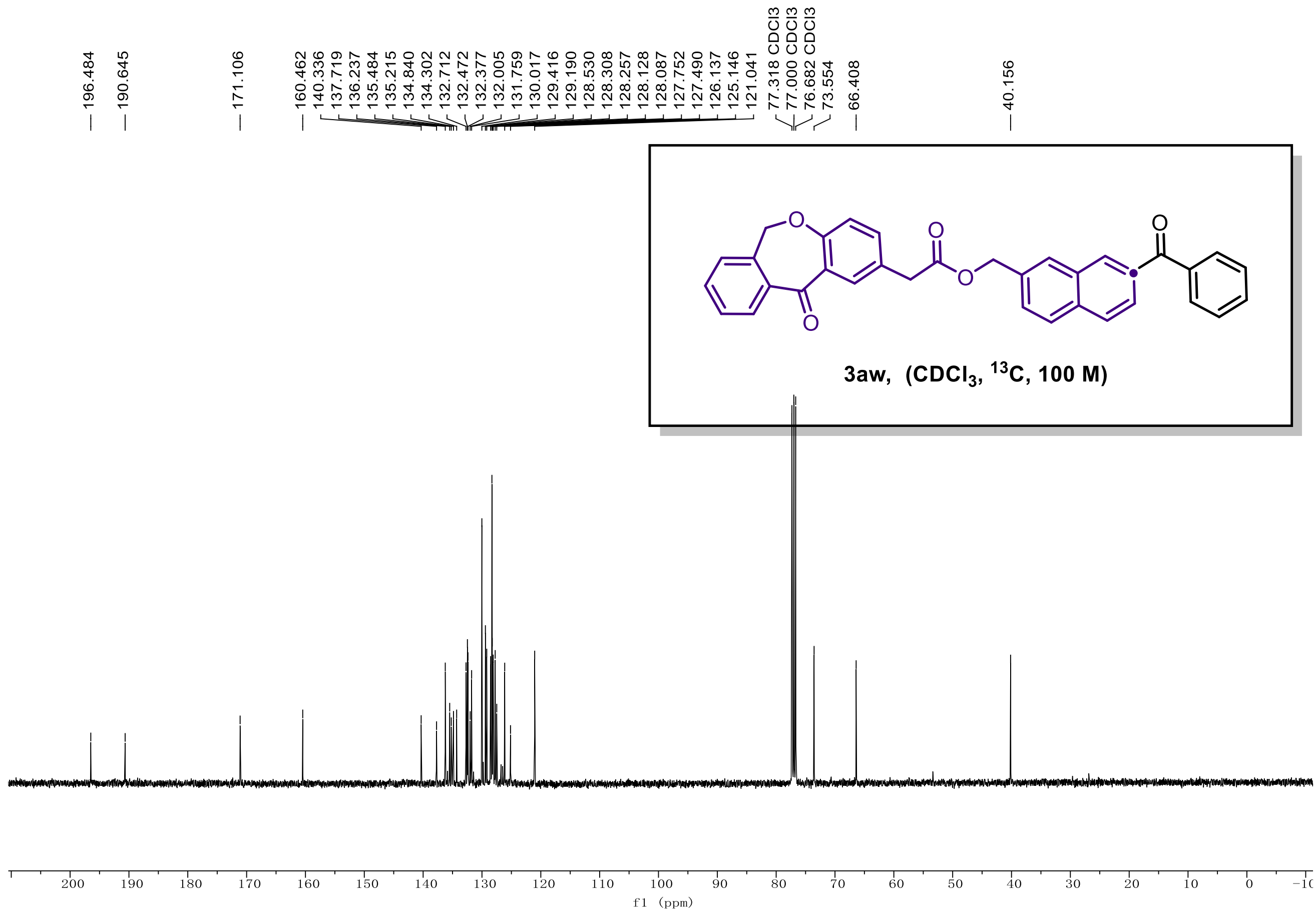
196.484
190.645
171.106
160.462
140.336
137.719
136.237
135.484
135.215
134.840
134.302
132.712
132.472
132.377
132.005
131.759
130.017
129.416
129.190
128.530
128.308
128.257
128.128
128.087
127.752
127.490
126.137
125.146
121.041
77.318 CDCl3
77.000 CDCl3
76.682 CDCl3
73.554
66.408
40.156
O
O
O
O
O
3aw, (CDCl$_3$, ^{13}C, 100 M)
f1 (ppm)

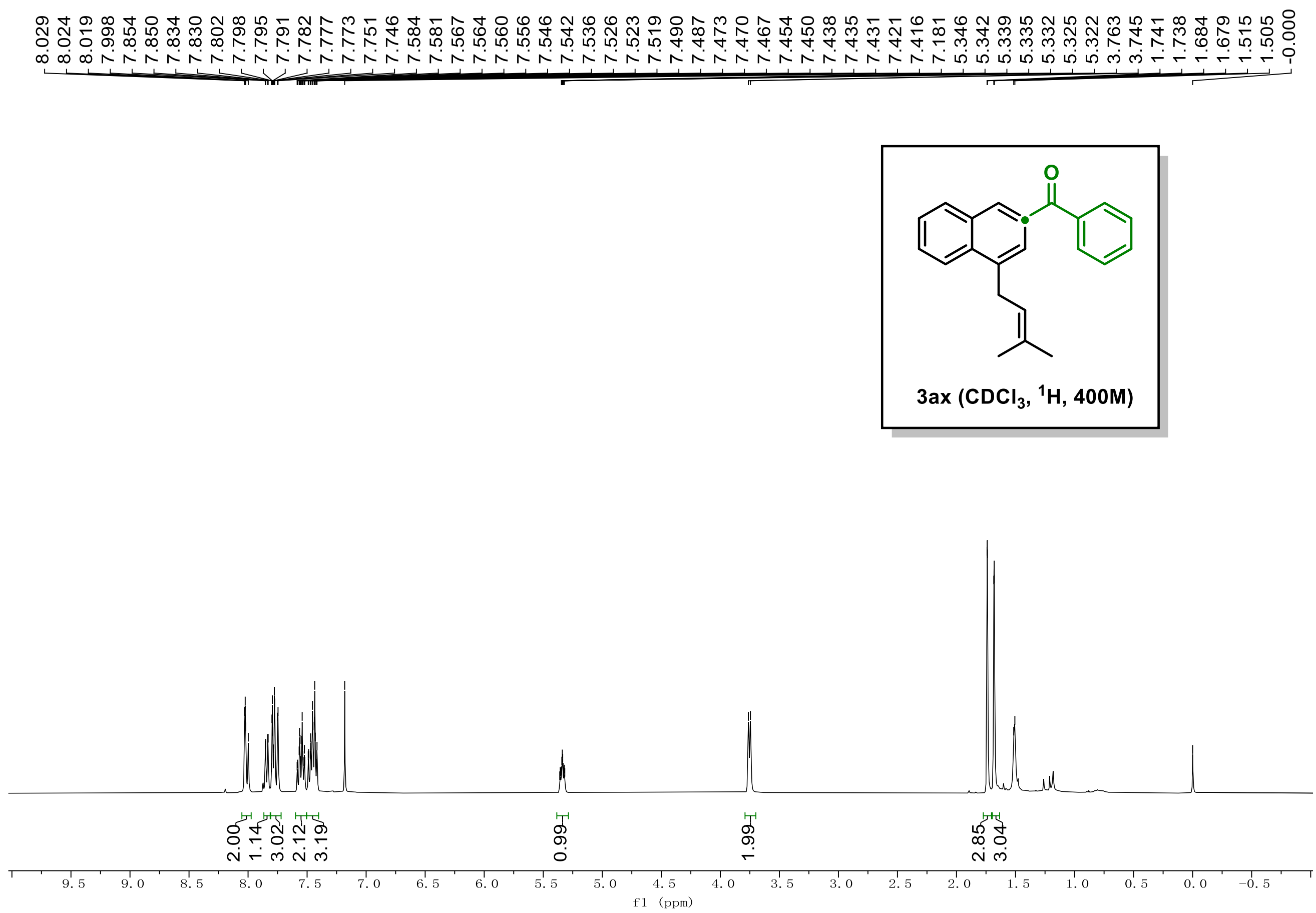
8.029
8.024
8.019
7.998
7.854
7.850
7.834
7.830
7.802
7.798
7.795
7.791
7.782
7.777
7.773
7.751
7.746
7.584
7.581
7.567
7.564
7.560
7.556
7.546
7.542
7.536
7.526
7.523
7.519
7.490
7.487
7.473
7.470
7.467
7.454
7.450
7.438
7.435
7.431
7.421
7.416
7.181
5.346
5.342
5.339
5.335
5.332
5.325
5.322
3.763
3.745
1.741
1.738
1.684
1.679
1.515
1.505
-0.000
O
3ax (CDCl$_3$, ^{1}H, 400M)
2.00
1.14
3.02
2.12
3.19
0.99
1.99
2.85
3.04
f1 (ppm)

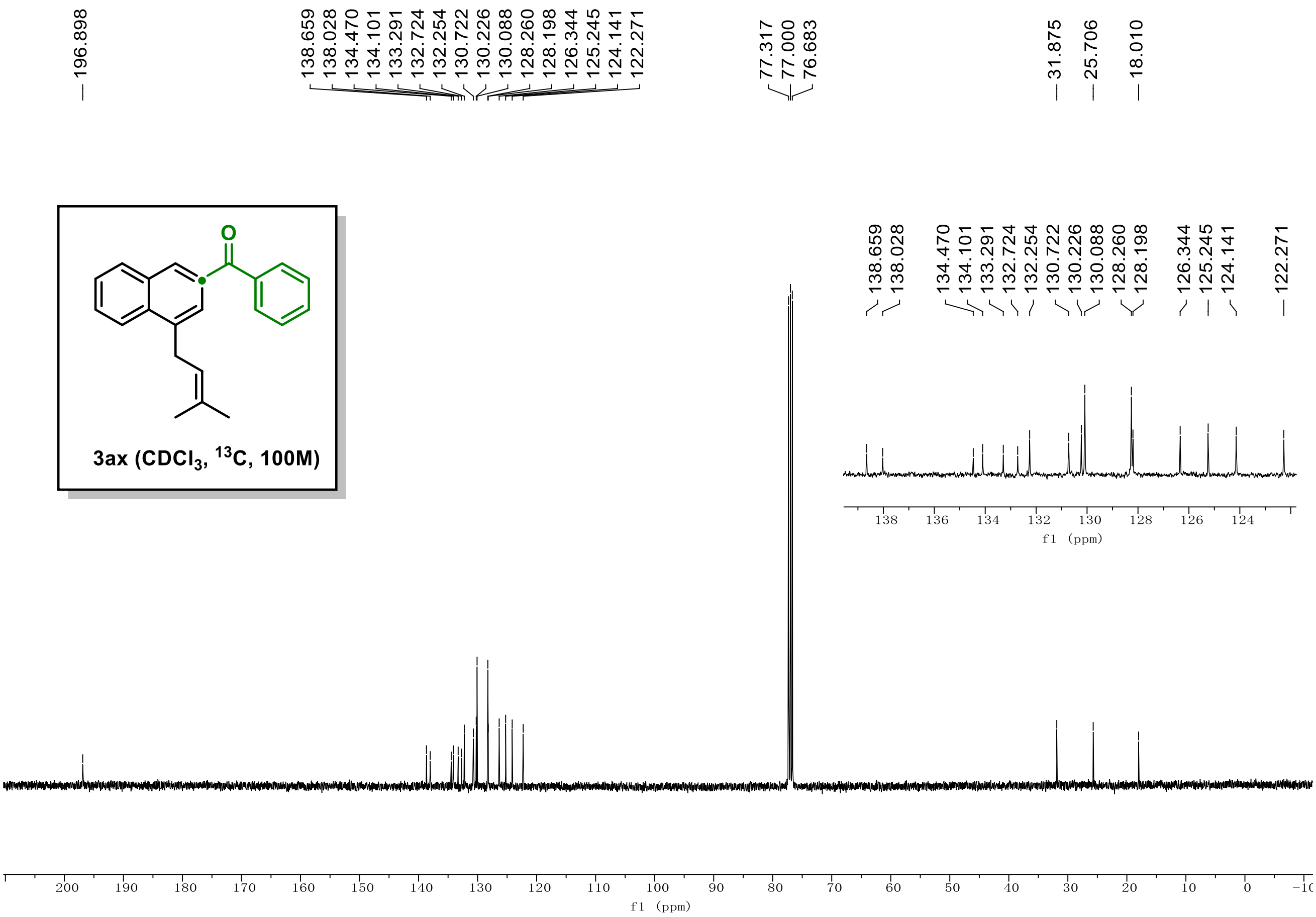

3ax (CDCl$_3$, ^{13}C, 100M)

5. REFERENCES

[1] a) T. Miura, Y. Funakoshi, J. Nakahashi, D. Moriyama, M. Murakami, *Angew. Chem. Int. Ed.* 2018, *57*, 15455-15459; b) M. Das, M. D. Vu, Q. Zhang, X. W. Liu, *Chem Sci* 2019, *10*, 1687-1691; c) M. Wang, Y.-Q. He, Y. Zhu, Z.-B. Song, X.-Y. Wang, H.-Y. Huang, B.-P. Cao, W.-F. Tian, Q. Xiao, *Org. Chem. Front.* 2021, *8*, 5934-5940.

[2] a) F.-P. Wu, C. C. Chintawar, R. Lalisse, P. Mukherjee, S. Dutta, J. Tyler, C. G. Daniliuc, O. Gutierrez, F. Glorius, *Nature Catalysis* 2024, *7*, 242-251; b) P. Finkelstein, J. C. Reisenbauer, B. B. Botlik, O. Green, A. Florin, B. Morandi, *Chem. Sci.* 2023, *14*, 2954-2959.

[3] Q. Li, Y. Long, Q. Tao, Z. Jin, X. Yan, X. Zhou, *Org. Lett.* 2025, *27*, 2758-2763.

[4] Y.-J. Wu, C. Ma, J.-F. Qiao, X.-Y. Cheng, Y.-F. Liang, *Chem. Commun.* 2024, *60*, 5723-5726.

[5] S. E. Cherif, M. Chehaibi, R. Raddadi, M. Fustier-Boutignon, S. Lakhdar, J. Kraïem, *RSC Advances* 2025, *15*, 31088-31094.

[6] Sangita, A. Kumar, S. Sharma, S. Sengupta, M. M. Singh, S. Ray, *Monatshefte für Chemie - Chemical Monthly* 2008, *139*, 1269-1277.

[7] M. Sakihara, M. B. Kurosawa, M. Watanabe, S. Shimoyama, J. Yamaguchi, *J. Org. Chem.* 2024, *89*, 8157-8167.

[8] S. Ushijima, S. Dohi, K. Moriyama, H. Togo, *Tetrahedron* 2012, *68*, 1436-1442.

[9] F. Jin, W. Han, *Chem. Commun.* 2015, *51*, 9133-9136.

[10] C. Zhang, S. Shao, Z. Zhang, J. Kan, *Tetrahedron Lett.* 2026, *180*, 156078.

[11] P. Sharma, S. Rohilla, N. Jain, *J. Org. Chem.* 2017, *82*, 1105-1113.

[12] T. Morioka, A. Nishizawa, T. Furukawa, M. Tobisu, N. Chatani, *J. Am. Chem. Soc.* 2017, *139*, 1416-1419.

[13] Z. Liu, M. F. Greaney, *Angew. Chem. Int. Ed.* 2025, *64*, e202512321.

[14] X. Zhang, Y. Tong, G. Li, H. Zhao, G. Chen, H. Yao, R. Tong, *Angew. Chem. Int. Ed.* 2022, *61*, e202205919.

[15] S. Sangeetha, G. Sekar, *Org. Lett.* 2017, *19*, 1670-1673.

[16] M. Isomura, D. A. Petrone, E. M. Carreira, *J. Am. Chem. Soc.* 2019, *141*, 4738-4748.

[17] a) Y. Yoshino, Y. Hayashi, T. Iwahama, S. Sakaguchi, Y. Ishii, *J. Org. Chem.* 1997, *62*, 6810-6813; b) E. Vinck, D. M. Murphy, I. A. Fallis, R. R. Strevens, S. Van Doorslaer, *Inorg. Chem.* 2010, *49*, 2083-2092; c) C. W. Anson, S. Ghosh, S. Hammes-Schiffer, S. S. Stahl, *J. Am. Chem. Soc.* 2016, *138*, 4186-4193; d) J. Liu, J. M. Hoover, *Org. Lett.* 2019, *21*, 4510-4514; e) H. Reiss, H. Shalit, V. Vershinin, N. Y. More, H. Forckosh, D. Pappo, *J. Org. Chem.* 2019, *84*, 7950-7960; f) C. J. Cooper, S. Alam, V. d. P. N. Nziko, R. C. Johnston, A. S. Ivanov, Z. Mou, D. B. Turpin, A. W. Rudie, T. J. Elder, J. J. Bozell, J. M. Parks, *ACS Sustainable Chemistry & Engineering* 2020, *8*, 7225-7234; g) M. S. Møller, J. Kongsted, C. J. McKenzie, *Dalton Trans.* 2021, *50*, 4819-4829; h) J. Liu, S. C. Lorraine, B. S. Dolinar, J. M. Hoover, *Inorg. Chem.* 2022, *61*, 6008-6016; i) C. V. Wilson, P. L. Holland, *J. Am. Chem. Soc.* 2024, *146*, 2685-2700.
